# Supplementary material for: Under-reported relationship: a comparative study of pharmaceutical industry and patient organisation payment disclosures in the UK (2012–2016)
Source: BMJ Open. 2020 Sep 19;10(9):e037351. doi: 10.1136/bmjopen-2020-037351 (PMC7511620; doi:10.1136/bmjopen-2020-037351)
Supplement: Supplementary data [file bmjopen-2020-037351supp001.pdf]

Web supplement 1 – Research process: pharmaceutical industry data

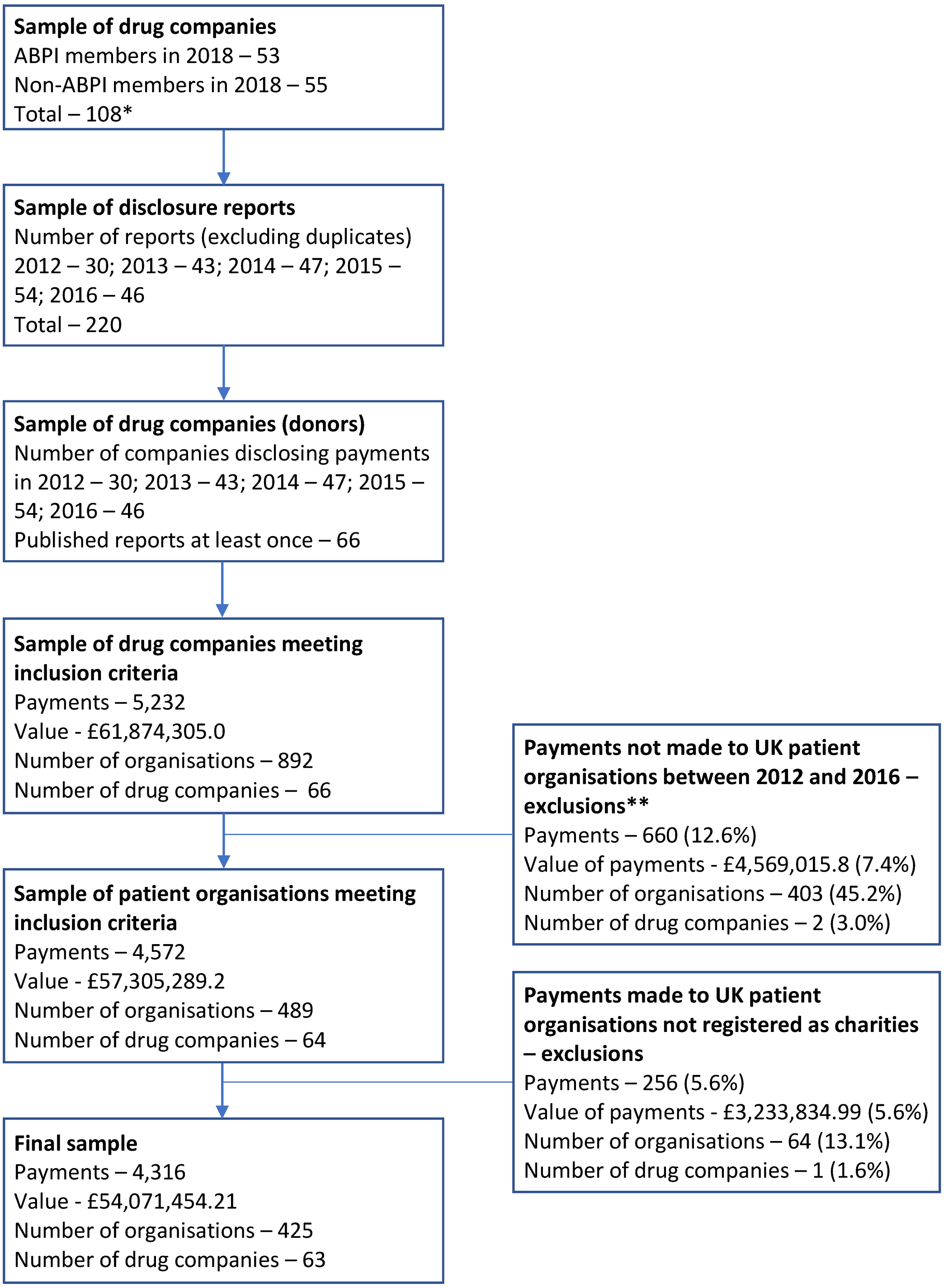

*Complete lists of companies provided in Table 1

**Detailed breakdown provided in Table 2

Table 1 Sample of drug companies – ABPI and non-ABPI members

ABPI member in 2018 – n=53	Non-ABPI member in 2018 – n=55
A. Menarini	Accretio
Abbvie	Actavis
Actelion	Aegerion
Alexion	Aguettant
Alimera Sciences	Alcon
ALK-Abelo	AMCo
Allergan	ApoPharma
Alliance	Astellas
Almirall	Baxalta
Amgen	Baxter
AstraZeneca	Besins
Bausch and Lomb	BGP Products
Bayer	Bio Products Laboratory
Biogen	Biotest
BioMarin	Bracco
Bristol-Myers Squibb	Britannia
Boehringer Ingelheim	CEB Pharma
Celgene	Consilient Health
Chiesi	CSL Behring
Chugai	Dermal
Daiichi Sankyo	Diurnal
Eisai	Ferring
Lilly	Flynn Pharma
Fresenius Medical Care	Fresenius Kabi
Grünenthal	Galen
GSK	Gedeon Richter UK
Ipsen	Genzyme Therapeutics
Janssen	Gilead
LEO Pharma	Guerbet
Lundbeck	Hospira UK
Merck	HRA Pharma
Mitsubishi Tanabe Pharma	Jazz Pharmaceuticals
MSD	Martindale Pharma
Napp Pharmaceuticals	Meda Pharmaceuticals
Norgine	Merz Pharma
Novartis	Mundipharma International
Novo Nordisk	Nicovations
Orion Pharma	Octapharma
Otsuka Pharmaceuticals	Orphan Europe
Pfizer	Profile Pharma

Pharma Mar	PTC Therapeutics
Pierre Fabre Ltd	RB
Quintiles Commercial (Novex)	Rosemont Pharmaceuticals
Roche Products	Sandoz
Sanofi	Sanofi Pasteur
Santen	Sigma Tau Rare Diseases
Servier Laboratories	Sobi
Shionogi Europe	Special Products
Shire	STD
Sunovion Pharmaceuticals	Stirling Anglian
Takeda	Syner-Med
UCB	Teva
Vifor Fresenius Medical Care Renal Pharma	Thea Pharmaceuticals
	Tillotts
	Vifor Pharma Group

Table 2 Breakdown of exclusions - payments not made to UK patient organisations between 2012 and 2016

Reason for exclusion	Number of payments (%)	Value of payments - £ (%)	Number of organisations (%)
Outside period of observation	4 (0.6)	10,039.8 (0.2)	0 (0.0)
Not an organisation	14 (2.1)	94,736.1 (2.1)	0 (0.0)
More than one organisation	17 (2.6)	354,390.0 (7.8)	0 (0.0)
Organisation - nature unclear	57 (8.6)	397,240.6 (8.7)	39 (9.7)
Not UK organisation	54 (8.2)	716,044.0 (15.7)	30 (7.4)
Excluded UK organisations			
Public sector – state organisation	12 (1.8)	18,629.7 (0.4)	7 (1.7)
Public healthcare provider	242 (36.7)	726,401.4 (15.9)	176 (43.7)
Private sector organisation	47 (7.1)	981,886.2 (21.5)	33 (8.2)
University	14 (2.1)	86,670.3 (1.9)	7 (1.7)
Professional organisation (third sector)	143 (21.7)	984,935.1 (21.6)	73 (18.1)
Community interest company or social enterprise - healthcare provider (third sector)	3 (0.5)	2,690.7 (0.1)	3 (0.7)
Healthcare provider (third sector)	20 (3.0)	46,522.0 (1.0)	10 (2.5)
Not focusing directly on health (third sector)	30 (4.5)	148,396.5 (3.2)	24 (6.0)
Community interest company or social enterprise - not focusing directly on health (third sector)	3 (0.5)	433.4 (0.0)	1 (0.2)
Total	660 (100)	4,569,015.8	403 (100)
