# Supplementary material for: Under-reported relationship: a comparative study of pharmaceutical industry and patient organisation payment disclosures in the UK (2012–2016)
Source: BMJ Open. 2020 Sep 19;10(9):e037351. doi: 10.1136/bmjopen-2020-037351 (PMC7511620; doi:10.1136/bmjopen-2020-037351)
Supplement: Supplementary data [file bmjopen-2020-037351supp002.pdf]

Web supplement 2 Research process: patient organisation data

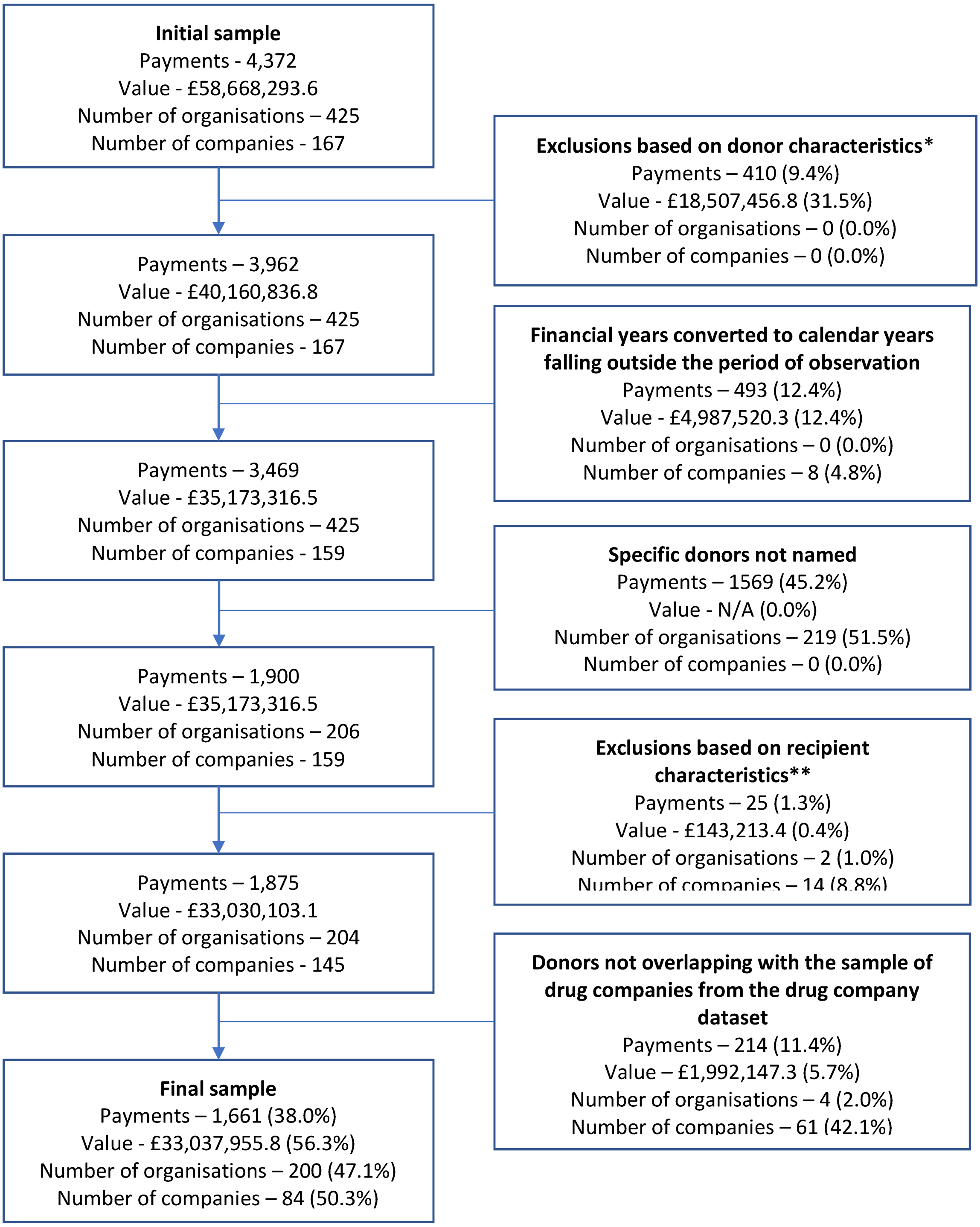

*More detailed breakdown in Table 3

**More detailed breakdown in Table 4

Table 3 Exclusions based on donor characteristics – detailed breakdown

Reason for exclusion	Number of Payments excluded	Value of payments excluded (£)
Exclusions unique to patient organisation dataset		
Regional branch (same charity number as national branch)	229	13,213,514.2
Subsumed under a larger organisation (same charity number as larger organisation)	125	291,862.9
Years before merger	14	1,515.0
Years after merger	9	-
Exclusions resulting from exclusions in drug company dataset		
Third sector - healthcare provider	6	-
Third sector - professional organisation	6	-
Third sector - not focusing directly on health	15	5,000,564.8
Not a charity	6	-
Total	410	18,507,456.8

Table 4 Exclusions based on recipient characteristics – detailed breakdown

	Number of Payments excluded	Value of payments excluded (£)	Number of drug companies excluded	Number of patient organisations excluded
More than one organisation	8	58,485.5	7	0
Not a pharmaceutical company	17	84,727.8	7	2
Total	25	143,213.4	14	2
