# Supplementary material for: Under-reported relationship: a comparative study of pharmaceutical industry and patient organisation payment disclosures in the UK (2012–2016)
Source: BMJ Open. 2020 Sep 19;10(9):e037351. doi: 10.1136/bmjopen-2020-037351 (PMC7511620; doi:10.1136/bmjopen-2020-037351)
Supplement: Supplementary data [file bmjopen-2020-037351supp003.pdf]

This online dataset comprises web supplements (WS) accompanying the paper entitled “An underreported re

Web Supplement 3. Donors - Absolute and relative differences in the number and value of payments (all ye

NUMBER OF PAYMENTS

Number of payments - industry data
Number of payments - patient organisation data
Number of drug companies with at least one payment - industry data
Number of drug companies with at least one payment - patient organisation data

NUMBER OF PAYMENTS - THRESHOLDS

Threshold 0: Number of drug companies with at least 1 one payment in at least one dataset (1 if condition me
Threshold 1: Number of drug companies with more than 1 one payment in at least one dataset (1 if condition
Threshold 2: Number of drug companies with more than 10 payments in at least one dataset (1 if condition m
Threshold 3: Number of drug companies with more than 100 payments in at least one dataset (1 if condition

NUMBER OF PAYMENTS - ABSOLUTE DIFFERENCES

Number of payments - threshold 0
Number of drug companies with exact match in both datasets
Number of drug companies with more paymens t in industry data
Number of drug companies with more payments in patient organisation data

Highest absolute difference between patient organisation and industry data - number of payments higher in i
Highest absolute difference between patient organisation and industry data - number of payments higher in p

Number of payments - threshold 1
Number of drug companies with exact match in both datasets
Number of drug companies with more paymens t in industry data
Number of drug companies with more payments in patient organisation data

Highest absolute difference between patient organisation and industry data - number of payments higher in i
Highest absolute difference between patient organisation and industry data - number of payments higher in p

Number of payments - threshold 2
Number of drug companies with exact match in both datasets
Number of drug companies with more paymens t in industry data
Number of drug companies with more payments in patient organisation data

Highest absolute difference between patient organisation and industry data - number of payments higher in i
Highest absolute difference between patient organisation and industry data - number of payments higher in p

Number of payments - threshold 3
Number of drug companies with exact match in both datasets
Number of drug companies with more paymens t in industry data

Number of drug companies with more payments in patient organisation data

Highest absolute difference between patient organisation and industry data - number of payments higher in i
Highest absolute difference between patient organisation and industry data - number of payments higher in p

NUMBER OF PAYMENTS - RELATIVE DIFFERENCES

Relative difference <10%
Relative difference <20%
Relative difference <50%
Relative difference = 100%

Number of payments - threshold 1
Relative difference <10%
Relative difference <20%
Relative difference <50%
Relative difference = 100%

Number of payments - threshold 2
Relative difference <10%
Relative difference <20%
Relative difference <50%
Relative difference = 100%

Number of payments - threshold 3
Relative difference <10%
Relative difference <20%
Relative difference <50%
Relative difference = 100%

VALUE OF PAYMENTS - ABSOLUTE DIFFERENCES

Value of payments - industry data (2016 £)
Value of payments - patient organisation data (2016 £)
Number of payments with value >£0 - industry data
Number of payments with value >£0 - patient organisation data
Number of drug companies with at least one payment with the value >£0 - industry data
Number of drug companies with at least one payment with the value >£0 - patient organisation data

VALUE OF PAYMENTS - THRESHOLDS

Threshold 1: Number of drug companies with at least one payment with value >£0 in at least one dataset (1 i
Threshold 2: Number of drug companies with payments with the value of more than £10,000 in at least one d
Threshold 3: Number of drug companies with payments with the value of more than £100,000 in at least one

VALUE OF PAYMENTS - ABSOLUTE DIFFERENCES

Number of payments - threshold 1

Number of drug companies with exact match in both datasets
Number of drug companies with higher payment value in industry data
Number of drug companies with higher payment value in patient organisation data

Highest absolute difference between patient organisation and industry data - number of payments higher in i
Highest absolute difference between patient organisation and industry data - number of payments higher in [illegible]

Number of payments - threshold 2
Number of drug companies with exact match in both datasets
Number of drug companies with higher payment value in industry data
Number of drug companies with higher payment value in patient organisation data

Highest absolute difference between patient organisation and industry data - number of payments higher in i
Highest absolute difference between patient organisation and industry data - number of payments higher in [illegible]

Number of payments - threshold 3
Number of drug companies with exact match in both datasets
Number of drug companies with higher payment value in industry data
Number of drug companies with higher payment value in patient organisation data

Highest absolute difference between patient organisation and industry data - number of payments higher in i
Highest absolute difference between patient organisation and industry data - number of payments higher in [illegible]

VALUE OF PAYMENTS - RELATIVE DIFFERENCES

Relative difference <10%
Relative difference <20%
Relative difference <50%
Relative difference = 100%

Number of payments - threshold 1
Relative difference <10%
Relative difference <20%
Relative difference <50%
Relative difference = 100%

Number of payments - threshold 2
Relative difference <10%
Relative difference <20%
Relative difference <50%
Relative difference = 100%

Number of payments - threshold 3
Relative difference <10%
Relative difference <20%

Relative difference <50%
Relative difference = 100%

		**A. Menarini**	**Abbvie**	**Actavis**	**Actelion**
		1	1	0	1
Total					
4316		3	281	5	38
1661		0	84	5	23
63		1	1	1	1
84		0	1	1	1
Number of drug c	% of all drug companies with at least one payment in at least one dataset				
87	100.0%	1	1	1	1
80	92.0%	1	1	1	1
47	54.0%		1		1
10	11.5%		1		
Number of drug c	% of all drug companies at a given threshold				
3	3.4%			1	
49	56.3%	1	1		1
35	40.2%				
Number of payments					
757		3	197		15
53					
Number of drug c	% of all drug companies at a given threshold				
3	3.8%			1	
49	61.3%	1	1		1
28	35.0%				
Number of payments					
757		3	197		15
53					
Number of drug c	% of all drug companies at a given threshold				
1	2.1%				
36	76.6%		1		1
10	21.3%				
Number of payments					
757			197		15
53					
Number of drug c	% of all drug companies at a given threshold				
0	0.0%				
10	100.0%		1		

0	0.0%				
Number of payments					
757			197		
0					
Number of drug c	% of all drug companies				
5	5.7%			0.0%	
9	10.3%			0.0%	
21	24.1%			0.0%	39.5%
27	31.0%	100.0%			
Number of drug c	% of all companies at a given threshold				
5	6.3%			0.0%	
9	11.3%			0.0%	
21	26.3%			0.0%	39.5%
20	25.0%	100.0%			
Number of drug c	% of all companies at a given threshold				
3	6.4%				
5	10.6%				
15	31.9%				39.5%
2	4.3%				
Number of drug c	% of all companies at a given threshold				
0	0.0%				
0	0.0%				
1	10.0%				
0	0.0%				
Total					
£54,071,454.2		33,907.5	3,584,604.6	49,000.0	364,970.8
£33,037,955.8		-	1,412,340.9	49,240.0	217,382.1
4235		3	281	5	38
772		0	40	2	16
62		1	1	1	1
62		0	1	1	1
Number of drug c	% of all drug companies with at least one payment with the value of at lea				
74	85.1%	1	1	1	1
45	51.7%		1		1
17	19.5%		1		
Number of drug c	% of all drug companies at a given threshold				

0	0.0%				
48	64.9%	1	1		1
26	35.1%			1	
Vallue of payments					
6,406,351.1		33908	2172264		147589
2,960,716.0				240	
Number of drug c	% of all drug companies at a given threshold				
0	0.0%				
32	71.1%		1		1
13	28.9%				
Vallue of payments					
6,406,351.1			2172264		147589
2,960,716.0					
Number of drug c	% of all drug companies at a given threshold				
0	0.0%				
13	76.5%		1		
4	23.5%				
Vallue of payments			0		
6,406,351.1			2172264		
2,960,716.0					
Number of drug c	% of all drug companies with at least one payment with value >£0 in at lea				
3	4.1%			0.5%	
9	12.2%			0.5%	
14	18.9%			0.5%	40.4%
24	32.4%	100.0%			
Number of drug c	% of all drug companies at a given threshold				
3	4.1%			0.5%	
9	12.2%			0.5%	
14	18.9%			0.5%	40.4%
24	32.4%	100.0%			
Number of drug c	% of all drug companies at a given threshold				
1	2.2%				
5	11.1%				
9	20.0%				40.4%
4	8.9%				
Number of drug c	% of all drug companies at a given threshold				
0	0.0%				
2	11.8%				

	2	11.8%
	1	5.9%

Aegerion	Alcon	Alexion	ALK-Abello	Allergan	Alliance	Almirall
0	0	1	1	1	1	1
5	1	5	0	25	0	9
4	9	17	2	26	1	2
1	1	1	0	1	0	1
1	1	1	1	1	1	1
1	1	1	1	1	1	1
1	1	1	1	1		1
		1		1		
1						1
	1	1	1	1	1	
1						7
	8	12	2	1	1	
1						1
	1	1	1	1		
1						7
	8	12	2	1		
		1		1		
		12		1		

				3.8%		
				3.8%		
20.0%				3.8%		
			100.0%		100.0%	
				3.8%		
				3.8%		
20.0%				3.8%		
			100.0%			
				3.8%		
				3.8%		
				3.8%		

35,040.1	15,150.0	60,600.0	-	678,030.8	-	62,968.9
-	83,733.8	70,583.4	-	148,240.9	-	-
5	1	5	0	25	0	9
0	5	8	0	4	0	0
1	1	1	0	1	0	1
0	1	1	0	1	0	0

st >£0 in at least one dataset

1	1	1		1		1
				1		

1			1	1
	1	1		
35040			529790	62969
	68584	9983		
			1	
			529790	

ıst one dataset

		14.1%		
		14.1%		
100.0%				100.0%
		14.1%		
		14.1%		
100.0%				100.0%

Amdipharm	Amgen	ApoPharma	Astellas	AstraZeneca	ausch and Lon
0	1	0	0	1	1
0	82	0	74	55	0
4	41	5	8	24	4
0	1	0	1	1	0
1	1	1	1	1	1
1	1	1	1	1	1
1	1	1	1	1	1
	1		1	1	
	1		1	1	
1		1			1
	41		66	31	
4		5			4
	1		1	1	
1		1			1
	41		66	31	
4		5			4
	1		1	1	
	41		66	31	

100.0%		100.0%			100.0%
100.0%		100.0%			100.0%
-	699,274.6	-	2,783,709.6	725,306.3	-
34,433.3	324,720.9	76,194.8	80,417.4	42,450.6	22,627.3
0	82	0	74	53	0
3	22	5	4	6	2
0	1	0	1	1	0
1	1	1	1	1	1
1	1	1	1	1	1
	1		1	1	
			1		

	1		1	1	
1		1			1
	374554		2703292	682856	
34433		76195			22627
	1		1	1	
	374554		2703292	682856	
			1		
			0		
			2703292		
100.0%		100.0%			100.0%
100.0%		100.0%			100.0%

Baxalta	Baxter	Bayer	Products Labor	Biogen	BioMarin
0	0	1	0	1	1
4	4	49	7	50	42
3	15	30	6	24	16
1	1	1	1	1	1
1	1	1	1	1	1
1	1	1	1	1	1
1	1	1	1	1	1
	1	1		1	1
1		1	1	1	1
	1				
1		19	1	26	26
	11				
1		1	1	1	1
	1				
1		19	1	26	26
	11				
		1		1	1
	1				
		19		26	26
	11				

			14.3%		
25.0%		38.8%	14.3%		
			14.3%		
25.0%		38.8%	14.3%		
		38.8%			
39,289.0	22,142.2	675,838.0	37,492.0	566,033.3	575,345.3
129,431.5	411,356.2	583,949.1	32,172.7	676,372.1	11,685.3
4	4	49	7	50	42
1	10	18	4	15	3
1	1	1	1	1	1
1	1	1	1	1	1
1	1	1	1	1	1
1	1	1		1	1

		1	1		1
1	1			1	
		91889	5319		563660
90143	389214			110339	
		1			1
1	1			1	
		91889			563660
90143	389214			110339	
		13.6%	14.2%	16.3%	
		13.6%	14.2%	16.3%	
		13.6%	14.2%	16.3%	
		13.6%	14.2%	16.3%	
		13.6%		16.3%	
		13.6%		16.3%	

Biotest	ehringer Ingelh	istol-Myers Squil	Britannia	Celgene	Chiesi
0	1	1	0	1	1
15	15	231	0	125	19
8	15	27	4	27	9
1	1	1	0	1	1
1	1	1	1	1	1
1	1	1	1	1	1
1	1	1	1	1	1
1	1	1		1	1
		1		1	
	1				
1		1		1	1
			1		
7		204		98	10
			4		
	1				
1		1		1	1
			1		
7		204		98	10
			4		
	1				
1		1		1	1
7		204		98	10
		1		1	

		204		98	

	0.0%				
	0.0%				
46.7%	0.0%				
			100.0%		
	0.0%				
	0.0%				
46.7%	0.0%				
			100.0%		
	0.0%				
	0.0%				
46.7%	0.0%				

88,420.4	133,153.7	1,759,705.1	-	1,828,014.0	188,262.1
131,337.0	134,736.3	281,373.4	3,884.0	493,270.0	57,591.4
15	15	230	0	125	19
7	7	17	1	11	2
1	1	1	0	1	1
1	1	1	1	1	1

1	1	1	1	1	1
1	1	1		1	1
		1		1	

		1		1	1
1	1		1		
		1478332		1334744	130671
42917	1583		3884		
		1		1	1
1	1				
		1478332		1334744	130671
42917	1583				
		1		1	
		0		0	
		1478332		1334744	
	1.2%				
	1.2%				
32.7%	1.2%				
			100.0%		
	1.2%				
	1.2%				
32.7%	1.2%				
			100.0%		
	1.2%				
	1.2%				
32.7%	1.2%				

Chugai	onsilient Healt	CSL Behring	Daiichi Sankyc	Dermal	Diurnal	Eisai
1	0	0	1	0	0	1
10	4	33	2	0	0	27
3	0	19	0	13	1	7
1	1	1	1	0	0	1
1	0	1	0	1	1	1
1	1	1	1	1	1	1
1	1	1	1	1		1
		1		1		1
1	1	1	1			1
				1	1	
7	4	14	2			20
				13	1	
1	1	1	1			1
				1		
7	4	14	2			20
				13		
		1				1
				1		
		14				20
				13		

		42.4%				
	100.0%		100.0%	100.0%	100.0%	
		42.4%				
	100.0%		100.0%	100.0%		
		42.4%				
				100.0%		
19,784.9	41,467.4	627,645.1	16,833.3	-	-	187,422.6
-	-	501,753.7	-	-	-	-
9	4	33	2	0	0	27
0	0	13	0	0	0	0
1	1	1	1	0	0	1
0	0	1	0	0	0	0
1	1	1	1			1
		1				1

1	1	1	1	1
19785	41467	125891	16833	187423
		1		1
		125891		187423
100.0%	100.0%	20.1%	100.0%	100.0%
100.0%	100.0%	20.1%	100.0%	100.0%
		20.1%		100.0%

Ferring	Flynn Pharma	enius Medical	Gedeon Richte	Genzyme	Gilead
0	0	1	0	0	0
23	19	0	0	0	174
29	4	3	2	52	90
1	1	0	0	0	1
1	1	1	1	1	1
1	1	1	1	1	1
1	1	1	1	1	1
1	1			1	1
					1
	1				1
1		1	1	1	
	15				84
6		3	2	52	
	1				1
1		1	1	1	
	15				84
6		3	2	52	
	1				1
1				1	
	15				84
6				52	
					1

					84
20.7%					48.3%
		100.0%	100.0%	100.0%	
20.7%					48.3%
		100.0%	100.0%	100.0%	
20.7%					48.3%
				100.0%	
					48.3%
180,131.8	38,577.9	-	-	-	1,616,738.8
44,886.0	5,175.2	15,940.6	-	1,225,894.6	1,306,716.1
23	19	0	0	0	174
3	3	3	0	25	69
1	1	0	0	0	1
1	1	1	0	1	1
1	1	1		1	1
1				1	1
				1	1

1	1			1
		1	1	
135246	33403			310023
		15941	1225895	
1				1
			1	
135246				310023
			1225895	
				1
			1	
			0	0
				310023
			1225895	
				19.2%
				19.2%
		100.0%	100.0%	
				19.2%
				19.2%
		100.0%	100.0%	
				19.2%
				19.2%
			100.0%	
				19.2%

19.2%

100.0%

Drug company names					
**Grünenthal**	**GSK**	**HRA Pharma**	**Ipsen**	**IQVIA**	**Janssen**
1	1	0	1	1	1
17	20	6	5	0	88
3	73	3	11	1	63
1	1	1	1	0	1
1	1	1	1	1	1
1	1	1	1	1	1
1	1	1	1		1
1	1		1		1
1		1			1
	1		1	1	
14		3			25
	53		6	1	
1		1			1
	1		1		
14		3			25
	53		6		
1					1
	1		1		
14					25
	53		6		

28.4%
100.0%

28.4%

28.4%

63,036.7	501,099.7	92,429.9	37,342.0	-	1,017,042.6
8,728.1	3,461,815.7	-	148,100.2	-	376,373.5
17	20	6	5	0	88
2	36	0	7	0	25
1	1	1	1	0	1
1	1	0	1	0	1
1	1	1	1		1
	1		1		1
	1				1

1 1 1

1 1

54309 92430 640669

2960716 110758

1

1 1

640669

2960716 110758

1

1

0 0

640669

2960716

100.0%

100.0%

Jazz	LEO Pharma	Lilly	Lundbeck	Meda	Merck
0	1	1	1	0	1
0	35	239	33	12	50
1	20	32	3	4	46
0	1	1	1	1	1
1	1	1	1	1	1
1	1	1	1	1	1
	1	1	1	1	1
	1	1	1	1	1
		1			
	1	1	1	1	1
1					
	15	207	30	8	4
1					
	1	1	1	1	1
	15	207	30	8	4
	1	1	1	1	1
	15	207	30	8	4
		1			

		207			
					8.0%
					8.0%
	42.9%				8.0%
100.0%					
					8.0%
					8.0%
	42.9%				8.0%
					8.0%
					8.0%
	42.9%				8.0%
-	151,378.2	2,825,173.2	223,802.5	77,182.6	455,388.5
-	10,000.0	143,038.3	-	75,750.0	905,077.7
0	34	237	33	12	50
0	1	12	0	1	19
0	1	1	1	1	1
0	1	1	0	1	1
	1	1	1	1	1
	1	1	1		1
		1			

1	1	1	1	
				1
141378	2682135	223803	1433	
				449689
1	1	1		
				1
141378	2682135	223803		
				449689
	1			
	0			
	2682135			
			1.9%	
			1.9%	
			1.9%	49.7%
		100.0%		
			1.9%	
			1.9%	
			1.9%	49.7%
		100.0%		
				49.7%
		100.0%		

ɔishi Tanabe P	MSD	Mylan	ɔp Pharmaceuti	Norgine	Novartis
1	1	0	1	1	1
0	235	0	60	10	435
1	55	1	24	10	154
0	1	0	1	1	1
1	1	1	1	1	1
1	1	1	1	1	1
	1		1	1	1
	1		1		1
	1				1
				1	
	1		1		1
1		1			
	180		36		281
1		1			
				1	
	1		1		1
	180		36		281
	1		1		1
	180		36		281
	1				1

	180				281
				0.0%	
				0.0%	
				0.0%	
100.0%		100.0%			
				0.0%	
				0.0%	
				0.0%	
-	1,750,167.8	-	173,152.9	102,297.0	5,195,484.0
5,070.3	837,180.0	-	82,633.4	33,700.5	2,102,441.4
0	233	0	60	10	433
1	29	0	10	2	104
0	1	0	1	1	1
1	1	0	1	1	1
1	1		1	1	1
	1		1	1	1
	1				1

1	1	1	1	1
5070	912988	90519	68596	3093043
	1	1	1	1
	912988	90519	68596	3093043
	1			1
	0			0
	912988			3093043
100.0%				
100.0%				

Novo Nordisk	Octapharma	Orion Pharma	Orphan Europe	ka Pharmaceut	Pfizer
1	0	1	0	1	1
47	2	8	0	13	886
17	7	4	3	11	129
1	1	1	0	1	1
1	1	1	1	1	1
1	1	1	1	1	1
1	1	1	1	1	1
1				1	1
					1
1		1		1	1
	1		1		
30		4		2	757
	5		3		
1		1		1	1
	1		1		
30		4		2	757
	5		3		
1				1	1
30				2	757
					1

					757
				15.4%	
				15.4%	
			100.0%		
				15.4%	
				15.4%	
			100.0%		
				15.4%	
				15.4%	
753,861.7	5,276.0	49,682.0	-	184,272.2	9,304,016.7
49,315.6	435,156.4	-	1,028.5	88,840.6	10,713,090.3
47	2	8	0	13	831
3	7	0	1	6	57
1	1	1	0	1	1
1	1	0	1	1	1
1	1	1	1	1	1
1	1			1	1
					1

1		1		1	
	1		1		1
704546		49682		95432	
	429880		1029		1409074
1				1	
	1				1
704546				95432	
	429880				1409074
					1
					0
					1409074
					13.2%
					13.2%
		100.0%	100.0%		
					13.2%
					13.2%
		100.0%	100.0%		
					13.2%
					13.2%
					13.2%

13.2%

Pharma Mar	PTC	RB	Roche Products	ont Pharmace	Sanofi
1	0	0	1	0	1
0	0	0	99	7	224
2	6	4	70	1	37
0	0	0	1	1	1
1	1	1	1	1	1
1	1	1	1	1	1
1	1	1	1	1	1
			1		1
					1
			1	1	1
1	1	1			
			29	6	187
2	6	4			
			1	1	1
1	1	1			
			29	6	187
2	6	4			
			1		1
			29		187
					1

					187
			29.3%		
100.0%	100.0%	100.0%			
			29.3%		
100.0%	100.0%	100.0%			
			29.3%		
-	-	-	1,369,625.3	-	2,562,336.3
6,312.5	-	144,270.0	660,704.8	-	189,271.9
0	0	0	99	0	219
1	0	4	27	0	10
0	0	0	1	0	1
1	0	1	1	0	1
1		1	1		1
		1	1		1
			1		1

		1	1
1	1		
		708920	2373064
6313	144270		
		1	1
	1		
		708920	2373064
	144270		
		1	1
		0	0
		708920	2373064
100.0%	100.0%		
100.0%	100.0%		
	100.0%		

nofi Pasteur M	Santen	rvier Laborato	Shire	n Tau Rare Dis	Sobi
0	1	1	1	0	1
0	1	20	115	0	36
2	2	3	37	8	13
0	1	1	1	0	1
1	1	1	1	1	1
1	1	1	1	1	1
1	1	1	1	1	1
		1	1		1
			1		
		1	1		1
1	1			1	
		17	78		23
2	1			8	
		1	1		1
1	1			1	
		17	78		23
2	1			8	
		1	1		1
		17	78		23
			1		

			78		
100.0%				100.0%	
100.0%				100.0%	
-	4,000.0	76,304.9	1,266,644.8	-	224,819.9
20,989.1	-	10,857.5	417,018.3	53,676.1	20,701.1
0	1	20	115	0	36
1	0	2	18	4	6
0	1	1	1	0	1
1	0	1	1	1	1
1	1	1	1	1	1
			1		1
			1		

1	1	1	1	1	1
20989	4000	65447	849626	53676	204119
			1		1
			849626		204119
			1		
			0<br>849626		
100.0%	100.0%			100.0%	
100.0%	100.0%			100.0%	

Stirling Anglia	Syner-Med	Takeda	Teva	Thea	Tillotts
0	0	1	0	0	0
3	0	54	24	0	7
1	3	42	28	1	6
1	0	1	1	0	1
1	1	1	1	1	1
1	1	1	1	1	1
1	1	1	1		1
		1	1		
1		1			1
	1		1	1	
2		12			1
	3		4	1	
1		1			1
	1		1		
2		12			1
	3		4		
		1			
			1		
		12			
			4		

			14.3%		14.3%
		22.2%	14.3%		14.3%
	100.0%			100.0%	
			14.3%		14.3%
		22.2%	14.3%		14.3%
	100.0%				
			14.3%		
		22.2%	14.3%		
11,110.0	-	6,614,214.3	642,199.4	-	48,612.7
-	-	207,863.1	274,284.3	-	32,002.9
3	0	54	23	0	7
0	0	16	13	0	2
1	0	1	1	0	1
0	0	1	1	0	1
1		1	1		1
		1	1		
		1			

1	1	1	1
11110	6406351	367915	16610
	1	1	
	6406351	367915	
	1		
	0		
	6406351		
			34.2%
100.0%			
			34.2%
100.0%			

UCB	Veriton	ifor Pharma Group
1	0	0
86	0	3
37	4	12
1	0	1
1	1	1
1	1	1
1	1	1
1		1
1		
	1	1
49		
	4	9
1		
	1	1
49		
	4	9
1		
		1
49		
		9

100.0%

100.0%

534,290.2	-	20,350.0
84,069.3	-	2,828,703.7
84	0	3
10	0	6
1	0	1
1	0	1
1		1
1		1
		1

1

1

450221

2808354

1

1

450221

2808354

1

0

2808354

Web Supplement 4. Donors - Absolute and relative differences in the number and value of payments (indiv

NUMBER OF PAYMENTS - ABSOLUTE DIFFERENCES

Threshold 0: At least 1 one payment in at least one dataset
Total
Number of drug companies with exact match in both datasets
Number of drug companies with more paymens t in industry data
Number of drug companies with more payments in patient organisation data

Highest absolute difference between patient organisation and industry data - number of payments higher in i
Highest absolute difference between patient organisation and industry data - number of payments higher in p

Threshold 1: More than 1 one payment in at least one datase
Total
Number of drug companies with exact match in both datasets
Number of drug companies with more paymens t in industry data
Number of drug companies with more payments in patient organisation data

Highest absolute difference between patient organisation and industry data - number of payments higher in i
Highest absolute difference between patient organisation and industry data - number of payments higher in p

Threshold 2: More than 10 payments in at least one dataset
Total
Number of drug companies with exact match in both datasets
Number of drug companies with more paymens t in industry data
Number of drug companies with more payments in patient organisation data

Highest absolute difference between patient organisation and industry data - number of payments higher in i
Highest absolute difference between patient organisation and industry data - number of payments higher in p

Threshold 3: More than 100 payments in at least one dataset
Total
Number of drug companies with exact match in both datasets
Number of drug companies with more paymens t in industry data
Number of drug companies with more payments in patient organisation data

Highest absolute difference between patient organisation and industry data - number of payments higher in i
Highest absolute difference between patient organisation and industry data - number of payments higher in p

NUMBER OF PAYMENTS - RELATIVE DIFFERENCES

Threshold 0: At least 1 one payment in at least one dataset
Total
Relative difference <10%

Relative difference <20%
Relative difference <50%
Relative difference = 100%

Threshold 1: More than 1 one payment in at least one datase
Total
Relative difference <10%
Relative difference <20%
Relative difference <50%
Relative difference = 100%

Threshold 2: More than 10 payments in at least one dataset
Total
Relative difference <10%
Relative difference <20%
Relative difference <50%
Relative difference = 100%

Threshold 3: More than 100 payments in at least one dataset
Total
Relative difference <10%
Relative difference <20%
Relative difference <50%
Relative difference = 100%

VALUE OF PAYMENTS - ABSOLUTE DIFFERENCES

Threshold 1: At least one payment with value >£0 in at least one dataset
Total
Number of drug companies with exact match in both datasets
Number of drug companies with higher payment value in industry data
Number of drug companies with higher payment value in patient organisation data

Highest absolute difference between patient organisation and industry data - number of payments higher in i
Highest absolute difference between patient organisation and industry data - number of payments higher in p

Threshold 2: Payments with the value of more than £10,000 in at least one dataset
Total
Number of drug companies with exact match in both datasets
Number of drug companies with higher payment value in industry data
Number of drug companies with higher payment value in patient organisation data

Highest absolute difference between patient organisation and industry data - number of payments higher in i
Highest absolute difference between patient organisation and industry data - number of payments higher in p

Threshold 3: Payments with the value of more than £100,000 in at least one dataset

Total
Number of drug companies with exact match in both datasets
Number of drug companies with higher payment value in industry data
Number of drug companies with higher payment value in patient organisation data

Highest absolute difference between patient organisation and industry data - number of payments higher in i
Highest absolute difference between patient organisation and industry data - number of payments higher in p

VALUE OF PAYMENTS- RELATIVE DIFFERENCES

Threshold 1: At least one payment with value >£0 in at least one dataset
Total
Relative difference <10%
Relative difference <20%
Relative difference <50%
Relative difference = 100%

Threshold 2: Payments with the value of at least £10,000 in at least one dataset
Total
Relative difference <10%
Relative difference <20%
Relative difference <50%
Relative difference = 100%

Threshold 3: Payments with the value of at least 100,000 in at least one dataset
Total
Relative difference <10%
Relative difference <20%
Relative difference <50%
Relative difference = 100%

All years			2012			2013		
Number of drug c	% of all drug	0	Number of drug	% of all drug	0	Number of dr	% of all drug	0
87	100.0%		61	100.0%		68	100.0%	
3	3.4%		1	1.6%		7	10.3%	
49	56.3%		28	45.9%		28	41.2%	
35	40.2%		32	52.5%		33	48.5%	
Number of payments			Number of payments			Number of payments		
757			216			135		
53			18			16		
Number of drug c	% of all drug	0	Number of drug	% of all drug	0	Number of dr	% of all drug	0
80	100.0%		46	100.0%		55	100.0%	
3	3.8%		1	2.2%		3	5.5%	
49	61.3%		28	60.9%		27	49.1%	
28	35.0%		17	37.0%		25	45.5%	
Number of payments			Number of payments			Number of payments		
757			216			135		
53			18			16		
Number of drug c	% of all drug	0	Number of drug	% of all drug	0	Number of dr	% of all drug	0
47	100.0%		16	100.0%		17	100.0%	
1	2.1%		0	0.0%		0	0.0%	
36	76.6%		13	81.3%		14	82.4%	
10	21.3%		3	18.8%		3	17.6%	
Number of payments			Number of payments			Number of payments		
757			216			135		
53			18			16		
Number of drug c	% of all drug	0	Number of drug	% of all drug	0	Number of dr	% of all drug	0
10	100.0%		1	100.0%		1	100.0%	
0	0.0%		0	0.0%		0	0.0%	
10	100.0%		1	100.0%		1	100.0%	
0	0.0%		0	0.0%		0	0.0%	
Number of payments			Number of payments			Number of payments		
757			216			135		
0			0			0		
Number of drug c	% of all drug co		Number of drug	% of all drug co		Number of dr	% of all drug co	
87	100.0%		61	100.0%		68	100.0%	
5	5.7%		1	1.6%		7	10.3%	

9	10.3%		2	3.3%		7	10.3%	
21	24.1%		7	11.5%		10	14.7%	
27	31.0%		36	59.0%		33	48.5%	
Number of drug c	% of all drug	0	Number of drug	% of all drug	0	Number of dr	% of all drug	0
80	100.0%		46	100.0%		55	100.0%	
5	6.3%		1	2.2%		3	5.5%	
9	11.3%		2	4.3%		3	5.5%	
21	26.3%		7	15.2%		6	10.9%	
20	25.0%		21	45.7%		24	43.6%	
Number of drug c	% of all drug	0	Number of drug	% of all drug	0	Number of dr	% of all drug	0
47	100.0%		16	100.0%		17	100.0%	
3	6.4%		0	0.0%		0	0.0%	
5	10.6%		0	0.0%		0	0.0%	
15	31.9%		1	6.3%		0	0.0%	
2	4.3%		3	18.8%		3	17.6%	
Number of drug c	% of all drug	0	Number of drug	% of all drug	0	Number of dr	% of all drug	0
10	100.0%		1	100.0%		1	100.0%	
0	0.0%		0	0.0%		0	0.0%	
0	0.0%		0	0.0%		0	0.0%	
1	10.0%		0	0.0%		0	0.0%	
0	0.0%		0	0.0%		0	0.0%	
Number of drug c	% of all drug	0	Number of drug	% of all drug	0	Number of dr	% of all drug	0
74	100.0%		52	100.0%		56	100.0%	
0	0.0%		0	0.0%		0	0.0%	
48	64.9%		26	50.0%		35	62.5%	
26	35.1%		26	50.0%		21	37.5%	
Value of payments			Value of payments			Value of payments		
6,406,351.2			1,576,293.48			666,171.11		
2,960,716.0			501,114.66			851,009.34		
Number of drug c	% of all drug	0	Number of drug	% of all drug	0	Number of dr	% of all drug	0
45	100.0%		20	100.0%		24	100.0%	
0	0.0%		0	0.0%		0	0.0%	
32	71.1%		13	65.0%		14	58.3%	
13	28.9%		7	35.0%		10	41.7%	
Value of payments			Value of payments			Value of payments		
6,406,351.2			1,576,293.5			666,171.1		
2,960,716.0			501,114.7			851,009.3		
Number of drug c	% of all drug	0	Number of drug	% of all drug	0	Number of dr	% of all drug	0

17	100.0%	2	100.0%	1	100.0%
0	0.0%	0	0.0%	0	0.0%
13	76.5%	2	100.0%	1	100.0%
4	23.5%	0	0.0%	0	0.0%

Value of payments	Value of payments	Value of payments
6,406,351.2	1,576,293.5	604,556.0
2,960,716.0	-	-

Number of drug c	% of all drug	0	Number of drug	% of all drug	0	Number of dr	% of all drug	0
74	100.0%		52	100.0%		56	100.0%	
3	4.1%		1	1.9%		0	0.0%	
9	12.2%		1	1.9%		4	7.1%	
14	18.9%		4	7.7%		7	12.5%	
24	32.4%		32	61.5%		30	53.6%	

Number of drug c	% of all drug	0	Number of drug	% of all drug	0	Number of dr	% of all drug	0
45	100.0%		20	100.0%		24	100.0%	
1	2.2%		0	0.0%		0	0.0%	
5	11.1%		0	0.0%		1	4.2%	
9	20.0%		1	5.0%		3	12.5%	
4	8.9%		8	40.0%		9	37.5%	

Number of drug c	% of all drug	0	Number of drug	% of all drug	0	Number of dr	% of all drug	0
17	100.0%		2	100.0%		1	100.0%	
0	0.0%		0	0.0%		0	0.0%	
2	11.8%		0	0.0%		0	0.0%	
2	11.8%		0	0.0%		1	100.0%	
1	5.9%		0	0.0%		0	0.0%	

2014			2015			2016	
Number of drug	% of all drug	0	Number of drug	% of all drug	0	Number of drug	% of all drug
72	100.0%		74	100.0%		69	100.0%
4	5.6%		7	9.5%		4	5.8%
38	52.8%		42	56.8%		39	56.5%
30	41.7%		25	33.8%		26	37.7%
Number of payments			Number of payments			Number of payments	
108			125			173	
13			13			12	
Number of drug	% of all drug	0	Number of drug	% of all drug	0	Number of drug	% of all drug
55	100.0%		57	100.0%		50	100.0%
1	1.8%		5	8.8%		3	6.0%
36	65.5%		42	73.7%		38	76.0%
18	32.7%		10	17.5%		9	18.0%
Number of payments			Number of payments			Number of payments	
108			125			173	
13			13			12	
Number of drug	% of all drug	0	Number of drug	% of all drug	0	Number of drug	% of all drug
21	100.0%		23	100.0%		23	100.0%
0	0.0%		0	0.0%		0	0.0%
18	85.7%		22	95.7%		22	95.7%
3	14.3%		1	4.3%		1	4.3%
Number of payments			Number of payments			Number of payments	
108			125			173	
13			13			12	
Number of drug	% of all drug	0	Number of drug	% of all drug	0	Number of drug	% of all drug
2	100.0%		1	100.0%		100.0%	100.0%
0	0.0%		0	0.0%		0	0.0%
2	100.0%		1	100.0%		1	100.0%
0	0.0%		0	0.0%		0	0.0%
Number of payments			Number of payments			Number of payments	
108			125			173	
0			0			0	
Number of drug	% of all drug co		Number of drug	% of all drug co		Number of drug	% of all drug
72	100.0%		74	100.0%		69	100.0%
4	5.6%		7	9.5%		4	5.8%

5	6.9%		9	12.2%		5	7.2%
12	16.7%		17	23.0%		12	17.4%
34	47.2%		28	37.8%		29	42.0%

Number of drug	% of all drug	0	Number of drug	% of all drug	0	Number of drug	% of all drug
55	100.0%		57	100.0%		50	100.0%
1	1.8%		5	8.8%		3	6.0%
2	3.6%		7	12.3%		4	8.0%
9	16.4%		15	26.3%		11	22.0%
20	36.4%		13	22.8%		11	22.0%

Number of drug	% of all drug	0	Number of drug	% of all drug	0	Number of drug	% of all drug
21	100.0%		23	100.0%		23	100.0%
0	0.0%		0	0.0%		0	0.0%
1	4.8%		0	0.0%		0	0.0%
3	14.3%		4	17.4%		2	8.7%
2	9.5%		1	4.3%		1	4.3%

Number of drug	% of all drug	0	Number of drug	% of all drug	0	Number of drug	% of all drug
2	100.0%		1	100.0%		100.0%	100.0%
0	0.0%		0	0.0%		0	0.0%
0	0.0%		0	0.0%		0	0.0%
0	0.0%		0	0.0%		0	0.0%
0	0.0%		0	0.0%		0	0.0%

Number of drug	% of all drug	0	Number of drug	% of all drug	0	Number of drug	% of all drug
59	100.0%		60	100.0%		55	100.0%
0	0.0%		1	1.7%		0	0.0%
39	66.1%		41	68.3%		38	69.1%
20	33.9%		18	30.0%		17	30.9%

Value of payments	Value of payments	Value of payments
902,858.00	1,637,974.69	5,986,743.37
2,123,196.45	729,868.42	5,541,099.82

Number of drug	% of all drug	0	Number of drug	% of all drug	0	Number of drug	% of all drug
28	100.0%		30	100.0%		28	100.0%
0	0.0%		0	0.0%		0	0.0%
21	75.0%		25	83.3%		23	82.1%
7	25.0%		5	16.7%		5	17.9%

Value of payments	Value of payments	Value of payments
902,858.0	1,637,974.7	5,986,743.4
2,123,196.4	729,868.4	5,541,099.8

Number of drug	% of all drug	0	Number of drug	% of all drug	0	Number of drug	% of all drug

3	100.0%	2	100.0%	2	100.0%
0	0.0%	0	0.0%	0	0.0%
2	66.7%	2	100.0%	1	50.0%
1	33.3%	0	0.0%	1	50.0%

Value of payments	Value of payments	Value of payments
902,858.0	1,637,974.7	5,986,743.4
2,123,196.4	-	5,541,099.8

Number of drug	% of all drug	0	Number of drug	% of all drug	0	Number of drug	% of all drug
59	100.0%		60	100.0%		55	100.0%
1	1.7%		5	8.3%		0	0.0%
1	1.7%		8	13.3%		4	7.3%
8	13.6%		17	28.3%		14	25.5%
31	52.5%		24	40.0%		22	40.0%

Number of drug	% of all drug	0	Number of drug	% of all drug	0	Number of drug	% of all drug
28	100.0%		30	100.0%		28	100.0%
1	3.6%		2	6.7%		0	0.0%
1	3.6%		2	6.7%		3	10.7%
5	17.9%		8	26.7%		10	35.7%
8	28.6%		6	20.0%		4	14.3%

Number of drug	% of all drug	0	Number of drug	% of all drug	0	Number of drug	% of all drug
3	100.0%		2	100.0%		2	100.0%
0	0.0%		0	0.0%		0	0.0%
0	0.0%		0	0.0%		0	0.0%
0	0.0%		0	0.0%		0	0.0%
1	33.3%		0	0.0%		0	0.0%

companies at a given threshold

companies at a given threshold

companies at a given threshold

companies at a given threshold

companies

companies at a given threshold

companies at a given threshold

companies at a given threshold

companies at a given threshold

companies at a given threshold

companies at a given threshold

companies at a given threshold

companies at a given threshold

companies at a given threshold

Web Supplement 5. Recipients - Absolute and relative differences in the number and value of payments (al

NUMBER OF PAYMENTS

Number of payments - industry data
Number of payments - patient organisation data
Number of patient organisations with at least 1 payment - industry data
Number of patient organisations with at least 1 payment - patient organisation data

NUMBER OF PAYMENTS - THRESHOLDS

Threshold 0: Number of patient organisations with at least 1 one payment in at least one dataset
Threshold 1: Number of patient organisations with more than 1 one payment in at least one dataset
Threshold 2: Number of patient organisations with more than 10 payments in at least one dataset
Threshold 3: Number of patient organisations with more than 100 payments in at least one dataset

NUMBER OF PAYMENTS - ABSOLUTE DIFFERENCES

Number of payments - threshold 0
Number of patient organisations with exact match in both datasets
Number of patient organisations with more paymens in industry data
Number of patient organisations with more payments in patient organisation data

Highest absolute difference between patient organisation and industry data - number of payments higher in i
Highest absolute difference between patient organisation and industry data - number of payments higher in p

Number of payments - threshold 1
Number of patient organisations with exact match in both datasets
Number of patient organisations with more paymens in industry data
Number of patient organisations with more payments in patient organisation data

Highest absolute difference between patient organisation and industry data - number of payments higher in i
Highest absolute difference between patient organisation and industry data - number of payments higher in p

Number of payments - threshold 2
Number of patient organisations with exact match in both datasets
Number of patient organisations with more paymens in industry data
Number of patient organisations with more payments in patient organisation data

Highest absolute difference between patient organisation and industry data - number of payments higher in i
Highest absolute difference between patient organisation and industry data - number of payments higher in p

Number of payments - threshold 3
Number of patient organisations with exact match in both datasets
Number of patient organisations with more paymens in industry data

Number of patient organisations with more payments in patient organisation data

Highest absolute difference between patient organisation and industry data - number of payments higher in i
Highest absolute difference between patient organisation and industry data - number of payments higher in p

NUMBER OF PAYMENTS - RELATIVE DIFFERENCES

Relative difference <10%
Relative difference <20%
Relative difference <50%
Relative difference = 100%

Number of payments - threshold 1
Relative difference <10%
Relative difference <20%
Relative difference <50%
Relative difference = 100%

Number of payments - threshold 2
Relative difference <10%
Relative difference <20%
Relative difference <50%
Relative difference = 100%

Number of payments - threshold 3
Relative difference <10%
Relative difference <20%
Relative difference <50%
Relative difference = 100%

VALUE OF PAYMENTS - ABSOLUTE DIFFERENCES

Value of payments - industry data (2016 £)
Value of payments - patient organisation data (2016 £)
Number of payments with value >£0 - industry data
Number of payments with value >£0 - patient organisation data
Number of patient organisations with at least one payemnt with the value >£0 - industry data
Number of patient organisations with at least one payemnt with the value >£0 - patient organisation data

VALUE OF PAYMENTS - THRESHOLDS

Threshold 1: Number of patient organisations with at least one payment with value >0 in at least one dataset
Threshold 2: Number of patient organisations with payments with the value of more than £10,000 in at least
Threshold 3: Number of patient organisations with payments with the value of more than £100,000 in at leas

VALUE OF PAYMENTS - ABSOLUTE DIFFERENCES

Number of payments - threshold 1

Number of patient organisations with exact match in both datasets
Number of patient organisations with higher payment value in industry data
Number of patient organisations with higher payment value in patient organisation data

Highest absolute difference between patient organisation and industry data - number of payments higher in i
Highest absolute difference between patient organisation and industry data - number of payments higher in p

Number of payments - threshold 2
Number of patient organisations with exact match in both datasets
Number of patient organisations with higher payment value in industry data
Number of patient organisations with higher payment value in patient organisation data

Highest absolute difference between patient organisation and industry data - number of payments higher in i
Highest absolute difference between patient organisation and industry data - number of payments higher in p

Number of payments - threshold 3
Number of patient organisations with exact match in both datasets
Number of patient organisations with higher payment value in industry data
Number of patient organisations with higher payment value in patient organisation data

Highest absolute difference between patient organisation and industry data - number of payments higher in i
Highest absolute difference between patient organisation and industry data - number of payments higher in p

VALUE OF PAYMENTS - RELATIVE DIFFERENCES

Relative difference <10%
Relative difference <20%
Relative difference <50%
Relative difference = 100%

Number of payments - threshold 1
Relative difference <10%
Relative difference <20%
Relative difference <50%
Relative difference = 100%

Number of payments - threshold 2
Relative difference <10%
Relative difference <20%
Relative difference <50%
Relative difference = 100%

Number of payments - threshold 3
Relative difference <10%
Relative difference <20%

Relative difference <50%
Relative difference = 100%

		21 and Co	AADC Resear	Action Bladd	Action Duche
Total					
4316		1	1	1	12
1661		0	0	0	0
425		1	1	1	1
200		0	0	0	0
Number of patien	% of all patient organisations with at least one payment in at least one				
425	100.0%	1	1	1	1
299	70.4%				1
115	27.1%				1
3	0.7%				
Number of patien	% of all patient organisations at a given threshold				
33	7.8%				
335	78.8%	1	1	1	1
57	13.4%				
Number of payments					
199		1	1	1	12
33					
Number of patien	% of all patient organisations at a given threshold				
13	4.3%				
229	76.6%				1
57	19.1%				
Number of payments					
199					12
33					
Number of patien	% of all patient organisations at a given threshold				
2	1.7%				
95	82.6%				1
18	15.7%				
Number of payments					
199					12
33					
Number of patien	% of all patient organisations at a given threshold				
0	0.0%				
3	100.0%				

0	0.0%				
Number of payments					
199					
0					
Number of patien	% of all patient organisations with at least one payment in at least one				
35	8.2%				
42	9.9%				
81	19.1%				
225	52.9%	100.0%	100.0%	100.0%	100.0%
Number of patien	% of all patient organisations at a given threshold				
15	5.0%				
22	7.4%				
61	20.4%				
119	39.8%				100.0%
Number of patien	% of all patient organisations at a given threshold				
4	3.5%				
7	6.1%				
31	27.0%				
33	28.7%				100.0%
Number of patien	% of all patient organisations at a given threshold				
0	0.0%				
0	0.0%				
0	0.0%				
1	33.3%				
£54,071,454.2		1029	5070	101	47076
£33,037,955.8		0	0	0	0
4235		1	1	1	12
772		0	0	0	0
416		1	1	1	1
121		0	0	0	0
416		1	1	1	1
104					
13					
Number of patien	% of all patient organisations at a given threshold				

8	1.9%				
356	85.6%	1	1	1	1
52	12.5%				
Vallue of payments		0	0	0	0
6,718,576.6		1029	5070	101	47076
6,493,237.1					
Number of patien	% of all patient organisations at a given threshold				
0	0.0%				
80	76.9%				
24	23.1%				
Vallue of payments					
6,718,576.6					
6,493,237.1					
Number of patien	% of all patient organisations at a given threshold				
0	0.0%				
6	46.2%				
7	53.8%				
Vallue of payments					
6,718,576.6					
6,493,237.1					
Number of patien	% of all patient organisations with at least one payment with value >£0				
21	5.0%				
34	8.2%				
73	17.5%				
295	70.9%	100.0%	100.0%	100.0%	100.0%
21	5.0%				
34	8.2%				
73	17.5%				
295	70.9%	100.0%	100.0%	100.0%	100.0%
4	3.8%				
8	7.7%				
23	22.1%				
54	51.9%				
0	0.0%				
0	0.0%				

	3	23.1%
	4	30.8%

	Action for Pu	Action on Pai	Action on Pr	Action on Sm	Action on Sm	Actionplus Fo	Add+Up
	3	12	1	1	5	2	3
	2	0	1	0	5	0	5
	1	1	1	1	1	1	1
	1	0	1	0	1	0	1
dataset							
	1	1	1	1	1	1	1
	1	1			1	1	1
		1					
			1		1		
	1	1		1		1	
							1
	1	12		1		2	
							2
					1		
	1	1				1	
							1
	1	12				2	
							2
		1					
		12					

dataset							
			0.0%		0.0%		
			0.0%		0.0%		
	33.3%		0.0%		0.0%		40.0%
		100.0%		100.0%		100.0%	
					0.0%		
					0.0%		
	33.3%				0.0%		40.0%
		100.0%				100.0%	
		100.0%					
	14053	13947	10521	2520	28933	7941	30629
	18300	0	10285	0	33478	0	37297
	3	10	1	1	5	2	3
	2	0	1	0	5	0	5
	1	1	1	1	1	1	1
	1	0	1	0	1	0	1
	1	1	1	1	1	1	1

	1	1	1		1	
1				1		1
0	0	0	0	0	0	0
	13947	236	2520		7941	
4247				4545		6667

in at least one dataset

		2.2%				
		2.2%		13.6%		17.9%
23.2%		2.2%		13.6%		17.9%
	100.0%		100.0%		100.0%	
		2.2%				
		2.2%		13.6%		17.9%
23.2%		2.2%		13.6%		17.9%
	100.0%		100.0%		100.0%	

Addaction	Addenbrooks	Addison's Dis	Adfam	ADHD Found	Africa Advoc	African Healt
20	1	3	1	9	8	12
2	0	1	2	1	7	6
1	1	1	1	1	1	1
1	0	1	1	1	1	1
1	1	1	1	1	1	1
1		1	1	1	1	1
1						1
1	1	1		1	1	1
			1			
18	1	2		8	1	6
			1			
1		1		1	1	1
			1			
18		2		8	1	6
			1			
1						1
18						6

					12.5%	
					12.5%	
	100.0%					
					12.5%	
					12.5%	
356861	3042	25000	270	27042	40023	57095
98542	0	25000	35800	20281	35149	23988
20	1	3	1	9	8	12
2	0	1	2	1	6	5
1	1	1	1	1	1	1
1	0	1	1	1	1	1
1	1	1	1	1	1	1
1						

		1				
1	1			1	1	1
			1			
0	0	0	0	0	0	0
258319	3042			6761	4874	33107
			35530			
1						
0						
258319						
		0.0%				
		0.0%			12.2%	
		0.0%		25.0%	12.2%	
	100.0%					
		0.0%				
		0.0%			12.2%	
		0.0%		25.0%	12.2%	
	100.0%					

African-Carib	African's Get	Age Related	Age UK	Age UK - Cym	AGE UK - Isle	aHUS UK
6	1	2	12	2	1	1
0	0	0	4	4	2	0
1	1	1	1	1	1	1
0	0	0	1	1	1	0
1	1	1	1	1	1	1
1		1	1	1	1	
			1			
1	1	1	1			1
				1	1	
6	1	2	8			1
				2	1	
1		1	1			
				1	1	
6		2	8			
				2	1	
			1			
			8			

100.0%	100.0%	100.0%				100.0%
100.0%		100.0%				
70152	130	39259	97127	11210	42574	25250
0	0	0	1825301	0	24278	0
6	1	2	12	2	1	1
0	0	0	1	0	1	0
1	1	1	1	1	1	1
0	0	0	1	0	1	0
1	1	1	1	1	1	1
			1			
			1			

1	1	1	1	1	1	1
0	0	0	0	0	0	0
70152	130	39259	1728174	11210	18296	25250
			1			
			0			
			1728174			
			1			
			0			
			1728174			
100.0%	100.0%	100.0%		100.0%	43.0%	100.0%
100.0%	100.0%	100.0%		100.0%	43.0%	100.0%

Airedale Volu	Alcohol Conc	ALD Life	Allergy Resea	Allergy UK	Alpha-1 Awa	Alpha-1 UK S
3	18	1	2	23	2	5
3	2	0	0	8	0	0
1	1	1	1	1	1	1
1	1	0	0	1	0	0
1	1	1	1	1	1	1
1	1		1	1	1	1
	1			1		
1						
	1	1	1	1	1	1
	16	1	2	15	2	5
1						
	1		1	1	1	1
	16		2	15	2	5
	1			1		
	16			15		

0.0%						
0.0%						
0.0%						
		100.0%	100.0%		100.0%	100.0%
0.0%						
0.0%						
0.0%						
			100.0%		100.0%	100.0%

34921	128034	262	21042	280169	16456	138585
34921	0	0	0	86271	0	0
3	18	1	2	23	2	5
3	0	0	0	2	0	0
1	1	1	1	1	1	1
1	0	0	0	1	0	0
1	1	1	1	1	1	1
	1			1		1

1						
	1	1	1	1	1	1
0	0	0	0	0	0	0
	128034	262	21042	193898	16456	138585
	1			1		1
	0			0		0
	128034			193898		138585
0.0%						
0.0%						
0.0%						
	100.0%	100.0%	100.0%		100.0%	100.0%
0.0%						
0.0%						
0.0%						
	100.0%	100.0%	100.0%		100.0%	100.0%
	100.0%					100.0%

BMJ Publishing Group Limited (BMJ) disclaims all liability and responsibility arising from any reliance placed on this supplemental material which has been supplied by the author(s)

Alzheimer Sc	Alzheimer's F	Alzheimer’s S	Alzheimers D	Anaphylaxis	Angelman Sy	Ankylosing S
2	7	18	1	8	1	6
0	10	5	0	3	0	0
1	1	1	1	1	1	1
0	1	1	0	1	0	0
1	1	1	1	1	1	1
1	1	1		1		1
		1				
1		1	1	1	1	1
	1					
2		13	1	5	1	6
	3					
1		1		1		1
	1					
2		13		5		6
	3					
		1				
		13				

	30.0%					
100.0%			100.0%		100.0%	100.0%
	30.0%					
100.0%						100.0%
564	443860	253057	1057	45474	303	38723
0	0	0	0	0	0	0
2	7	18	1	8	1	6
0	0	0	0	0	0	0
1	1	1	1	1	1	1
0	0	0	0	0	0	0
1	1	1	1	1	1	1
	1	1				

1	1	1	1	1	1	1
0	0	0	0	0	0	0
564	443860	253057	1057	45474	303	38723
	1	1				
	0	0				
	443860	253057				
100.0%	100.0%	100.0%	100.0%	100.0%	100.0%	100.0%
100.0%	100.0%	100.0%	100.0%	100.0%	100.0%	100.0%
	100.0%	100.0%				

Anthony Nol	Anticoagulati	Anxiety UK	Aplastic Ana	Arrhythmia A	Arthritis and	Arthritis Care
3	76	8	1	34	32	50
2	0	2	0	0	0	10
1	1	1	1	1	1	1
1	0	1	0	0	0	1
1	1	1	1	1	1	1
1	1	1		1	1	1
	1			1	1	1
1	1	1	1	1	1	1
1	76	6	1	34	32	40
1	1	1		1	1	1
1	76	6		34	32	40
	1			1	1	1
	76			34	32	40

33.3%						
	100.0%		100.0%	100.0%	100.0%	
33.3%						
	100.0%			100.0%	100.0%	
	100.0%			100.0%	100.0%	
30494	375207	21339	1616	329518	262194	391558
0	0	0	0	0	0	0
3	76	7	1	34	32	49
0	0	0	0	0	0	0
1	1	1	1	1	1	1
0	0	0	0	0	0	0
1	1	1	1	1	1	1
	1			1	1	1

1	1	1	1	1	1	1
0	0	0	0	0	0	0
30494	375207	21339	1616	329518	262194	391558
	1			1	1	1
	0			0	0	0
	375207			329518	262194	391558
100.0%	100.0%	100.0%	100.0%	100.0%	100.0%	100.0%
100.0%	100.0%	100.0%	100.0%	100.0%	100.0%	100.0%
	100.0%			100.0%	100.0%	100.0%

Arthritis Rese	Association f	Association f	Asthma and A	Asthma Relie	Asthma UK	Asyabi UK
6	13	6	1	1	34	1
3	1	5	0	0	34	0
1	1	1	1	1	1	1
1	1	1	0	0	1	0
1	1	1	1	1	1	1
1	1	1			1	
	1				1	
					1	
1	1	1	1	1		1
3	12	1	1	1		1
					1	
1	1	1				
3	12	1				
					1	
	1					
	12					

					0.0%	
		16.7%			0.0%	
		16.7%			0.0%	
			100.0%	100.0%		100.0%
					0.0%	
		16.7%			0.0%	
		16.7%			0.0%	
					0.0%	
					0.0%	
					0.0%	
248103	71616	35059	507	1014	301952	1500
0	0	20100	0	0	0	0
5	13	5	1	1	33	1
0	0	2	0	0	0	0
1	1	1	1	1	1	1
0	0	1	0	0	0	0
1	1	1	1	1	1	1
1					1	

1	1	1	1	1	1	1
0	0	0	0	0	0	0
248103	71616	14959	507	1014	301952	1500
1					1	
0					0	
248103					301952	
100.0%	100.0%	42.7%	100.0%	100.0%	100.0%	100.0%
100.0%	100.0%	42.7%	100.0%	100.0%	100.0%	100.0%
100.0%					100.0%	

Ataxia UK	Atrial Fibrilla	Back on Trac	BackCare	Barts and Th	Basil Skyers	Batten Disea
8	104	1	11	1	4	10
8	0	0	4	0	0	4
1	1	1	1	1	1	1
1	0	0	1	0	0	1
1	1	1	1	1	1	1
1	1		1		1	1
	1		1			
	1					
1						
	1	1	1	1	1	1
	104	1	7	1	4	6
1						
	1		1		1	1
	104		7		4	6
	1		1			
	104		7			
	1					

	104					
0.0%						
0.0%						
0.0%						
	100.0%	100.0%		100.0%	100.0%	
0.0%						
0.0%						
0.0%						
	100.0%				100.0%	
	100.0%					
	100.0%					
33556	621144	3809	43576	206	35201	73762
36402	0	0	9127	0	0	0
8	102	1	8	1	4	10
2	0	0	2	0	0	0
1	1	1	1	1	1	1
1	0	0	1	0	0	0
1	1	1	1	1	1	1
	1					

	1	1	1	1	1	1
1						
0	0	0	0	0	0	0
	621144	3809	34450	206	35201	73762
2846						
	1					
	0					
	621144					
7.8%						
7.8%						
7.8%						
	100.0%	100.0%		100.0%	100.0%	100.0%
7.8%						
7.8%						
7.8%						
	100.0%	100.0%		100.0%	100.0%	100.0%
	100.0%					

Beat Parkins	Beating Bow	Behcets Sync	Bipolar Scotl	Bipolar UK	Birdshot Uve	Bladder and B
1	42	1	3	3	1	38
0	0	0	0	0	0	0
1	1	1	1	1	1	1
0	0	0	0	0	0	0
1	1	1	1	1	1	1
	1		1	1		1
	1					1
1	1	1	1	1	1	1
1	42	1	3	3	1	38
	1		1	1		1
	42		3	3		38
	1					1
	42					38

100.0%	100.0%	100.0%	100.0%	100.0%	100.0%	100.0%
	100.0%		100.0%	100.0%		100.0%
	100.0%					100.0%
5050	342673	20281	3194	4145	10000	2271217
0	0	0	0	0	0	0
1	42	1	3	3	1	38
0	0	0	0	0	0	0
1	1	1	1	1	1	1
0	0	0	0	0	0	0
1	1	1	1	1	1	1
	1					1
						1

1	1	1	1	1	1	1
0	0	0	0	0	0	0
5050	342673	20281	3194	4145	10000	2271217
	1					1
	0					0
	342673					2271217
						1
						0
						2271217
100.0%	100.0%	100.0%	100.0%	100.0%	100.0%	100.0%
100.0%	100.0%	100.0%	100.0%	100.0%	100.0%	100.0%
	100.0%					100.0%

100.0%

Bladder Heal	Bliss	Blood Pressu	Bloodwise	Bluebell Fou	Body and So	Bone Cancer
17	13	1	19	2	6	3
13	6	0	1	0	0	0
1	1	1	1	1	1	1
1	1	0	1	0	0	0
1	1	1	1	1	1	1
1	1		1	1	1	1
1	1		1			
1	1	1	1	1	1	1
4	7	1	18	2	6	3
1	1		1	1	1	1
4	7		18	2	6	3
1	1		1			
4	7		18			

23.5%						
		100.0%		100.0%	100.0%	100.0%
23.5%						
				100.0%	100.0%	100.0%
23.5%						
76682	204923	10521	197710	776	97946	12161
0	0	0	0	0	0	0
16	13	1	19	2	6	3
0	0	0	0	0	0	0
1	1	1	1	1	1	1
0	0	0	0	0	0	0
1	1	1	1	1	1	1
	1		1			

1	1	1	1	1	1	1
0	0	0	0	0	0	0
76682	204923	10521	197710	776	97946	12161
	1		1			
	0		0			
	204923		197710			
100.0%	100.0%	100.0%	100.0%	100.0%	100.0%	100.0%
100.0%	100.0%	100.0%	100.0%	100.0%	100.0%	100.0%
	100.0%		100.0%			

Bowel Cance	Brain Charity	Brain Tumor	Brain Tumou	Breakthroug	Breast Cance	Breast Cance
16	1	1	1	16	7	28
13	0	0	0	3	0	19
1	1	1	1	1	1	1
1	0	0	0	1	0	1
1	1	1	1	1	1	1
1				1	1	1
1				1		1
1	1	1	1	1	1	1
3	1	1	1	13	7	9
1				1	1	1
3				13	7	9
1				1		1
3				13		9

18.8%						
18.8%						32.1%
	100.0%	100.0%	100.0%		100.0%	
18.8%						
18.8%						32.1%
					100.0%	
18.8%						
18.8%						32.1%
109272	981	12676	262	109107	81316	332296
0	0	0	0	0	0	369592
16	1	1	1	16	6	25
0	0	0	0	0	0	14
1	1	1	1	1	1	1
0	0	0	0	0	0	1
1	1	1	1	1	1	1
1				1		1

1	1	1	1	1	1	
						1
0	0	0	0	0	0	0
109272	981	12676	262	109107	81316	
						37296
1				1		
						1
0				0		0
109272				109107		
						37296
						10.1%
						10.1%
100.0%	100.0%	100.0%	100.0%	100.0%	100.0%	
						10.1%
						10.1%
100.0%	100.0%	100.0%	100.0%	100.0%	100.0%	
						10.1%
						10.1%
100.0%				100.0%		

Breast Cance	Breast Cancer I	British Dupuy	British Heart	British Liver T	British Lung F	British Obesi
5	4	8	7	29	50	1
0	1	0	0	5	23	0
1	1	1	1	1	1	1
0	1	0	0	1	1	0
1	1	1	1	1	1	1
1	1	1	1	1	1	
				1	1	
1	1	1	1	1	1	1
5	3	8	7	24	27	1
1	1	1	1	1	1	
5	3	8	7	24	27	
				1	1	
				24	27	

100.0%		100.0%	100.0%			100.0%
100.0%		100.0%	100.0%			
47894	3506763	55592	1882	229800	574095	421
0	10000000	0	0	0	1139917	0
5	2	8	7	29	47	1
0	1	0	0	0	22	0
1	1	1	1	1	1	1
0	1	0	0	0	1	0
1	1	1	1	1	1	1
	1			1	1	
	1				1	

1		1	1	1		1
	1				1	
0	0	0	0	0	0	0
47894		55592	1882	229800		421
	6493237				565821	
				1		
	1				1	
	0			0	0	
				229800		
	6493237				565821	
	1				1	
	0				0	
	6493237				565821	
					49.6%	
100.0%		100.0%	100.0%	100.0%		100.0%
					49.6%	
100.0%		100.0%	100.0%	100.0%		100.0%
					49.6%	
				100.0%		

49.6%

British Polio	British Pregn	British Skin F	British Thyro	Brittle Bone	Brook	Building for t
1	5	6	1	1	12	1
0	2	0	5	2	0	0
1	1	1	1	1	1	1
0	1	0	1	1	0	0
1	1	1	1	1	1	1
	1	1	1	1	1	
					1	
1	1	1			1	1
			1	1		
1	3	6			12	1
			4	1		
	1	1			1	
			1	1		
	3	6			12	
			4	1		
					1	
					12	

100.0%		100.0%			100.0%	100.0%
		100.0%			100.0%	
					100.0%	
0	127999	9785	250	15428	95962	243
0	302001	0	44574	20000	0	0
0	5	6	1	1	12	1
0	2	0	4	1	0	0
0	1	1	1	1	1	1
0	1	0	1	1	0	0
	1	1	1	1	1	1
	1					

FALSE						
		1			1	1
	1		1	1		
	0	0	0	0	0	0
		9785			95962	243
	174002		44324	4572		
	1					
	0					
	174002					
				22.9%		
		100.0%			100.0%	100.0%
				22.9%		
		100.0%			100.0%	100.0%

BMJ Publishing Group Limited (BMJ) disclaims all liability and responsibility arising from any reliance placed on this supplemental material which has been supplied by the author(s)

Butterfly Thy	Cambridge C	Cambridge R	Cancer 52	Cancer Black	Cancer Focus	Cancer Resear
3	1	4	26	2	13	57
9	2	0	0	4	0	0
1	1	1	1	1	1	1
1	1	0	0	1	0	0
1	1	1	1	1	1	1
1	1	1	1	1	1	1
			1		1	1
		1	1		1	1
1	1			1		
		4	26		13	57
6	1			2		
		1	1		1	1
1	1			1		
		4	26		13	57
6	1			2		
			1		1	1
			26		13	57

		100.0%	100.0%		100.0%	100.0%
		100.0%	100.0%		100.0%	100.0%
			100.0%		100.0%	100.0%
32775	507	9320	249099	9615	29164	1817635
115983	610	0	0	14876	0	0
3	1	4	26	2	12	56
9	2	0	0	3	0	0
1	1	1	1	1	1	1
1	1	0	0	1	0	0
1	1	1	1	1	1	1
1			1			1
						1

		1	1		1	1
1	1			1		
0	0	0	0	0	0	0
		9320	249099		29164	1817635
83208	103			5260		
			1			1
1						
0			0			0
			249099			1817635
83208						
						1
						0
						1817635
	16.9%					
	16.9%			35.4%		
		100.0%	100.0%		100.0%	100.0%
	16.9%					
	16.9%			35.4%		
		100.0%	100.0%		100.0%	100.0%
			100.0%			100.0%

BMJ Publishing Group Limited (BMJ) disclaims all liability and responsibility arising from any reliance placed on this supplemental material which has been supplied by the author(s)

100.0%

Candlelighte	Cara Trust	Cardiac Risk i	Cardiomyopa	Catholics for	Centre for Al	Changing Fac
1	5	7	5	1	1	8
0	5	0	0	0	4	3
1	1	1	1	1	1	1
0	1	0	0	0	1	1
1	1	1	1	1	1	1
	1	1	1		1	1
	1					
1		1	1	1		1
					1	
1		7	5	1		5
					3	
	1					
		1	1			1
					1	
		7	5			5
					3	

	0.0%					
	0.0%					
	0.0%					
100.0%		100.0%	100.0%	100.0%		
	0.0%					
	0.0%					
	0.0%					
		100.0%	100.0%			

0	15570	6271	2338	4089	253	32753
0	20523	0	0	0	303	10000
0	5	7	5	1	1	8
0	4	0	0	0	1	1
0	1	1	1	1	1	1
0	1	0	0	0	1	1
	1	1	1	1	1	1

FALSE						
		1	1	1		1
	1				1	
	0	0	0	0	0	0
		6271	2338	4089		22753
	4953				51	
					16.7%	
	24.1%				16.7%	
		100.0%	100.0%	100.0%		
					16.7%	
	24.1%				16.7%	
		100.0%	100.0%	100.0%		

Charlie Walle	Child Growth	Children Livi	Children with	Children with	Children's Bo	Children's Liv
1	9	9	1	2	4	2
0	3	14	1	0	9	0
1	1	1	1	1	1	1
0	1	1	1	0	1	0
1	1	1	1	1	1	1
	1	1		1	1	1
		1				
			1			
1	1			1		1
		1			1	
1	6			2		2
		5			5	
	1			1		1
		1			1	
	6			2		2
		5			5	
		1				
		5				

			0.0%			
			0.0%			
		35.7%	0.0%			
100.0%				100.0%		100.0%
		35.7%				
				100.0%		100.0%
		35.7%				

1000	14236	31845	20180	625	33966	16010
0	0	23564	20099	0	32665	0
1	9	9	1	2	4	2
0	0	8	1	0	2	0
1	1	1	1	1	1	1
0	0	1	1	0	1	0
1	1	1	1	1	1	1

1	1	1	1	1	1	1
0	0	0	0	0	0	0
1000	14236	8282	81	625	1300	16010
			0.4%		3.8%	
			0.4%		3.8%	
		26.0%	0.4%		3.8%	
100.0%	100.0%			100.0%		100.0%
			0.4%		3.8%	
			0.4%		3.8%	
		26.0%	0.4%		3.8%	
100.0%	100.0%			100.0%		100.0%

Children's He	Children's HI	Chinese Nati	Chronic Lymp	Chronic Myel	Cleft Lip and	CLIC Sargent
1	10	1	12	26	2	3
0	13	0	0	16	0	0
1	1	1	1	1	1	1
0	1	0	0	1	0	0
1	1	1	1	1	1	1
	1		1	1	1	1
	1		1	1		
1		1	1	1	1	1
	1					
1		1	12	10	2	3
	3					
			1	1	1	1
	1					
			12	10	2	3
	3					
			1	1		
	1					
			12	10		
	3					

	23.1%			38.5%		
100.0%		100.0%	100.0%		100.0%	100.0%
	23.1%			38.5%		
			100.0%		100.0%	100.0%
	23.1%			38.5%		
			100.0%			
736	49078	253	86711	180581	121	5412
0	0	0	0	185184	0	0
1	10	1	12	25	2	3
0	0	0	0	16	0	0
1	1	1	1	1	1	1
0	0	0	0	1	0	0
1	1	1	1	1	1	1
				1		

1	1	1	1		1	1
				1		
0	0	0	0	0	0	0
736	49078	253	86711		121	5412
				4603		
				1		
				0		
				4603		
				2.5%		
				2.5%		
				2.5%		
100.0%	100.0%	100.0%	100.0%		100.0%	100.0%
				2.5%		
				2.5%		
				2.5%		
100.0%	100.0%	100.0%	100.0%		100.0%	100.0%
				2.5%		
				2.5%		
				2.5%		

Coeliac UK	Community (	Community H	Community L	Confederatio	Contact a Far	Coping with (
6	1	1	1	2	3	1
2	0	1	2	2	0	0
1	1	1	1	1	1	1
1	0	1	1	1	0	0
1	1	1	1	1	1	1
1			1	1	1	
		1		1		
1	1				1	1
			1			
4	1				3	1
			1			
				1		
1					1	
			1			
4					3	
			1			

		0.0%		0.0%		
		0.0%		0.0%		
		0.0%		0.0%		
	100.0%				100.0%	100.0%
				0.0%		
				0.0%		
				0.0%		
					100.0%	

33029	1029	3042	6060	72000	20369	4187
32003	0	3042	10051	0	0	0
6	1	1	1	2	3	1
2	0	1	2	0	0	0
1	1	1	1	1	1	1
1	0	1	1	0	0	0
1	1	1	1	1	1	1

		1				
1	1			1	1	1
			1			
0	0	0	0	0	0	0
1027	1029			72000	20369	4187
			3991			
3.1%		0.0%				
3.1%		0.0%				
3.1%		0.0%	39.7%			
	100.0%			100.0%	100.0%	100.0%
3.1%		0.0%				
3.1%		0.0%				
3.1%		0.0%	39.7%			
	100.0%			100.0%	100.0%	100.0%

Coppafeel!	Core Charity	Crohn's and C	Crohn's in Ch	Cure and Act	Cure Parkins	Cystic Fibrosi
1	5	49	9	2	9	17
1	3	35	0	1	0	0
1	1	1	1	1	1	1
1	1	1	0	1	0	0
1	1	1	1	1	1	1
	1	1	1	1	1	1
		1				1
1						
	1	1	1	1	1	1
	2	14	9	1	9	17
	1	1	1	1	1	1
	2	14	9	1	9	17
		1				1
		14				17

0.0%						
0.0%						
0.0%	40.0%	28.6%				
			100.0%		100.0%	100.0%
	40.0%	28.6%				
			100.0%		100.0%	100.0%
		28.6%				
						100.0%
10000	44609	593275	27581	120	24749	281197
10100	0	0	0	9447	0	0
1	5	49	9	2	9	17
1	0	0	0	1	0	0
1	1	1	1	1	1	1
1	0	0	0	1	0	0
1	1	1	1	1	1	1
		1				1

	1	1	1		1	1
1				1		
0	0	0	0	0	0	0
	44609	593275	27581		24749	281197
100				9327		
		1				1
		0				0
		593275				281197
1.0%						
1.0%						
1.0%						
	100.0%	100.0%	100.0%		100.0%	100.0%
1.0%						
1.0%						
1.0%						
	100.0%	100.0%	100.0%		100.0%	100.0%
		100.0%				100.0%

David Lewis (	Deaf Educati	Delete Blood	Dementia UK	Dermatrust	Diabetes Res	Diabetes UK
1	1	3	1	1	3	210
0	0	0	0	2	0	11
1	1	1	1	1	1	1
0	0	0	0	1	0	1
1	1	1	1	1	1	1
		1		1	1	1
						1
						1
1	1	1	1		1	1
				1		
1	1	3	1		3	199
				1		
		1			1	1
				1		
		3			3	199
				1		
						1
						199
						1

						199
100.0%	100.0%	100.0%	100.0%		100.0%	
		100.0%			100.0%	
1892	526	14665	2593	29823	6595	3050180
0	0	0	0	45823	0	0
1	1	3	1	1	3	209
0	0	0	0	2	0	0
1	1	1	1	1	1	1
0	0	0	0	1	0	0
1	1	1	1	1	1	1
						1
						1

1	1	1	1	1	1	1
0	0	0	0	0	0	0
1892	526	14665	2593	16000	6595	3050180
						1
						0
						3050180
						1
						0
						3050180
100.0%	100.0%	100.0%	100.0%	34.9%	100.0%	100.0%
100.0%	100.0%	100.0%	100.0%	34.9%	100.0%	100.0%
						100.0%

100.0%

Diabetics wit	Diamond Bla	Different Str	Disabled Livi	Douglas Bade	Edinburgh an	Education fo
4	1	5	6	2	2	3
0	0	0	2	0	0	8
1	1	1	1	1	1	1
0	0	0	1	0	0	1
1	1	1	1	1	1	1
1		1	1	1	1	1
1	1	1	1	1	1	1
4	1	5	4	2	2	5
1		1	1	1	1	1
4		5	4	2	2	5

100.0%	100.0%	100.0%		100.0%	100.0%	
100.0%		100.0%		100.0%	100.0%	
1547	15150	10787	23840	1384	804	5343
0	0	0	0	0	0	45921
4	1	5	6	2	2	3
0	0	0	0	0	0	3
1	1	1	1	1	1	1
0	0	0	0	0	0	1
1	1	1	1	1	1	1

1	1	1	1	1	1	
						1
0	0	0	0	0	0	0
1547	15150	10787	23840	1384	804	
						40578
100.0%	100.0%	100.0%	100.0%	100.0%	100.0%	
100.0%	100.0%	100.0%	100.0%	100.0%	100.0%	

Encephalitis	Endometrios	Epilepsy Acti	Epilepsy Con	Epilepsy Scot	Epilepsy Soci	European Pa
2	2	9	2	7	18	4
0	5	7	0	0	15	11
1	1	1	1	1	1	1
0	1	1	0	0	1	1
1	1	1	1	1	1	1
1	1	1	1	1	1	1
					1	1
1		1	1	1	1	
	1					1
2		2	2	7	3	
	3					7
1		1	1	1	1	
	1					1
2		2	2	7	3	
	3					7
					1	
						1
					3	
						7

					16.7%	
		22.2%			16.7%	
100.0%			100.0%	100.0%		
					16.7%	
		22.2%			16.7%	
100.0%			100.0%	100.0%		
					16.7%	
					16.7%	
767	21096	92855	4085	6117	188332	64681
0	21096	199382	0	0	111712	239475
2	2	9	2	7	18	4
0	2	6	0	0	3	7
1	1	1	1	1	1	1
0	1	1	0	0	1	1
1	1	1	1	1	1	1
		1			1	1

	1					
1			1	1	1	
		1				1
0	0	0	0	0	0	0
767			4085	6117	76620	
		106527				174794
					1	
		1				1
		0			0	0
					76620	
		106527				174794
	0.0%					
	0.0%					
	0.0%				40.7%	
100.0%			100.0%	100.0%		
	0.0%					
	0.0%					
	0.0%				40.7%	
100.0%			100.0%	100.0%		
					40.7%	

Family Plann	Fertility Netw	Findacure	Food Chain	FORCE	Forum Link	Foundation f
12	17	6	3	1	6	1
1	17	7	1	0	0	1
1	1	1	1	1	1	1
1	1	1	1	0	0	1
1	1	1	1	1	1	1
1	1	1	1		1	
1	1					
	1					1
1			1	1	1	
		1				
11			2	1	6	
		1				
	1					
1			1		1	
		1				
11			2		6	
		1				
	1					
1						
11						

	0.0%					0.0%
	0.0%	14.3%				0.0%
	0.0%	14.3%				0.0%
				100.0%	100.0%	
	0.0%					
	0.0%	14.3%				
	0.0%	14.3%				
					100.0%	
	0.0%					
	0.0%					
	0.0%					
107247	189815	27591	13092	507	26164	57537
15150	56317	46176	5000	0	0	0
11	17	6	3	1	6	1
1	5	7	1	0	0	0
1	1	1	1	1	1	1
1	1	1	1	0	0	0
1	1	1	1	1	1	1
1	1					

1	1		1	1	1	1
		1				
0	0	0	0	0	0	0
92097	133497		8092	507	26164	57537
		18585				
1	1					
0	0					
92097	133497					
		40.2%				
				100.0%	100.0%	100.0%
		40.2%				
				100.0%	100.0%	100.0%

Freshwinds	Friends of FO	Fungal Infect	Gauchers Ass	Gay Men's H	Genetic Allia	George Hous
2	1	8	29	13	67	2
0	1	11	6	3	86	1
1	1	1	1	1	1	1
0	1	1	1	1	1	1
1	1	1	1	1	1	1
1		1	1	1	1	1
		1	1	1	1	
	1					
1			1	1		1
		1			1	
2			23	10		1
		3			19	
1			1	1		1
		1			1	
2			23	10		1
		3			19	
			1	1		
		1			1	
			23	10		
		3			19	

	0.0%					
	0.0%					
	0.0%	27.3%			22.1%	
100.0%						
		27.3%			22.1%	
100.0%						
		27.3%			22.1%	
11930	0	264961	182227	93592	616355	21000
0	0	307187	0	69319	235212	1000
2	0	8	29	13	66	2
0	0	10	0	3	12	1
1	0	1	1	1	1	1
0	0	1	0	1	1	1
1		1	1	1	1	1
		1	1		1	

	FALSE					
1			1	1	1	1
		1				
0		0	0	0	0	0
11930			182227	24273	381142	20000
		42226				
			1		1	
		1				
		0	0		0	
			182227		381142	
		42226				
		13.7%				
		13.7%		25.9%		
100.0%			100.0%			
		13.7%				
		13.7%		25.9%		
100.0%			100.0%			
		13.7%				
		13.7%				
			100.0%			

GIST Support	Groundswell	Group B Stre	Guillain Barr	Haemophilia	Haemophilia	Haemophilia
21	1	1	2	17	57	10
6	1	0	0	2	0	0
1	1	1	1	1	1	1
1	1	0	0	1	0	0
1	1	1	1	1	1	1
1			1	1	1	1
1				1	1	
	1					
1		1	1	1	1	1
15		1	2	15	57	10
1			1	1	1	1
15			2	15	57	10
1				1	1	
15				15	57	

	0.0%					
	0.0%					
	0.0%					
		100.0%	100.0%		100.0%	100.0%
			100.0%		100.0%	100.0%
					100.0%	
103706	3042	154	6400	47143	577522	13721
53906	0	0	0	25605	0	0
21	1	1	2	16	56	10
6	0	0	0	2	0	0
1	1	1	1	1	1	1
1	0	0	0	1	0	0
1	1	1	1	1	1	1
1					1	

1	1	1	1	1	1	1
0	0	0	0	0	0	0
49800	3042	154	6400	21538	577522	13721
1					1	
0					0	
49800					577522	
48.0%	100.0%	100.0%	100.0%	45.7%	100.0%	100.0%
48.0%	100.0%	100.0%	100.0%	45.7%	100.0%	100.0%
48.0%					100.0%	

Hafal	Headway	Health and S	Heart of Mer	Heart UK	Heartbeat	Hepatitis B P
1	1	1	2	94	1	1
0	0	0	2	5	0	0
1	1	1	1	1	1	1
0	0	0	1	1	0	0
1	1	1	1	1	1	1
			1	1		
				1		
			1			
1	1	1		1	1	1
1	1	1		89	1	1
			1			
				1		
				89		
				1		
				89		

			0.0%			
			0.0%			
			0.0%			
100.0%	100.0%	100.0%			100.0%	100.0%
			0.0%			
			0.0%			
			0.0%			

4000	150	38	31563	909013	758	5070
0	0	0	15781	0	0	0
1	1	1	2	90	1	1
0	0	0	1	0	0	0
1	1	1	1	1	1	1
0	0	0	1	0	0	0
1	1	1	1	1	1	1
				1		

1	1	1	1	1	1	1
0	0	0	0	0	0	0
4000	150	38	15781	909013	758	5070
				1		
				0		
				909013		
100.0%	100.0%	100.0%		100.0%	100.0%	100.0%
100.0%	100.0%	100.0%		100.0%	100.0%	100.0%
				100.0%		

Hepatitis C Tr	Hereditary A	Herts Aid	Hidradenitis	HIV i-Base	HIV Scotland	Hughes Synd
84	15	4	7	13	3	1
25	6	2	2	0	3	0
1	1	1	1	1	1	1
1	1	1	1	0	1	0
1	1	1	1	1	1	1
1	1	1	1	1	1	
1	1			1		
					1	
1	1	1	1	1		1
59	9	2	5	13		1
					1	
1	1	1	1	1		
59	9	2	5	13		
1	1			1		
59	9			13		

					0.0%	
					0.0%	
					0.0%	
				100.0%		100.0%
					0.0%	
					0.0%	
					0.0%	
				100.0%		
				100.0%		

1022603	200669	11529	32910	63462	23200	2104
1566080	194617	8050	25750	0	20509	0
83	15	4	7	13	3	1
25	6	2	2	0	3	0
1	1	1	1	1	1	1
1	1	1	1	0	1	0
1	1	1	1	1	1	1
1	1					
1						

	1	1	1	1	1	1
1						
0	0	0	0	0	0	0
	6051	3479	7160	63462	2691	2104
543477						
	1					
1						
0	0					
	6051					
543477						
1						
0						
543477						
	3.0%					
	3.0%				11.6%	
34.7%	3.0%	30.2%	21.8%		11.6%	
				100.0%		100.0%
	3.0%					
	3.0%				11.6%	
34.7%	3.0%	30.2%	21.8%		11.6%	
				100.0%		100.0%
	3.0%					
	3.0%					
34.7%	3.0%					

34.7%

Huntington's	IBS Network	ICUsteps	Immune Thr	Imperial Coll	Independent	Input Diabet
1	1	2	15	1	11	1
3	4	0	29	0	0	0
1	1	1	1	1	1	1
1	1	0	1	0	0	0
1	1	1	1	1	1	1
1	1	1	1		1	
			1		1	
		1		1	1	1
1	1		1			
		2		1	11	1
2	3		14			
		1			1	
1	1		1			
		2			11	
2	3		14			
					1	
			1			
					11	
			14			

			48.3%			
		100.0%		100.0%	100.0%	100.0%
			48.3%			
		100.0%			100.0%	
			48.3%			
					100.0%	
30159	8417	4157	67352	4000	29891	30322
0	0	0	44323	0	0	0
1	1	2	15	1	11	1
0	0	0	8	0	0	0
1	1	1	1	1	1	1
0	0	0	1	0	0	0
1	1	1	1	1	1	1

1	1	1	1	1	1	1
0	0	0	0	0	0	0
30159	8417	4157	23028	4000	29891	30322
100.0%	100.0%	100.0%	34.2%	100.0%	100.0%	100.0%
100.0%	100.0%	100.0%	34.2%	100.0%	100.0%	100.0%

International	International	International	International	International	Jo's Cervical	Joint Epileps
23	2	9	1	10	2	1
39	1	19	8	31	14	4
1	1	1	1	1	1	1
1	1	1	1	1	1	1
1	1	1	1	1	1	1
1	1	1	1	1	1	1
1		1		1	1	
	1					
1		1	1	1	1	1
	1					
16		10	7	21	12	3
	1					
1		1	1	1	1	1
	1					
16		10	7	21	12	3
1		1		1	1	
16		10		21	12	

41.0%

41.0%

41.0%

652377	73355	24458	18514	222272	25150	12856
873965	0	0	0	1681140	36360	0
23	2	9	1	10	2	1
39	0	0	0	31	2	0
1	1	1	1	1	1	1
1	0	0	0	1	1	0
1	1	1	1	1	1	1
1				1		
				1		

	1	1	1			1
1				1	1	
0	0	0	0	0	0	0
	73355	24458	18514			12856
221588				1458868	11210	
1				1		
0				0		
221588				1458868		
				1		
				0		
				1458868		
25.4%					30.8%	
	100.0%	100.0%	100.0%			100.0%
25.4%					30.8%	
	100.0%	100.0%	100.0%			100.0%
25.4%						

Patient organisation names						
Juvenile Diab	KAITY	Karen Cliffor	Kent Associa	Kidney Cance	Kidney Cance	Kidney Care U
19	1	10	1	9	37	2
6	0	0	2	0	0	0
1	1	1	1	1	1	1
1	0	0	1	0	0	0
1	1	1	1	1	1	1
1		1	1	1	1	1
1					1	
1	1	1		1	1	1
			1			
13	1	10		9	37	2
			1			
1		1		1	1	1
			1			
13		10		9	37	2
			1			
1					1	
13					37	

	100.0%	100.0%		100.0%	100.0%	100.0%
		100.0%		100.0%	100.0%	100.0%
					100.0%	
417065	2000	91665	18513	101336	183714	754
10563	0	0	18938	0	0	0
19	1	9	1	8	35	2
1	0	0	1	0	0	0
1	1	1	1	1	1	1
1	0	0	1	0	0	0
1	1	1	1	1	1	1
1				1	1	

1	1	1		1	1	1
			1			
0	0	0	0	0	0	0
406503	2000	91665		101336	183714	754
			424			
1				1	1	
0				0	0	
406503				101336	183714	
			2.2%			
			2.2%			
			2.2%			
	100.0%	100.0%		100.0%	100.0%	100.0%
			2.2%			
			2.2%			
			2.2%			
	100.0%	100.0%		100.0%	100.0%	100.0%
				100.0%	100.0%	

Kidney Resea	KIDS	KwaAfrica	LATCH Welsh	Laurence Mo	Leeds Teachi	Leicestershir
17	1	1	1	1	1	4
47	1	1	0	1	0	0
1	1	1	1	1	1	1
1	1	1	0	1	0	0
1	1	1	1	1	1	1
1						1
1						
	1	1		1		
			1		1	1
1						
			1		1	4
30						
						1
1						
						4
30						
1						
30						

	0.0%	0.0%		0.0%		
	0.0%	0.0%		0.0%		
	0.0%	0.0%		0.0%		
			100.0%		100.0%	100.0%
						100.0%
178461	7676	5000	1010	1010	354	22216
3144648	7676	5000	0	0	0	0
17	1	1	1	1	1	4
47	1	1	0	0	0	0
1	1	1	1	1	1	1
1	1	1	0	0	0	0
1	1	1	1	1	1	1
1						
1						

	1	1				
			1	1	1	1
1						
0	0	0	0	0	0	0
			1010	1010	354	22216
2966187						
1						
0						
2966187						
1						
0						
2966187						
	0.0%	0.0%				
	0.0%	0.0%				
	0.0%	0.0%				
			100.0%	100.0%	100.0%	100.0%
	0.0%	0.0%				
	0.0%	0.0%				
	0.0%	0.0%				
			100.0%	100.0%	100.0%	100.0%

Leukaemia C/	Life Educatio	Little Hearts	Liver4Life	Lullaby Trust	Lupus Europe	Lupus UK
62	1	1	24	1	4	1
39	0	0	0	0	0	0
1	1	1	1	1	1	1
1	0	0	0	0	0	0
1	1	1	1	1	1	1
1			1		1	
1			1			
1	1	1	1	1	1	1
23	1	1	24	1	4	1
1			1		1	
23			24		4	
1			1			
23			24			

37.1%						
	100.0%	100.0%	100.0%	100.0%	100.0%	100.0%
37.1%						
			100.0%		100.0%	
37.1%						
			100.0%			
472981	519	253	248299	0	55503	707
351393	0	0	0	0	0	0
61	1	1	24	0	4	1
30	0	0	0	0	0	0
1	1	1	1	0	1	1
1	0	0	0	0	0	0
1	1	1	1		1	1
1			1			

				FALSE		
1	1	1	1		1	1
0	0	0	0		0	0
121588	519	253	248299		55503	707
1			1			
0			0			
121588			248299			
25.7%						
	100.0%	100.0%	100.0%		100.0%	100.0%
25.7%						
	100.0%	100.0%	100.0%		100.0%	100.0%
25.7%						
			100.0%			

Lymphoma A	Macmillan Ca	Macular Soci	Maggie's Cen	Make A Wish	Malnutrition	MAMA Acad
58	50	1	3	2	1	1
84	2	0	0	0	0	0
1	1	1	1	1	1	1
1	1	0	0	0	0	0
1	1	1	1	1	1	1
1	1		1	1		
1	1					
	1	1	1	1	1	1
1						
	48	1	3	2	1	1
26						
	1		1	1		
1						
	48		3	2		
26						
	1					
1						
	48					
26						

31.0%						
		100.0%	100.0%	100.0%	100.0%	100.0%
31.0%						
			100.0%	100.0%		
31.0%						
158813	190660	15	3413	765	884	202
31792	0	0	0	0	0	0
57	50	1	3	2	1	1
9	0	0	0	0	0	0
1	1	1	1	1	1	1
1	0	0	0	0	0	0
1	1	1	1	1	1	1
1	1					

1	1	1	1	1	1	1
0	0	0	0	0	0	0
127022	190660	15	3413	765	884	202
1	1					
0	0					
127022	190660					
	100.0%	100.0%	100.0%	100.0%	100.0%	100.0%
	100.0%	100.0%	100.0%	100.0%	100.0%	100.0%
	100.0%					

Manchester	Marie Curie	Martin Fishe	MDS UK Sup	Medical Four	Melanoma F	Melanoma F
3	3	1	13	5	4	3
2	0	0	6	3	1	1
1	1	1	1	1	1	1
1	0	0	1	1	1	1
1	1	1	1	1	1	1
1	1		1	1	1	1
			1			
1	1	1	1	1	1	1
1	3	1	7	2	3	2
1	1		1	1	1	1
1	3		7	2	3	2
			1			
			7			

33.3%				40.0%		
	100.0%	100.0%				

33.3%				40.0%		
	100.0%					

19538	643	15000	145332	58641	61418	23080
9438	0	0	131702	63480	0	5050
3	3	1	13	5	4	3
2	0	0	6	3	0	1
1	1	1	1	1	1	1
1	0	0	1	1	0	1

1	1	1	1	1	1	1
			1			

1	1	1	1	1	1	1
0	0	0	0	0	0	0
10100	643	15000	13630	4840	61418	18030
			1			
			0			
			13630			
			9.4%	7.6%		
			9.4%	7.6%		
			9.4%	7.6%		
	100.0%	100.0%			100.0%	
			9.4%	7.6%		
			9.4%	7.6%		
			9.4%	7.6%		
	100.0%	100.0%			100.0%	
			9.4%			
			9.4%			
			9.4%			

Melanoma U	Men’s Health	Meningitis N	Meningitis R	Mental Healt	Mesotheliom	Migraine Acti
24	14	33	60	8	7	8
0	5	20	13	1	0	1
1	1	1	1	1	1	1
0	1	1	1	1	0	1
1	1	1	1	1	1	1
1	1	1	1	1	1	1
1	1	1	1			
1	1	1	1	1	1	1
24	9	13	47	7	7	7
1	1	1	1	1	1	1
24	9	13	47	7	7	7
1	1	1	1			
24	9	13	47			

		39.4%				
100.0%					100.0%	
		39.4%				
100.0%					100.0%	
		39.4%				
100.0%						
386833	94419	532482	700445	168898	34571	107846
0	64124	266721	216480	5143	0	0
24	11	32	59	8	7	8
0	5	16	8	1	0	0
1	1	1	1	1	1	1
0	1	1	1	1	0	0
1	1	1	1	1	1	1
1		1	1	1		1

1	1	1	1	1	1	1
0	0	0	0	0	0	0
386833	30294	265761	483965	163756	34571	107846
1		1	1	1		1
0		0	0	0		0
386833		265761	483965	163756		107846
	32.1%	49.9%				
100.0%					100.0%	100.0%
	32.1%	49.9%				
100.0%					100.0%	100.0%
		49.9%				
100.0%						100.0%

Migraine Tru	Motor Neuro	Mouth Cance	Movember F	MPN Voice	Multi Organ '	Multiple Birt
9	2	1	1	6	1	1
9	0	2	0	0	0	0
1	1	1	1	1	1	1
1	0	1	0	0	0	0
1	1	1	1	1	1	1
1	1	1		1		
1						
	1		1	1	1	1
		1				
	2		1	6	1	1
		1				
1						
	1			1		
		1				
	2			6		
		1				

0.0%						
0.0%						
0.0%						
	100.0%		100.0%	100.0%	100.0%	100.0%

0.0%						
0.0%						
0.0%						
	100.0%			100.0%		

308512	280	1000	1010	44237	6084	505
0	0	0	0	0	0	0
9	2	1	1	6	1	1
0	0	0	0	0	0	0
1	1	1	1	1	1	1
0	0	0	0	0	0	0
1	1	1	1	1	1	1
1						

1	1	1	1	1	1	1
0	0	0	0	0	0	0
308512	280	1000	1010	44237	6084	505
1						
0						
308512						
100.0%	100.0%	100.0%	100.0%	100.0%	100.0%	100.0%
100.0%	100.0%	100.0%	100.0%	100.0%	100.0%	100.0%
100.0%						

Multiple Scle	Multiple Scle	Multiple Scle	Multiple Scle	Muscular Dys	Myasthenia (	Myeloma UK
7	38	62	1	3	1	53
28	24	18	0	10	0	15
1	1	1	1	1	1	1
1	1	1	0	1	0	1
1	1	1	1	1	1	1
1	1	1		1		1
1	1	1				1
	1	1	1		1	1
1				1		
	14	44	1		1	38
21				7		
	1	1				1
1				1		
	14	44				38
21				7		
	1	1				1
1						
	14	44				38
21						

	36.8%					
			100.0%		100.0%	
	36.8%					
	36.8%					
317100	282676	533370	603	17028	2020	7430106
2524665	564884	55200	0	0	0	711530
7	38	62	1	3	1	52
28	24	5	0	0	0	15
1	1	1	1	1	1	1
1	1	1	0	0	0	1
1	1	1	1	1	1	1
1	1	1				1
1						1

		1	1	1	1	1
1	1					
0	0	0	0	0	0	0
		478170	603	17028	2020	6718577
2207565	282208					
		1				1
1	1					
0	0	0				0
		478170				6718577
2207565	282208					
						1
1						
0						0
						6718577
2207565						
	50.0%					
			100.0%	100.0%	100.0%	
	50.0%					
			100.0%	100.0%	100.0%	
	50.0%					

Myrovlytis T	NARA - The E	National AID	National AID	National Ank	National Ass	National Atte
1	1	41	28	55	1	9
0	0	7	17	33	0	7
1	1	1	1	1	1	1
0	0	1	1	1	0	1
1	1	1	1	1	1	1
		1	1	1		1
		1	1	1		
1	1	1	1	1	1	1
1	1	34	11	22	1	2
		1	1	1		1
		34	11	22		2
		1	1	1		
		34	11	22		

			39.3%	40.0%		22.2%
100.0%	100.0%				100.0%	
			39.3%	40.0%		22.2%
			39.3%	40.0%		
1420	0	454331	284499	902728	152	12006
0	0	198306	316593	527875	0	27951
1	0	41	28	54	1	9
0	0	6	17	18	0	7
1	0	1	1	1	1	1
0	0	1	1	1	0	1
1		1	1	1	1	1
		1	1	1		

	FALSE					
1		1		1	1	
			1			1
0		0	0	0	0	0
1420		256025		374853	152	
			32094			15945
		1		1		
			1			
		0	0	0		
		256025		374853		
			32094			
			10.1%			
			10.1%	41.5%		
100.0%					100.0%	
			10.1%			
			10.1%	41.5%		
100.0%					100.0%	
			10.1%			
			10.1%	41.5%		

	National Can	National Chil	National Cou	National Ecze	National Kidr	National Obe	National Oste
	1	1	1	14	30	3	35
	0	1	3	0	0	0	0
	1	1	1	1	1	1	1
	0	1	1	0	0	0	0
	1	1	1	1	1	1	1
			1	1	1	1	1
				1	1		1
		1					
	1			1	1	1	1
			1				
	1			14	30	3	35
			2				
				1	1	1	1
			1				
				14	30	3	35
			2				
				1	1		1
				14	30		35

	0.0%					
	0.0%					
	0.0%					
100.0%			100.0%	100.0%	100.0%	100.0%
			100.0%	100.0%	100.0%	100.0%
			100.0%	100.0%		100.0%
1000	9600	10521	23676	396718	3508	326741
0	0	23780	0	0	0	0
1	1	1	14	30	3	34
0	0	3	0	0	0	0
1	1	1	1	1	1	1
0	0	1	0	0	0	0
1	1	1	1	1	1	1
				1		1

1	1	1	1	1	1	1
0	0	0	0	0	0	0
1000	9600	13259	23676	396718	3508	326741
				1		1
				0		0
				396718		326741
100.0%	100.0%		100.0%	100.0%	100.0%	100.0%
100.0%	100.0%		100.0%	100.0%	100.0%	100.0%
				100.0%		100.0%

National Rhe	National Voi	NAZ	NET Patient F	Neurological	Niemann-Pic	NMO Resear
102	12	10	28	15	12	3
20	21	4	14	31	14	0
1	1	1	1	1	1	1
1	1	1	1	1	1	0
1	1	1	1	1	1	1
1	1	1	1	1	1	1
1	1		1	1	1	
1						
1		1	1			1
	1			1	1	
82		6	14			3
	9			16	2	
1		1	1			1
	1			1	1	
82		6	14			3
	9			16	2	
1			1			
	1			1	1	
82			14			
	9			16	2	
1						

82						
					14.3%	
	42.9%				14.3%	
						100.0%
					14.3%	
	42.9%				14.3%	
						100.0%
					14.3%	
	42.9%				14.3%	
688957	87459	105845	196851	147434	129795	6650
0	212771	133957	269645	0	102765	0
100	12	10	27	15	12	3
0	15	4	14	0	12	0
1	1	1	1	1	1	1
0	1	1	1	0	1	0
1	1	1	1	1	1	1
1	1	1	1	1	1	

1				1	1	1
	1	1	1			
0	0	0	0	0	0	0
688957				147434	27030	6650
	125313	28112	72795			

1				1	1
	1	1	1		
0	0	0	0	0	0
688957				147434	27030
	125313	28112	72795		

		21.0%	27.0%		20.8%	
100.0%				100.0%		100.0%

		21.0%	27.0%		20.8%	
100.0%				100.0%		100.0%

		21.0%	27.0%		20.8%
100.0%				100.0%	

North of Engl	Northern Irel	Northern Irel	Northern Ne	OcuMel UK	Oesophageal	Older People
4	1	2	2	2	1	2
0	0	0	1	0	1	1
1	1	1	1	1	1	1
0	0	0	1	0	1	1
1	1	1	1	1	1	1
1		1	1	1		1
					1	
1	1	1	1	1		1
4	1	2	1	2		1
1		1	1	1		1
4		2	1	2		1

					0.0%	
					0.0%	
					0.0%	
100.0%	100.0%	100.0%		100.0%		

100.0%		100.0%		100.0%		

5775	1029	4035	21913	2081	1636	30300
0	0	0	21913	0	0	25351
4	1	2	1	2	1	2
0	0	0	1	0	0	1
1	1	1	1	1	1	1
0	0	0	1	0	0	1
1	1	1	1	1	1	1

			1			
1	1	1		1	1	1
0	0	0	0	0	0	0
5775	1029	4035		2081	1636	4949

			0.0%			
			0.0%			16.3%
			0.0%			16.3%
100.0%	100.0%	100.0%		100.0%	100.0%	
			0.0%			
			0.0%			16.3%
			0.0%			16.3%
100.0%	100.0%	100.0%		100.0%	100.0%	

Olive Tree Ca	Oliver King F	Orchid	Organisation	Ovacome	Ovarian Canc	Paget's Assoc
1	1	2	2	6	3	3
0	0	0	0	0	3	0
1	1	1	1	1	1	1
0	0	0	0	0	1	0
1	1	1	1	1	1	1
		1	1	1	1	1
					1	
1	1	1	1	1		1
1	1	2	2	6		3
					1	
		1	1	1		1
		2	2	6		3

					0.0%	
					0.0%	
					0.0%	
100.0%	100.0%	100.0%	100.0%	100.0%		100.0%

					0.0%	
					0.0%	
					0.0%	
		100.0%	100.0%	100.0%		100.0%

3400	511	3334	4184	64181	39552	24721
0	0	0	0	0	0	0
1	1	2	2	6	3	3
0	0	0	0	0	0	0
1	1	1	1	1	1	1
0	0	0	0	0	0	0

1	1	1	1	1	1	1

1	1	1	1	1	1	1
0	0	0	0	0	0	0
3400	511	3334	4184	64181	39552	24721
100.0%	100.0%	100.0%	100.0%	100.0%	100.0%	100.0%
100.0%	100.0%	100.0%	100.0%	100.0%	100.0%	100.0%

Pain Associat	Pain Concern	Pain Relief Fo	Pain UK	Pancreatic Ca	Pancreatic Ca	Parkinson's U
10	11	3	10	7	17	12
0	0	2	0	2	3	8
1	1	1	1	1	1	1
0	0	1	0	1	1	1
1	1	1	1	1	1	1
1	1	1	1	1	1	1
	1				1	1
1	1	1	1	1	1	1
10	11	1	10	5	14	4
1	1	1	1	1	1	1
10	11	1	10	5	14	4
	1				1	1
	11				14	4

		33.3%				33.3%
100.0%	100.0%		100.0%			
		33.3%				33.3%
100.0%	100.0%		100.0%			
						33.3%
	100.0%					
4636	16888	1442	30101	34907	209550	29736
0	0	0	0	0	0	15781
9	9	3	10	7	17	11
0	0	0	0	0	0	2
1	1	1	1	1	1	1
0	0	0	0	0	0	1
1	1	1	1	1	1	1
					1	

1	1	1	1	1	1	1
0	0	0	0	0	0	0
4636	16888	1442	30101	34907	209550	13955
					1	
					0	
					209550	
						46.9%
100.0%	100.0%	100.0%	100.0%	100.0%	100.0%	
						46.9%
100.0%	100.0%	100.0%	100.0%	100.0%	100.0%	
					100.0%	

Patients Asso	Patients On I	Paula Carr Di	PBC Foundat	Pelican Canc	Pelvic Pain Su	Pink Ribbon I
48	5	1	6	1	4	1
0	0	0	3	0	0	0
1	1	1	1	1	1	1
0	0	0	1	0	0	0
1	1	1	1	1	1	1
1	1		1		1	
1						
1	1	1	1	1	1	1
48	5	1	3	1	4	1
1	1		1		1	
48	5		3		4	
1						
48						

100.0%	100.0%	100.0%		100.0%	100.0%	100.0%
100.0%	100.0%				100.0%	
100.0%						
345520	29849	400	17418	8500	2655	10000
0	0	0	15150	0	0	0
45	5	1	6	1	4	1
0	0	0	1	0	0	0
1	1	1	1	1	1	1
0	0	0	1	0	0	0
1	1	1	1	1	1	1
1						

1	1	1	1	1	1	1
0	0	0	0	0	0	0
345520	29849	400	2268	8500	2655	10000
1						
0						
345520						
			13.0%			
			13.0%			
100.0%	100.0%	100.0%		100.0%	100.0%	100.0%
			13.0%			
			13.0%			
100.0%	100.0%	100.0%		100.0%	100.0%	100.0%
100.0%						

Pituitary Fou	PNH Support	Polycystic Ki	Portsmouth I	Positive Actio	Positive East	Positive Help
23	1	4	1	1	7	1
21	1	0	0	0	2	1
1	1	1	1	1	1	1
1	1	0	0	0	1	1
1	1	1	1	1	1	1
1		1			1	
1						
	1					1
1		1	1	1	1	
2		4	1	1	5	
1		1			1	
2		4			5	
1						
2						

8.7%	0.0%					0.0%
8.7%	0.0%					0.0%
8.7%	0.0%					0.0%
		100.0%	100.0%	100.0%		
8.7%						
8.7%						
8.7%						
		100.0%				
8.7%						
8.7%						
8.7%						

110099	15150	68956	152	0	42960	3042
42209	15150	0	0	0	32097	15261
23	1	4	1	0	7	1
4	1	0	0	0	2	1
1	1	1	1	0	1	1
1	1	0	0	0	1	1
1	1	1	1		1	1
1						

	1			FALSE		
1		1	1		1	
						1
0	0	0	0		0	0
67891		68956	152		10863	
						12218
1						
0						
67891						

0.0%				
0.0%				
0.0%			25.3%
	100.0%	100.0%		
0.0%				
0.0%				
0.0%			25.3%
	100.0%	100.0%		

Positively UK	Prader-Willi S	Primary Imm	Progress Edu	Progressive S	Prostate Can	Prostate Can
31	1	8	2	3	1	2
20	0	8	10	0	0	4
1	1	1	1	1	1	1
1	0	1	1	0	0	1
1	1	1	1	1	1	1
1		1	1	1		1
1						
		1				
1	1			1	1	
			1			1
11	1			3	1	
			8			2
		1				
1				1		
			1			1
11				3		
			8			2
1						
11						

		0.0%				
		0.0%				
35.5%		0.0%				
	100.0%			100.0%	100.0%	

		0.0%				
		0.0%				
35.5%		0.0%				
				100.0%		

35.5%						

130874	473	73527	1373	564	1052	2088
146722	0	69182	0	0	0	0
31	1	8	2	3	1	2
20	0	8	0	0	0	0
1	1	1	1	1	1	1
1	0	1	0	0	0	0

1	1	1	1	1	1	1
1						

	1	1	1	1	1	1
1						
0	0	0	0	0	0	0
	473	4345	1373	564	1052	2088
15848						
1						
0						
15848						
		5.9%				
10.8%		5.9%				
10.8%		5.9%				
	100.0%		100.0%	100.0%	100.0%	100.0%
		5.9%				
10.8%		5.9%				
10.8%		5.9%				
	100.0%		100.0%	100.0%	100.0%	100.0%
10.8%						
10.8%						

Prostate Can(	Prostate Cym	Psoriasis Ass(	Pulmonary H	Pumping Ma	QUIT	Rainbow Cen
15	1	37	18	29	13	1
2	0	70	13	9	0	0
1	1	1	1	1	1	1
1	0	1	1	1	0	0
1	1	1	1	1	1	1
1		1	1	1	1	
1		1	1	1	1	
1	1		1	1	1	1
		1				
13	1		5	20	13	1
		33				
1			1	1	1	
		1				
13			5	20	13	
		33				
1			1	1	1	
		1				
13			5	20	13	
		33				

		47.1%	27.8%			
	100.0%				100.0%	100.0%
		47.1%	27.8%			
					100.0%	
		47.1%	27.8%			
					100.0%	
108777	640	427224	274170	307930	79340	579
0	0	0	278788	96358	0	0
14	1	37	18	29	13	1
0	0	0	13	5	0	0
1	1	1	1	1	1	1
0	0	0	1	1	0	0
1	1	1	1	1	1	1
1		1	1	1		

1	1	1		1	1	1
			1			
0	0	0	0	0	0	0
108777	640	427224		211573	79340	579
			4618			
1		1		1		
			1			
0		0	0	0		
108777		427224		211573		
			4618			
			1.7%			
			1.7%			
			1.7%			
100.0%	100.0%	100.0%			100.0%	100.0%
			1.7%			
			1.7%			
			1.7%			
100.0%	100.0%	100.0%			100.0%	100.0%
			1.7%			
			1.7%			
			1.7%			
100.0%		100.0%				

Rainbow Tru	Rapid Effecti	Rarer Cancer	Raynaud's ar	Release	Restless Leg	Rethink Men
1	4	62	14	1	5	4
0	2	0	4	1	0	8
1	1	1	1	1	1	1
0	1	0	1	1	0	1
1	1	1	1	1	1	1
	1	1	1		1	1
		1	1			
				1		
1	1	1	1		1	
						1
1	2	62	10		5	
						4
	1	1	1		1	
						1
	2	62	10		5	
						4
		1	1			
		62	10			

				0.0%		
				0.0%		
				0.0%		
100.0%		100.0%			100.0%	
		100.0%			100.0%	
		100.0%				
1010	10915	682971	29651	10063	12940	61642
0	0	0	0	5143	0	12625
1	4	62	13	1	5	4
0	0	0	0	1	0	1
1	1	1	1	1	1	1
0	0	0	0	1	0	1
1	1	1	1	1	1	1
		1				

1	1	1	1	1	1	1
0	0	0	0	0	0	0
1010	10915	682971	29651	4921	12940	49017
		1				
		0				
		682971				
				48.9%		
100.0%	100.0%	100.0%	100.0%		100.0%	
				48.9%		
100.0%	100.0%	100.0%	100.0%		100.0%	
		100.0%				

Revive Multi	Richmond Fe	River House	Ronald McDc	Roy Castle Lu	Royal Free Cl	Royal Marsd
3	1	1	1	70	2	1
1	0	0	0	0	3	0
1	1	1	1	1	1	1
1	0	0	0	0	1	0
1	1	1	1	1	1	1
1				1	1	
				1		
1	1	1	1	1		1
					1	
2	1	1	1	70		1
					1	
1				1		
					1	
2				70		
					1	
				1		
				70		

					33.3%	
	100.0%	100.0%	100.0%	100.0%		100.0%
					33.3%	
				100.0%		
				100.0%		
109067	736	253	771	770325	11827	355
29239	0	0	0	0	0	0
3	1	1	1	69	2	1
1	0	0	0	0	0	0
1	1	1	1	1	1	1
1	0	0	0	0	0	0
1	1	1	1	1	1	1
1				1		

1	1	1	1	1	1	1
0	0	0	0	0	0	0
79828	736	253	771	770325	11827	355
1				1		
0				0		
79828				770325		
	100.0%	100.0%	100.0%	100.0%	100.0%	100.0%
	100.0%	100.0%	100.0%	100.0%	100.0%	100.0%
				100.0%		

Royal Nationa	Salamander T	Samson Cent	SANE	Sarcoma UK	Save Babies T	Saving Lives
43	2	1	7	6	1	3
23	0	0	0	8	1	6
1	1	1	1	1	1	1
1	0	0	0	1	1	1
1	1	1	1	1	1	1
1	1		1	1		1
1						
					1	
1	1	1	1			
				1		1
20	2	1	7			
				2		3
1	1		1			
				1		1
20	2		7			
				2		3
1						
20						

					0.0%	
					0.0%	
46.5%				25.0%	0.0%	
	100.0%	100.0%	100.0%			
46.5%				25.0%		
	100.0%		100.0%			
46.5%						
1236891	721	4500	83687	29998	981	32786
982982	0	0	0	13677	0	70437
42	2	1	7	6	1	3
23	0	0	0	4	0	6
1	1	1	1	1	1	1
1	0	0	0	1	0	1
1	1	1	1	1	1	1
1						
1						

1	1	1	1	1	1	
						1
0	0	0	0	0	0	0
253909	721	4500	83687	16321	981	
						37650
1						
0						
253909						
1						
0						
253909						
20.5%						
	100.0%	100.0%	100.0%		100.0%	
20.5%						
	100.0%	100.0%	100.0%		100.0%	
20.5%						

20.5%

Scleroderma	Scleroderma	Scoliosis Ass	Scotland Pati	Scottish Drug	Scottish Epile	Scottish Kidn
1	5	1	1	2	2	1
3	1	0	0	0	0	0
1	1	1	1	1	1	1
1	1	0	0	0	0	0
1	1	1	1	1	1	1
1	1			1	1	
	1	1	1	1	1	1
1						
	4	1	1	2	2	1
2						
	1			1	1	
1						
	4			2	2	
2						

		100.0%	100.0%	100.0%	100.0%	100.0%
				100.0%	100.0%	
2000	4513	278	1029	4056	78625	505
11110	634	0	0	0	0	0
1	5	1	1	2	2	1
3	1	0	0	0	0	0
1	1	1	1	1	1	1
1	1	0	0	0	0	0
1	1	1	1	1	1	1

	1	1	1	1	1	1
1						
0	0	0	0	0	0	0
	3879	278	1029	4056	78625	505
9110						

100.0%	100.0%	100.0%	100.0%	100.0%
100.0%	100.0%	100.0%	100.0%	100.0%

Scottish Netv	SeeAbility	Self Help Ser	Sense	Service by En	Sexual Advic	Shift.MS
3	1	2	1	1	9	26
0	0	0	0	0	6	4
1	1	1	1	1	1	1
0	0	0	0	0	1	1
1	1	1	1	1	1	1
1		1			1	1
						1
1	1	1	1	1	1	1
3	1	2	1	1	3	22
1		1			1	1
3		2			3	22
						1
						22

					33.3%	
100.0%	100.0%	100.0%	100.0%	100.0%		
					33.3%	
100.0%		100.0%				
1139	473	514	152	210	115400	279784
0	0	0	0	0	0	0
3	1	2	1	1	8	26
0	0	0	0	0	0	0
1	1	1	1	1	1	1
0	0	0	0	0	0	0
1	1	1	1	1	1	1
					1	1

1	1	1	1	1	1	1
0	0	0	0	0	0	0
1139	473	514	152	210	115400	279784
					1	1
					0	0
					115400	279784
100.0%	100.0%	100.0%	100.0%	100.0%	100.0%	100.0%
100.0%	100.0%	100.0%	100.0%	100.0%	100.0%	100.0%
					100.0%	100.0%

Short Bowel	Sickle Cell an	Sickle Cell So	Sign Health	Silver Star	Skin Care Cyr	Society for Mu
1	10	18	1	12	8	78
0	5	8	1	0	0	12
1	1	1	1	1	1	1
0	1	1	1	0	0	1
1	1	1	1	1	1	1
	1	1		1	1	1
		1		1		1
			1			
1	1	1		1	1	1
1	5	10		12	8	66
	1	1		1	1	1
	5	10		12	8	66
		1		1		1
		10		12		66

			0.0%			
			0.0%			
			0.0%			
100.0%				100.0%	100.0%	
				100.0%	100.0%	
				100.0%		
224	28443	50877	12625	117959	19925	1031298
0	37172	41925	0	0	0	0
1	10	17	1	12	8	78
0	4	8	0	0	0	0
1	1	1	1	1	1	1
0	1	1	0	0	0	0
1	1	1	1	1	1	1
				1		1
						1

1		1	1	1	1	1
	1					
0	0	0	0	0	0	0
224		8952	12625	117959	19925	1031298
	8728					
				1		1
				0		0
				117959		1031298
						1
						0
						1031298
		17.6%				
	23.5%	17.6%				
100.0%			100.0%	100.0%	100.0%	100.0%
		17.6%				
	23.5%	17.6%				
100.0%			100.0%	100.0%	100.0%	100.0%
				100.0%		100.0%

100.0%

Somerville Fc	Sophia Forun	South Asian I	Spectra	Spinal Injurie	Spinal Injurie	Spinal Muscu
3	2	1	1	1	1	2
0	0	1	0	0	0	4
1	1	1	1	1	1	1
0	0	1	0	0	0	1
1	1	1	1	1	1	1
1	1					1
		1				
1	1		1	1	1	
						1
3	2		1	1	1	
						2
1	1					
						1
3	2					
						2

		0.0%				
		0.0%				
		0.0%				
100.0%	100.0%		100.0%	100.0%	100.0%	
100.0%	100.0%					
7328	10120	35998	1010	253	1052	46302
0	0	0	0	0	0	0
3	2	1	1	1	1	2
0	0	0	0	0	0	0
1	1	1	1	1	1	1
0	0	0	0	0	0	0
1	1	1	1	1	1	1

1	1	1	1	1	1	1
0	0	0	0	0	0	0
7328	10120	35998	1010	253	1052	46302
100.0%	100.0%	100.0%	100.0%	100.0%	100.0%	100.0%
100.0%	100.0%	100.0%	100.0%	100.0%	100.0%	100.0%

St Thomas Lu	Steps	Stick N Step	Stroke Associ	SUDEP Actio	Surrey Young	Sussex Beaco
4	1	1	39	2	1	1
0	0	1	4	0	2	0
1	1	1	1	1	1	1
0	0	1	1	0	1	0
1	1	1	1	1	1	1
1			1	1	1	
			1			
		1				
1	1		1	1		1
					1	
4	1		35	2		1
					1	
1			1	1		
					1	
4			35	2		
					1	
			1			
			35			

		0.0%				
		0.0%				
		0.0%				
100.0%	100.0%			100.0%		100.0%
100.0%				100.0%		
62950	152	1349	256932	2334	5070	1000
0	0	0	20570	0	10100	0
4	1	1	38	2	1	1
0	0	0	1	0	1	0
1	1	1	1	1	1	1
0	0	0	1	0	1	0
1	1	1	1	1	1	1
			1			

1	1	1	1	1		1
					1	
0	0	0	0	0	0	0
62950	152	1349	236362	2334		1000
					5030	
			1			
			0			
			236362			
					49.8%	
100.0%	100.0%	100.0%		100.0%		100.0%
					49.8%	
100.0%	100.0%	100.0%		100.0%		100.0%

Tackle Africa	Tackle Prosta	Target Ovaria	TB Alert	Team Margo	Teenage Can	Tenovus Canc
1	13	1	2	1	11	9
2	0	3	3	0	3	0
1	1	1	1	1	1	1
1	0	1	1	0	1	0
1	1	1	1	1	1	1
1	1	1	1		1	1
	1				1	
	1			1	1	1
1		1	1			
	13			1	8	9
1		2	1			
	1				1	1
1		1	1			
	13				8	9
1		2	1			
	1				1	
	13				8	

			33.3%			
	100.0%			100.0%		100.0%
			33.3%			
	100.0%					100.0%
	100.0%					
3042	148470	0	27401	5050	11904	170503
6072	0	0	0	0	0	0
1	13	0	2	1	10	9
2	0	0	0	0	0	0
1	1	0	1	1	1	1
1	0	0	0	0	0	0
1	1		1	1	1	1
	1					1

		FALSE				
	1		1	1	1	1
1						
0	0		0	0	0	0
	148470		27401	5050	11904	170503
3030						
	1					1
	0					0
	148470					170503
49.9%						
	100.0%		100.0%	100.0%	100.0%	100.0%
49.9%						
	100.0%		100.0%	100.0%	100.0%	100.0%
	100.0%					100.0%

Terrence Hig	Thames Valle	Thrombosis U	Tiny Tickers	Tommy's	Transplant Li	Transverse M
13	2	17	2	1	2	1
12	0	0	2	5	0	0
1	1	1	1	1	1	1
1	0	0	1	1	0	0
1	1	1	1	1	1	1
1	1	1	1	1	1	
1		1				
			1			
1	1	1			1	1
				1		
1	2	17			2	1
				4		
			1			
1	1	1			1	
				1		
1	2	17			2	
				4		
1		1				
1		17				

7.7%			0.0%			
7.7%			0.0%			
7.7%			0.0%			
	100.0%	100.0%			100.0%	100.0%
7.7%			0.0%			
7.7%			0.0%			
7.7%			0.0%			
	100.0%	100.0%			100.0%	
7.7%						
7.7%						
7.7%						
		100.0%				
108993	5736	45782	12221	10285	1532	15150
67744	0	0	0	0	0	0
13	2	17	2	1	2	1
8	0	0	0	0	0	0
1	1	1	1	1	1	1
1	0	0	0	0	0	0
1	1	1	1	1	1	1
1						

1	1	1	1	1	1	1
0	0	0	0	0	0	0
41249	5736	45782	12221	10285	1532	15150
1						
0						
41249						
37.8%						
	100.0%	100.0%	100.0%	100.0%	100.0%	100.0%
37.8%						
	100.0%	100.0%	100.0%	100.0%	100.0%	100.0%
37.8%						

Tree of Hope	Trigeminal N	Tuberous Scl	Turner Syndr	Twins And M	UK Chronic L	UK Gout Soci
1	3	8	21	6	1	3
0	0	4	11	5	0	0
1	1	1	1	1	1	1
0	0	1	1	1	0	0
1	1	1	1	1	1	1
	1	1	1	1		1
			1			
1	1	1	1	1	1	1
1	3	4	10	1	1	3
	1	1	1	1		1
	3	4	10	1		3
			1			
			10			

				16.7%		
			47.6%	16.7%		
100.0%	100.0%				100.0%	100.0%
				16.7%		
			47.6%	16.7%		
	100.0%					100.0%
			47.6%			
152	4208	91245	39241	42922	4056	38407
0	0	120378	0	0	0	0
1	1	8	20	6	1	3
0	0	4	0	0	0	0
1	1	1	1	1	1	1
0	0	1	0	0	0	0
1	1	1	1	1	1	1
		1				

1	1		1	1	1	1
		1				
0	0	0	0	0	0	0
152	4208		39241	42922	4056	38407
		29133				
		1				
		0				
		29133				
		24.2%				
100.0%	100.0%		100.0%	100.0%	100.0%	100.0%
		24.2%				
100.0%	100.0%		100.0%	100.0%	100.0%	100.0%
		24.2%				

UK Primary Ir	UK Sepsis Trı	UK Thalassae	Urology Four	Visionary	Waldenstron	Walk the Wa
15	1	7	3	6	6	1
1	0	12	5	2	3	0
1	1	1	1	1	1	1
1	0	1	1	1	1	0
1	1	1	1	1	1	1
1		1	1	1	1	
1		1				
1	1			1	1	1
		1	1			
14	1			4	3	1
		5	2			
1				1	1	
		1	1			
14				4	3	
		5	2			
1						
		1				
14						
		5				

		41.7%	40.0%			
	100.0%					100.0%
		41.7%	40.0%			
		41.7%				
107005	0	43405	30621	95543	25021	406
0	0	132930	0	15273	0	0
15	0	7	3	6	6	1
0	0	12	0	2	0	0
1	0	1	1	1	1	1
0	0	1	0	1	0	0
1		1	1	1	1	1
1		1				

	FALSE					
1			1	1	1	1
		1				
0		0	0	0	0	0
107005			30621	80270	25021	406
		89525				
1						
		1				
0		0				
107005						
		89525				
100.0%			100.0%		100.0%	100.0%
100.0%			100.0%		100.0%	100.0%
100.0%						

Waverley Ca	Wellbeing of	WellChild	Willow Foun	World Cance	World Child (	York Air Amb
18	1	2	1	1	15	1
0	1	1	0	0	0	0
1	1	1	1	1	1	1
0	1	1	0	0	0	0
1	1	1	1	1	1	1
1		1			1	
1					1	
	1					
1		1	1	1	1	1
18		1	1	1	15	1
1		1			1	
18		1			15	
1					1	
18					15	

	0.0%					
	0.0%					
	0.0%					
100.0%			100.0%	100.0%	100.0%	100.0%
100.0%					100.0%	
100.0%					100.0%	
103557	4114	262000	258	5000	342276	0
0	0	0	0	0	0	0
18	1	2	1	1	15	0
0	0	0	0	0	0	0
1	1	1	1	1	1	0
0	0	0	0	0	0	0
1	1	1	1	1	1	
1		1			1	

						FALSE
1	1	1	1	1	1	
0	0	0	0	0	0	
103557	4114	262000	258	5000	342276	
1		1			1	
0		0			0	
103557		262000			342276	
100.0%	100.0%	100.0%	100.0%	100.0%	100.0%	
100.0%	100.0%	100.0%	100.0%	100.0%	100.0%	
100.0%		100.0%			100.0%	

Young Epilepsy

14
7
1
1

1
1
1

1

7

1

7

1

7

147292
0
13
0
1
0

1
1

1

0
147292

1

0
147292

100.0%

100.0%

100.0%

Web Supplement 6. Recipients - Absolute and relative differences in the number and value of payments (in

NUMBER OF PAYMENTS - ABSOLUTE DIFFERENCES

Threshold 0: At least 1 one payment in at least one dataset
Total
Number of patient organisations with exact match in both datasets
Number of patient organisations with more paymens in industry data
Number of patient organisations with more payments in patient organisation data

Highest absolute difference between patient organisation and industry data - number of payments higher in i
Highest absolute difference between patient organisation and industry data - number of payments higher in p

Threshold 1: More than 1 one payment in at least one datase
Total
Number of patient organisations with exact match in both datasets
Number of patient organisations with more paymens in industry data
Number of patient organisations with more payments in patient organisation data

Highest absolute difference between patient organisation and industry data - number of payments higher in i
Highest absolute difference between patient organisation and industry data - number of payments higher in p

Threshold 2: More than 10 payments in at least one dataset
Total
Number of patient organisations with exact match in both datasets
Number of patient organisations with more paymens in industry data
Number of patient organisations with more payments in patient organisation data

Highest absolute difference between patient organisation and industry data - number of payments higher in i
Highest absolute difference between patient organisation and industry data - number of payments higher in p

Threshold 3: More than 100 payments in at least one dataset
Total
Number of patient organisations with exact match in both datasets
Number of patient organisations with more paymens in industry data
Number of patient organisations with more payments in patient organisation data

Highest absolute difference between patient organisation and industry data - number of payments higher in i
Highest absolute difference between patient organisation and industry data - number of payments higher in p

NUMBER OF PAYMENTS - RELATIVE DIFFERENCES

Threshold 0: At least 1 one payment in at least one dataset
Total
Relative difference <10%

Relative difference <20%
Relative difference <50%
Relative difference = 100%

Threshold 1: More than 1 one payment in at least one datase
Total
Relative difference <10%
Relative difference <20%
Relative difference <50%
Relative difference = 100%

Threshold 2: More than 10 payments in at least one dataset
Total
Relative difference <10%
Relative difference <20%
Relative difference <50%
Relative difference = 100%

Threshold 3: More than 100 payments in at least one dataset
Total
Relative difference <10%
Relative difference <20%
Relative difference <50%
Relative difference = 100%

VALUE OF PAYMENTS - ABSOLUTE DIFFERENCES

Threshold 1: At least one payment with value >£0 in at least one dataset
Total
Number of patient organisations with exact match in both datasets
Number of patient organisations with higher payment value in industry data
Number of patient organisations with higher payment value in patient organisation data

Highest absolute difference between patient organisation and industry data - number of payments higher in i
Highest absolute difference between patient organisation and industry data - number of payments higher in [illegible]

Threshold 2: Payments with the value of more than £10,000 in at least one dataset
Total
Number of patient organisations with exact match in both datasets
Number of patient organisations with higher payment value in industry data
Number of patient organisations with higher payment value in patient organisation data

Highest absolute difference between patient organisation and industry data - number of payments higher in i
Highest absolute difference between patient organisation and industry data - number of payments higher in [illegible]

Threshold 3: Payments with the value of more than £100,000 in at least one dataset

Total
Number of patient organisations with exact match in both datasets
Number of patient organisations with higher payment value in industry data
Number of patient organisations with higher payment value in patient organisation data

Highest absolute difference between patient organisation and industry data - number of payments higher in i
Highest absolute difference between patient organisation and industry data - number of payments higher in p

VALUE OF PAYMENTS - RELATIVE DIFFERENCES

Threshold 1: At least one payment with value >£0 in at least one dataset
Total
Relative difference <10%
Relative difference <20%
Relative difference <50%
Relative difference = 100%

Threshold 2: Payments with the value of more than £10,000 in at least one dataset
Total
Relative difference <10%
Relative difference <20%
Relative difference <50%
Relative difference = 100%

Threshold 3: Payments with the value of more than £100,000 in at least one dataset
Total
Relative difference <10%
Relative difference <20%
Relative difference <50%
Relative difference = 100%

**All years**		**2012**		**2013**	
Number of pat	% of all patient	Number of pa	% of all patient	Number of pa	% of all patient
425	100.0%	219	100.0%	225	100.0%
33	7.8%	17	7.8%	18	8.0%
335	78.8%	160	73.1%	157	69.8%
57	13.4%	42	19.2%	50	22.2%
Number of payments		Number of payments		Number of payments	
199		36		35	
33		9		17	
Number of pat	% of all patient	Number of pa	% of all patient	Number of pa	% of all patient
299	100.0%	140	100.0%	137	100.0%
13	4.3%	6	4.3%	9	6.6%
229	76.6%	105	75.0%	95	69.3%
57	19.1%	29	20.7%	33	24.1%
Number of payments		Number of payments		Number of payments	
199		36		35	
33		9		17	
Number of pat	% of all patient	Number of pa	% of all patient	Number of pa	% of all patient
115	100.0%	20	100.0%	13	100.0%
2	1.7%	1	5.0%	0	0.0%
95	82.6%	16	80.0%	9	69.2%
18	15.7%	3	15.0%	4	30.8%
Number of payments		Number of payments		Number of payments	
199		36		35	
33		9		17	
Number of pat	% of all patient	Number of pa	% of all patient	Number of pa	% of all patient
3	100.0%	0	N/A	0	N/A
0	0.0%	N/A	N/A	N/A	N/A
3	100.0%	N/A	N/A	N/A	N/A
0	0.0%	N/A	N/A	N/A	N/A
Number of payments		Number of payments		Number of payments	
199		0		0	
0		0		0	
Number of pat	% of all patient	Number of pa	% of all patient	Number of pa	% of all patient
425	100.0%	219	100.0%	225	100.0%
35	8.2%	17	7.8%	18	8.0%

42	9.9%	18	8.2%	19	8.4%
81	19.1%	30	13.7%	34	15.1%
225	52.9%	138	63.0%	150	66.7%
Number of pat	% of all patient	Number of pa	% of all patient	Number of pa	% of all patient
299	100.0%	140	100.0%	137	100.0%
15	5.0%	6	4.3%	9	6.6%
22	7.4%	7	5.0%	10	7.3%
61	20.4%	19	13.6%	25	18.2%
119	39.8%	70	50.0%	71	51.8%
Number of pat	% of all patient	Number of pa	% of all patient	Number of pa	% of all patient
115	100.0%	20	100.0%	13	100.0%
4	3.5%	1	5.0%	0	0.0%
7	6.1%	2	10.0%	0	0.0%
31	27.0%	4	20.0%	1	7.7%
33	28.7%	7	35.0%	5	38.5%
Number of pat	% of all patient	Number of pa	% of all patient	Number of pa	% of all patient
3	100.0%	0	N/A	0	N/A
0	0.0%	N/A	N/A	N/A	N/A
0	0.0%	N/A	N/A	N/A	N/A
0	0.0%	N/A	N/A	N/A	N/A
1	33.3%	N/A	N/A	N/A	N/A
Number of pat	% of all patient	Number of pa	% of all patient	Number of pa	% of all patient
416	100.0%	206	100.0%	204	100.0%
8	1.9%	4	1.9%	4	2.0%
356	85.6%	168	81.6%	164	80.4%
52	12.5%	34	16.5%	36	17.6%
Value of payments		Value of payments		Value of payments	
6,718,576.6		660,846.1		369,657.9	
6,493,237.1		283,684.7		829,991.7	
Number of pat	% of all patient	Number of pa	% of all patient	Number of pa	% of all patient
104	100.0%	29	100.0%	20	100.0%
0	0.0%	0	0.0%	0	0.0%
80	76.9%	19	65.5%	12	60.0%
24	23.1%	10	34.5%	8	40.0%
Value of payments		Value of payments		Value of payments	
6,718,576.6		660,846.1		369,657.9	
6,493,237.1		283,684.7		829,991.7	
Number of pat	% of all patient	Number of pa	% of all patient	Number of pa	% of all patient

13	100.0%		0 N/A		0 N/A
0	0.0%	N/A	N/A	N/A	N/A
6	46.2%	N/A	N/A	N/A	N/A
7	53.8%	N/A	N/A	N/A	N/A

Value of payments	Value of payments	Value of payments
6,718,576.6	-	-
6,493,237.1	-	-

Number of pat	% of all patient	Number of pa	% of all patient	Number of pa	% of all patient
416	100.0%	206	100.0%	204	100.0%
21	5.0%	6	2.9%	8	3.9%
34	8.2%	11	5.3%	13	6.4%
73	17.5%	20	9.7%	21	10.3%
295	70.9%	158	76.7%	164	80.4%

Number of pat	% of all patient	Number of pa	% of all patient	Number of pa	% of all patient
104	100.0%	29	100.0%	20	100.0%
4	3.8%	0	0.0%	0	0.0%
8	7.7%	2	6.9%	0	0.0%
23	22.1%	4	13.8%	2	10.0%
54	51.9%	17	58.6%	11	55.0%

Number of pat	% of all patient	Number of pa	% of all patient	Number of pa	% of all patient
13	100.0%	0	N/A	0	N/A
0	0.0%	N/A	N/A	N/A	N/A
0	0.0%	N/A	N/A	N/A	N/A
3	23.1%	N/A	N/A	N/A	N/A
4	30.8%	N/A	N/A	N/A	N/A

2014		2015		2016
Number of pati	% of all patient	Number of pati	% of all patient organisatio	Number of pati
247	100.0%	267	100.0%	260
29	11.7%	31	11.6%	21
173	70.0%	186	69.7%	208
45	18.2%	50	18.7%	31
Number of payments		Number of payments		Number of payr
36		44		55
15		10		11
Number of pati	% of all patient	Number of pati	% of all patient organisatio	Number of pati
146	100.0%	149	100.0%	166
6	4.1%	8	5.4%	7
110	75.3%	107	71.8%	138
30	20.5%	34	22.8%	21
Number of payments		Number of payments		Number of payr
36		44		55
15		10		11
Number of pati	% of all patient	Number of pati	% of all patient organisatio	Number of pati
15	100.0%	22	100.0%	29
0	0.0%	0	0.0%	0
11	73.3%	19	86.4%	26
4	26.7%	3	13.6%	3
Number of payments		Number of payments		Number of payr
36		44		55
15		10		11
Number of pati	% of all patient	Number of pati	% of all patient organisatio	Number of pati
0	N/A	0	N/A	0
N/A	N/A	N/A	N/A	N/A
N/A	N/A	N/A	N/A	N/A
N/A	N/A	N/A	N/A	N/A
Number of payments		Number of payments		Number of payr
0		0		0
0		0		0

Number of pati	% of all patient	Number of pati	% of all patient organisatio	Number of pati
247	100.0%	267	100.0%	260
29	11.7%	31	11.6%	21

32	13.0%	33	12.4%	24
48	19.4%	49	18.4%	45
153	61.9%	164	61.4%	159

Number of pati	% of all patient	Number of pati	% of all patient organisatic	Number of pati
146	100.0%	149	100.0%	166
6	4.1%	8	5.4%	7
9	6.2%	10	6.7%	10
25	17.1%	26	17.4%	31
75	51.4%	69	46.3%	79

Number of pati	% of all patient	Number of pati	% of all patient organisatic	Number of pati
15	100.0%	22	100.0%	29
0	0.0%	0	0.0%	0
1	6.7%	1	4.5%	1
3	20.0%	4	18.2%	6
5	33.3%	7	31.8%	13

Number of pati	% of all patient	Number of pati	% of all patient organisatic	Number of pati
0	N/A	0	N/A	0
N/A	N/A	N/A	N/A	N/A
N/A	N/A	N/A	N/A	N/A
N/A	N/A	N/A	N/A	N/A
N/A	N/A	N/A	N/A	N/A

Number of pati	% of all patient	Number of pati	% of all patient organisatic	Number of pati
234	100.0%	249	100.0%	249
11	4.7%	15	6.0%	8
196	83.8%	198	79.5%	211
27	11.5%	36	14.5%	30

Value of payments	Value of payments	Value of payme
538,969.2	1,473,965.2	6,006,677.7
1,824,997.0	815,956.8	6,493,237.1

Number of pati	% of all patient	Number of pati	% of all patient organisatic	Number of pati
31	100.0%	34	100.0%	36
0	0.0%	0	0.0%	0
24	77.4%	25	73.5%	25
7	22.6%	9	26.5%	11

Value of payments	Value of payments	Value of payme
538,969.2	1,473,965.2	6,006,677.7
1,824,997.0	815,956.8	6,493,237.1

Number of pati	% of all patient	Number of pati	% of all patient organisatic	Number of pati

1	100.0%	1	100.0%	2
0	0.0%	0	0.0%	0
0	0.0%	1	100.0%	1
1	100.0%	0	0.0%	1

Value of payments	Value of payments	Value of payme
-	1,473,965.24	6,006,677.66
1,824,996.99	-	6,493,237.05

Number of pati	% of all patient	Number of pati	% of all patient organisatic	Number of pati
234	100.0%	249	100.0%	249
16	6.8%	20	8.0%	18
19	8.1%	25	10.0%	26
28	12.0%	39	15.7%	36
185	79.1%	189	75.9%	197

Number of pati	% of all patient	Number of pati	% of all patient organisatic	Number of pati
31	100.0%	34	100.0%	36
1	3.2%	0	0.0%	2
2	6.5%	0	0.0%	4
5	16.1%	5	14.7%	8
20	64.5%	21	61.8%	20

Number of pati	% of all patient	Number of pati	% of all patient organisatic	Number of pati
1	100.0%	1	100.0%	2
0	0.0%	0	0.0%	0
0	0.0%	0	0.0%	0
0	0.0%	0	0.0%	0
0	0.0%	1	100.0%	0

% of all patient organisationsa given threshold
100.0%
8.1%
80.0%
11.9%

nents

% of all patient organisationsa given threshold
100.0%
4.2%
83.1%
12.7%

nents

% of all patient organisationsa given threshold
100.0%
0.0%
89.7%
10.3%

nents

% of all patient organisationsa given threshold
N/A
N/A
N/A
N/A

nents

% of all patient organisationsa given threshold
100.0%
8.1%

9.2%
17.3%
61.2%

% of all patient organisationsa given threshold
100.0%
4.2%
6.0%
18.7%
47.6%

% of all patient organisationsa given threshold
100.0%
0.0%
3.4%
20.7%
44.8%

% of all patient organisationsa given threshold
N/A
N/A
N/A
N/A
N/A

% of all patient organisationsa given threshold
100.0%
3.2%
84.7%
12.0%

nts

% of all patient organisationsa given threshold
100.0%
0.0%
69.4%
30.6%

nts

% of all patient organisationsa given threshold

100.0%
0.0%
50.0%
50.0%

nts

% of all patient organisationsa given threshold
100.0%
7.2%
10.4%
14.5%
79.1%

% of all patient organisationsa given threshold
100.0%
5.6%
11.1%
22.2%
55.6%

% of all patient organisationsa given threshold
100.0%
0.0%
0.0%
0.0%
0.0%

Web Supplement 7. Donors - summary of payments missing from

	Year
	2012
	2013
	2014
	2015
	2016
**Total missing - calculated annually**	
**Total missing - calculated for all years**	

**industry data**
**Number of companies missing from industry data**
31
30
27
23
0
**N/A**
**24**

Companies missing from industry dataset as a share of companies in industry

103.3%
78.9%
60.0%
45.1%
0.0%
**N/A**
**38.1%**

Number of payments missing from industry data	Number of payments missing from industry data
124	16.1%
131	18.6%
74	9.0%
52	5.6%
54	5.0%
**435**	**10.1%**
**128**	**3.0%**

Value of payments missing from industry data	Value of payments missing from industry datas as a
1,731,257.8	22.2%
2,666,247.8	43.1%
218,768.4	2.5%
1,367,440.0	12.0%
474,975.0	2.4%
**6,458,689.1**	**11.9%**
**1,610,321.1**	**3.0%**

share of payments in industry datas

Web Supplement 8. Donors - payments missing from industry data (yearly breakdown)

Year	Drug company name	Number of payments	Value of payments (2016 £)
	Alcon	3	18937.5
	Alexion	1	0.0
	ApoPharma	1	23145.8
	Baxter	7	170239.7
	Bayer	8	205156.3
	BioMarin	1	0.0
	Biotest	1	22480.9
	Boehringer Ingelheim	2	9468.8
	Dermal	3	0.0
	Eisai	1	0.0
	Flynn Pharma	1	2104.2
	Fresenius Medical Care	1	7890.6
	Genzyme	9	116178.4
	Gilead	8	50500.0
	GSK	18	501114.7
2012	Ipsen	3	25250.0
	Janssen	12	50804.1
	LEO Pharma	2	0.0
	Norgine	1	0.0
	Novo Nordisk	3	0.0
	Octapharma	1	134882.3
	Pharma Mar	1	6312.5
	RB	1	83114.6
	Roche Products	13	237981.3
	Sanofi Pasteur MSD	1	20989.1
	Sigma Tau Rare Diseases	1	0.0
	Sobi	1	1052.1
	Takeda	6	23145.8
	Teva	5	15248.9
	UCB	7	5260.4
	Veriton	1	0.0
	Alcon	4	64796.3
	Alexion	2	5142.6
	Almirall	2	0.0
	Amdipharm	1	0.0
	ApoPharma	3	22627.3
	Bausch and Lomb	2	22627.3
	Baxter	3	135138.4
	Bayer	9	299046.4
	Britannia	1	0.0
	Dermal	3	0.0
	Gedeon Richter	1	0.0
	Genzyme	9	278174.8
	Gilead	10	116016.3
	GSK	16	406794.5
	Ipsen	2	20570.3
2013			

[illegible]	IQVIA	1	0.0
	Janssen	13	42110.4
	LEO Pharma	4	0.0
	Lundbeck	1	0.0
	Meda	1	0.0
	Merck	8	238799.2
	Norgine	2	0.0
	Orphan Europe	1	1028.5
	PTC	2	0.0
	RB	2	30855.4
	Roche Products	14	126301.4
	Sigma Tau Rare Diseases	2	514.3
	Takeda	9	4695.2
	Veriton	1	0.0
	Vifor Pharma Group	2	851009.3
	Actavis	1	0.0
	Alcon	1	0.0
	Alexion	5	15210.8
	Alliance	1	0.0
	Amdipharm	1	18253.0
	ApoPharma	1	30421.7
	Baxter	2	70948.4
	Bayer	4	27301.4
	Britannia	1	0.0
	Dermal	2	0.0
	Gedeon Richter	1	0.0
	Genzyme	13	225781.6
	GSK	12	2123196.4
2014	Ipsen	2	34873.4
	Meda	1	0.0
	Mitsubishi Tanabe Pharma	1	5070.3
	Norgine	2	16884.0
	Novo Nordisk	4	16144.8
	Orion Pharma	1	0.0
	PTC	2	0.0
	Rosemont Pharmaceuticals	1	0.0
	Sigma Tau Rare Diseases	2	28869.2
	Stirling Anglian	1	0.0
	Syner-Med	2	0.0
	Teva	6	7098.4
	Veriton	2	0.0
	Vifor Pharma Group	2	470654.9
	Actavis	2	24240.0
	Aegerion	1	0.0
	ALK-Abello	1	0.0
	Amdipharm	1	15680.3
	Bausch and Lomb	1	0.0
	Britannia	1	0.0

	Dermal	3	0.0
	Fresenius Medical Care	1	5050.0
	Genzyme	9	257424.8
	GSK	13	211609.1
	HRA Pharma	1	0.0
2015	Norgine	3	16816.5
	Octapharma	2	64864.2
	Orphan Europe	1	0.0
	Pharma Mar	1	0.0
	PTC	1	0.0
	RB	1	30300.0
	Sanofi Pasteur MSD	1	0.0
	Santen	1	0.0
	Sigma Tau Rare Diseases	2	11586.7
	Syner-Med	1	0.0
	Thea	1	0.0
	Vifor Pharma Group	3	729868.4
	Meda	1	0.0
	ALK-Abello	1	0.0
	Bausch and Lomb	1	0.0
	Genzyme	12	348335.0
	HRA Pharma	1	0.0
	BioMarin	4	5000.0
	Allergan	5	0.0
	Napp Pharmaceuticals	2	23000.0
	Amdipharm	1	500.0
	Alexion	4	27000.0
	Orphan Europe	1	0.0
	Ferring	6	0.0
2016	Britannia	1	3884.0
	Aegerion	1	0.0
	Baxalta	1	0.0
	Diurnal	1	0.0
	PTC	1	0.0
	Bio Products Laboratory	1	10000.0
	Octapharma	2	24766.0
	Sigma Tau Rare Diseases	1	12706.0
	Baxter	1	16784.0
	Fresenius Medical Care	1	3000.0
	Jazz	1	0.0
	Mylan	1	0.0
	Dermal	2	0.0

Web Supplement 9. Donors - payments missing from industry data (breakdown for all years)

Year	Drug company name	Number of payments	Value of payments (2016 £)
2012-2016	RB	4	144270.0
	Genzyme	52	1225894.6
	Sigma Tau Rare Diseases	8	53676.1
	Veriton	4	0.0
	Sanofi Pasteur MSD	2	20989.1
	Fresenius Medical Care	3	15940.6
	Dermal	13	0.0
	Pharma Mar	2	6312.5
	ApoPharma	5	76194.8
	Amdipharm	4	34433.3
	Orphan Europe	3	1028.5
	Gedeon Richter	2	0.0
	PTC	6	0.0
	IQVIA	1	0.0
	Britannia	4	3884.0
	Bausch and Lomb	4	22627.3
	Syner-Med	3	0.0
	Mitsubishi Tanabe Pharma	1	5070.3
	Alliance	1	0.0
	ALK-Abello	2	0.0
	Thea	1	0.0
	Diurnal	1	0.0
	Jazz	1	0.0
	Mylan	1	0.0

Web Supplement 10. Donors - summary of payments missing from patier

Year	Number of companies missing from patient orgaı
**2012**	5
**2013**	3
**2014**	7
**2015**	5
**2016**	4
**Total missing - calculate**	**N/A**
**Total missing - calculate**	**3**

ıt organisation data

Companies missing from patient organisation data as a share of companies in
8.9%
4.6%
10.8%
7.1%
6.2%
**N/A**
**3.6%**

Number of payments missing from patient orga	Number of payments missing from patient orga
14	4.8%
6	1.8%
17	4.9%
20	5.5%
10	3.0%
**67**	**4.0%**
**9**	**0.5%**

Value of payments missing from patient organ	Value of payments missing from patient organisatic
113,328.0	3.3%
68,397.8	1.5%
138,893.5	2.6%
52,758.7	1.1%
20,649.1	0.1%
**394,027.2**	**1.2%**
**92,208.2**	**0.3%**

›n data as a share of payments in patient organisation data

Web Supplement 11. Donors - payments missing from patient organisation data (yearly break

Year	Drug company name	Number of payments	Value of payments (2016 £)
2012	A. Menarini	2	26302.1
	Consilient Health	2	21041.7
	Daiichi Sankyo	2	16833.3
	Meda	4	30589.3
	Servier Laboratories	4	18561.6
2013	Consilient Health	1	10285.1
	HRA Pharma	3	46797.4
	Servier Laboratories	2	11315.4
2014	A. Menarini	1	7605.4
	Almirall	3	47559.2
	Chugai	2	811.2
	Consilient Health	1	10140.6
	Grünenthal	3	12939.4
	Lundbeck	5	50702.8
	Servier Laboratories	2	9134.9
2015	Almirall	3	12538.1
	Chugai	2	15409.9
	Flynn Pharma	5	13700.7
	Rosemont Pharmaceuticals	7	0.0
	Stirling Anglian	3	11110.0
2016	Almirall	3	2871.6
	Chugai	3	2612.5
	Flynn Pharma	3	5165.0
	Orion Pharma	1	10000.0

:down)

Web Supplement 12. Donors - payments missing from patient organisation data (all yea

Year	Drug company name	Number of payments	Value of payments (2016 £)
	A. Menarini	3	33907.5
2012-2016	Consilient Health	4	41467.4
	Daiichi Sankyo	2	16833.3

BMJ Publishing Group Limited (BMJ) disclaims all liability and responsibility arising from any reliance placed on this supplemental material which has been supplied by the author(s)

ars)

Web Supplement 13. Recipients - summary of payments missing from patient organisatio

Year	Number of patient organisations missing from pa
**2012**	116
**2013**	123
**2014**	131
**2015**	141
**2016**	143
**Total missing - calculated annually**	**N/A**
**Total missing - calculated for all years**	**225**

n data

Patient organisations missing from patient organisation data as a share of all
112.6%
120.6%
112.9%
111.9%
122.2%
**N/A**
**112.5%**

Number of payments missing from patient orga	Number of payments missing from patient orga
399	137.1%
369	113.2%
411	118.8%
462	128.0%
533	158.2%
**2174**	**130.9%**
**1472**	**88.6%**

Value of payments missing from patient organ	Value of payments missing from patient organisatio
3,659,293.41	105.8%
2,340,576.29	53.0%
4,427,502.34	83.5%
6,591,354.62	136.6%
4,871,241.58	32.4%
**21,889,968.23**	**66.3%**
**14,023,475.41**	**42.4%**

on data as share of the value of payments in patient organisation data

BMJ Publishing Group Limited (BMJ) disclaims all liability and responsibility arising from any reliance placed on this supplemental material which has been supplied by the author(s)

Web Suppler
Year

2012

2013

2014

2015

2016

nent 14. Recipients - payments missing from patient organisation data (yearly breakdown)

Patient organisation name
Action on Pain
Action on Pre-Eclampsia
African-Caribbean Leukaemia Trust
Age Related Diseases and Health Trust
Allergy Research Foundation
Alzheimer Scotland
Alzheimers Dementia Support
Anaphylaxis Campaign
Anticoagulation UK
Arrhythmia Alliance
Arthritis and Musculoskeletal Alliance
Atrial Fibrillation Association
Beating Bowel Cancer
Bipolar Scotland
Bipolar UK
Bladder and Bowel Foundation (taken over by Bladder and Bowel Community in 2017)
Bladder Health UK
Blood Pressure UK
Bluebell Foundation
Brain Charity
Breakthrough Breast Cancer (current name Breast Cancer Now, following merger with Breast Cancer Campai
Breast Cancer Campaign (current name Breast Cancer Now, following merger with Breakthrough Breast Canc
British Dupuytren's Society
British Heart Foundation
British Obesity Society
Brook
Cancer 52
Cancer Focus Northern Ireland
Cancer Research UK
Child Growth Foundation
Children with Cancer UK
Children's Heart Unit Fund
Children's HIV Association
Chinese National Healthy Living Centre
Cleft Lip and Palate Association
Coping with Cancer in Leicestershire and Rutland
Core Charity
Crohn's in Childhood Research Association
Cure Parkinson's Trust
Cystic Fibrosis Trust
Deaf Education through Listening and Talking
Diabetes Research and Wellness Foundation
Diabetes UK
Disabled Living
Epilepsy Scotland
European Parkinson's Disease Association

Family Planning Association
Forum Link
Gay Men's Health
GIST Support UK
Group B Strep Support UK
Haemophilia Society
Health and Social Care Alliance Scotland
Heartbeat
Hereditary Angioedema UK
HIV i-Base
Hughes Syndrome Foundation
ICUsteps
Independent Cancer Patients' Voice
International Alliance of Patients' Organizations
Karen Clifford Skcin cancer charity
Kidney Cancer UK
Leicestershire AIDS Support Services
Macmillan Cancer Support
Maggie's Centre
Malnutrition and Nutritional Care in the UK
Marie Curie Cancer Care
Medical Foundation for HIV and Sexual Health
Melanoma UK
Mental Health Foundation
Migraine Action
MPN Voice
Myeloma UK
Myrovlytis Trust
National Eczema Society
National Kidney Federation
National Obesity Forum
National Osteoporosis Society
OcuMel UK
Organisation for Sickle Cell Anaemia Research and Thalassaemia Support
Paget's Association
Pain Association Scotland
Pain Concern
Pain Relief Foundation
Pain UK
Pancreatic Cancer UK
Patients Association
Pelvic Pain Support Network
Positive East
Prader-Willi Syndrome Association UK
Prostate Cancer Research Centre
QUIT
Rarer Cancers Foundation
Raynaud's and Scleroderma Association (current name: Scleroderma and Raynauds UK, following the merger

Richmond Fellowship
Roy Castle Lung Cancer Foundation
Salamander Trust
SANE
SeeAbility
Service by Emergency Response Volunteers - Suffolk and Cambridgeshire
Shift.MS
Silver Star
Society for Mucopolysaccharide Diseases
Somerville Foundation
Spinal Injuries Scotland
Spinal Muscular Atrophy Support UK
Stroke Association
Tackle Prostate Cancer
Tenovus Cancer Care
Thames Valley Positive Support
Thrombosis UK
Trigeminal Neuralgia Association
UK Gout Society
UK Primary Immune-deficiency Patient Support
Waverley Care
World Child Cancer
21 and Co
Action on Pain
Action on Smoking and Health - Scotland
ADHD Foundation
African-Caribbean Leukaemia Trust
African's Getting Involved
Alpha-1 Awareness UK
Alpha-1 UK Support Group
Anaphylaxis Campaign
Anticoagulation UK
Arrhythmia Alliance
Arthritis and Musculoskeletal Alliance
Association for Glycogen Storage Disease
Association for Multiple Endocrine Neoplasia Disorders
Atrial Fibrillation Association
Barts and The London Charity
Beating Bowel Cancer
Bladder and Bowel Foundation (taken over by Bladder and Bowel Community in 2017)
Bladder Health UK
Bloodwise
Bowel Cancer UK
Breakthrough Breast Cancer (current name Breast Cancer Now, following merger with Breast Cancer Campai[illegible]
Breast Cancer Haven (The Haven)
British Dupuytren's Society
British Heart Foundation
British Liver Trust

British Polio Fellowship
Brittle Bone Society
Brook
Cancer 52
Cancer Research UK
Cardiac Risk in the Young
Cardiomyopathy UK
Cleft Lip and Palate Association
Coeliac UK
Community Cancer Centre
Contact a Family
Crohn's in Childhood Research Association
Cure Parkinson's Trust
Cystic Fibrosis Trust
David Lewis Centre
Dementia UK
Diabetes Research and Wellness Foundation
Douglas Bader Foundation
Epilepsy Connections
Epilepsy Scotland
Family Planning Association
Forum Link
Gauchers Association
Gay Men's Health
Haemophilia Scotland
Haemophilia Society
Heart UK
Hereditary Angioedema UK
HIV i-Base
ICUsteps
Independent Cancer Patients' Voice
International Alliance of Patients' Organizations
Juvenile Diabetes Research Foundation
Kidney Cancer Support Network
Kidney Cancer UK
Leicestershire AIDS Support Services
Life Education Wessex
Liver4Life
Lupus Europe
Macmillan Cancer Support
Maggie's Centre
Marie Curie Cancer Care
Melanoma UK
Men’s Health Forum
Mesothelioma UK
MPN Voice
Myeloma UK
National AIDS Manual

National Eczema Society
National Kidney Federation
National Obesity Forum
National Osteoporosis Society
Northern Ireland Chest, Heart and Stroke
OcuMel UK
Oliver King Foundation
Orchid
Organisation for Sickle Cell Anaemia Research and Thalassaemia Support
Ovacome
Ovarian Cancer Action
Pain Association Scotland
Pain Concern
Pain UK
Pancreatic Cancer Action
Pancreatic Cancer UK
Parkinson's UK
Patients Association
PBC Foundation UK
Polycystic Kidney Disease Charity
Positive East
Prostate Cancer Support Organisation
Prostate Cancer UK (merged with Prostate Action in 2012, retaining its original name)
Pumping Marvellous Foundation
QUIT
Rarer Cancers Foundation
Restless Leg Syndrome UK
Ronald McDonald House Charities
Roy Castle Lung Cancer Foundation
Royal Free Charity
SANE
Sarcoma UK
Scoliosis Association UK
Scotland Patients Association
Scottish Epilepsy Initiative
Self Help Services
Shift.MS
Silver Star
South Asian Health Foundation
SUDEP Action
Target Ovarian Cancer
Tenovus Cancer Care
Thrombosis UK
Transplant Links
Trigeminal Neuralgia Association
UK Primary Immune-deficiency Patient Support
UK Sepsis Trust
Waverley Care

World Child Cancer
AADC Research Trust
Action Duchenne
Action for Pulmonary Fibrosis
Action on Pain
Action on Smoking and Health - Wales
Actionplus Foundation
Addaction
Addenbrooks Charitable Trust
African Health Policy Network
African-Caribbean Leukaemia Trust
Alcohol Concern
Alpha-1 UK Support Group
Anaphylaxis Campaign
Anticoagulation UK
Arrhythmia Alliance
Arthritis and Musculoskeletal Alliance
Arthritis Research UK
Association for Glycogen Storage Disease
Asthma and Allergy Foundation
Asthma Relief
Atrial Fibrillation Association
Basil Skyers Myeloma Foundation
Beating Bowel Cancer
Behcets Syndrome Society
Bladder and Bowel Foundation (taken over by Bladder and Bowel Community in 2017)
Bloodwise
Bone Cancer Research Trust
Brain Tumor Charity
Breast Cancer Campaign (current name Breast Cancer Now, following merger with Breakthrough Breast Canc
Breast Cancer Haven (The Haven)
British Dupuytren's Society
British Heart Foundation
British Liver Trust
British Pregnancy Advisory Service
British Skin Foundation
Brook
Building for the Future
Cancer 52
Cancer Focus Northern Ireland
Cancer Research UK
Catholics for AIDS Prevention and Support
Child Growth Foundation
Children with Cancer in Malawi
Chronic Lymphocytic Leukaemia Support Association
Contact a Family
Core Charity
Crohn's in Childhood Research Association

Cure Parkinson's Trust
Cystic Fibrosis Trust
Diabetes Research and Wellness Foundation
Disabled Living
Edinburgh and Lothian Prostate Cancer Support Group
Epilepsy Connections
Epilepsy Scotland
European Parkinson’s Disease Association
Family Planning Association
Food Chain
FORCE
Forum Link
Freshwinds
Gay Men's Health
Haemophilia Society
Heart UK
Hepatitis B Positive Trust
HIV i-Base
Independent Cancer Patients' Voice
Juvenile Diabetes Research Foundation
Karen Clifford Skcin cancer charity
Kidney Cancer Support Network
Kidney Cancer UK
Leukaemia CARE
Liver4Life
Make A Wish Foundation
Melanoma UK
Mental Health Foundation
Mesothelioma UK
Migraine Action
MPN Voice
Multi Organ Transplant Support
National AIDS Manual
National Association for Pre-Menstrual Syndrome
National Eczema Society
National Kidney Federation
National Osteoporosis Society
Niemann-Pick UK
North of England Bone Marrow and Thalassaemia Association
Northern Ireland Rare Disease Partnership
Orchid
Ovacome
Ovarian Cancer Action
Paget's Association
Pain Concern
Pain UK
Pancreatic Cancer UK
Parkinson’s UK

Patients Association
PBC Foundation UK
Polycystic Kidney Disease Charity
Positive East
Prostate Cancer Support Organisation
Prostate Cancer UK (merged with Prostate Action in 2012, retaining its original name)
Prostate Cymru
Rainbow Centre for Conductive Education
Rapid Effective Assistance For Children With Potentially Terminal Illness
Rarer Cancers Foundation
Raynaud's and Scleroderma Association (current name: Scleroderma and Raynauds UK, following the merger
Release
Restless Leg Syndrome UK
Revive Multiple Sclerosis Support
Roy Castle Lung Cancer Foundation
Royal Marsden Cancer Charity
Royal National Institute of Blind People
SANE
Sarcoma UK
Scleroderma Society (current name: Scleroderma and Raynauds UK, following the merger of Raynaud's and S
Scottish Drugs Forum
Scottish Epilepsy Initiative
Silver Star
Skin Care Cymru
Sophia Forum
St Thomas Lupus Trust
Stroke Association
SUDEP Action
Tackle Prostate Cancer
Transplant Links
UK Chronic Lymphocytic Leukaemia Forum
UK Gout Society
UK Primary Immune-deficiency Patient Support
Walk the Walk Worldwide
Waverley Care
World Child Cancer
Action Duchenne
Action on Pain
Addaction
ADHD Foundation
African Health Policy Network
Age UK
aHUS UK
Alcohol Concern
Alpha-1 UK Support Group
Anaphylaxis Campaign
Angelman Syndrome Support Education Research Trust
Anticoagulation UK

Anxiety UK
Aplastic Anaemia Trust
Arrhythmia Alliance
Arthritis and Musculoskeletal Alliance
Association for Glycogen Storage Disease
Atrial Fibrillation Association
BackCare
Basil Skyers Myeloma Foundation
Beat Parkinson's
Beating Bowel Cancer
Bladder and Bowel Foundation (taken over by Bladder and Bowel Community in 2017)
Bloodwise
Bluebell Foundation
Body and Soul
Bone Cancer Research Trust
Breast Cancer Campaign (current name Breast Cancer Now, following merger with Breakthrough Breast Canc
Breast Cancer Haven (The Haven)
Breast Cancer Now (created after the merger of Breast Cancer Campaign and Breakthrough Breast Cancer in
British Dupuytren's Society
British Heart Foundation
British Liver Trust
British Skin Foundation
Brook
Cambridge Rare Disease Network
Cancer 52
Cancer Focus Northern Ireland
Cancer Research UK
Candlelighters
Children with Cancer UK
Children's Liver Disease Foundation
Chronic Lymphocytic Leukaemia Support Association
CLIC Sargent Cancer Care For Children
Contact a Family
Cure Parkinson's Trust
Cystic Fibrosis Trust
Delete Blood Cancer
Diabetes UK
Diabetics with Eating Disorders
Diamond Blackfan Anaemia UK
Douglas Bader Foundation
Encephalitis Society
Food Chain
Forum Link
Freshwinds
Friends of FOP
Gauchers Association
Gay Men's Health
Haemophilia Scotland

Haemophilia Society
Haemophilia Wales
Hidradenitis Suppurativa Trust
HIV i-Base
Independent Cancer Patients' Voice
Input Diabetes
International Chronic Myeloid Leukemia Foundation
Karen Clifford Skcin cancer charity
Kidney Cancer Support Network
Kidney Cancer UK
Kidney Care UK
LATCH Welsh Children's Cancer Charity
Leeds Teaching Hospital Charitable Foundation
Little Hearts Matter
Liver4Life
Lullaby Trust
Lupus Europe
Lupus UK
Macular Society
Maggie's Centre
MAMA Academy
Melanoma UK
Mental Health Foundation
Mesothelioma UK
Movember Foundation
Multiple Births Foundation
Myasthenia Gravis Association
NARA - The Breathing Charity
National AIDS Manual
National Eczema Society
National Kidney Federation
National Osteoporosis Society
NMO Research Foundation
North of England Bone Marrow and Thalassaemia Association
Older People's Advocacy Alliance
Ovacome
Pancreatic Cancer UK
Patients Association
Patients On Intravenous and Nasogastric Nutrition Therapy
Pelvic Pain Support Network
Portsmouth Down Syndrome Association
Positive Action
Progressive Supranuclear Palsy Association
Prostate Cancer UK (merged with Prostate Action in 2012, retaining its original name)
QUIT
Rainbow Trust Children's Charity
Rapid Effective Assistance For Children With Potentially Terminal Illness
Rarer Cancers Foundation

Raynaud's and Scleroderma Association (current name: Scleroderma and Raynauds UK, following the merger
Restless Leg Syndrome UK
Rethink Mental Illness
River House Trust
Roy Castle Lung Cancer Foundation
Royal Free Charity
Royal National Institute of Blind People
Salamander Trust
SANE
Scleroderma Society (current name: Scleroderma and Raynauds UK, following the merger of Raynaud's and S
Scottish Kidney Federation
Sense
Silver Star
Skin Care Cymru
Sophia Forum
Spectra
Spinal Injuries Association
St Thomas Lupus Trust
Steps
Stroke Association
Tackle Prostate Cancer
TB Alert
Team Margot
Teenage Cancer Trust
Tenovus Cancer Care
Thrombosis UK
Transverse Myelitis Society
Tree of Hope
UK Primary Immune-deficiency Patient Support
UK Thalassaemia Society
Waverley Care
World Child Cancer
York Air Ambulance
Action Bladder Cancer UK
Action Duchenne
Action on Smoking and Health - Wales
Actionplus Foundation
Addaction
ADHD Foundation
African-Caribbean Leukaemia Trust
ALD Life
Allergy UK
Alpha-1 UK Support Group
Ankylosing Spondylitis International Federation
Anthony Nolan
Anticoagulation UK
Arrhythmia Alliance
Arthritis and Musculoskeletal Alliance

Asyabi UK
Atrial Fibrillation Association
Back on Track
BackCare
Basil Skyers Myeloma Foundation
Beating Bowel Cancer
Birdshot Uveitis Society
Bladder and Bowel Foundation (taken over by Bladder and Bowel Community in 2017)
Body and Soul
Brain Tumour Research
Breast Cancer Haven (The Haven)
British Dupuytren's Society
British Liver Trust
British Skin Foundation
Brook
Cambridge Rare Disease Network
Cancer 52
Cancer Focus Northern Ireland
Cancer Research UK
Cardiac Risk in the Young
Cardiomyopathy UK
Charlie Waller Memorial Trust
Child Growth Foundation
Children's Liver Disease Foundation
Chronic Lymphocytic Leukaemia Support Association
CLIC Sargent Cancer Care For Children
Coppafeel!
Crohn's in Childhood Research Association
Cure and Action for Tay-Sachs
Cure Parkinson's Trust
Cystic Fibrosis Trust
Delete Blood Cancer
Diabetes UK
Diabetics with Eating Disorders
Different Strokes
Disabled Living
Encephalitis Society
Epilepsy Action
Epilepsy Scotland
Family Planning Association
Foundation for Liver Research
GIST Support UK
Guillain Barre and associated inflammatory neuropathies
Haemophilia Scotland
Haemophilia Society
Haemophilia Wales
Hafal
Headway

Heart UK
HIV i-Base
HIV Scotland
Imperial College Healthcare Charity
Independent Cancer Patients' Voice
Juvenile Diabetes Research Foundation
KAITY
Karen Clifford Skcin cancer charity
Kidney Cancer Support Network
Kidney Cancer UK
Kidney Care UK
Liver4Life
Lupus Europe
Macmillan Cancer Support
Make A Wish Foundation
Marie Curie Cancer Care
Martin Fisher Foundation
MDS UK Support Group
Melanoma Focus
Melanoma Fund
Melanoma UK
Mesothelioma UK
Migraine Action
Motor Neurone Disease Association
MPN Voice
Multiple Sclerosis UK
Muscular Dystrophy UK
National Attention Deficit Disorder Information and Support Service
National Cancer Research Institute
National Childbirth Trust
National Eczema Society
National Kidney Federation
National Osteoporosis Society
National Rheumatoid Arthritis Society
NMO Research Foundation
Northern Ireland Rare Disease Partnership
Olive Tree Cancer Support Centre - Crawley
Ovacome
Ovarian Cancer Action
Paget's Association
Pain Concern
Pancreatic Cancer Action
Parkinson’s UK
Patients Association
Patients On Intravenous and Nasogastric Nutrition Therapy
Paula Carr Diabetes Trust
Pelican Cancer Foundation
Pink Ribbon Foundation – Breast Cancer Charity

Polycystic Kidney Disease Charity
Progressive Supranuclear Palsy Association
QUIT
Rarer Cancers Foundation
Restless Leg Syndrome UK
Roy Castle Lung Cancer Foundation
Royal National Institute of Blind People
Samson Centre for MS
SANE
Sarcoma UK
Save Babies Through Screening Foundation UK
Scleroderma and Raynauds UK (established following the merger of Raynaud's and Scleroderma Association a
Scottish Network Children with Arthrities
Sexual Advice Association
Short Bowel Survivor and Friends
Skin Care Cymru
Somerville Foundation
Sussex Beacon
Tackle Prostate Cancer
Teenage Cancer Trust
Tenovus Cancer Care
Thames Valley Positive Support
Thrombosis UK
Tuberous Sclerosis Association
Visionary
Waldenstroms Macroglobulinaemia UK
Waverley Care
WellChild
Willow Foundation
World Cancer Research Fund
World Child Cancer
Young Epilepsy

Number of payments	Value of payments (2016 £)
3	3488.9
1	10520.8
1	15781.3
2	39258.9
2	21041.7
2	563.6
1	1057.3
1	15860.2
12	20260.5
6	7381.0
5	37832.9
36	227017.3
5	36010.8
3	3193.8
3	4145.2
13	415672.2
7	6165.9
1	10520.8
1	473.4
1	981.5
2	5298.0
1	6312.5
2	599.7
1	117.8
1	420.8
4	33929.7
5	33140.6
3	5681.3
10	660846.1
1	1578.1
1	473.4
1	736.5
1	7890.6
1	252.5
1	58.9
1	4187.3
1	8416.7
3	12625.0
1	254.6
2	12650.6
1	526.0
1	2104.2
29	315722.5
1	5260.4
1	37.6
1	21362.0

3	7680.2
2	747.0
3	9153.1
4	12414.6
1	153.6
9	50719.9
1	37.6
1	757.5
3	24923.9
4	16970.1
1	2104.2
1	2099.5
2	4103.1
3	122044.1
1	10520.8
8	24125.3
2	925.8
5	19768.6
1	157.8
1	883.8
1	241.4
1	5260.4
2	21041.7
2	79095.6
2	25776.0
2	18170.9
7	215976.6
1	1420.3
9	20541.9
8	144188.0
2	1157.3
3	13082.8
1	1052.1
1	2104.2
1	2104.2
4	3000.9
4	6689.5
1	631.3
4	16916.6
1	8416.7
10	66089.5
3	1188.0
1	16496.7
1	473.4
1	1052.1
7	19502.5
16	134019.4
5	5946.4

1	736.5
11	136368.8
1	417.7
1	1052.1
1	473.4
1	210.4
1	2865.9
4	40505.2
14	161724.0
2	6327.7
1	1052.1
1	26302.1
3	4771.9
1	5260.4
1	45554.2
1	736.5
5	19805.5
2	4208.3
1	10520.8
2	5643.4
2	9021.6
2	16096.9
1	1028.5
6	5139.5
1	2519.9
1	205.7
1	18513.2
1	129.6
2	16456.2
2	15942.0
1	25712.8
20	73150.1
6	78372.7
5	52570.4
1	2057.0
1	0.0
20	108390.1
1	205.7
5	30968.5
2	21418.9
3	8377.2
1	236.7
1	10285.1
6	29896.9
1	771.4
3	2611.4
3	742.3
2	15633.4

1	0.0
1	15427.7
3	23347.3
4	32912.4
9	33791.8
1	462.8
1	518.7
1	62.0
1	129.6
1	1028.5
1	7713.8
2	7207.8
2	3983.1
3	1016.7
1	1892.5
1	2592.9
1	2057.0
1	778.1
1	2057.0
1	1285.6
4	41449.1
2	5425.4
6	23100.4
2	2879.8
4	6428.2
5	65192.3
25	188350.6
4	40673.6
4	16263.9
1	2057.0
1	3085.5
3	76571.8
4	51906.0
1	48032.0
3	32912.4
2	21290.2
1	518.7
1	360.0
1	8733.1
7	21486.0
1	2809.2
1	251.5
3	19582.9
2	5130.7
2	10626.4
1	1028.5
8	207709.3
4	23005.8

2	2327.5
7	62885.3
1	2351.2
8	22156.9
1	1028.5
1	1028.5
1	510.9
1	3085.5
1	2079.9
2	5656.8
1	10285.1
6	1635.3
5	5471.7
3	6582.5
1	771.4
1	8228.1
3	8964.6
9	61969.6
1	475.1
2	9745.2
2	579.1
1	1626.1
4	3594.9
2	31883.9
3	22722.3
13	122253.9
1	2571.3
1	771.4
14	124200.5
1	11321.9
1	20428.5
1	1028.5
1	277.7
1	1028.5
1	2571.3
2	514.3
3	41140.5
6	48340.1
1	35998.0
1	1563.3
1	0.0
1	2571.3
3	14399.2
1	771.4
1	0.0
4	19688.8
1	0.0
1	4114.1

2	20914.8
1	5070.3
2	3042.2
1	595.8
2	4056.2
1	2028.1
1	2940.8
9	122041.7
1	3042.2
2	20854.0
3	20857.1
4	30421.7
1	60843.4
1	608.4
8	60166.5
6	89084.8
9	85180.7
3	583.1
1	7098.4
1	507.0
1	1014.1
20	86721.7
1	7098.4
8	133200.9
1	20281.1
6	234694.2
3	16934.7
2	10140.6
1	12675.7
5	65913.7
2	12417.3
1	11465.2
2	354.9
2	33463.9
1	507.0
3	3549.2
3	25604.9
1	243.4
5	39041.2
7	10280.6
9	425099.6
1	4089.1
1	1521.1
1	20179.7
1	20281.1
1	7605.4
1	8112.4
2	7098.4

2	342.6
4	7250.5
1	2433.7
3	17827.1
2	804.5
1	2028.1
3	3442.7
2	24984.5
3	22917.7
1	3042.2
1	507.0
1	6084.3
1	3042.2
3	11734.7
15	112989.0
18	178932.5
1	5070.3
2	10647.6
1	3042.2
8	205566.7
5	43874.2
1	507.0
5	31800.8
8	78082.3
5	59736.0
1	507.0
5	133910.2
1	1216.9
1	9944.8
1	33463.9
1	22682.4
1	6084.3
7	82397.2
1	152.1
1	304.2
6	80617.5
3	77459.8
2	16267.8
3	4563.3
1	2535.1
1	248.6
1	10951.8
1	19267.1
1	15210.8
1	3042.2
3	6601.5
9	38939.8
3	6135.0

9	69674.0
2	608.4
1	15210.8
1	4563.3
1	461.8
5	26839.5
1	639.6
1	579.3
1	2738.0
14	194892.6
2	1139.3
1	10063.5
2	3338.3
1	40562.2
13	179410.6
1	354.9
9	282414.7
2	26365.5
1	1014.1
1	671.8
2	4056.2
1	76054.2
1	19013.6
2	1014.1
1	5070.3
2	835.0
9	65132.8
1	770.7
4	9011.9
1	760.5
1	4056.2
2	27886.5
3	21428.0
1	405.6
3	19058.2
7	163600.7
5	32262.1
1	1262.5
9	189819.4
3	16160.0
4	10327.3
2	555.5
1	25250.0
6	52988.6
1	60600.0
4	3030.0
1	303.0
18	118527.0

1	3282.5
1	1616.0
10	108324.5
8	61610.0
4	18555.7
14	107478.3
1	3535.0
1	18852.7
1	5050.0
9	77810.4
11	1473965.2
6	40753.5
1	303.0
4	57945.7
1	2020.0
1	9090.0
1	19705.1
1	0.0
1	31916.0
1	666.6
5	17018.5
1	4155.1
1	8080.0
1	1574.6
5	63630.0
2	12378.2
14	662211.6
1	0.0
1	151.5
1	1010.0
5	47672.0
2	4559.1
1	5050.0
1	10254.5
2	9595.0
2	14402.6
44	899692.9
3	1347.3
1	15150.0
1	606.0
1	505.0
1	5050.0
1	13907.7
1	8888.0
1	0.0
3	19493.0
1	505.0
8	13847.3

14	190024.6
5	5972.6
2	6019.6
1	8080.0
2	4545.0
1	30322.2
1	34105.2
3	27270.0
3	20200.0
10	56661.0
1	526.2
1	1010.0
1	353.5
1	252.5
8	131184.9
1	0.0
1	25668.1
1	707.0
1	15.2
1	445.9
1	202.0
6	124798.6
1	1212.0
1	10100.0
1	1010.0
1	505.0
1	2020.0
1	0.0
7	47908.3
1	202.0
5	61027.2
8	95647.0
1	5050.0
1	1212.0
2	30300.0
2	32572.5
4	122715.0
9	64371.2
1	1520.2
1	1466.7
1	151.5
1	0.0
1	151.5
2	5302.5
2	32115.0
1	1010.0
1	2462.4
10	126755.0

3	20200.0
1	3030.0
1	757.5
1	252.5
14	164788.4
1	505.0
7	230060.8
1	303.0
2	15840.8
2	2473.5
1	505.0
1	151.5
1	10100.0
4	18060.7
1	5050.0
1	1010.0
1	252.5
2	62115.0
1	151.5
9	47571.0
5	83197.3
2	27401.3
1	5050.0
4	3838.0
4	70809.1
2	2525.0
1	15150.0
1	151.5
2	20701.0
1	4040.0
5	15361.1
1	50499.9
1	0.0
1	101.0
5	11772.1
1	5000.0
1	5000.0
2	45000.0
4	6620.0
1	15000.0
1	262.3
3	10160.5
1	1200.0
6	38722.8
1	20000.0
18	103102.5
6	46355.0
5	25000.0

1	1500.0
14	91536.8
1	3809.2
2	4000.0
2	9250.0
15	64682.6
1	10000.0
6	125466.9
2	40000.0
1	262.3
1	15000.0
1	9000.0
8	43810.0
2	2080.2
1	5000.0
3	7745.7
7	80375.0
1	824.2
15	35685.8
6	5808.1
4	1819.2
1	1000.0
2	5937.2
1	15000.0
6	18757.8
1	853.0
1	10000.0
2	650.0
2	120.0
3	9914.0
6	250684.0
1	262.3
55	972606.1
1	200.0
5	10786.9
1	500.0
1	262.3
1	1624.0
2	1351.0
1	15000.0
1	57537.0
5	35143.0
2	6400.0
2	13000.0
14	158595.7
5	7748.3
1	4000.0
1	150.0

15	227311.4
2	11500.0
1	3000.0
1	4000.0
5	15115.4
1	36000.0
1	2000.0
1	10000.0
4	32597.2
11	38214.9
1	228.0
10	57018.2
2	21101.4
14	98362.3
1	257.6
1	150.0
1	15000.0
3	7068.8
3	46207.0
2	15000.0
8	87500.0
3	3900.0
3	1139.7
2	280.0
2	2355.5
1	603.4
1	6000.0
1	45.0
1	1000.0
1	9600.0
1	300.0
4	48000.0
13	118394.2
15	121433.8
2	1600.0
1	1500.0
1	3400.0
1	15000.0
1	10000.0
1	7405.6
1	1685.0
1	1250.0
4	1984.0
11	83416.0
4	28328.7
1	400.0
1	8500.0
1	10000.0

1	44000.0
2	412.3
1	5000.0
9	105050.0
1	4000.0
18	165556.8
9	178153.0
1	4500.0
1	20000.0
1	3000.0
1	981.0
1	2000.0
3	1139.3
1	30000.0
1	224.0
2	850.0
1	1000.0
1	1000.0
3	51000.0
2	2538.0
3	51568.0
1	5000.0
7	9052.8
1	23754.0
2	15000.0
3	9851.0
7	56002.3
2	262000.0
1	257.6
1	5000.0
3	91163.6
1	18000.0

Web Supplement 15. Recipients - payments missing from patient organisation data (all years)

Patient organisation name

21 and Co
AADC Research Trust
Action Bladder Cancer UK
Action Duchenne
Action on Pain
Action on Smoking and Health - Scotland
Actionplus Foundation
Addenbrooks Charitable Trust
African-Caribbean Leukaemia Trust
African's Getting Involved
Age Related Diseases and Health Trust
aHUS UK
ALD Life
Allergy Research Foundation
Alpha-1 Awareness UK
Alpha-1 UK Support Group
Alzheimer Scotland
Alzheimers Dementia Support
Angelman Syndrome Support Education Research Trust
Ankylosing Spondylitis International Federation
Anticoagulation UK
Aplastic Anaemia Trust
Arrhythmia Alliance
Arthritis and Musculoskeletal Alliance
Asthma and Allergy Foundation
Asthma Relief
Asyabi UK
Atrial Fibrillation Association
Back on Track
Barts and The London Charity
Basil Skyers Myeloma Foundation
Beat Parkinson’s
Beating Bowel Cancer
Behcets Syndrome Society
Bipolar Scotland
Bipolar UK
Birdshot Uveitis Society
Bladder and Bowel Foundation (taken over by Bladder and Bowel Community in 2017)
Blood Pressure UK
Bluebell Foundation
Body and Soul
Bone Cancer Research Trust
Brain Charity
Brain Tumor Charity
Brain Tumour Research
Breast Cancer Campaign (current name Breast Cancer Now, following merger with Breakthrough Breast Canc

Breast Cancer Haven (The Haven)
British Dupuytren's Society
British Heart Foundation
British Obesity Society
British Polio Fellowship
British Skin Foundation
Brook
Building for the Future
Cambridge Rare Disease Network
Cancer 52
Cancer Focus Northern Ireland
Cancer Research UK
Candlelighters
Cardiac Risk in the Young
Cardiomyopathy UK
Catholics for AIDS Prevention and Support
Charlie Waller Memorial Trust
Children with Cancer UK
Children's Liver Disease Foundation
Children’s Heart Unit Fund
Chinese National Healthy Living Centre
Chronic Lymphocytic Leukaemia Support Association
Cleft Lip and Palate Association
CLIC Sargent Cancer Care For Children
Community Cancer Centre
Contact a Family
Coping with Cancer in Leicestershire and Rutland
Crohn's in Childhood Research Association
Cure Parkinson's Trust
Cystic Fibrosis Trust
David Lewis Centre
Deaf Education through Listening and Talking
Delete Blood Cancer
Dementia UK
Diabetes Research and Wellness Foundation
Diabetics with Eating Disorders
Diamond Blackfan Anaemia UK
Different Strokes
Douglas Bader Foundation
Edinburgh and Lothian Prostate Cancer Support Group
Encephalitis Society
Epilepsy Connections
Epilepsy Scotland
FORCE
Forum Link
Freshwinds
Group B Strep Support UK
Guillain Barre and associated inflammatory neuropathies

Haemophilia Society
Haemophilia Wales
Hafal
Headway
Health and Social Care Alliance Scotland
Heartbeat
Hepatitis B Positive Trust
HIV i-Base
Hughes Syndrome Foundation
ICUsteps
Imperial College Healthcare Charity
Independent Cancer Patients' Voice
Input Diabetes
KAITY
Karen Clifford Skcin cancer charity
Kidney Cancer Support Network
Kidney Cancer UK
Kidney Care UK
LATCH Welsh Children's Cancer Charity
Leeds Teaching Hospital Charitable Foundation
Leicestershire AIDS Support Services
Life Education Wessex
Little Hearts Matter
Liver4Life
Lullaby Trust
Lupus Europe
Lupus UK
Macular Society
Maggie's Centre
Make A Wish Foundation
Malnutrition and Nutritional Care in the UK
MAMA Academy
Marie Curie Cancer Care
Martin Fisher Foundation
Melanoma UK
Mesothelioma UK
Motor Neurone Disease Association
Movember Foundation
MPN Voice
Multi Organ Transplant Support
Multiple Births Foundation
Multiple Sclerosis UK
Myasthenia Gravis Association
Myrovlytis Trust
NARA - The Breathing Charity
National Association for Pre-Menstrual Syndrome
National Cancer Research Institute
National Eczema Society

National Kidney Federation
National Obesity Forum
National Osteoporosis Society
NMO Research Foundation
North of England Bone Marrow and Thalassaemia Association
Northern Ireland Chest, Heart and Stroke
Northern Ireland Rare Disease Partnership
OcuMel UK
Olive Tree Cancer Support Centre - Crawley
Oliver King Foundation
Orchid
Organisation for Sickle Cell Anaemia Research and Thalassaemia Support
Ovacome
Paget's Association
Pain Association Scotland
Pain Concern
Pain UK
Patients Association
Patients On Intravenous and Nasogastric Nutrition Therapy
Paula Carr Diabetes Trust
Pelican Cancer Foundation
Pelvic Pain Support Network
Pink Ribbon Foundation – Breast Cancer Charity
Polycystic Kidney Disease Charity
Portsmouth Down Syndrome Association
Positive Action
Prader-Willi Syndrome Association UK
Progressive Supranuclear Palsy Association
Prostate Cancer Research Centre
Prostate Cymru
QUIT
Rainbow Centre for Conductive Education
Rainbow Trust Children's Charity
Rarer Cancers Foundation
Restless Leg Syndrome UK
Richmond Fellowship
River House Trust
Ronald McDonald House Charities
Roy Castle Lung Cancer Foundation
Royal Marsden Cancer Charity
Salamander Trust
Samson Centre for MS
SANE
Scoliosis Association UK
Scotland Patients Association
Scottish Drugs Forum
Scottish Epilepsy Initiative
Scottish Kidney Federation

Scottish Network Children with Arthrities
SeeAbility
Self Help Services
Sense
Service by Emergency Response Volunteers - Suffolk and Cambridgeshire
Short Bowel Survivor and Friends
Silver Star
Skin Care Cymru
Somerville Foundation
Sophia Forum
Spectra
Spinal Injuries Association
Spinal Injuries Scotland
St Thomas Lupus Trust
Steps
SUDEP Action
Sussex Beacon
Tackle Prostate Cancer
Team Margot
Tenovus Cancer Care
Thames Valley Positive Support
Thrombosis UK
Transplant Links
Transverse Myelitis Society
Tree of Hope
Trigeminal Neuralgia Association
UK Chronic Lymphocytic Leukaemia Forum
UK Gout Society
UK Sepsis Trust
Walk the Walk Worldwide
Waverley Care
Willow Foundation
World Cancer Research Fund
World Child Cancer
York Air Ambulance

Number of payments	Value of payments (2016 £)
1	1028.5
1	5070.3
1	101.0
12	47076.4
12	13947.1
1	2519.9
2	7940.8
1	3042.2
6	70151.6
1	129.6
2	39258.9
1	25250.0
1	262.3
2	21041.7
2	16456.2
5	138585.3
2	563.6
1	1057.3
1	303.0
6	38722.8
76	375206.6
1	1616.0
34	329518.1
32	262194.0
1	507.0
1	1014.1
1	1500.0
104	621144.3
1	3809.2
1	205.7
4	35201.1
1	5050.0
42	342673.1
1	20281.1
3	3193.8
3	4145.2
1	10000.0
38	2271217.4
1	10520.8
2	776.4
6	97945.7
3	12160.6
1	981.5
1	12675.7
1	262.3
7	81316.2

5	47893.8
8	55592.3
7	1881.6
1	420.8
1	0.0
6	9784.6
12	95961.9
1	243.4
4	9320.3
26	249099.2
13	29164.3
57	1817634.9
1	0.0
7	6270.9
5	2338.0
1	4089.1
1	1000.0
2	624.9
2	16010.0
1	736.5
1	252.5
12	86711.0
2	120.9
3	5412.1
1	1028.5
3	20369.3
1	4187.3
9	27581.2
9	24748.9
17	281196.7
1	1892.5
1	526.0
3	14664.9
1	2592.9
3	6594.9
4	1547.3
1	15150.0
5	10786.9
2	1384.1
2	804.5
2	767.3
2	4085.1
7	6116.9
1	507.0
6	26164.4
2	11930.2
1	153.6
2	6400.0

57	577521.6
10	13720.9
1	4000.0
1	150.0
1	37.6
1	757.5
1	5070.3
13	63461.6
1	2104.2
2	4156.5
1	4000.0
11	29891.2
1	30322.2
1	2000.0
10	91665.0
9	101336.2
37	183714.4
2	754.3
1	1010.0
1	353.5
4	22216.1
1	518.7
1	252.5
24	248299.0
1	0.0
4	55502.5
1	707.0
1	15.2
3	3412.9
2	764.6
1	883.8
1	202.0
3	643.0
1	15000.0
24	386833.4
7	34571.2
2	280.0
1	1010.0
6	44237.3
1	6084.3
1	505.0
1	603.4
1	2020.0
1	1420.3
1	0.0
1	152.1
1	1000.0
14	23675.7

30	396718.0
3	3508.5
35	326740.6
3	6650.0
4	5775.3
1	1028.5
2	4035.1
2	2080.6
1	3400.0
1	510.9
2	3334.2
2	4184.1
6	64181.1
3	24720.6
10	4636.2
11	16888.4
10	30100.6
48	345520.4
5	29848.9
1	400.0
1	8500.0
4	2654.7
1	10000.0
4	68956.0
1	151.5
1	0.0
1	473.4
3	563.8
1	1052.1
1	639.6
13	79339.8
1	579.3
1	1010.0
62	682970.9
5	12939.6
1	736.5
1	252.5
1	771.4
70	770325.1
1	354.9
2	720.7
1	4500.0
7	83686.8
1	277.7
1	1028.5
2	4056.2
2	78625.5
1	505.0

3	1139.3
1	473.4
2	514.3
1	151.5
1	210.4
1	224.0
12	117958.9
8	19924.8
3	7327.7
2	10120.3
1	1010.0
1	252.5
1	1052.1
4	62950.0
1	151.5
2	2334.0
1	1000.0
13	148469.6
1	5050.0
9	170502.5
2	5736.5
17	45782.5
2	1531.9
1	15150.0
1	151.5
3	4208.3
1	4056.2
3	38407.4
1	0.0
1	405.6
18	103557.2
1	257.6
1	5000.0
15	342275.9
1	0.0

Web Supplement 16. Shared ties in industry and patient organisation data based on the number and value

Ties calculated based on the number of payments

Total number of ties reported in industry data involving at least one payment
Total number of ties reported in patient organisation data involving at least one payment

Number of shared ties (donors and recipients are the same in industry and patient organisation data)
Shared ties as a share of all ties in industry data
Shared ties as a share of all ties in patient organisation data

Number of ties involving the same number of payments
Number of ties involving the same number of payments as a share of shared ties

Number of payments forming ties with the equal number of payments in both datasets
Payments forming ties with the equal number of payments in both datasets as a a share of all payments in in[illegible]
Payments forming ties with the equal number of payments in both datasets as a a share of all payments in pa[illegible]

Ties calculated based on the value of payments

Total number of ties reported in industry data involving at least one payment with value >£0
Total number of ties reported in patient data involving at least one payment with value >£0

Number of shared ties (donors and recipients are the same in industry and patient organisation data)
Shared ties as a share of all ties in industry data
Shared ties as a share of all ties in patient organisation data

Number of shared ties involving the same value of payments
Shared ties involving the same alue of payments as a share of shared ties

Value of payments in which donors and recipients provided the same value (2016 £)
Value of shared payments as a share of payments with values >£0 in industry data
Value of shared payments as a share of payments with values >£0 in patient organisation data

All years	2012	2013	2014	2015	2016
1101	369	366	432	479	523
626	252	276	293	319	287
428	110	122	146	168	160
38.9%	29.8%	33.3%	33.8%	35.1%	30.6%
68.4%	43.7%	44.2%	49.8%	52.7%	55.7%
87	45	66	69	89	64
20.3%	40.9%	54.1%	47.3%	53.0%	40.0%
162	47	69	79	97	73
3.8%	6.1%	9.8%	9.6%	10.4%	6.7%
9.8%	16.2%	21.2%	22.8%	26.9%	21.7%
1088	366	357	431	470	522
326	127	122	119	150	140
34	21	24	28	32	24
3.1%	5.7%	6.7%	6.5%	6.8%	4.6%
10.4%	16.5%	19.7%	23.5%	21.3%	17.1%
20	17	22	26	30	23
58.8%	81.0%	91.7%	92.9%	93.8%	95.8%
325,108.4	216,055.8	201,362.3	251,964.6	517,139.2	482,216.0
0.6%	2.8%	3.3%	2.9%	4.5%	2.4%
1.0%	6.2%	4.6%	4.8%	10.7%	3.2%

Web Supplement 17. Overlap between recipient lists reported by donors and patient organ

	Total number of donors with at least one payment	Minimum overlap with patient organisation data	Number of donors with minium overlap with patient organisation data	Donors with minium overlap with patient organisation data as a share of all donors	Number of donors with less than 50% overlap with patient organisation data	Donors with less than 50% overlap with patient organisation data as a share of all donors
All years	63	0.0%	6	9.5%	40	63.5%
2012	30	0.0%	7	23.3%	23	76.7%
2013	38	0.0%	9	23.7%	25	65.8%
2014	45	0.0%	12	26.7%	35	77.8%
2015	51	0.0%	11	21.6%	33	64.7%
2016	44	0.0%	10	22.7%	32	72.7%

isation data - yearly breakdown based on the number of paymen

Maximum overlap with patient organisation data	Number of donors with maximum overlap with patient organisation data	Donors with maximum overlap with patient organisation data as a share of all donors
100.0%	3	4.8%
100.0%	1	3.3%
100.0%	4	10.5%
100.0%	3	6.7%
100.0%	3	5.9%
100.0%	3	6.8%

Web Supplement 18. Overlap between recipient lists reported by donors and patient organ

	Number of donors with at least one payemnt with the value >£0	Minimum overlap with patient organisation data	Number of donors with minium overlap with patient organisation data	Donors with minium overlap with patient organisation data as a share of all donors	Number of donors with less than 50% overlap with patient organisation data	Donors with less than 50% overlap with patient organisation data as a share of all donors
All years	62	0.0%	17	27.4%	56	90.3%
2012	30	0.0%	13	43.3%	29	96.7%
2013	38	0.0%	18	47.4%	36	94.7%
2014	45	0.0%	26	57.8%	45	100.0%
2015	50	0.0%	21	42.0%	42	84.0%
2016	44	0.0%	15	34.1%	39	88.6%

isation data - yearly breakdown based on the value of payments

Maximum overlap with patient organisation data	Number of donors with maximum overlap with patient organisation data	Donors with maximum overlap with patient organisation data as a share of all donors
100.0%	2	3.2%
50.0%	1	3.3%
66.7%	1	2.6%
40.0%	1	2.2%
100.0%	1	2.0%
66.7%	1	2.3%

Web Supplement 19. Overlap between donor lists reported by recipeints and industry data

	Number of recipients with at least 1 payment	Minimum overlap with industry data	Number of recipients with minium overlap with industry data	Recipients with minium overlap with industry data as a share of all recipients	Number of recipients with less than 50% overlap with industry data	Recipients with less than 50% overlap with industry data as a share of all recipients
All years	200	0.0%	14	7.0%	32	16.0%
2012	103	0.0%	37	35.9%	51	49.5%
2013	102	0.0%	38	37.3%	50	49.0%
2014	116	0.0%	34	29.3%	49	42.2%
2015	126	0.0%	37	29.4%	47	37.3%
2016	117	0.0%	28	23.9%	40	34.2%

- yearly breakdown based on the number of payments

Maximum overlap with industry data	Number of recipients with maximum overlap with industry data	Recipeints with maximum overlap with industry data as a share of all recipients
100.0%	102	51.0%
100.0%	29	28.2%
100.0%	24	23.5%
100.0%	41	35.3%
100.0%	49	38.9%
100.0%	50	42.7%

Web Supplement 20. Overlap between donor lists eported by recipeints and industry data -

	Number of patient organisations with at least one payemnt with the value >0	Minimum overlap with industry data	Number of recipients with minium overlap with industry data	Recipients with minium overlap with industry data as a share of all recipients	Number of recipients with less than 50% overlap with industry data	Recipients with less than 50% overlap with industry data as a share of all recipients
All years	121	0.0%	12	9.9%	18	14.9%
2012	57	0.0%	22	38.6%	30	52.6%
2013	51	0.0%	19	37.3%	26	51.0%
2014	58	0.0%	17	29.3%	21	36.2%
2015	72	0.0%	22	30.6%	26	36.1%
2016	57	0.0%	10	17.5%	15	26.3%

· yearly breakdown based on the value of payments

Maximum overlap with industry data	Number of recipients with maximum overlap with industry data	Recipeints with maximum overlap with industry data as a share of all recipients
100.0%	71	58.7%
100.0%	16	28.1%
100.0%	14	27.5%
100.0%	27	46.6%
100.0%	35	48.6%
100.0%	31	54.4%
