# Supplementary material for: Under-reported relationship: a comparative study of pharmaceutical industry and patient organisation payment disclosures in the UK (2012–2016)
Source: BMJ Open. 2020 Sep 19;10(9):e037351. doi: 10.1136/bmjopen-2020-037351 (PMC7511620; doi:10.1136/bmjopen-2020-037351)
Supplement: Author's manuscript [file bmjopen-2020-037351.draft_revisions.pdf]

BMJ Open is committed to open peer review. As part of this commitment we make the peer review history of every article we publish publicly available.

When an article is published we post the peer reviewers' comments and the authors' responses online. We also post the versions of the paper that were used during peer review. These are the versions that the peer review comments apply to.

The versions of the paper that follow are the versions that were submitted during the peer review process. They are not the versions of record or the final published versions. They should not be cited or distributed as the published version of this manuscript.

BMJ Open is an open access journal and the full, final, typeset and author-corrected version of record of the manuscript is available on our site with no access controls, subscription charges or pay-per-view fees (http://bmjopen.bmj.com).

If you have any questions on BMJ Open's open peer review process please email info.bmjopen@bmj.com

BMJ Open

An underreported relationship: a comparative study of pharmaceutical industry and patient organisation payment disclosures in the UK (2012-2016)

Journal:	*BMJ Open*
Manuscript ID	bmjopen-2020-037351
Article Type:	Original research
Date Submitted by the Author:	30-Jan-2020

Complete List of Authors:	Ozieranski, Piotr; University of Bath, Social and Policy Sciences<br>Csanádi, Marcell ; Syreon Research Institute<br>Rickard, Emily; University of Bath, Social and Policy Sciences<br>Mulinari, Shai; Lunds Universitet, Sociology

Keywords:	Health policy < HEALTH SERVICES ADMINISTRATION & MANAGEMENT, Protocols & guidelines < HEALTH SERVICES ADMINISTRATION & MANAGEMENT, MEDICAL ETHICS

SCHOLARONE™
Manuscripts

I, the Submitting Author has the right to grant and does grant on behalf of all authors of the Work (as defined in the below author licence), an exclusive licence and/or a non-exclusive licence for contributions from authors who are: i) UK Crown employees; ii) where BMJ has agreed a CC-BY licence shall apply, and/or iii) in accordance with the terms applicable for US Federal Government officers or employees acting as part of their official duties; on a worldwide, perpetual, irrevocable, royalty-free basis to BMJ Publishing Group Ltd ("BMJ") its licensees and where the relevant Journal is co-owned by BMJ to the co-owners of the Journal, to publish the Work in this journal and any other BMJ products and to exploit all rights, as set out in our licence.

The Submitting Author accepts and understands that any supply made under these terms is made by BMJ to the Submitting Author unless you are acting as an employee on behalf of your employer or a postgraduate student of an affiliated institution which is paying any applicable article publishing charge ("APC") for Open Access articles. Where the Submitting Author wishes to make the Work available on an Open Access basis (and intends to pay the relevant APC), the terms of reuse of such Open Access shall be governed by a Creative Commons licence – details of these licences and which Creative Commons licence will apply to this Work are set out in our licence referred to above.

Other than as permitted in any relevant BMJ Author's Self Archiving Policies, I confirm this Work has not been accepted for publication elsewhere, is not being considered for publication elsewhere and does not duplicate material already published. I confirm all authors consent to publication of this Work and authorise the granting of this licence.

For peer review only

An underreported relationship: a comparative study of pharmaceutical industry and patient organisation payment disclosures in the UK (2012-2016)

Piotr Ozieranski,[1*] Marcell Csanadi,[2] Emily Rickard,[3] Shai Mulinari [4]

[1] – Department of Social and Policy Sciences, University of Bath, Claverton Down, Bath, UK

[2] – Syreon Research Institute, Budapest, Hungary

[3] – Department of Social and Policy Sciences, University of Bath, Claverton Down, Bath, UK

[4] – Department of Sociology, Lund University, Lund, Sweden

*Corresponding author email: p.ozieranski@bath.ac.uk

Key words: pharmaceutical industry, patient organisations, conflicts of interest, financial disclosures, transparency

Wordcount: 4190

For peer review only

Abstract

Objectives: To examine the underreporting of pharmaceutical company payments to patient organisations by donors and recipients.

Design: Comparative descriptive analysis of payments disclosed on drug company and charity regulator websites.

Setting: UK.

Participants: 87 donors (drug companies) and 425 recipients (patient organisations) reporting payments in 2012-2016.

Main outcome measures: Number and value of payments reported by donors and recipients; differences in payments from/to the same donors and recipients; payments reported in either dataset but not the other one; agreement between donor-recipient ties established by payments; overlap between recipient and donor lists and, respectively, patient organisation and industry data.

Results: Of 87 donors, 63 (72.4%) reported payments but 84 (96.6%) were mentioned by recipients. Although donors listed 425 recipients, only 200 (47.1%) reported payments. The number and value of payments reported by donors were 259.8% and 163.7% greater than those reported by recipients, respectively. The number of donors with matching payment numbers and values in both datasets were 3.4% and 0%, respectively; for recipients these figures were 7.8% and 1.9%. There were 24 and 3 donors missing from industry and patient organisation data during the entire study period, representing 38.1% and 3.6% of those in the respective datasets. The share of donor-recipient ties in which industry and patient organisation data agreed about donors and recipients was 38.9% and 68.4% in each dataset, respectively. Of 63 donors reporting payments, only 3 (4.8%) had their recipient lists fully overlapping with patient organisation data. Of 200 recipients reporting industry funding, 102 (51.0%) had their donor lists fully overlapping with industry data.

Conclusions: Both donors and recipients underreported payments. Existing donor and recipient disclosure systems cannot manage potential conflicts of interest associated with industry payments. Increased standardisation could limit the underreporting by each side but only an integrated donor-recipient database could eliminate it.

Strengths and limitations

- We examine the underreporting of pharmaceutical industry payments to patient organisations using large samples of donors and recipients over a period of time.
- We systematically compare the underreporting by donors and recipients using five complementary measures at different levels of analysis
- One key limitation is that the samples of donors and recipients were not exhaustive

For peer review only

- The full extent of underreporting remains unknown as no definitive list of payments exists

For peer review only

For peer review only - http://bmjopen.bmj.com/site/about/guidelines.xhtml

Introduction

Many patient organisations accept funding from drug companies. A recent UK analysis showed that the industry donated over £57m (€65m; $73m) to 508 patient organisations, with the annual sum more than doubling from 2012 to 2016.[1] Another study of 289 US patient organisations found that 156 (67%) received funding from for-profit companies, with a median proportion of 45% of their income coming from drug, medical device, or biotechnology companies.[2] Although industry funding may benefit patient organisations,[3 4] it raises concerns about potential conflict of interests compromising patient organisations' independence and credibility.[5 6 7 8 9]

In responding to these concerns, both donors[10] and recipients[4] assert that any conflict of interests can be managed by careful disclosure of funding. On the donor side, since 2012 members of pharmaceutical industry trade groups in European countries,[11] such as the Association of the British Pharmaceutical Industry (ABPI), have disclosed payments to patient organisations annually on each company's website, including their monetary value and purpose.[12] In the UK, the number of companies subscribing to the ABPI Code, and therefore committing to the disclosure of payments to patient organisations, also includes over sixty non-members of the ABPI; hence, nearly every relevant company is covered.[13] However, the self-regulatory approach to payment disclosure has major shortcomings, including absent reports, unclear or inadequate payment descriptions, and unstandardised reporting.[1 14] Likewise, on the recipient side, disclosures published on patient organisations' websites have been criticised as incomplete or uninformative.[15 16 17 18 19] In the UK, a possibly more reliable, yet rarely examined,[14 20] source of disclosures are mandatory annual accounts of patient organisations registered as charities with an annual income over £25,000. These accounts serve a dual-purpose of detailing the charity's activities and ensuring financial transparency by listing sources of income and how income is spent.

Notwithstanding the shortcomings of each side's disclosures, and the potentially complementary information they provide, donor and recipient disclosures are rarely compared. One exception is a recent UK study which identified companies not disclosing payments to patient organisations being mentioned in their annual accounts as donors.[14] Another UK study found discrepancies between annual accounts of some patient organisations contributing to health technology assessment in England and drug company payment disclosures.[20] More broadly, cross-interrogation of different data sources has revealed undisclosed industry ties among treatment guideline panellists,[21 22 23] clinical trialists, authors of medical journal articles,[24 25 26 27 28] and some clinical commissioning groups[29] and NHS trusts in England.[30]

We examine the underreporting of payments to UK patient organisations from 2012 to 2016 by comparing payment disclosures made by 87 companies to 425 patient organisations with the annual accounts of the same set of patient organisations. Specifically, by considering the extent to which the industry and patient organisation disclosures differ in information about who provided and received funding, and how much was paid and received, we examine the reliability of the two disclosures systems said to neutralise concerns about conflict of interests.

For peer review only - http://bmjopen.bmj.com/site/about/guidelines.xhtml

Methods

Data sources and extraction

Our sample of drug companies comprised all 108 participants of Disclosure UK in 2015, including 53 ABPI members (web supplement 1). Disclosure UK is a self-regulatory initiative covering payments to healthcare organisations[31] and professionals,[32] but because its participants subscribe to the ABPI Code, they should also separately disclose, on their websites, payments to patient organisations.[1 14] In June 2017 and January 2018, ER searched the company websites, identifying 220 disclosure reports published between 2012 and 2016. ER downloaded them and extracted payment data into a single Excel spreadsheet, converting all payment values to 2016 sterling. There were 66 (61.1%) companies disclosing payments in at least one year during this period. Following the exclusion of ineligible payments based on their timing or recipient characteristics, we established that 64 companies reported 4,572 payments, worth £57,305,289.2 to 489 UK patient organisations (web supplement 1).

In September 2017 and July 2018, ER searched for these organisations on the websites of the UK charity regulators – the Charity Commission for England and Wales, the Scottish Charity Regulator and the Northern Irish Charity Regulator – to which registered charities must submit their annual accounts. We considered accounts from financial years ending in 2012 to 2017 to cover all calendar year data from drug company reports. We found that 425 (86.9%) patient organisations drug companies listed as recipients published their annual accounts at least once with at least one charity regulator. In industry data, 63 (98.4%) companies reported 4,316 (94.4%) payments, worth £54,071,454.21 (94.4%) to these patient organisations.

ER read all annual accounts and extracted any information pertaining to drug company payments to an Excel spreadsheet. As with industry data, if several values were mentioned in a single payment description they were considered as separate payments. As patient organisation yearly accounts lack specific monthly end date,[20] we turned those covering months up to June into the previous calendar year, and the rest into the current calendar year. The 425 patient organisations registered as charities reported 4,372 payments, worth £58,668,293.6, from 167 donors. Following exclusion criteria referring to donor and recipient characteristics as well as the conversion of financial into calendar years (web supplement 2), we established that 200 (47.1%) organisations reported 1,661 (38.0%) payments, worth £33,037,955.8 (56.3%), from 84 (47.1%) drug companies. In particular, we excluded 2,014 payments, worth £1,992,147.3, made by 61 companies found in patient organisation data but missing from the original sample of Disclosure UK participants.

ER and PO checked the details of the data extraction. Any exclusions of payments were agreed.

The ethical implications of this study article were approved via a peer ethics review process at the Department of Social and Policy Sciences, University of Bath in April 2016. This study did not

For peer review only - http://bmjopen.bmj.com/site/about/guidelines.xhtml

require a full ethical approval as it relied on publicly available data aggregated at the organisational level.

Analysis

MC and PO converted the data into donor-recipient matrices and analysed it descriptively in Excel. We examined the overall pattern of the number and value of payments in each dataset and its changes over time. To provide further context for the reported results we present additional data as web supplements (3-20) throughout the article [*Please note that these web supplements are only available in the Excel file format and therefore could not be uploaded as part of the online submission; we will provide them via email when requested by the Editorial Office*].

Outcome measures

Absent an exhaustive list of payments, enabling a direct comparison of industry and patient organisation data, we studied underreporting with five complementary measures.

First, we compared the overall volume of payments reported in industry and patient organisation data, including payments lacking specific values.

Second, we examined the absolute and relative differences in payments reported in relation to the same donors and recipients in industry and patient organisation data. We calculated the absolute difference as the difference between the number/value of payments reported in the two datasets; the relative difference was the absolute difference divided by the number/value of payments in the dataset with the higher number/value of payments. To compare larger and smaller donors and recipients we considered absolute and relative differences at different thresholds of the overall number (i.e. ≥1, >1, >10, >100 in at least one dataset) and value of payments (i.e. >£0, >£10,000, >£100,000 in at least one dataset).

Third, focusing on the highest relative differences, in which donors or recipients were present in one but absent from the other dataset, we calculated the number and value of payments related to donors and recipients missing from each dataset.

Fourth, we compared the extent to which industry and patient organisation data conveyed the same pattern of connections between donors and recipients. Specifically, we analysed the overlap of ties (or connections) between donors and recipients – established by the presence of payments – in each dataset.

Finally, building on the analysis of donor-industry ties at the level of the two datasets, we moved to the donor and recipient level by examining the extent of overlap between recipient and donor lists in patient organisation and industry data, respectively.

Patient and public involvement

No patients were involved in this study.

For peer review only - http://bmjopen.bmj.com/site/about/guidelines.xhtml

Results

Overview of industry and patient organisation data

Although 63 donors reported payments, 84 were mentioned by recipients. In industry data, donors disclosed payments to 425 recipients but only 200 (47.1%) of those reported payments during the study period (Table 1).

The number of payments disclosed by donors was 259.8% greater than those disclosed by recipients (Table 1). Each side was disclosing more payments over time, but the increase from 2012 to 2016 was greater in industry data (141.2% v 115.8%, respectively). In industry data, 4,235 (98.1%) payments reported by 62 (98.4%) donors to 416 (97.9%) recipients had values greater than 0. In patient organisation data, only 772 (46.5%) payments reported by 121 (60.5%) recipients from 62 (73.8%) donors had values greater than 0. The value of payments disclosed by donors was 163.7% higher than those disclosed by recipients. Over the five-year period the value of payments reported by donors increased by 257.6%, while those by recipients – by 434.7%.

For peer review only

Table 1 Patterns in payments reported by donors and recipients

	All years	2012	2013	2014	2015	2016
**Calculations based on the number of payments**						
Number of payments - industry data	4316	769	706	821	934	1086
Number of payments - patient organisation data	1661	291	326	346	361	337
Number of drug companies with at least one payment - industry data	63	30	38	45	51	44
Number of drug companies with at least one payment - patient organisation data	84	56	65	65	69	65
Number of patient organisations with at least one payment - industry data	425	197	198	225	244	244
Number of patient organisations with at least one payment - patient organisation data	200	103	102	116	126	117
**Calculations based on the value of payments**						
Number of payments with value >£0 - industry data	4235	748	682	813	915	1077
Number of payments with value >£0 - patient organisation data	772	144	148	144	173	163
Value of payments - industry data (2016 £)	54,071,454.2	7,797,600.4	6,189,152.7	8,583,101.6	11,415,293.2	20,086,306.3
Value of payments - patient organisation data (2016 £)	33,037,955.8	3,459,605.6	4,414,328.4	5,301,728.3	4,825,091.2	15,037,202.3
Number of drug companies with at least one payment with the value >£0 - industry data	62	30	38	45	50	44
Number of drug companies with at least one payment with the value >£0 - patient organisation data	62	42	44	42	46	44
Number of patient organisations with at least one payment with the value >£0 - industry data	416	197	193	225	237	244
Number of patient organisations with at least one payment with the value >£0 - patient organisation data	121	57	51	58	72	57

For peer review only - http://bmjopen.bmj.com/site/about/guidelines.xhtml

Record of the same donors and recipients in industry and patient organisation data

Having considered the overall pattern of payments, we now compare those reported in relation to the same donors and recipients in each dataset.

Donors

Of the 87 donors identified in industry and patient organisation data, three (3.4%) had matching payment numbers, and 49 (56.3%) had more payments in patient organisation data (Table 2; web supplement 3). In relative terms, only 21 (24.1%) donors had a difference in the number of payments less than 50%, and 27 (31.0%) had a difference of 100%, equivalent to only either donors or recipients reporting payments.

In both datasets, 74 (85.1%) donors had payment values greater than 0 (web supplement 3). Of those, 48 (64.9%) had a higher payment value in industry data and the remaining ones – in patient organisation data. Only 14 (18.9%) donors with payment values greater than 0 had a relative difference lower than 50%, while 24 (32.4%) donors had a relative difference of 100%.

Overall, donors with a high overall number and value of payments in either dataset typically had high absolute differences (web supplement 3). Contrastingly, donors with a small number or value of payments usually had higher relative differences, that is, high relative differences often resulted from few payments or payments of small value in one dataset but none in the other.

Over time, there was little improvement in the shares of donors with matching numbers of payments (from 1.6% in 2012 to 5.5% in 2016); the share of donors with matching payment values showed no improvement (0%). However, the shares of those with relative differences in payment numbers or values lower than 50% increased (from 11.5 to 17.4%; and from 7.7% to 25.5%, respectively) (web supplement 4).

For peer review only

Table 2 Donors – absolute and relative differences in the number and value of payments

	All years	%
**Calculations based on the number of payments - absolute differences**		
Number of drug companies with at least one payment in at least one dataset - industry and patient organisation data	87	
Number of drug companies with exact match in both datasets	3	3.4%
Number of drug companies with more payments in industry data	49	56.3%
Number of drug companies with more payments in patient organisation data	35	40.2%
Highest absolute difference between patient organisation and industry data - number of payments higher in industry data	757	
Highest absolute difference between patient organisation and industry data - number of payments higher in patient organisation data	53	
**Calculations based on the number of payments - relative differences**		
Number of drug companies with relative difference <10%	5	5.7%
Number of drug companies with relative difference <20%	9	10.3%
Number of drug companies with relative difference <50%	21	24.1%
Number of drug companies with relative difference = 100%	27	31.0%
**Calculations based on the value of payments - absolute differences**		
Number of drug companies with at least one payment with value >£0 in at least one dataset	74	100%
Number of drug companies with exact match in both datasets	0	0.0%
Number of drug companies with higher payment value in industry data	48	64.9%
Number of drug companies with higher payment value in patient organisation data	26	35.1%

For peer review only

Highest absolute difference between patient organisation and industry data - number of payments higher in industry data (2016 £)	6,406,351.2	
Highest absolute difference between patient organisation and industry data - number of payments higher in patient organisation data (2016 £)	2,960,716.0	

Calculations based on the value of payments - relative differences

Number of drug companies with relative difference <10%	3	4.1%
Number of drug companies with relative difference <20%	9	12.2%
Number of drug companies with relative difference <50%	14	18.9%
Number of drug companies with relative difference = 100%	24	32.4%

For peer review only

Recipients

Of the 425 recipients identified in industry data, 33 (7.8%) had matching payments numbers in both datasets, while 335 (78.8%) had a greater number in industry data (Table 3, web supplement 5). In relative terms, 225 (52.9%) recipients had payments reported only in industry data (the relative difference of 100%), while 81 (19.1%) had a relative difference lower than 50%.

Of 425 recipients, 416 (97.9%) had payments with a value greater than 0 in at least one dataset (web supplement 5). Of those, 356 (85.6%) had a higher value of payments in industry data, and 8 (1.9%) had matching payment values. In relative terms, 295 (70.9%) recipients had payments reported exclusively in industry data, and 73 (17.5%) recipients had a relative difference lower than 50%.

Overall, recipients with a high overall number and value of payments usually had high absolute differences (web supplement 5). Relative differences were lower for recipients with higher overall number and value of payments.

The shares of recipients with matching numbers and values of payments showed no improvement over time, but the shares of those with relative differences lower than 50% increased (from 13.7% to 17.3% and from 9.7% to 14.5%, respectively) (web supplement 6).

For peer review only

Table 3 – Recipients – absolute and relative differences in the number and value of payments

	All years	%
**Calculations based on the number of payments - absolute differences**		
Number of patient organisations with at least one payment in at least one dataset - industry and patient organisation data	425	
Number of patient organisations with exact match in both datasets	33	7.8%
Number of patient organisations with more payments in industry data	335	78.8%
Number of patient organisations with more payments in patient organisation data	57	13.4%
Highest absolute difference between patient organisation and industry data - number of payments higher in industry data	199	
Highest absolute difference between patient organisation and industry data - number of payments higher in patient organisation data	33	
**Calculations based on the number of payments - relative differences**		
Number of patient organisations with relative difference <10%	35	8.2%
Number of patient organisations with relative difference <20%	42	9.9%
Number of patient organisations with relative difference <50%	81	19.1%
Number of patient organisations with relative difference = 100%	225	52.9%
**Calculations based on the value of payments - absolute differences**		
Number of patient organisations with at least one payment with value >£0 in at least one dataset	416	100%
Number of patient organisations with exact match in both datasets	8	1.9%
Number of patient organisations with higher payment value in industry data	356	85.6%
Number of patient organisations with higher payment value in patient organisation data	52	12.5%

For peer review only

Highest absolute difference between patient organisation and industry data - number of payments higher in industry data (2016 £)	6,718,576.6
Highest absolute difference between patient organisation and industry data - number of payments higher in patient organisation data (2016 £)	6,493,237.1

Calculations based on the value of payments - relative differences

Number of patient organisations with relative difference <10%	21	5.0%
Number of patient organisations with relative difference <20%	34	8.2%
Number of patient organisations with relative difference <50%	73	17.5%
Number of patient organisations with relative difference = 100%	295	70.9%

For peer review only

Missing payments

After analysing the pattern of absolute and relative differences we now focus on donors and recipients with the relative difference of 100%, that is, those with payments reported only in either industry or patient organisation data.

Industry data

There were 24 donors, representing 38.1% of those in industry data, which seemed not to comply with the ABPI Code[12] as their payments were reported exclusively in patient organisation data over the entire period of observation (web supplement 7, 8, 9). The number and value of payments associated with these donors (in patient organisation data) were 128 and £1,610,321.1, equivalent to 3% of the number and value of payments in industry data. The annual shares of missing donors and their payments were considerably higher as some companies did not report payments only in some years. The annual shares of missing payments were decreasing over time.

Patient organisation data

There were 3 donors, constituting 3.6% of those in patient organisation data, whose payments were only reported in industry data (web supplement 10, 11, 12). The number and value of payments associated with these donors (in industry data) were 9 and £92.208.2, constituting 0.5% and 0.3% of the number and value of payments in patient organisation data. As with industry data, the annual shares of missing donors and their payments were higher as some donors were not reported in patient organisation data only in some years. The annual shares of missing payments were decreasing during the study period.

There were 225 recipients missing from patient organisation data, representing 112.5% of recipients in this dataset (web supplement 13, 14, 15). The number and value of missing payments were 1,472 and £14,023,475.41 (88.6% and 42.4% of the respective figures in patient organisation data). The yearly share of missing recipients and their payments increased, while the yearly share of missing payment values decreased.

Agreement between donor-recipient ties reported by industry and patient organisations

After describing the underreporting as a donor/recipient characteristic we now consider it by examining the distribution of ties – or connections – between donors and recipients in each dataset

There were 1101 ties between donors and recipients formed by at least one payment in industry data and 626 in patient organisation data (Table 4). In 428 instances both datasets agreed about the donors and recipients, representing 38.9% and 68.4% of ties in each dataset, respectively. Of these, in 87 (20.3%) instances both donors and recipients reported the same number of payments. The number of payments constituting those matching ties

For peer review only

was 162, equivalent to 3.8% and 9.8% of the total in industry and patient organisation data, respectively.

There were 1,088 ties involving payments with a value greater than zero in industry data and 326 in patient organisation data. In 34 instances, both datasets agreed about donors and recipients (3.1% and 10.4% of ties in each dataset). Of these, in 20 instances (58.8%) instances both sides provided matching payment values. The ties with matching values were worth £325,108.4, representing 0.6% and 1.0.% of the value of payments in industry and patient organisation data, respectively.

The proportion of shared ties remained stable across time (web supplement 16).

For peer review only

Table 4 – Shared ties in industry and patient organisation data

Ties calculated based on the number of payments	All years
Total number of ties reported in industry data involving at least one payment	1101
Total number of ties reported in patient organisation data involving at least one payment	626
Number of shared ties (donors and recipients are the same in industry and patient organisation data)	428
Shared ties as a share of all ties in industry data	38.9%
Shared ties as a share of all ties in patient organisation data	68.4%
Number of ties involving the same number of payments	87
Number of ties involving the same number of payments as a share of shared ties	20.3%
Number of payments forming ties with the equal number of payments in both datasets	162
Payments forming ties with the equal number of payments in both datasets as a a share of all payments in industry data	3.8%
Payments forming ties with the equal number of payments in both datasets as a a share of all payments in patient organisation data	9.8%
**Ties calculated based on the value of payments**	
Total number of ties reported in industry data involving at least one payment with value >£0	1088
Total number of ties reported in patient data involving at least one payment with value >£0	326
Number of shared ties (donors and recipients are the same in industry and patient organisation data)	34
Shared ties as a share of all ties in industry data	3.1%
Shared ties as a share of all ties in patient organisation data	10.4%
Number of shared ties involving the same value of payments	20
Shared ties involving the same value of payments as a share of shared ties	58.8%

For peer review only

Value of payments in which donors and recipients provided the same value (2016 £)	325,108.4
Value of shared payments as a share of payments with values >£0 in industry data	0.6%
Value of shared payments as a share of payments with values >£0 in patient organisation data	1.0%

For peer review only

For peer review only - http://bmjopen.bmj.com/site/about/guidelines.xhtml

Overlap between recipient and donor lists in the two datasets

Having compared the distribution of ties in each dataset, we now consider ties as a characteristic of donors and recipients. Specifically, we analyse underreporting by comparing donor and recipient lists in each dataset against the other dataset.

Figure 1 Overlap between recipient and donor lists

Overlap between recipient lists and patient organisation data

Of 63 donors reporting at least one payment in the industry dataset, only 3 (4.8%) had all their recipients mentioning them as donors, the situation representing the full overlap between recipient lists and patient organisation data (Figure 1A). Conversely, the recipient lists of 6 (9.5%) donors had zero overlap with patient organisation data. The recipient lists of the remaining donors were situated between the two extremes, with 40 (63.5%) donors having less than 50% overlap, meaning that only half of the recipients were reflected in patient organisation data.

Considering the recipient lists calculated using the value of payments (62 donors reporting payment values greater than zero), the full and zero overlap occurred for 2 (3.2%) and 17 (27.0%) donors, respectively (Figure 1B). Overall, 56 (90.3%) donors had less than 50% overlap with patient organisation data.

Overlap between donors lists and industry data

Of the 200 recipients in patient organisation data, 102 (51.0%) had all donors mentioning them as recipients, which represented the full overlap between donor lists and industry data (Figure 1C). Contrastingly, the donor lists of 14 (7.4%) recipients had no overlap with industry data. Overall, 32 (16.0%) recipients had less than 50% overlap with industry data.

Based on the value of payments (121 recipients reporting payment values greater than zero), the full and zero overlap occurred for 71 (58.7%) and 12 (9.9%) recipients, respectively (Figure 1D). There were 18 (14.9%) donors with less than 50% overlap with industry data.

Over time, there was little improvement in the share of patient organisations reporting funding from drug companies listing them as recipients (web supplement 17 and 18). On the other hand, donors were reporting an increasing share of patient organisations mentioning their funding (web supplement 19 and 20).

Discussion

Principal findings

For peer review only - http://bmjopen.bmj.com/site/about/guidelines.xhtml

Our study confirms that earlier concerns about the underreporting of payments in relation to specific drug companies[14] and charities[33 34] were not isolated instances. It demonstrates that neither the industry self-regulatory system for disclosure of payments to patient organisations[1] nor a state-run system built for broader financial transparency purposes succeeded in preventing a high extent of underreporting. Importantly, the underreporting occurred despite the increasing media and research salience of the transparency of financial relationships between the industry and patient organisations[18 35 36 37].

Although some yearly variation might be expected given, for example, differences between when a payment was made and when it was received or spent, the overall large and seemingly increasing discrepancies between industry and patient organisation records are concerning. The number and value of payments disclosed by drug companies were likely to be higher because the ABPI Code has a formal definition of a payment and stipulates that payment values should be recorded whenever possible.[12] Charity regulators lack similar specific requirements.[38] Further, some patient organisations may redistribute money to other collaborating entities and therefore they may report smaller amounts than donating drug companies.[39] Nevertheless, any differences between donor and recipient records should be minimised by the requirement for a written contract between the parties, introduced by the ABPI Code, implying a shared understanding of how much is paid, to whom, and how.[12]

What also indicates underreporting is that few companies and patient organisations had matching – or even broadly similar – records in the two datasets, with many having differences exceeding hundreds of payments or worth millions of pounds. Both drug companies and patient organisations had a larger number of payments in the other dataset, but for both sides the value of payments was greater in industry data. The contrast between the datasets was higher for patient organisations, with an overwhelming majority having a greater number and value of payments reported in industry data. This pattern suggests inadequacies of the existing governance of the reporting of corporate payments by the charity regulators. It further corresponds with the results of a journalistic investigation into the underreporting of payments by a major charity,[33 34] prompting a rebuke from a charity regulator.[40]

We also unearthed a number of unreported payments associated with companies missing from either dataset. The underreporting of payments is further indicated by the fact that ties – or donor-recipient connections – shared by both datasets were in minority. This was particularly the case for ties involving matching payment numbers or values. The extent of the overlap between donor and recipient lists reported in the two datasets was similarly limited. Overall, this means that donors and recipients rarely disclosed payments reported by the other side.

As drug companies from our sample were signatories of the ABPI Code, missing payments indicate that they either did not meet the obligation to disclose or met it but subsequently removed their online disclosure reports. We could not find any evidence of specific obligations for UK charities to name funders in their accounts.[38] Their absence is motivated by concerns that “the loss of donor anonymity would result in a decrease in voluntary income reported by charities. There were also concerns about the practical implications and

For peer review only

about how much interest 'general users' of charity accounts would have in this disclosure."[41] Nevertheless, any unreported payments could be misleading for patient organisation members, supporters, expert bodies relying on patient testimonies,[20] policymakers, and the public.[33 40] Unreported payments may sometimes indicate a culture characterised by corporate manipulation of patient organisations.[42 43] Indeed, an oft-used argument for increasing transparency of payments not only to patient organisations but also medical professionals and scientists is that "sunshine" mitigates against misbehaviour and undue influence.[44]

A similarly high extent of underreporting has been identified in other countries and other recipients of industry payments. For example, a study of the underreporting of conflicts of interest (COIs) in Danish clinical practice guidelines in 2012 identified that only one of the 45 surveyed guidelines disclosed author COIs.[22] A related Australian study found that about one in four guideline writers may have potentially relevant undisclosed ties to pharmaceutical companies.[23] Similarly, US studies have repeatedly found significant discrepancies between self-reported COIs by authors of scientific publications and the mandatory payment reports in the Open Payments database;[25 26 28] for example, a study of oncologist authors of clinical trial publications in high-impact journals established that one-third failed to completely disclose payments from the study sponsor.[26]

Strengths and limitations

This is the first study examining the underreporting of payments to patient organisations using large samples of donors and recipients over a period of time. It systematically compares the underreporting by the two sides using five complementary measures. These measures account for the varying nature of different types of payment data and therefore can be replicated in other countries.

Our study has several important limitations. First, while Disclosure UK covers a vast majority of the UK pharmaceutical industry,[13] our sample was not exhaustive, as demonstrated by additional companies found in patient organisation accounts. Second, excluding companies outside of our sample is likely to have underestimated the underreporting. However, several further rounds of data scrapping on industry and charity regulators' websites would be necessary to reach data saturation. Third, we identified patient organisations using drug company disclosure reports, but a sampling strategy starting from patient organisations could have led to different results. Fourth, patient organisations registered with charity regulators are not required to submit annual accounts if their yearly income is lower than £25,000; therefore we might have excluded, entirely or in specific years, some of the patient organisations identified as recipients of industry funding, if their yearly incomes were below that threshold. Fifth, some patient organisations were excluded due to the conversion of financial years into calendar years, which might have increased discrepancies with drug company disclosure reports. Sixth, some companies might have reported payments made in 2016 in subsequent years, but the extent of delayed reporting is likely to have been minimal.[1] We did not identify instances of delayed reporting in patient organisation annual accounts. Seventh, we only considered annual accounts submitted to charity regulators, while funders might have also (or instead) been disclosed on the charity's website. However, website disclosures would be likely to undergo frequent changes, and have often been

For peer review only - http://bmjopen.bmj.com/site/about/guidelines.xhtml

found to lack transparency.[15 16 17 18 19] Eight, evaluating the extent of underreporting precisely is impossible because no definitive list of payments exists; consequently, there could be payments undisclosed by both sides. Finally, considering payment descriptions could reveal further discrepancies, including different payment goals reported by donors and recipients; it could also identify payments made via third parties (e.g. public relations or consultancy companies[40]) or benefitting patient organisations indirectly.[14]

Conclusions and policy implications

Although the full scale of underreporting of industry payments to patient organisations remains unknown, it concerns both donors and recipients, and involves a considerable number and value of payments. Our findings put a question mark over the key claim that – at least in their current form – publicly available payment disclosures effectively address concerns about COIs resulting from industry payments.

Against this background, we provide evidence for developing easily achievable improvements in the reporting of payments by both donors and recipients. Drug companies should improve data presentation using recommendations already formulated,[14] especially standardisation of reporting and elimination of payments with no assigned values. Companies should store data for at least as long as is required by the Charity Commission, that is five years. Given the shortcomings of the industry self-regulatory system, charity regulators should introduce tailored solutions related to reporting corporate funding in annual accounts, including a standardised template comprising a short payment description (including payment form and goal), its value, and donor name. Patient organisation websites should report this information separately or include clear signposting to the annual accounts. However, only a single state-run permanent database integrating payments reported by drug companies and patient organisations could eliminate underreporting. Finally, any payment disclosure system must have built-in checks to ensure comprehensive disclosure.

For peer review only

References

1. Ozieranski P, Rickard E, Mulinari, Shai. Exposing drug industry funding of UK patient organisations. *BMJ* 2019;365:l1806. doi: 10.1136/bmj.l1806
2. Rose SL, Highland J, Karafa MT, et al. Patient advocacy organizations, industry funding, and conflicts of interest. *JAMA internal medicine* 2017;177(3):344-50.
3. Taylor J, Denegri S. Industry links with patient organisations. *BMJ* 2017
4. Kent A. Should patient groups accept money from drug companies? Yes. *BMJ* 2007;334(7600):934. doi: 10.1136/bmj.39185.461968.AD
5. Hemminki E, Toiviainen HK, Vuorenkoski L. Co-operation between patient organisations and the drug industry in Finland. *Social science & medicine* 2010;70(8):1171-75.
6. McCoy MS, Carniol M, Chockley K, et al. Conflicts of interest for patient-advocacy organizations. *New England Journal of Medicine* 2017;376(9):880-85.
7. Fabbri A, Swandari S, Lau E, et al. Pharmaceutical Industry Funding of Health Consumer Groups in Australia: A Cross-sectional Analysis. *International Journal of Health Services* 2019:0020731418823376.
8. Lexchin J. Association between commercial funding of Canadian patient groups and their views about funding of medicines: An observational study. *PLOS ONE* 2019;14(2):e0212399. doi: 10.1371/journal.pone.0212399
9. Kang S-Y, Bai G, Karas L, et al. Pharmaceutical Industry Support of US Patient Advocacy Organizations: An International Context. *American journal of public health* 2019;109(4):559-61.
10. Pearcy J. ABPI guidance on drug companies and patient organisations. *BMJ* 2019;364:l740. doi: 10.1136/bmj.l740
11. EFPIA. EFPIA Code of Practice on relationships between the pharmaceutical industry and Patient Organisations. 2011
12. ABPI. ABPI Code of Practice for the Pharmaceutical Industry. 2016
13. PMCPA. Annual report 2016. 2016
14. Rickard E, Ozieranski P, Mulinari S. Evaluating the transparency of pharmaceutical company disclosure of payments to patient organisations in the UK. *Health Policy* 2019
15. Mosconi P. Industry funding of patients' support groups: Declaration of competing interests is rare in Italian breast cancer associations. *BMJ: British Medical Journal* 2003;327(7410):344.
16. Ball DE, Tisocki K, Herxheimer A. Advertising and disclosure of funding on patient organisation websites: a cross-sectional survey. *BMC Public Health* 2006;6(1):201.
17. Rothman SM, Raveis VH, Friedman A, et al. Health advocacy organizations and the pharmaceutical industry: an analysis of disclosure practices. *American Journal of Public Health* 2011;101(4):602-09.
18. Colombo C, Mosconi P, Villani W, et al. Patient organizations’ funding from pharmaceutical companies: is disclosure clear, complete and accessible to the public? An Italian survey. *PLoS One* 2012;7(5):e34974.
19. Lau E, Fabbri A, Mintzes B. How do health consumer organisations in Australia manage pharmaceutical industry sponsorship? A cross-sectional study. *Australian Health Review* 2018

For peer review only - http://bmjopen.bmj.com/site/about/guidelines.xhtml

20. Mandeville KL, Barker R, Packham A, et al. Financial interests of patient organisations contributing to technology assessment at England's National Institute for Health and Care Excellence: policy review. *bmj* 2019;364:k5300.
21. Neuman J, Korenstein D, Ross JS, et al. Prevalence of financial conflicts of interest among panel members producing clinical practice guidelines in Canada and United States: cross sectional study. *BMJ* 2011;343:d5621. doi: 10.1136/bmj.d5621
22. Bindslev JBB, Schroll J, Gøtzsche PC, et al. Underreporting of conflicts of interest in clinical practice guidelines: cross sectional study. *BMC Medical Ethics* 2013;14(1):19. doi: 10.1186/1472-6939-14-19
23. Moynihan R, Lai A, Jarvis H, et al. Undisclosed financial ties between guideline writers and pharmaceutical companies: a cross-sectional study across 10 disease categories. *BMJ Open* 2019;9(2):e025864. doi: 10.1136/bmjopen-2018-025864
24. Rasmussen K, Schroll J, Gøtzsche PC, et al. Under-reporting of conflicts of interest among trialists: a cross-sectional study. *J R Soc Med* 2014;108 doi: 10.1177/0141076814557878
25. Olavarria OA, Holihan JL, Cherla D, et al. Comparison of Conflicts of Interest among Published Hernia Researchers Self-Reported with the Centers for Medicare and Medicaid Services Open Payments Database. *Journal of the American College of Surgeons* 2017;224(5):800-04. doi: https://doi.org/10.1016/j.jamcollsurg.2017.01.052
26. Wayant C, Turner E, Meyer C, et al. Financial conflicts of interest among oncologist authors of reports of clinical drug trials. *JAMA oncology* 2018;4(10):1426-28.
27. Kesselheim AS, Wang B, Studdert DM, et al. Conflict of Interest Reporting by Authors Involved in Promotion of Off-Label Drug Use: An Analysis of Journal Disclosures. *PLOS Medicine* 2012;9(8):e1001280. doi: 10.1371/journal.pmed.1001280
28. Cherla DV, Olavarria OA, Holihan JL, et al. Discordance of conflict of interest self-disclosure and the Centers of Medicare and Medicaid Services. *Journal of Surgical Research* 2017;218:18-22.
29. Moberly T. The pharma deals that CCGs fail to declare. *BMJ* 2018;360:j5915. doi: 10.1136/bmj.j5915
30. Moberly T. NHS joint working with industry is out of public sight. *BMJ* 2019;364:l1353. doi: 10.1136/bmj.l1353
31. Ozieranski P, Csanadi M, Rickard E, et al. Analysis of Pharmaceutical Industry Payments to UK Health Care Organizations in 2015. *JAMA Network Open* 2019;2(6):e196253-e53.
32. Mulinari S, Ozieranski P. Disclosure of payments by pharmaceutical companies to healthcare professionals in the UK: analysis of the Association of the British Pharmaceutical Industry's Disclosure UK database, 2015 and 2016 cohorts. *BMJ open* 2018;8(10):e023094.
33. Gregory A. Watchdog: patient charities must come clean about Big Pharma cash. *The Sunday Times* 2019.
34. Gregory A. Patients' charity Pain UK took cash from Big Pharma. *The Sunday Times* 2019.
35. Vitry A, Löfgren H. 16. Health consumer groups and the pharmaceutical industry: is transparency the answer? *Democratizing Health: Consumer groups in the policy process* 2011:239.
36. Rose SL. Patient advocacy organizations: institutional conflicts of interest, trust, and trustworthiness: SAGE Publications Sage CA: Los Angeles, CA, 2013.

For peer review only

37. Moynihan R, Bero L. Toward a Healthier Patient Voice: More Independence, Less Industry Funding. *JAMA internal medicine* 2017;177(3):350-51.
38. Charity Commission. Charities SORP (FRS 102) Accounting and Reporting by Charities: Statement of Recommended Practice. 2014
39. Ridley SH. Rapid response to: Exposing drug industry funding of UK patient organisations. *BMJ* 2019;365:l1806.
40. Charity Commission. Pain UK: regulatory compliance case conclusions. 2019
41. Jones G. Charities will not have to name donors, says SORP Committee. *Civil Society News* 2017.
42. Kanter GP. Extending the Sunshine Act From Physicians to Patient Advocacy Organizations. *American Journal of Public Health* 2018;108(8):978-79. doi: 10.2105/AJPH.2018.304520
43. Lin DH, Lucas E, Murimi IB, et al. Financial Conflicts of Interest and the Centers for Disease Control and Prevention's 2016 Guideline for Prescribing Opioids for Chronic Pain. *JAMA Internal Medicine* 2017;177(3):427-28. doi: 10.1001/jamainternmed.2016.8471
44. Rosenthal MB, Mello MM. Sunlight as Disinfectant — New Rules on Disclosure of Industry Payments to Physicians. *New England Journal of Medicine* 2013;368(22):2052-54. doi: 10.1056/NEJMp1305090

For peer review only

Figure 1

The bars in Figures 1A-1B show the number of donors with varying level of overlap between their recipient lists and patient organisation data based on the number (Figure 1A) and value (Figure 1B) of payments. For example, based on the number of payments there were 13 companies with 20-29.9% recipients also being reported in patient organisation data (Figure 1A).

The bars in Figures 1C-1D show the number of recipients with varying level of overlap between their donor lists and industry data based on the number (Figure 1C) and value (Figure 1D) of payments. For example, based on the number of payments there were 24 recipients with 50-59.9% of donors also being reported in industry data.

For peer review only

Contributors

PO conceived and designed the study, managed, analysed and interpreted the data, as well as drafted the article MC analysed the data and drafted the article. ER collected and managed the data, and drafted the article. SM designed the study, contributed to interpretation, drafted the article

Funding

Shai Mulinari and Piotr Ozieranski's work on this study was supported by the grant "What can be learnt from the new pharmaceutical industry payment disclosures?" awarded by the Swedish Research Council for Health, Working Life and Welfare (FORTE #2016-00875). Emily Rickard's work was funded through the above grant. Marcell Csanadi did not receive funding for this work.

Details of the role of study sponsors

The funding body has played no part in the design or conduct of this study

Statement of independence of researchers from funders

All researchers working on this study are independent from the funding body.

Competing interests

We have read and understood the BMJ Group policy on declaration of interests and declare the following interests:

Shai Mulinari and Piotr Ozieranski's work on the study reported in this paper was supported by the grant "What can be learnt from the new pharmaceutical industry payment disclosures?" awarded by the Swedish Research Council for Health, Working Life and Welfare (FORTE #2016-00875). Emily Rickard's work was funded through the above grant. Marcell Csanadi did not receive funding for this work. We have no other competing interests to declare.

For peer review only

Patient consent

None required.

Ethical approval

No ethical approval was needed.

Data sharing

The authors of this study agree to share data underpinning this study in the form of web supplements. The raw data poses no risk to anonymity of individuals as it draws on publicly available reports concerning financial transfers between organisations.

Exclusive license

The Corresponding Author has the right to grant on behalf of all authors and does grant on behalf of all authors, a worldwide licence to the Publishers and its licensees in perpetuity, in all forms, formats and media (whether known now or created in the future), to i) publish, reproduce, distribute, display and store the Contribution, ii) translate the Contribution into other languages, create adaptations, reprints, include within collections and create summaries, extracts and/or, abstracts of the Contribution, iii) create any other derivative work(s) based on the Contribution, iv) to exploit all subsidiary rights in the Contribution, v) the inclusion of electronic links from the Contribution to third party material where-ever it may be located; and, vi) licence any third party to do any or all of the above."

Transparency declaration

The guarantor (Piotr Ozieranski) affirms that this manuscript is an honest, accurate, and transparent account of the study being reported; that no important aspects of the study have been omitted; and that any discrepancies from the study as planned (and, if relevant, registered) have been explained.

For peer review only

Figure 1. Overlap between recipient and donor lists

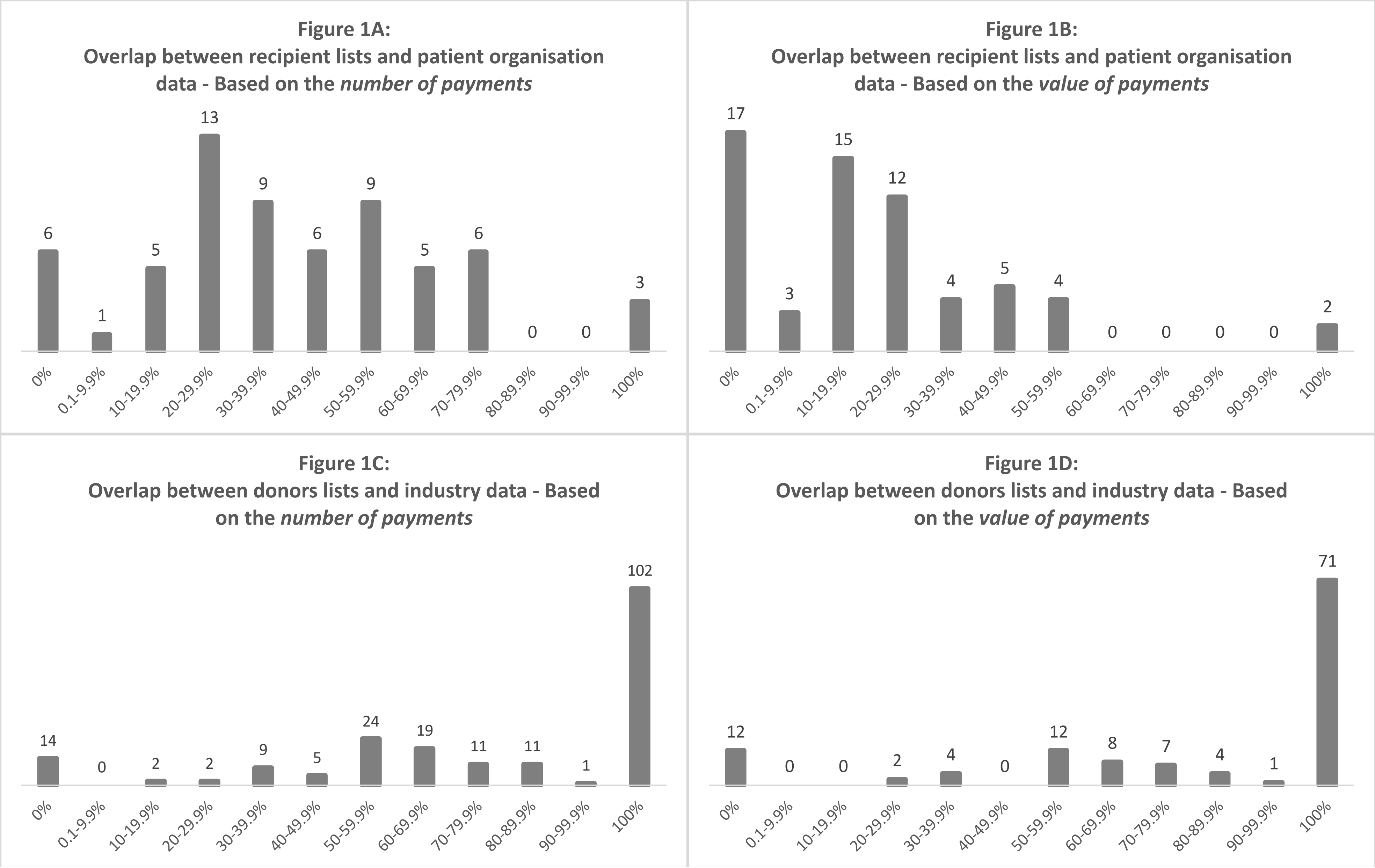

Web supplement 1 – Research process: pharmaceutical industry data

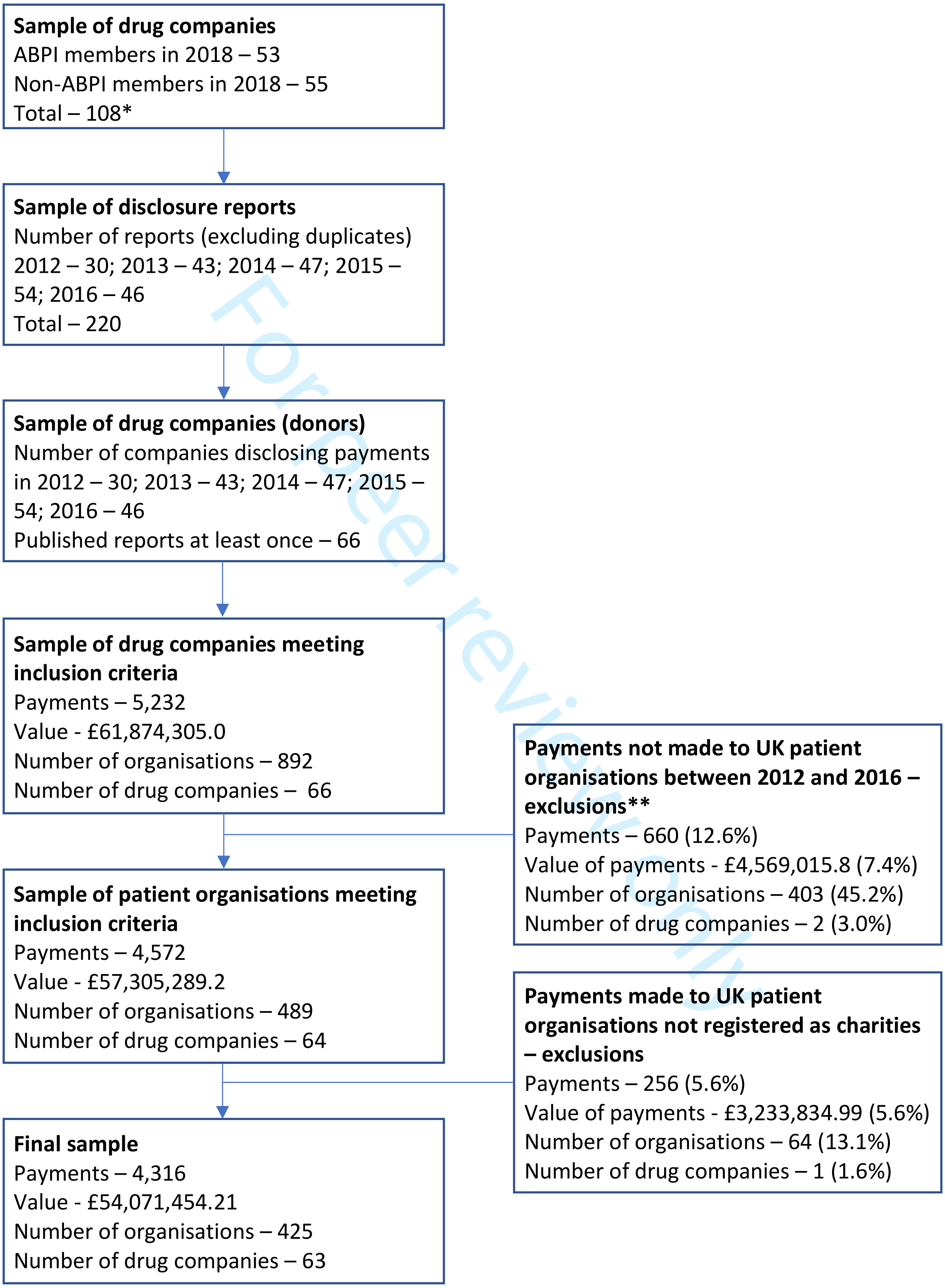

*Complete lists of companies provided in Table 1

**Detailed breakdown provided in Table 2

For peer review only

Table 1. Sample of drug companies – ABPI and non-ABPI members

ABPI member in 2018 – n=53	Non-ABPI member in 2018 – n=55
A. Menarini	Accretio
Abbvie	Actavis
Actelion	Aegerion
Alexion	Aguettant
Alimera Sciences	Alcon
ALK-Abelo	AMCo
Allergan	ApoPharma
Alliance	Astellas
Almirall	Baxalta
Amgen	Baxter
AstraZeneca	Besins
Bausch and Lomb	BGP Products
Bayer	Bio Products Laboratory
Biogen	Biotest
BioMarin	Bracco
Bristol-Myers Squibb	Britannia
Boehringer Ingelheim	CEB Pharma
Celgene	Consilient Health
Chiesi	CSL Behring
Chugai	Dermal
Daiichi Sankyo	Diurnal
Eisai	Ferring
Lilly	Flynn Pharma
Fresenius Medical Care	Fresenius Kabi
Grünenthal	Galen
GSK	Gedeon Richter UK
Ipsen	Genzyme Therapeutics
Janssen	Gilead
LEO Pharma	Guerbet
Lundbeck	Hospira UK
Merck	HRA Pharma
Mitsubishi Tanabe Pharma	Jazz Pharmaceuticals
MSD	Martindale Pharma
Napp Pharmaceuticals	Meda Pharmaceuticals
Norgine	Merz Pharma
Novartis	Mundipharma International
Novo Nordisk	Nicovations
Orion Pharma	Octapharma
Otsuka Pharmaceuticals	Orphan Europe
Pfizer	Profile Pharma

For peer review only

Pharma Mar	PTC Therapeutics
Pierre Fabre Ltd	RB
Quintiles Commercial (Novex)	Rosemont Pharmaceuticals
Roche Products	Sandoz
Sanofi	Sanofi Pasteur
Santen	Sigma Tau Rare Diseases
Servier Laboratories	Sobi
Shionogi Europe	Special Products
Shire	STD
Sunovion Pharmaceuticals	Stirling Anglian
Takeda	Syner-Med
UCB	Teva
Vifor Fresenius Medical Care Renal Pharma	Thea Pharmaceuticals
	Tillotts
	Vifor Pharma Group

Table 2. Breakdown of exclusions - payments not made to UK patient organisations between 2012 and 2016

Reason for exclusion	Number of payments (%)	Value of payments - £ (%)	Number of organisations (%)
Outside period of observation	4 (0.6)	10,039.8 (0.2)	0 (0.0)
Not an organisation	14 (2.1)	94,736.1 (2.1)	0 (0.0)
More than one organisation	17 (2.6)	354,390.0 (7.8)	0 (0.0)
Organisation - nature unclear	57 (8.6)	397,240.6 (8.7)	39 (9.7)
Not UK organisation	54 (8.2)	716,044.0 (15.7)	30 (7.4)
Excluded UK organisations			
Public sector – state organisation	12 (1.8)	18,629.7 (0.4)	7 (1.7)
Public healthcare provider	242 (36.7)	726,401.4 (15.9)	176 (43.7)
Private sector organisation	47 (7.1)	981,886.2 (21.5)	33 (8.2)
University	14 (2.1)	86,670.3 (1.9)	7 (1.7)
Professional organisation (third sector)	143 (21.7)	984,935.1 (21.6)	73 (18.1)
Community interest company or social enterprise - healthcare provider (third sector)	3 (0.5)	2,690.7 (0.1)	3 (0.7)
Healthcare provider (third sector)	20 (3.0)	46,522.0 (1.0)	10 (2.5)
Not focusing directly on health (third sector)	30 (4.5)	148,396.5 (3.2)	24 (6.0)
Community interest company or social enterprise - not focusing directly on health (third sector)	3 (0.5)	433.4 (0.0)	1 (0.2)
Total	660 (100)	4,569,015.8	403 (100)

For peer review only

Web supplement 2 Research process: patient organisation data

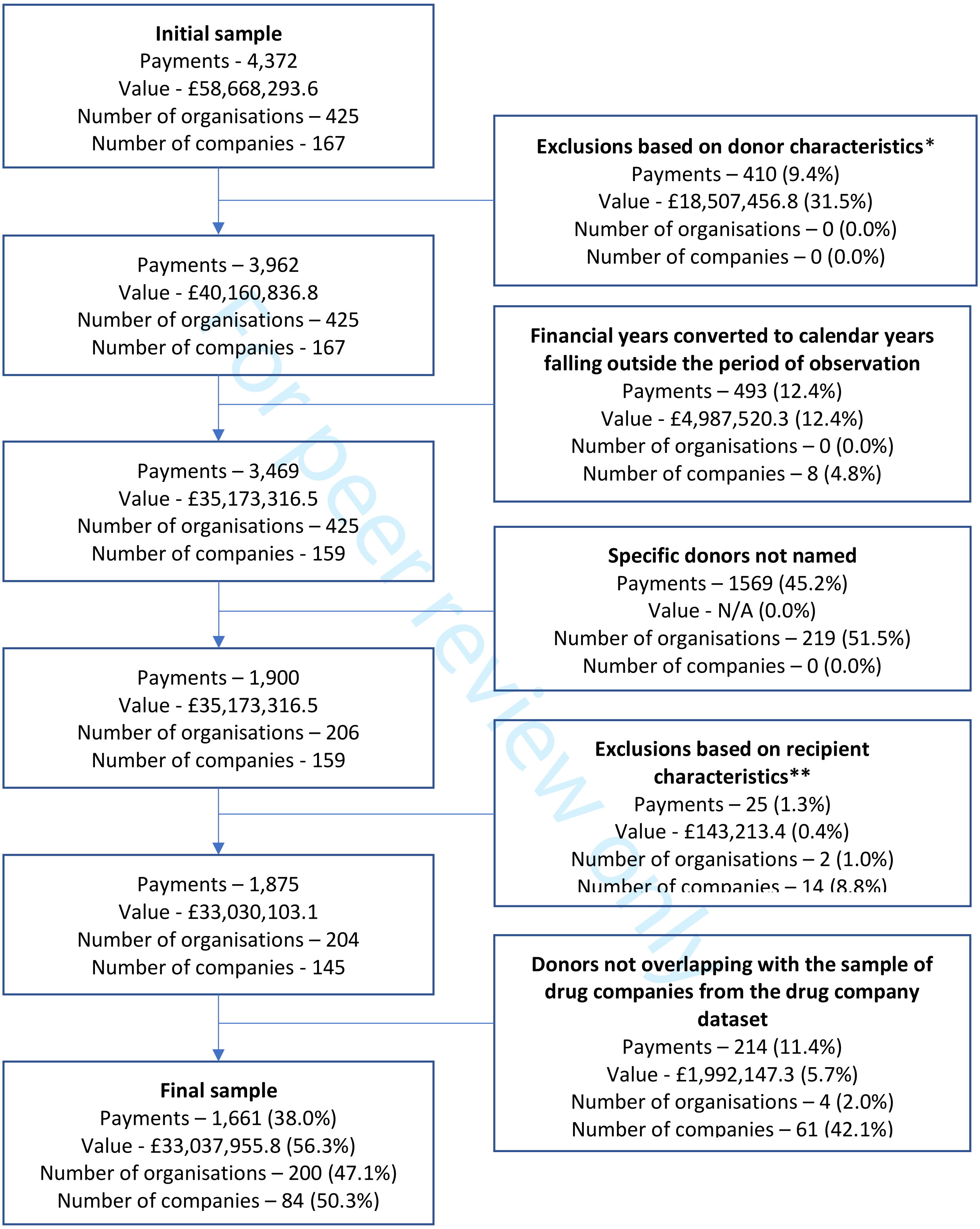

*More detailed breakdown in Table 3
**More detailed breakdown in Table 4

For peer review only

Table 3. Exclusions based on donor characteristics – detailed breakdown

Reason for exclusion	Number of Payments excluded	Value of payments excluded (£)
Exclusions unique to patient organisation dataset		
Regional branch (same charity number as national branch)	229	13,213,514.2
Subsumed under a larger organisation (same charity number as larger organisation)	125	291,862.9
Years before merger	14	1,515.0
Years after merger	9	-
Exclusions resulting from exclusions in drug company dataset		
Third sector - healthcare provider	6	-
Third sector - professional organisation	6	-
Third sector - not focusing directly on health	15	5,000,564.8
Not a charity	6	-
Total	410	18,507,456.8

Table 4. Exclusions based on recipient characteristics – detailed breakdown

	Number of Payments excluded	Value of payments excluded (£)	Number of drug companies excluded	Number of patient organisations excluded
More than one organisation	8	58,485.5	7	0
Not a pharmaceutical company	17	84,727.8	7	2
Total	25	143,213.4	14	2

For peer review only

Web Supplement 3. Donors - Absolute and relative differences in the number and value of payment

NUMBER OF PAYMENTS

Number of payments - industry data
Number of payments - patient organisation data
Number of drug companies with at least one payment - industry data
Number of drug companies with at least one payment - patient organisation data

NUMBER OF PAYMENTS - THRESHOLDS

Threshold 0: Number of drug companies with at least 1 one payment in at least one dataset (1 if cond
Threshold 1: Number of drug companies with more than 1 one payment in at least one dataset (1 if co
Threshold 2: Number of drug companies with more than 10 payments in at least one dataset (1 if con
Threshold 3: Number of drug companies with more than 100 payments in at least one dataset (1 if co

NUMBER OF PAYMENTS - ABSOLUTE DIFFERENCES

Number of payments - threshold 0
Number of drug companies with exact match in both datasets
Number of drug companies with more paymens t in industry data
Number of drug companies with more payments in patient organisation data

Highest absolute difference between patient organisation and industry data - number of payments hi
Highest absolute difference between patient organisation and industry data - number of payments hi

Number of payments - threshold 1
Number of drug companies with exact match in both datasets
Number of drug companies with more paymens t in industry data
Number of drug companies with more payments in patient organisation data

Highest absolute difference between patient organisation and industry data - number of payments hi
Highest absolute difference between patient organisation and industry data - number of payments hi

Number of payments - threshold 2
Number of drug companies with exact match in both datasets
Number of drug companies with more paymens t in industry data
Number of drug companies with more payments in patient organisation data

Highest absolute difference between patient organisation and industry data - number of payments hi
Highest absolute difference between patient organisation and industry data - number of payments hi

Number of payments - threshold 3

For peer review only

Number of drug companies with exact match in both datasets
Number of drug companies with more paymens t in industry data
Number of drug companies with more payments in patient organisation data

Highest absolute difference between patient organisation and industry data - number of payments hi
Highest absolute difference between patient organisation and industry data - number of payments hi

NUMBER OF PAYMENTS - RELATIVE DIFFERENCES

Relative difference <10%
Relative difference <20%
Relative difference <50%
Relative difference = 100%

Number of payments - threshold 1
Relative difference <10%
Relative difference <20%
Relative difference <50%
Relative difference = 100%

Number of payments - threshold 2
Relative difference <10%
Relative difference <20%
Relative difference <50%
Relative difference = 100%

Number of payments - threshold 3
Relative difference <10%
Relative difference <20%
Relative difference <50%
Relative difference = 100%

VALUE OF PAYMENTS - ABSOLUTE DIFFERENCES

Value of payments - industry data (2016 £)
Value of payments - patient organisation data (2016 £)
Number of payments with value >£0 - industry data
Number of payments with value >£0 - patient organisation data
Number of drug companies with at least one payment with the value >£0 - industry data
Number of drug companies with at least one payment with the value >£0 - patient organisation data

VALUE OF PAYMENTS - THRESHOLDS

Threshold 1: Number of drug companies with at least one payment with value >£0 in at least one data
Threshold 2: Number of drug companies with payments with the value of more than £10,000 in at lea

For peer review only

Threshold 3: Number of drug companies with payments with the value of more than £100,000 in at le

VALUE OF PAYMENTS - ABSOLUTE DIFFERENCES

Number of payments - threshold 1

Number of drug companies with exact match in both datasets
Number of drug companies with higher payment value in industry data
Number of drug companies with higher payment value in patient organisation data

Highest absolute difference between patient organisation and industry data - number of payments hi
Highest absolute difference between patient organisation and industry data - number of payments hi

Number of payments - threshold 2

Number of drug companies with exact match in both datasets
Number of drug companies with higher payment value in industry data
Number of drug companies with higher payment value in patient organisation data

Highest absolute difference between patient organisation and industry data - number of payments hi
Highest absolute difference between patient organisation and industry data - number of payments hi

Number of payments - threshold 3

Number of drug companies with exact match in both datasets
Number of drug companies with higher payment value in industry data
Number of drug companies with higher payment value in patient organisation data

Highest absolute difference between patient organisation and industry data - number of payments hi
Highest absolute difference between patient organisation and industry data - number of payments hi

VALUE OF PAYMENTS - RELATIVE DIFFERENCES

Relative difference <10%
Relative difference <20%
Relative difference <50%
Relative difference = 100%

Number of payments - threshold 1

Relative difference <10%
Relative difference <20%
Relative difference <50%
Relative difference = 100%

Number of payments - threshold 2

Relative difference <10%
Relative difference <20%

For peer review only

Relative difference <50%

Relative difference = 100%

Number of payments - threshold 3

Relative difference <10%

Relative difference <20%

Relative difference <50%

Relative difference = 100%

For peer review only

			A. Menarini	Abbvie	Actavis	Actelion
			1	1	0	1
Total						
	4316		3	281	5	38
	1661		0	84	5	23
	63		1	1	1	1
	84		0	1	1	1
Number of drug cc	% of all drug companies with at least one payment in at least one dataset					
	87	100.0%	1	1	1	1
	80	92.0%	1	1	1	1
	47	54.0%		1		1
	10	11.5%		1		
Number of drug cc	% of all drug companies at a given threshold					
	3	3.4%			1	
	49	56.3%	1	1		1
	35	40.2%				
Number of payments						
	757		3	197		15
	53					
Number of drug cc	% of all drug companies at a given threshold					
	3	3.8%			1	
	49	61.3%	1	1		1
	28	35.0%				
Number of payments						
	757		3	197		15
	53					
Number of drug cc	% of all drug companies at a given threshold					
	1	2.1%				
	36	76.6%		1		1
	10	21.3%				
Number of payments						
	757			197		15
	53					
Number of drug cc	% of all drug companies at a given threshold					

For peer review only

0	0.0%				
10	100.0%		1		
0	0.0%				
Number of payments					
757			197		
0					
Number of drug cc	% of all drug companies				
5	5.7%			0.0%	
9	10.3%			0.0%	
21	24.1%			0.0%	39.5%
27	31.0%	100.0%			
Number of drug cc	% of all companies at a given threshold				
5	6.3%			0.0%	
9	11.3%			0.0%	
21	26.3%			0.0%	39.5%
20	25.0%	100.0%			
Number of drug cc	% of all companies at a given threshold				
3	6.4%				
5	10.6%				
15	31.9%				39.5%
2	4.3%				
Number of drug cc	% of all companies at a given threshold				
0	0.0%				
0	0.0%				
1	10.0%				
0	0.0%				
Total					
£54,071,454.2		33,907.5	3,584,604.6	49,000.0	364,970.8
£33,037,955.8		-	1,412,340.9	49,240.0	217,382.1
4235		3	281	5	38
772		0	40	2	16
62		1	1	1	1
62		0	1	1	1
Number of drug cc	% of all drug companies with at least one payment with the value of at least				
74	85.1%	1	1	1	1
45	51.7%		1		1

For peer review only

17	19.5%		1		
Number of drug cc	% of all drug companies at a given threshold				
0	0.0%				
48	64.9%	1	1		1
26	35.1%			1	
Vallue of payments					
6,406,351.1		33908	2172264		147589
2,960,716.0				240	
Number of drug cc	% of all drug companies at a given threshold				
0	0.0%				
32	71.1%		1		1
13	28.9%				
Vallue of payments					
6,406,351.1			2172264		147589
2,960,716.0					
Number of drug cc	% of all drug companies at a given threshold				
0	0.0%				
13	76.5%		1		
4	23.5%				
Vallue of payments			0		
6,406,351.1			2172264		
2,960,716.0					
Number of drug cc	% of all drug companies with at least one payment with value >£0 in at least				
3	4.1%			0.5%	
9	12.2%			0.5%	
14	18.9%			0.5%	40.4%
24	32.4%	100.0%			
Number of drug cc	% of all drug companies at a given threshold				
3	4.1%			0.5%	
9	12.2%			0.5%	
14	18.9%			0.5%	40.4%
24	32.4%	100.0%			
Number of drug cc	% of all drug companies at a given threshold				
1	2.2%				
5	11.1%				

For peer review only

9	20.0%	40.4%
4	8.9%	

Number of drug cc	% of all drug companies at a given threshold
0	0.0%
2	11.8%
2	11.8%
1	5.9%

For peer review only

Aegerion	Alcon	Alexion	ALK-Abello	Allergan	Alliance	Almirall
0	0	1	1	1	1	1
5	1	5	0	25	0	9
4	9	17	2	26	1	2
1	1	1	0	1	0	1
1	1	1	1	1	1	1
1	1	1	1	1	1	1
1	1	1	1	1		1
		1		1		
1						1
	1	1	1	1	1	
1						7
	8	12	2	1	1	
1						1
	1	1	1	1		
1						7
	8	12	2	1		
		1		1		
		12		1		

For peer review only

				3.8%		
				3.8%		
20.0%				3.8%		
			100.0%		100.0%	
				3.8%		
				3.8%		
20.0%				3.8%		
			100.0%			
				3.8%		
				3.8%		
				3.8%		
35,040.1	15,150.0	60,600.0	-	678,030.8	-	62,968.9
-	83,733.8	70,583.4	-	148,240.9	-	-
5	1	5	0	25	0	9
0	5	8	0	4	0	0
1	1	1	0	1	0	1
0	1	1	0	1	0	0

t >£0 in at least one dataset

1	1	1		1		1
				1		

For peer review only

1			1	1
	1	1		
35040			529790	62969
	68584	9983		
			1	
			529790	
t one dataset				
		14.1%		
		14.1%		
100.0%				100.0%
		14.1%		
		14.1%		
100.0%				100.0%

For peer review only

For peer review only

Amdipharm	Amgen	ApoPharma	Astellas	AstraZeneca	ausch and Lom
0	1	0	0	1	1
0	82	0	74	55	0
4	41	5	8	24	4
0	1	0	1	1	0
1	1	1	1	1	1
1	1	1	1	1	1
1	1	1	1	1	1
	1		1	1	
	1		1	1	
1		1			1
	41		66	31	
4		5			4
	1		1	1	
1		1			1
	41		66	31	
4		5			4
	1		1	1	
	41		66	31	

For peer review only

100.0%		100.0%			100.0%
100.0%		100.0%			100.0%
-	699,274.6	-	2,783,709.6	725,306.3	-
34,433.3	324,720.9	76,194.8	80,417.4	42,450.6	22,627.3
0	82	0	74	53	0
3	22	5	4	6	2
0	1	0	1	1	0
1	1	1	1	1	1
1	1	1	1	1	1
	1		1	1	

For peer review only

			1		
	1		1	1	
1		1			1
	374554		2703292	682856	
34433		76195			22627
	1		1	1	
	374554		2703292	682856	
			1		
			0		
			2703292		
100.0%		100.0%			100.0%
100.0%		100.0%			100.0%

For peer review only

For peer review only

Baxalta	Baxter	Bayer	roducts Labor	Biogen	BioMarin
0	0	1	0	1	1
4	4	49	7	50	42
3	15	30	6	24	16
1	1	1	1	1	1
1	1	1	1	1	1
1	1	1	1	1	1
1	1	1	1	1	1
	1	1		1	1
1		1	1	1	1
	1				
1		19	1	26	26
	11				
1		1	1	1	1
	1				
1		19	1	26	26
	11				
		1		1	1
	1				
		19		26	26
	11				

For peer review only

			14.3%		
25.0%		38.8%	14.3%		
			14.3%		
25.0%		38.8%	14.3%		
		38.8%			
39,289.0	22,142.2	675,838.0	37,492.0	566,033.3	575,345.3
129,431.5	411,356.2	583,949.1	32,172.7	676,372.1	11,685.3
4	4	49	7	50	42
1	10	18	4	15	3
1	1	1	1	1	1
1	1	1	1	1	1
1	1	1	1	1	1
1	1	1		1	1

For peer review only

1	1	1	1	1	1
90143	389214	91889	5319	110339	563660
1	1	1		1	1
90143	389214	91889		110339	563660
		13.6%	14.2%	16.3%	
		13.6%	14.2%	16.3%	
		13.6%	14.2%	16.3%	
		13.6%	14.2%	16.3%	
		13.6%		16.3%	

For peer review only

13.6%	16.3%

For peer review only

Biotest	ehringer Ingelh	istol-Myers Squi	Britannia	Celgene	Chiesi
0	1	1	0	1	1
15	15	231	0	125	19
8	15	27	4	27	9
1	1	1	0	1	1
1	1	1	1	1	1
1	1	1	1	1	1
1	1	1	1	1	1
1	1	1		1	1
		1		1	
	1				
1		1		1	1
			1		
7		204		98	10
			4		
	1				
1		1		1	1
			1		
7		204		98	10
			4		
	1				
1		1		1	1
7		204		98	10

For peer review only

		1		1	
		204		98	
	0.0%				
	0.0%				
46.7%	0.0%				
			100.0%		
	0.0%				
	0.0%				
46.7%	0.0%				
			100.0%		
	0.0%				
	0.0%				
46.7%	0.0%				
88,420.4	133,153.7	1,759,705.1	-	1,828,014.0	188,262.1
131,337.0	134,736.3	281,373.4	3,884.0	493,270.0	57,591.4
15	15	230	0	125	19
7	7	17	1	11	2
1	1	1	0	1	1
1	1	1	1	1	1
1	1	1	1	1	1
1	1	1		1	1

For peer review only

		1		1	
		1		1	1
1	1		1		
		1478332		1334744	130671
42917	1583		3884		
		1		1	1
1	1				
		1478332		1334744	130671
42917	1583				
		1		1	
		0		0	
		1478332		1334744	
	1.2%				
	1.2%				
32.7%	1.2%				
			100.0%		
	1.2%				
	1.2%				
32.7%	1.2%				
			100.0%		
	1.2%				
	1.2%				

For peer review only

32.7%	1.2%

For peer review only

Chugai	onsilient Healt	CSL Behring	Daiichi Sankyo	Dermal	Diurnal
1	0	0	1	0	0
10	4	33	2	0	0
3	0	19	0	13	1
1	1	1	1	0	0
1	0	1	0	1	1
1	1	1	1	1	1
1	1	1	1	1	
		1		1	
1	1	1	1	1	1
7	4	14	2	13	1
1	1	1	1	1	
7	4	14	2	13	
		1		1	
		14		13	

For peer review only

		42.4%			
	100.0%		100.0%	100.0%	100.0%
		42.4%			
	100.0%		100.0%	100.0%	
		42.4%			
				100.0%	
19,784.9	41,467.4	627,645.1	16,833.3	-	-
-	-	501,753.7	-	-	-
9	4	33	2	0	0
0	0	13	0	0	0
1	1	1	1	0	0
0	0	1	0	0	0
1	1	1	1		
		1			

For peer review only

1	1	1	1
19785	41467	125891	16833
		1	
		125891	
		20.1%	
100.0%	100.0%		100.0%
		20.1%	
100.0%	100.0%		100.0%

For peer review only

20.1%

For peer review only

Eisai	Ferring	Flynn Pharma	enius Medical	Gedeon Richter	Genzyme
1	0	0	1	0	0
27	23	19	0	0	0
7	29	4	3	2	52
1	1	1	0	0	0
1	1	1	1	1	1
1	1	1	1	1	1
1	1	1	1	1	1
1	1	1			1
1		1			
	1		1	1	1
20		15			
	6		3	2	52
1		1			
	1		1	1	1
20		15			
	6		3	2	52
1		1			
	1				1
20		15			
	6				52

For peer review only

For peer review only

	20.7%				
			100.0%	100.0%	100.0%
	20.7%				
			100.0%	100.0%	100.0%
	20.7%				
					100.0%
187,422.6	180,131.8	38,577.9	-	-	-
-	44,886.0	5,175.2	15,940.6	-	1,225,894.6
27	23	19	0	0	0
0	3	3	3	0	25
1	1	1	0	0	0
0	1	1	1	0	1
1	1	1	1		1
1	1				1

				1
1	1	1		
			1	1
187423	135246	33403		
			15941	1225895
1	1			
				1
187423	135246			
				1225895
				1
				0
				1225895
100.0%			100.0%	100.0%
100.0%			100.0%	100.0%

For peer review only

100.0% 100.0%

100.0%

For peer review only

Drug company names					
Gilead	Grünenthal	GSK	HRA Pharma	Ipsen	IQVIA
0	1	1	0	1	1
174	17	20	6	5	0
90	3	73	3	11	1
1	1	1	1	1	0
1	1	1	1	1	1
1	1	1	1	1	1
1	1	1	1	1	
1	1	1		1	
1					
1	1		1		
		1		1	1
84	14		3		
		53		6	1
1	1		1		
		1		1	
84	14		3		
		53		6	
1	1				
		1		1	
84	14				
		53		6	

For peer review only

1					
84					
48.3%					
					100.0%
48.3%					
48.3%					
48.3%					
1,616,738.8	63,036.7	501,099.7	92,429.9	37,342.0	-
1,306,716.1	8,728.1	3,461,815.7	-	148,100.2	-
174	17	20	6	5	0
69	2	36	0	7	0
1	1	1	1	1	0
1	1	1	0	1	0
1	1	1	1	1	
1		1		1	

For peer review only

1		1		
1	1		1	
		1		1
310023	54309		92430	
		2960716		110758
1				
		1		1
310023				
		2960716		110758
1				
		1		
0		0		
310023				
		2960716		
19.2%				
19.2%				
			100.0%	
19.2%				
19.2%				
			100.0%	
19.2%				

For peer review only

19.2%

19.2%

19.2%

For peer review only

Janssen	Jazz	LEO Pharma	Lilly	Lundbeck	Meda
1	0	1	1	1	0
88	0	35	239	33	12
63	1	20	32	3	4
1	0	1	1	1	1
1	1	1	1	1	1
1	1	1	1	1	1
1		1	1	1	1
1		1	1	1	1
			1		
1		1	1	1	1
	1				
25		15	207	30	8
	1				
1		1	1	1	1
25		15	207	30	8
1		1	1	1	1
25		15	207	30	8

For peer review only

			1		
			207		
28.4%		42.9%			
	100.0%				
28.4%		42.9%			
28.4%		42.9%			
1,017,042.6	-	151,378.2	2,825,173.2	223,802.5	77,182.6
376,373.5	-	10,000.0	143,038.3	-	75,750.0
88	0	34	237	33	12
25	0	1	12	0	1
1	0	1	1	1	1
1	0	1	1	0	1
1		1	1	1	1
1		1	1	1	

For peer review only

1		1		
1	1	1	1	1
640669	141378	2682135	223803	1433
1	1	1	1	
640669	141378	2682135	223803	
1		1		
0		0		
640669		2682135		
				1.9%
				1.9%
				1.9%
			100.0%	
				1.9%
				1.9%
				1.9%
			100.0%	

For peer review only

100.0%

For peer review only

Merck	ishi Tanabe P	MSD	Mylan	p Pharmaceuti	Norgine
1	1	1	0	1	1
50	0	235	0	60	10
46	1	55	1	24	10
1	0	1	0	1	1
1	1	1	1	1	1
1	1	1	1	1	1
1		1		1	1
1		1		1	
		1			
					1
1		1		1	
	1		1		
4		180		36	
	1		1		
					1
1		1		1	
4		180		36	
1		1		1	
4		180		36	

For peer review only

		1			
		180			
8.0%					0.0%
8.0%					0.0%
8.0%					0.0%
	100.0%		100.0%		
8.0%					0.0%
8.0%					0.0%
8.0%					0.0%
8.0%					
8.0%					
8.0%					
455,388.5	-	1,750,167.8	-	173,152.9	102,297.0
905,077.7	5,070.3	837,180.0	-	82,633.4	33,700.5
50	0	233	0	60	10
19	1	29	0	10	2
1	0	1	0	1	1
1	1	1	0	1	1
1	1	1		1	1
1		1		1	1

For peer review only

		1		
		1	1	1
1	1			
		912988	90519	68596
449689	5070			
		1	1	1
1				
		912988	90519	68596
449689				
		1		
		0		
		912988		
49.7%				
	100.0%			
49.7%				
	100.0%			

For peer review only

49.7%

For peer review only

Novartis	Novo Nordisk	Octapharma	Orion Pharma	Orphan Europe	ka Pharmaceut
1	1	0	1	0	1
435	47	2	8	0	13
154	17	7	4	3	11
1	1	1	1	0	1
1	1	1	1	1	1
1	1	1	1	1	1
1	1	1	1	1	1
1	1				1
1					
1	1		1		1
		1		1	
281	30		4		2
		5		3	
1	1		1		1
		1		1	
281	30		4		2
		5		3	
1	1				1
281	30				2

For peer review only

1					
281					
					15.4%
					15.4%
				100.0%	
					15.4%
					15.4%
				100.0%	
					15.4%
					15.4%
5,195,484.0	753,861.7	5,276.0	49,682.0	-	184,272.2
2,102,441.4	49,315.6	435,156.4	-	1,028.5	88,840.6
433	47	2	8	0	13
104	3	7	0	1	6
1	1	1	1	0	1
1	1	1	0	1	1
1	1	1	1	1	1
1	1	1			1

For peer review only

1					
1	1		1		1
		1		1	
3093043	704546		49682		95432
		429880		1029	
1	1				1
		1			
3093043	704546				95432
		429880			
1					
0					
3093043					
			100.0%	100.0%	
			100.0%	100.0%	

For peer review only

For peer review only

Pfizer	Pharma Mar	PTC	RB	Roche Products	ont Pharmace
1	1	0	0	1	0
886	0	0	0	99	7
129	2	6	4	70	1
1	0	0	0	1	1
1	1	1	1	1	1
1	1	1	1	1	1
1	1	1	1	1	1
1				1	
1					
1				1	1
	1	1	1		
757				29	6
	2	6	4		
1				1	1
	1	1	1		
757				29	6
	2	6	4		
1				1	
757				29	

For peer review only

1					
757					
				29.3%	
	100.0%	100.0%	100.0%		
				29.3%	
	100.0%	100.0%	100.0%		
				29.3%	
9,304,016.7	-	-	-	1,369,625.3	-
10,713,090.3	6,312.5	-	144,270.0	660,704.8	-
831	0	0	0	99	0
57	1	0	4	27	0
1	0	0	0	1	0
1	1	0	1	1	0
1	1		1	1	
1			1	1	

For peer review only

1			1
			1
1	1	1	
			708920
1409074	6313	144270	
			1
1		1	
			708920
1409074		144270	
			1
1			
0			0
			708920
1409074			
13.2%			
13.2%			
	100.0%	100.0%	
13.2%			
13.2%			
	100.0%	100.0%	
13.2%			

For peer review only

13.2%

100.0%

13.2%

13.2%

For peer review only

Sanofi	nofi Pasteur MS	Santen	vier Laborator	Shire	a Tau Rare Dis
1	0	1	1	1	0
224	0	1	20	115	0
37	2	2	3	37	8
1	0	1	1	1	0
1	1	1	1	1	1
1	1	1	1	1	1
1	1	1	1	1	1
1			1	1	
1				1	
1			1	1	
	1	1			1
187			17	78	
	2	1			8
1			1	1	
	1	1			1
187			17	78	
	2	1			8
1			1	1	
187			17	78	

For peer review only

For peer review only - http://bmjopen.bmj.com/site/about/guidelines.xhtml

1				1	
187				78	
	100.0%				100.0%
	100.0%				100.0%
2,562,336.3	-	4,000.0	76,304.9	1,266,644.8	-
189,271.9	20,989.1	-	10,857.5	417,018.3	53,676.1
219	0	1	20	115	0
10	1	0	2	18	4
1	0	1	1	1	0
1	1	0	1	1	1
1	1	1	1	1	1
1				1	

For peer review only

1				1	
1		1	1	1	
	1				1
2373064		4000	65447	849626	
	20989				53676
1				1	
2373064				849626	
1				1	
0				0	
2373064				849626	
	100.0%	100.0%			100.0%
	100.0%	100.0%			100.0%

For peer review only

For peer review only

Sobi	Stirling Angliar	Syner-Med	Takeda	Teva	Thea
1	0	0	1	0	0
36	3	0	54	24	0
13	1	3	42	28	1
1	1	0	1	1	0
1	1	1	1	1	1
1	1	1	1	1	1
1	1	1	1	1	
1			1	1	
1	1		1		
		1		1	1
23	2		12		
		3		4	1
1	1		1		
		1		1	
23	2		12		
		3		4	
1			1		
				1	
23			12		
				4	

For peer review only

				14.3%	
			22.2%	14.3%	
		100.0%			100.0%
				14.3%	
			22.2%	14.3%	
		100.0%			
				14.3%	
			22.2%	14.3%	
224,819.9	11,110.0	-	6,614,214.3	642,199.4	-
20,701.1	-	-	207,863.1	274,284.3	-
36	3	0	54	23	0
6	0	0	16	13	0
1	1	0	1	1	0
1	0	0	1	1	0
1	1		1	1	
1			1	1	

For peer review only

		1	
1	1	1	1
204119	11110	6406351	367915
1		1	1
204119		6406351	367915
		1	
		0	
		6406351	
	100.0%		
	100.0%		

For peer review only

For peer review only

Tillotts	UCB	Veriton	ifor Pharma Grou
0	1	0	0
7	86	0	3
6	37	4	12
1	1	0	1
1	1	1	1
1	1	1	1
1	1	1	1
	1		1
1	1		
		1	1
1	49		
		4	9
1	1		
		1	1
1	49		
		4	9
	1		
			1
	49		
			9

For peer review only

14.3%			
14.3%			
		100.0%	
14.3%			
14.3%			
		100.0%	
48,612.7	534,290.2	-	20,350.0
32,002.9	84,069.3	-	2,828,703.7
7	84	0	3
2	10	0	6
1	1	0	1
1	1	0	1
1	1		1
	1		1

For peer review only

		1
1	1	
		1
16610	450221	
		2808354
	1	
		1
	450221	
		2808354
		1
		0
		2808354
34.2%		
34.2%		

For peer review only

For peer review only

Web Supplement 4. Donors - Absolute and relative differences in the number and value of payment

NUMBER OF PAYMENTS - ABSOLUTE DIFFERENCES

Threshold 0: At least 1 one payment in at least one dataset
Total
Number of drug companies with exact match in both datasets
Number of drug companies with more paymens t in industry data
Number of drug companies with more payments in patient organisation data

Highest absolute difference between patient organisation and industry data - number of payments hi
Highest absolute difference between patient organisation and industry data - number of payments hi

Threshold 1: More than 1 one payment in at least one datase
Total
Number of drug companies with exact match in both datasets
Number of drug companies with more paymens t in industry data
Number of drug companies with more payments in patient organisation data

Highest absolute difference between patient organisation and industry data - number of payments hi
Highest absolute difference between patient organisation and industry data - number of payments hi

Threshold 2: More than 10 payments in at least one dataset
Total
Number of drug companies with exact match in both datasets
Number of drug companies with more paymens t in industry data
Number of drug companies with more payments in patient organisation data

Highest absolute difference between patient organisation and industry data - number of payments hi
Highest absolute difference between patient organisation and industry data - number of payments hi

Threshold 3: More than 100 payments in at least one dataset
Total
Number of drug companies with exact match in both datasets
Number of drug companies with more paymens t in industry data
Number of drug companies with more payments in patient organisation data

Highest absolute difference between patient organisation and industry data - number of payments hi
Highest absolute difference between patient organisation and industry data - number of payments hi

NUMBER OF PAYMENTS - RELATIVE DIFFERENCES

Threshold 0: At least 1 one payment in at least one dataset

For peer review only

Total
Relative difference <10%
Relative difference <20%
Relative difference <50%
Relative difference = 100%

Threshold 1: More than 1 one payment in at least one datase
Total
Relative difference <10%
Relative difference <20%
Relative difference <50%
Relative difference = 100%

Threshold 2: More than 10 payments in at least one dataset
Total
Relative difference <10%
Relative difference <20%
Relative difference <50%
Relative difference = 100%

Threshold 3: More than 100 payments in at least one dataset
Total
Relative difference <10%
Relative difference <20%
Relative difference <50%
Relative difference = 100%

VALUE OF PAYMENTS - ABSOLUTE DIFFERENCES

Threshold 1: At least one payment with value >£0 in at least one dataset
Total
Number of drug companies with exact match in both datasets
Number of drug companies with higher payment value in industry data
Number of drug companies with higher payment value in patient organisation data

Highest absolute difference between patient organisation and industry data - number of payments hi
Highest absolute difference between patient organisation and industry data - number of payments hi

Threshold 2: Payments with the value of more than £10,000 in at least one dataset
Total
Number of drug companies with exact match in both datasets
Number of drug companies with higher payment value in industry data
Number of drug companies with higher payment value in patient organisation data

For peer review only

Highest absolute difference between patient organisation and industry data - number of payments hi[illegible]
Highest absolute difference between patient organisation and industry data - number of payments hi[illegible]

Threshold 3: Payments with the value of more than £100,000 in at least one dataset
Total
Number of drug companies with exact match in both datasets
Number of drug companies with higher payment value in industry data
Number of drug companies with higher payment value in patient organisation data

Highest absolute difference between patient organisation and industry data - number of payments hi[illegible]
Highest absolute difference between patient organisation and industry data - number of payments hi[illegible]

VALUE OF PAYMENTS- RELATIVE DIFFERENCES

Threshold 1: At least one payment with value >£0 in at least one dataset
Total
Relative difference <10%
Relative difference <20%
Relative difference <50%
Relative difference = 100%

Threshold 2: Payments with the value of at least £10,000 in at least one dataset
Total
Relative difference <10%
Relative difference <20%
Relative difference <50%
Relative difference = 100%

Threshold 3: Payments with the value of at least 100,000 in at least one dataset
Total
Relative difference <10%
Relative difference <20%
Relative difference <50%
Relative difference = 100%

For peer review only

**All years**			**2012**			**2013**		
Number of drug cc	% of all drug (	0	Number of drug	% of all drug (	0	Number of dru	% of all drug (	0
87	100.0%		61	100.0%		68	100.0%	
3	3.4%		1	1.6%		7	10.3%	
49	56.3%		28	45.9%		28	41.2%	
35	40.2%		32	52.5%		33	48.5%	
Number of payments			Number of payments			Number of payments		
757			216			135		
53			18			16		
Number of drug cc	% of all drug (	0	Number of drug	% of all drug (	0	Number of dru	% of all drug (	0
80	100.0%		46	100.0%		55	100.0%	
3	3.8%		1	2.2%		3	5.5%	
49	61.3%		28	60.9%		27	49.1%	
28	35.0%		17	37.0%		25	45.5%	
Number of payments			Number of payments			Number of payments		
757			216			135		
53			18			16		
Number of drug cc	% of all drug (	0	Number of drug	% of all drug (	0	Number of dru	% of all drug (	0
47	100.0%		16	100.0%		17	100.0%	
1	2.1%		0	0.0%		0	0.0%	
36	76.6%		13	81.3%		14	82.4%	
10	21.3%		3	18.8%		3	17.6%	
Number of payments			Number of payments			Number of payments		
757			216			135		
53			18			16		
Number of drug cc	% of all drug (	0	Number of drug	% of all drug (	0	Number of dru	% of all drug (	0
10	100.0%		1	100.0%		1	100.0%	
0	0.0%		0	0.0%		0	0.0%	
10	100.0%		1	100.0%		1	100.0%	
0	0.0%		0	0.0%		0	0.0%	
Number of payments			Number of payments			Number of payments		
757			216			135		
0			0			0		
Number of drug cc	% of all drug cor		Number of drug	% of all drug cor		Number of dru	% of all drug cor	

For peer review only

87	100.0%	61	100.0%	68	100.0%
5	5.7%	1	1.6%	7	10.3%
9	10.3%	2	3.3%	7	10.3%
21	24.1%	7	11.5%	10	14.7%
27	31.0%	36	59.0%	33	48.5%
Number of drug cc	% of all drug (0	Number of drug	% of all drug (0	Number of dru	% of all drug (0
80	100.0%	46	100.0%	55	100.0%
5	6.3%	1	2.2%	3	5.5%
9	11.3%	2	4.3%	3	5.5%
21	26.3%	7	15.2%	6	10.9%
20	25.0%	21	45.7%	24	43.6%
Number of drug cc	% of all drug (0	Number of drug	% of all drug (0	Number of dru	% of all drug (0
47	100.0%	16	100.0%	17	100.0%
3	6.4%	0	0.0%	0	0.0%
5	10.6%	0	0.0%	0	0.0%
15	31.9%	1	6.3%	0	0.0%
2	4.3%	3	18.8%	3	17.6%
Number of drug cc	% of all drug (0	Number of drug	% of all drug (0	Number of dru	% of all drug (0
10	100.0%	1	100.0%	1	100.0%
0	0.0%	0	0.0%	0	0.0%
0	0.0%	0	0.0%	0	0.0%
1	10.0%	0	0.0%	0	0.0%
0	0.0%	0	0.0%	0	0.0%
Number of drug cc	% of all drug (0	Number of drug	% of all drug (0	Number of dru	% of all drug (0
74	100.0%	52	100.0%	56	100.0%
0	0.0%	0	0.0%	0	0.0%
48	64.9%	26	50.0%	35	62.5%
26	35.1%	26	50.0%	21	37.5%
Value of payments		Value of payments		Value of payments	
6,406,351.2		1,576,293.48		666,171.11	
2,960,716.0		501,114.66		851,009.34	
Number of drug cc	% of all drug (0	Number of drug	% of all drug (0	Number of dru	% of all drug (0
45	100.0%	20	100.0%	24	100.0%
0	0.0%	0	0.0%	0	0.0%
32	71.1%	13	65.0%	14	58.3%
13	28.9%	7	35.0%	10	41.7%
Value of payments		Value of payments		Value of payments	

For peer review only

6,406,351.2	1,576,293.5	666,171.1
2,960,716.0	501,114.7	851,009.3

Number of drug cc	% of all drug (	0	Number of drug	% of all drug (	0	Number of dru	% of all drug (	0
17	100.0%		2	100.0%		1	100.0%	
0	0.0%		0	0.0%		0	0.0%	
13	76.5%		2	100.0%		1	100.0%	
4	23.5%		0	0.0%		0	0.0%	

Value of payments	Value of payments	Value of payments
6,406,351.2	1,576,293.5	604,556.0
2,960,716.0	-	-

Number of drug cc	% of all drug (	0	Number of drug	% of all drug (	0	Number of dru	% of all drug (	0
74	100.0%		52	100.0%		56	100.0%	
3	4.1%		1	1.9%		0	0.0%	
9	12.2%		1	1.9%		4	7.1%	
14	18.9%		4	7.7%		7	12.5%	
24	32.4%		32	61.5%		30	53.6%	

Number of drug cc	% of all drug (	0	Number of drug	% of all drug (	0	Number of dru	% of all drug (	0
45	100.0%		20	100.0%		24	100.0%	
1	2.2%		0	0.0%		0	0.0%	
5	11.1%		0	0.0%		1	4.2%	
9	20.0%		1	5.0%		3	12.5%	
4	8.9%		8	40.0%		9	37.5%	

Number of drug cc	% of all drug (	0	Number of drug	% of all drug (	0	Number of dru	% of all drug (	0
17	100.0%		2	100.0%		1	100.0%	
0	0.0%		0	0.0%		0	0.0%	
2	11.8%		0	0.0%		0	0.0%	
2	11.8%		0	0.0%		1	100.0%	
1	5.9%		0	0.0%		0	0.0%	

For peer review only

2014			2015			2016	
Number of drug	% of all drug (	0	Number of drug	% of all drug (	0	Number of drug	% of all drug (
72	100.0%		74	100.0%		69	100.0%
4	5.6%		7	9.5%		4	5.8%
38	52.8%		42	56.8%		39	56.5%
30	41.7%		25	33.8%		26	37.7%
Number of payments			Number of payments			Number of payments	
108			125			173	
13			13			12	
Number of drug	% of all drug (	0	Number of drug	% of all drug (	0	Number of drug	% of all drug (
55	100.0%		57	100.0%		50	100.0%
1	1.8%		5	8.8%		3	6.0%
36	65.5%		42	73.7%		38	76.0%
18	32.7%		10	17.5%		9	18.0%
Number of payments			Number of payments			Number of payments	
108			125			173	
13			13			12	
Number of drug	% of all drug (	0	Number of drug	% of all drug (	0	Number of drug	% of all drug (
21	100.0%		23	100.0%		23	100.0%
0	0.0%		0	0.0%		0	0.0%
18	85.7%		22	95.7%		22	95.7%
3	14.3%		1	4.3%		1	4.3%
Number of payments			Number of payments			Number of payments	
108			125			173	
13			13			12	
Number of drug	% of all drug (	0	Number of drug	% of all drug (	0	Number of drug	% of all drug (
2	100.0%		1	100.0%		100.0%	100.0%
0	0.0%		0	0.0%		0	0.0%
2	100.0%		1	100.0%		1	100.0%
0	0.0%		0	0.0%		0	0.0%
Number of payments			Number of payments			Number of payments	
108			125			173	
0			0			0	
Number of drug	% of all drug cor		Number of drug	% of all drug cor		Number of drug	% of all drug (

For peer review only

72	100.0%	74	100.0%	69	100.0%
4	5.6%	7	9.5%	4	5.8%
5	6.9%	9	12.2%	5	7.2%
12	16.7%	17	23.0%	12	17.4%
34	47.2%	28	37.8%	29	42.0%

Number of drug	% of all drug (0	Number of drug	% of all drug (0	Number of drug	% of all drug (
55	100.0%	57	100.0%	50	100.0%
1	1.8%	5	8.8%	3	6.0%
2	3.6%	7	12.3%	4	8.0%
9	16.4%	15	26.3%	11	22.0%
20	36.4%	13	22.8%	11	22.0%

Number of drug	% of all drug (0	Number of drug	% of all drug (0	Number of drug	% of all drug (
21	100.0%	23	100.0%	23	100.0%
0	0.0%	0	0.0%	0	0.0%
1	4.8%	0	0.0%	0	0.0%
3	14.3%	4	17.4%	2	8.7%
2	9.5%	1	4.3%	1	4.3%

Number of drug	% of all drug (0	Number of drug	% of all drug (0	Number of drug	% of all drug (
2	100.0%	1	100.0%	100.0%	100.0%
0	0.0%	0	0.0%	0	0.0%
0	0.0%	0	0.0%	0	0.0%
0	0.0%	0	0.0%	0	0.0%
0	0.0%	0	0.0%	0	0.0%

Number of drug	% of all drug (0	Number of drug	% of all drug (0	Number of drug	% of all drug (
59	100.0%	60	100.0%	55	100.0%
0	0.0%	1	1.7%	0	0.0%
39	66.1%	41	68.3%	38	69.1%
20	33.9%	18	30.0%	17	30.9%

Value of payments	Value of payments	Value of payments
902,858.00	1,637,974.69	5,986,743.37
2,123,196.45	729,868.42	5,541,099.82

Number of drug	% of all drug (0	Number of drug	% of all drug (0	Number of drug	% of all drug (
28	100.0%	30	100.0%	28	100.0%
0	0.0%	0	0.0%	0	0.0%
21	75.0%	25	83.3%	23	82.1%
7	25.0%	5	16.7%	5	17.9%

Value of payments	Value of payments	Value of payments

For peer review only

902,858.0	1,637,974.7	5,986,743.4
2,123,196.4	729,868.4	5,541,099.8

Number of drug	% of all drug (	0 Number of drug	% of all drug (	0 Number of drug	% of all drug (
3	100.0%	2	100.0%	2	100.0%
0	0.0%	0	0.0%	0	0.0%
2	66.7%	2	100.0%	1	50.0%
1	33.3%	0	0.0%	1	50.0%

Value of payments	Value of payments	Value of payments
902,858.0	1,637,974.7	5,986,743.4
2,123,196.4	-	5,541,099.8

Number of drug	% of all drug (	0 Number of drug	% of all drug (	0 Number of drug	% of all drug (
59	100.0%	60	100.0%	55	100.0%
1	1.7%	5	8.3%	0	0.0%
1	1.7%	8	13.3%	4	7.3%
8	13.6%	17	28.3%	14	25.5%
31	52.5%	24	40.0%	22	40.0%

Number of drug	% of all drug (	0 Number of drug	% of all drug (	0 Number of drug	% of all drug (
28	100.0%	30	100.0%	28	100.0%
1	3.6%	2	6.7%	0	0.0%
1	3.6%	2	6.7%	3	10.7%
5	17.9%	8	26.7%	10	35.7%
8	28.6%	6	20.0%	4	14.3%

Number of drug	% of all drug (	0 Number of drug	% of all drug (	0 Number of drug	% of all drug (
3	100.0%	2	100.0%	2	100.0%
0	0.0%	0	0.0%	0	0.0%
0	0.0%	0	0.0%	0	0.0%
0	0.0%	0	0.0%	0	0.0%
1	33.3%	0	0.0%	0	0.0%

For peer review only

companies at a given threshold

companies at a given threshold

companies at a given threshold

companies at a given threshold

companies

For peer review only

companies at a given threshold

companies at a given threshold

companies at a given threshold

companies at a given threshold

companies at a given threshold

For peer review only

companies at a given threshold

companies at a given threshold

companies at a given threshold

companies at a given threshold

For peer review only

Web Supplement 5. Recipients - Absolute and relative differences in the number and value of paym

NUMBER OF PAYMENTS

Number of payments - industry data
Number of payments - patient organisation data
Number of patient organisations with at least 1 payment - industry data
Number of patient organisations with at least 1 payment - patient organisation data

NUMBER OF PAYMENTS - THRESHOLDS

Threshold 0: Number of patient organisations with at least 1 one payment in at least one dataset
Threshold 1: Number of patient organisations with more than 1 one payment in at least one dataset
Threshold 2: Number of patient organisations with more than 10 payments in at least one dataset
Threshold 3: Number of patient organisations with more than 100 payments in at least one dataset

NUMBER OF PAYMENTS - ABSOLUTE DIFFERENCES

Number of payments - threshold 0
Number of patient organisations with exact match in both datasets
Number of patient organisations with more paymens in industry data
Number of patient organisations with more payments in patient organisation data

Highest absolute difference between patient organisation and industry data - number of payments hi
Highest absolute difference between patient organisation and industry data - number of payments hi

Number of payments - threshold 1
Number of patient organisations with exact match in both datasets
Number of patient organisations with more paymens in industry data
Number of patient organisations with more payments in patient organisation data

Highest absolute difference between patient organisation and industry data - number of payments hi
Highest absolute difference between patient organisation and industry data - number of payments hi

Number of payments - threshold 2
Number of patient organisations with exact match in both datasets
Number of patient organisations with more paymens in industry data
Number of patient organisations with more payments in patient organisation data

Highest absolute difference between patient organisation and industry data - number of payments hi
Highest absolute difference between patient organisation and industry data - number of payments hi

Number of payments - threshold 3

For peer review only

Number of patient organisations with exact match in both datasets
Number of patient organisations with more paymens in industry data
Number of patient organisations with more payments in patient organisation data

Highest absolute difference between patient organisation and industry data - number of payments hi
Highest absolute difference between patient organisation and industry data - number of payments hi

NUMBER OF PAYMENTS - RELATIVE DIFFERENCES

Relative difference <10%
Relative difference <20%
Relative difference <50%
Relative difference = 100%

Number of payments - threshold 1
Relative difference <10%
Relative difference <20%
Relative difference <50%
Relative difference = 100%

Number of payments - threshold 2
Relative difference <10%
Relative difference <20%
Relative difference <50%
Relative difference = 100%

Number of payments - threshold 3
Relative difference <10%
Relative difference <20%
Relative difference <50%
Relative difference = 100%

VALUE OF PAYMENTS - ABSOLUTE DIFFERENCES
Value of payments - industry data (2016 £)
Value of payments - patient organisation data (2016 £)
Number of payments with value >£0 - industry data
Number of payments with value >£0 - patient organisation data
Number of patient organisations with at least one payemnt with the value >£0 - industry data
Number of patient organisations with at least one payemnt with the value >£0 - patient organisation

VALUE OF PAYMENTS - THRESHOLDS

Threshold 1: Number of patient organisations with at least one payment with value >0 in at least one
Threshold 2: Number of patient organisations with payments with the value of more than £10,000 in
Threshold 3: Number of patient organisations with payments with the value of more than £100,000 ir

For peer review only

VALUE OF PAYMENTS - ABSOLUTE DIFFERENCES

Number of payments - threshold 1

Number of patient organisations with exact match in both datasets
Number of patient organisations with higher payment value in industry data
Number of patient organisations with higher payment value in patient organisation data

Highest absolute difference between patient organisation and industry data - number of payments hi[illegible]
Highest absolute difference between patient organisation and industry data - number of payments hi[illegible]

Number of payments - threshold 2

Number of patient organisations with exact match in both datasets
Number of patient organisations with higher payment value in industry data
Number of patient organisations with higher payment value in patient organisation data

Highest absolute difference between patient organisation and industry data - number of payments hi[illegible]
Highest absolute difference between patient organisation and industry data - number of payments hi[illegible]

Number of payments - threshold 3

Number of patient organisations with exact match in both datasets
Number of patient organisations with higher payment value in industry data
Number of patient organisations with higher payment value in patient organisation data

Highest absolute difference between patient organisation and industry data - number of payments hi[illegible]
Highest absolute difference between patient organisation and industry data - number of payments hi[illegible]

VALUE OF PAYMENTS - RELATIVE DIFFERENCES

Relative difference <10%
Relative difference <20%
Relative difference <50%
Relative difference = 100%

Number of payments - threshold 1

Relative difference <10%
Relative difference <20%
Relative difference <50%
Relative difference = 100%

Number of payments - threshold 2

Relative difference <10%
Relative difference <20%

For peer review only

Relative difference <50%

Relative difference = 100%

Number of payments - threshold 3

Relative difference <10%

Relative difference <20%

Relative difference <50%

Relative difference = 100%

For peer review only

		21 and Co	AADC Resear	Action Bladde	Action Duche
Total					
4316		1	1	1	12
1661		0	0	0	0
425		1	1	1	1
200		0	0	0	0
Number of patient	% of all patient organisations with at least one payment in at least one d				
425	100.0%	1	1	1	1
299	70.4%				1
115	27.1%				1
3	0.7%				
Number of patient	% of all patient organisations at a given threshold				
33	7.8%				
335	78.8%	1	1	1	1
57	13.4%				
Number of payments					
199		1	1	1	12
33					
Number of patient	% of all patient organisations at a given threshold				
13	4.3%				
229	76.6%				1
57	19.1%				
Number of payments					
199					12
33					
Number of patient	% of all patient organisations at a given threshold				
2	1.7%				
95	82.6%				1
18	15.7%				
Number of payments					
199					12
33					
Number of patient	% of all patient organisations at a given threshold				

For peer review only

0	0.0%				
3	100.0%				
0	0.0%				
Number of payments					
199					
0					
Number of patient	% of all patient organisations with at least one payment in at least one d				
35	8.2%				
42	9.9%				
81	19.1%				
225	52.9%	100.0%	100.0%	100.0%	100.0%
Number of patient	% of all patient organisations at a given threshold				
15	5.0%				
22	7.4%				
61	20.4%				
119	39.8%				100.0%
Number of patient	% of all patient organisations at a given threshold				
4	3.5%				
7	6.1%				
31	27.0%				
33	28.7%				100.0%
Number of patient	% of all patient organisations at a given threshold				
0	0.0%				
0	0.0%				
0	0.0%				
1	33.3%				
£54,071,454.2		1029	5070	101	47076
£33,037,955.8		0	0	0	0
4235		1	1	1	12
772		0	0	0	0
416		1	1	1	1
121		0	0	0	0
416		1	1	1	1
104					
13					

For peer review only

Number of patient	% of all patient organisations at a given threshold				
8	1.9%				
356	85.6%	1	1	1	1
52	12.5%				
Vallue of payments		0	0	0	0
6,718,576.6		1029	5070	101	47076
6,493,237.1					
Number of patient	% of all patient organisations at a given threshold				
0	0.0%				
80	76.9%				
24	23.1%				
Vallue of payments					
6,718,576.6					
6,493,237.1					
Number of patient	% of all patient organisations at a given threshold				
0	0.0%				
6	46.2%				
7	53.8%				
Vallue of payments					
6,718,576.6					
6,493,237.1					
Number of patient	% of all patient organisations with at least one payment with value >£0 i				
21	5.0%				
34	8.2%				
73	17.5%				
295	70.9%	100.0%	100.0%	100.0%	100.0%
21	5.0%				
34	8.2%				
73	17.5%				
295	70.9%	100.0%	100.0%	100.0%	100.0%
4	3.8%				
8	7.7%				

For peer review only

23	22.1%
54	51.9%
0	0.0%
0	0.0%
3	23.1%
4	30.8%

For peer review only

	Action for Pul	Action on Pai	Action on Pre	Action on Sm	Action on Sm	Actionplus Fo	Add+Up
	3	12	1	1	5	2	3
	2	0	1	0	5	0	5
	1	1	1	1	1	1	1
	1	0	1	0	1	0	1
lataset							
	1	1	1	1	1	1	1
	1	1			1	1	1
		1					
			1		1		
	1	1		1		1	
							1
	1	12		1		2	
							2
					1		
	1	1				1	
							1
	1	12				2	
							2
		1					
		12					

For peer review only

lataset

		0.0%		0.0%		
		0.0%		0.0%		
33.3%		0.0%		0.0%		40.0%
	100.0%		100.0%		100.0%	
				0.0%		
				0.0%		
33.3%				0.0%		40.0%
	100.0%				100.0%	
	100.0%					
14053	13947	10521	2520	28933	7941	30629
18300	0	10285	0	33478	0	37297
3	10	1	1	5	2	3
2	0	1	0	5	0	5
1	1	1	1	1	1	1
1	0	1	0	1	0	1
1	1	1	1	1	1	1

For peer review only

	1	1	1		1	
1				1		1
0	0	0	0	0	0	0
	13947	236	2520		7941	
4247				4545		6667

For peer review only

in at least one dataset

		2.2%				
		2.2%		13.6%		17.9%
23.2%		2.2%		13.6%		17.9%
	100.0%		100.0%		100.0%	
		2.2%				
		2.2%		13.6%		17.9%
23.2%		2.2%		13.6%		17.9%
	100.0%		100.0%		100.0%	

For peer review only

Addaction	Addenbrooks	Addison's Dis	Adfam	ADHD Founda	Africa Advoca	African Healt
20	1	3	1	9	8	12
2	0	1	2	1	7	6
1	1	1	1	1	1	1
1	0	1	1	1	1	1
1	1	1	1	1	1	1
1		1	1	1	1	1
1						1
1	1	1		1	1	1
			1			
18	1	2		8	1	6
			1			
1		1		1	1	1
			1			
18		2		8	1	6
			1			
1						1
18						6

For peer review only

12.5%

12.5%

100.0%

12.5%

12.5%

For peer review only

356861	3042	25000	270	27042	40023	57095
98542	0	25000	35800	20281	35149	23988
20	1	3	1	9	8	12
2	0	1	2	1	6	5
1	1	1	1	1	1	1
1	0	1	1	1	1	1
1	1	1	1	1	1	1
1						

		1				
1	1			1	1	1
			1			
0	0	0	0	0	0	0
258319	3042			6761	4874	33107
			35530			
1						
0						
258319						
		0.0%				
		0.0%			12.2%	
		0.0%		25.0%	12.2%	
	100.0%					
		0.0%				
		0.0%			12.2%	
		0.0%		25.0%	12.2%	
	100.0%					

For peer review only

For peer review only

African-Caribl	African's Gett	Age Related [	Age UK	Age UK - Cym	AGE UK - Isle	aHUS UK
6	1	2	12	2	1	1
0	0	0	4	4	2	0
1	1	1	1	1	1	1
0	0	0	1	1	1	0
1	1	1	1	1	1	1
1		1	1	1	1	
			1			
1	1	1	1			1
				1	1	
6	1	2	8			1
				2	1	
1		1	1			
				1	1	
6		2	8			
				2	1	
			1			
			8			

For peer review only

100.0%	100.0%	100.0%				100.0%
100.0%		100.0%				
70152	130	39259	97127	11210	42574	25250
0	0	0	1825301	0	24278	0
6	1	2	12	2	1	1
0	0	0	1	0	1	0
1	1	1	1	1	1	1
0	0	0	1	0	1	0
1	1	1	1	1	1	1
			1			
			1			

For peer review only

1	1	1		1	1	1
			1			
0	0	0	0	0	0	0
70152	130	39259		11210	18296	25250
			1728174			
			1			
			0			
			1728174			
			1			
			0			
			1728174			
					43.0%	
100.0%	100.0%	100.0%		100.0%		100.0%
					43.0%	
100.0%	100.0%	100.0%		100.0%		100.0%

For peer review only

For peer review only

Airedale Volu	Alcohol Conce	ALD Life	Allergy Resea	Allergy UK	Alpha-1 Awar	Alpha-1 UK Su
3	18	1	2	23	2	5
3	2	0	0	8	0	0
1	1	1	1	1	1	1
1	1	0	0	1	0	0
1	1	1	1	1	1	1
1	1		1	1	1	1
	1			1		
1						
	1	1	1	1	1	1
	16	1	2	15	2	5
1						
	1		1	1	1	1
	16		2	15	2	5
	1			1		
	16			15		

For peer review only

0.0%						
0.0%						
0.0%						
		100.0%	100.0%		100.0%	100.0%
0.0%						
0.0%						
0.0%						
			100.0%		100.0%	100.0%
34921	128034	262	21042	280169	16456	138585
34921	0	0	0	86271	0	0
3	18	1	2	23	2	5
3	0	0	0	2	0	0
1	1	1	1	1	1	1
1	0	0	0	1	0	0
1	1	1	1	1	1	1
	1			1		1

For peer review only

1						
	1	1	1	1	1	1
0	0	0	0	0	0	0
	128034	262	21042	193898	16456	138585
	1			1		1
	0			0		0
	128034			193898		138585
0.0%						
0.0%						
0.0%						
	100.0%	100.0%	100.0%		100.0%	100.0%
0.0%						
0.0%						
0.0%						
	100.0%	100.0%	100.0%		100.0%	100.0%

For peer review only

100.0% 100.0%

For peer review only

**Alzheimer Sc**	**Alzheimer's R**	**Alzheimer’s S**	**Alzheimers D**	**Anaphylaxis (**	**Angelman Sy**	**Ankylosing Sp**
2	7	18	1	8	1	6
0	10	5	0	3	0	0
1	1	1	1	1	1	1
0	1	1	0	1	0	0
1	1	1	1	1	1	1
1	1	1		1		1
		1				
1		1	1	1	1	1
	1					
2		13	1	5	1	6
	3					
1		1		1		1
	1					
2		13		5		6
	3					
		1				
		13				

For peer review only

For peer review only

	30.0%					
100.0%			100.0%		100.0%	100.0%
	30.0%					
100.0%						100.0%
564	443860	253057	1057	45474	303	38723
0	0	0	0	0	0	0
2	7	18	1	8	1	6
0	0	0	0	0	0	0
1	1	1	1	1	1	1
0	0	0	0	0	0	0
1	1	1	1	1	1	1
	1	1				

1	1	1	1	1	1	1
0	0	0	0	0	0	0
564	443860	253057	1057	45474	303	38723
	1	1				
	0	0				
	443860	253057				
100.0%	100.0%	100.0%	100.0%	100.0%	100.0%	100.0%
100.0%	100.0%	100.0%	100.0%	100.0%	100.0%	100.0%

For peer review only

100.0%	100.0%

For peer review only

Anthony Nola	Anticoagulati	Anxiety UK	Aplastic Anae	Arrhythmia A	Arthritis and I	Arthritis Care
3	76	8	1	34	32	50
2	0	2	0	0	0	10
1	1	1	1	1	1	1
1	0	1	0	0	0	1
1	1	1	1	1	1	1
1	1	1		1	1	1
	1			1	1	1
1	1	1	1	1	1	1
1	76	6	1	34	32	40
1	1	1		1	1	1
1	76	6		34	32	40
	1			1	1	1
	76			34	32	40

For peer review only

33.3%						
	100.0%		100.0%	100.0%	100.0%	
33.3%						
	100.0%			100.0%	100.0%	
	100.0%			100.0%	100.0%	
30494	375207	21339	1616	329518	262194	391558
0	0	0	0	0	0	0
3	76	7	1	34	32	49
0	0	0	0	0	0	0
1	1	1	1	1	1	1
0	0	0	0	0	0	0
1	1	1	1	1	1	1
	1			1	1	1

For peer review only

1	1	1	1	1	1	1
0	0	0	0	0	0	0
30494	375207	21339	1616	329518	262194	391558
	1			1	1	1
	0			0	0	0
	375207			329518	262194	391558
100.0%	100.0%	100.0%	100.0%	100.0%	100.0%	100.0%
100.0%	100.0%	100.0%	100.0%	100.0%	100.0%	100.0%

For peer review only

100.0%	100.0%	100.0%	100.0%

For peer review only

Arthritis Rese	Association fo	Association fo	Asthma and A	Asthma Relie	Asthma UK	Asyabi UK
6	13	6	1	1	34	1
3	1	5	0	0	34	0
1	1	1	1	1	1	1
1	1	1	0	0	1	0
1	1	1	1	1	1	1
1	1	1			1	
	1				1	
					1	
1	1	1	1	1		1
3	12	1	1	1		1
					1	
1	1	1				
3	12	1				
					1	
	1					
	12					

For peer review only

					0.0%	
		16.7%			0.0%	
		16.7%			0.0%	
			100.0%	100.0%		100.0%
					0.0%	
		16.7%			0.0%	
		16.7%			0.0%	
					0.0%	
					0.0%	
					0.0%	
248103	71616	35059	507	1014	301952	1500
0	0	20100	0	0	0	0
5	13	5	1	1	33	1
0	0	2	0	0	0	0
1	1	1	1	1	1	1
0	0	1	0	0	0	0
1	1	1	1	1	1	1
1					1	

For peer review only

1	1	1	1	1	1	1
0	0	0	0	0	0	0
248103	71616	14959	507	1014	301952	1500
1					1	
0					0	
248103					301952	
		42.7%				
100.0%	100.0%		100.0%	100.0%	100.0%	100.0%
		42.7%				
100.0%	100.0%		100.0%	100.0%	100.0%	100.0%

For peer review only

100.0% 100.0%

For peer review only

Ataxia UK	Atrial Fibrillat	Back on Track	BackCare	Barts and The	Basil Skyers M	Batten Diseas
8	104	1	11	1	4	10
8	0	0	4	0	0	4
1	1	1	1	1	1	1
1	0	0	1	0	0	1
1	1	1	1	1	1	1
1	1		1		1	1
	1		1			
	1					
1						
	1	1	1	1	1	1
	104	1	7	1	4	6
1						
	1		1		1	1
	104		7		4	6
	1		1			
	104		7			

For peer review only

	1					
	104					
0.0%						
0.0%						
0.0%						
	100.0%	100.0%		100.0%	100.0%	
0.0%						
0.0%						
0.0%						
	100.0%				100.0%	
	100.0%					
	100.0%					
33556	621144	3809	43576	206	35201	73762
36402	0	0	9127	0	0	0
8	102	1	8	1	4	10
2	0	0	2	0	0	0
1	1	1	1	1	1	1
1	0	0	1	0	0	0
1	1	1	1	1	1	1
	1					

For peer review only

For peer review only - http://bmjopen.bmj.com/site/about/guidelines.xhtml

	1	1	1	1	1	1
1						
0	0	0	0	0	0	0
	621144	3809	34450	206	35201	73762
2846						
	1					
	0					
	621144					
7.8%						
7.8%						
7.8%						
	100.0%	100.0%		100.0%	100.0%	100.0%
7.8%						
7.8%						
7.8%						
	100.0%	100.0%		100.0%	100.0%	100.0%

For peer review only

100.0%

For peer review only

Beat Parkinso	Beating Bowe	Behcets Synd	Bipolar Scotla	Bipolar UK	Birdshot Uvei	Bladder and Bo
1	42	1	3	3	1	38
0	0	0	0	0	0	0
1	1	1	1	1	1	1
0	0	0	0	0	0	0
1	1	1	1	1	1	1
	1		1	1		1
	1					1
1	1	1	1	1	1	1
1	42	1	3	3	1	38
	1		1	1		1
	42		3	3		38
	1					1
	42					38

For peer review only

For peer review only

100.0%	100.0%	100.0%	100.0%	100.0%	100.0%	100.0%
	100.0%		100.0%	100.0%		100.0%
	100.0%					100.0%
5050	342673	20281	3194	4145	10000	2271217
0	0	0	0	0	0	0
1	42	1	3	3	1	38
0	0	0	0	0	0	0
1	1	1	1	1	1	1
0	0	0	0	0	0	0
1	1	1	1	1	1	1
	1					1
						1

1	1	1	1	1	1	1
0	0	0	0	0	0	0
5050	342673	20281	3194	4145	10000	2271217
	1					1
	0					0
	342673					2271217
						1
						0
						2271217
100.0%	100.0%	100.0%	100.0%	100.0%	100.0%	100.0%
100.0%	100.0%	100.0%	100.0%	100.0%	100.0%	100.0%

For peer review only

100.0% 100.0%

100.0%

For peer review only

Bladder Healt	Bliss	Blood Pressui	Bloodwise	Bluebell Foun	Body and Sou	Bone Cancer I
17	13	1	19	2	6	3
13	6	0	1	0	0	0
1	1	1	1	1	1	1
1	1	0	1	0	0	0
1	1	1	1	1	1	1
1	1		1	1	1	1
1	1		1			
1	1	1	1	1	1	1
4	7	1	18	2	6	3
1	1		1	1	1	1
4	7		18	2	6	3
1	1		1			
4	7		18			

For peer review only

23.5%						
		100.0%		100.0%	100.0%	100.0%
23.5%						
				100.0%	100.0%	100.0%
23.5%						
76682	204923	10521	197710	776	97946	12161
0	0	0	0	0	0	0
16	13	1	19	2	6	3
0	0	0	0	0	0	0
1	1	1	1	1	1	1
0	0	0	0	0	0	0
1	1	1	1	1	1	1
	1		1			

For peer review only

1	1	1	1	1	1	1
0	0	0	0	0	0	0
76682	204923	10521	197710	776	97946	12161
	1		1			
	0		0			
	204923		197710			
100.0%	100.0%	100.0%	100.0%	100.0%	100.0%	100.0%
100.0%	100.0%	100.0%	100.0%	100.0%	100.0%	100.0%

For peer review only

100.0%	100.0%

For peer review only

Bowel Cancer	Brain Charity	Brain Tumor (	Brain Tumour	Breakthrough	Breast Cance	Breast Cancer
16	1	1	1	16	7	28
13	0	0	0	3	0	19
1	1	1	1	1	1	1
1	0	0	0	1	0	1
1	1	1	1	1	1	1
1				1	1	1
1				1		1
1	1	1	1	1	1	1
3	1	1	1	13	7	9
1				1	1	1
3				13	7	9
1				1		1
3				13		9

For peer review only

18.8%						
18.8%						32.1%
	100.0%	100.0%	100.0%		100.0%	
18.8%						
18.8%						32.1%
					100.0%	
18.8%						
18.8%						32.1%
109272	981	12676	262	109107	81316	332296
0	0	0	0	0	0	369592
16	1	1	1	16	6	25
0	0	0	0	0	0	14
1	1	1	1	1	1	1
0	0	0	0	0	0	1
1	1	1	1	1	1	1
1				1		1

For peer review only

1	1	1	1	1	1	
						1
0	0	0	0	0	0	0
109272	981	12676	262	109107	81316	
						37296
1				1		
						1
0				0		0
109272				109107		
						37296
						10.1%
						10.1%
100.0%	100.0%	100.0%	100.0%	100.0%	100.0%	
						10.1%
						10.1%
100.0%	100.0%	100.0%	100.0%	100.0%	100.0%	
						10.1%

For peer review only

10.1%

100.0% 100.0%

For peer review only

Breast Cancer	Breast Cancer N	British Dupuy	British Heart	British Liver T	British Lung Fo	British Obesit
5	4	8	7	29	50	1
0	1	0	0	5	23	0
1	1	1	1	1	1	1
0	1	0	0	1	1	0
1	1	1	1	1	1	1
1	1	1	1	1	1	
				1	1	
1	1	1	1	1	1	1
5	3	8	7	24	27	1
1	1	1	1	1	1	
5	3	8	7	24	27	
				1	1	
				24	27	

For peer review only

100.0%		100.0%	100.0%			100.0%
100.0%		100.0%	100.0%			
47894	3506763	55592	1882	229800	574095	421
0	10000000	0	0	0	1139917	0
5	2	8	7	29	47	1
0	1	0	0	0	22	0
1	1	1	1	1	1	1
0	1	0	0	0	1	0
1	1	1	1	1	1	1
	1			1	1	
	1				1	

For peer review only

1		1	1	1		1
	1				1	
0	0	0	0	0	0	0
47894		55592	1882	229800		421
	6493237				565821	
				1		
	1				1	
	0			0	0	
				229800		
	6493237				565821	
	1				1	
	0				0	
	6493237				565821	
					49.6%	
100.0%		100.0%	100.0%	100.0%		100.0%
					49.6%	
100.0%		100.0%	100.0%	100.0%		100.0%

For peer review only

49.6%

100.0%

49.6%

For peer review only

British Polio F	British Pregna	British Skin F	British Thyroi	Brittle Bone S	Brook	Building for tl
1	5	6	1	1	12	1
0	2	0	5	2	0	0
1	1	1	1	1	1	1
0	1	0	1	1	0	0
1	1	1	1	1	1	1
	1	1	1	1	1	
					1	
1	1	1			1	1
			1	1		
1	3	6			12	1
			4	1		
	1	1			1	
			1	1		
	3	6			12	
			4	1		
					1	
					12	

For peer review only

For peer review only

100.0%		100.0%			100.0%	100.0%
		100.0%			100.0%	
					100.0%	
0	127999	9785	250	15428	95962	243
0	302001	0	44574	20000	0	0
0	5	6	1	1	12	1
0	2	0	4	1	0	0
0	1	1	1	1	1	1
0	1	0	1	1	0	0
	1	1	1	1	1	1
	1					

FALSE						
		1			1	1
	1		1	1		
	0	0	0	0	0	0
		9785			95962	243
	174002		44324	4572		
	1					
	0					
	174002					
				22.9%		
		100.0%			100.0%	100.0%
				22.9%		
		100.0%			100.0%	100.0%

For peer review only

For peer review only

Butterfly Thy	Cambridge Ca	Cambridge Ra	Cancer 52	Cancer Black	Cancer Focus	Cancer Researc
3	1	4	26	2	13	57
9	2	0	0	4	0	0
1	1	1	1	1	1	1
1	1	0	0	1	0	0
1	1	1	1	1	1	1
1	1	1	1	1	1	1
			1		1	1
		1	1		1	1
1	1			1		
		4	26		13	57
6	1			2		
		1	1		1	1
1	1			1		
		4	26		13	57
6	1			2		
			1		1	1
			26		13	57

For peer review only

		100.0%	100.0%		100.0%	100.0%
		100.0%	100.0%		100.0%	100.0%
			100.0%		100.0%	100.0%
32775	507	9320	249099	9615	29164	1817635
115983	610	0	0	14876	0	0
3	1	4	26	2	12	56
9	2	0	0	3	0	0
1	1	1	1	1	1	1
1	1	0	0	1	0	0
1	1	1	1	1	1	1
1			1			1
						1

For peer review only

		1	1		1	1
1	1			1		
0	0	0	0	0	0	0
		9320	249099		29164	1817635
83208	103			5260		
			1			1
1						
0			0			0
			249099			1817635
83208						
						1
						0
						1817635
	16.9%					
	16.9%			35.4%		
		100.0%	100.0%		100.0%	100.0%
	16.9%					
	16.9%			35.4%		
		100.0%	100.0%		100.0%	100.0%

For peer review only

100.0%	100.0%
	100.0%

For peer review only

Candlelighter	Cara Trust	Cardiac Risk i	Cardiomyopa	Catholics for /	Centre for All	Changing Fac
1	5	7	5	1	1	8
0	5	0	0	0	4	3
1	1	1	1	1	1	1
0	1	0	0	0	1	1
1	1	1	1	1	1	1
	1	1	1		1	1
	1					
1		1	1	1		1
					1	
1		7	5	1		5
					3	
	1					
		1	1			1
					1	
		7	5			5
					3	

For peer review only

	0.0%					
	0.0%					
	0.0%					
100.0%		100.0%	100.0%	100.0%		
	0.0%					
	0.0%					
	0.0%					
		100.0%	100.0%			
0	15570	6271	2338	4089	253	32753
0	20523	0	0	0	303	10000
0	5	7	5	1	1	8
0	4	0	0	0	1	1
0	1	1	1	1	1	1
0	1	0	0	0	1	1
	1	1	1	1	1	1

For peer review only

FALSE						
		1	1	1		1
	1				1	
	0	0	0	0	0	0
		6271	2338	4089		22753
	4953				51	
					16.7%	
	24.1%				16.7%	
		100.0%	100.0%	100.0%		
					16.7%	
	24.1%				16.7%	
		100.0%	100.0%	100.0%		

For peer review only

For peer review only

Charlie Walle	Child Growth	Children Livin	Children with	Children with	Children's Bo	Children's Liv
1	9	9	1	2	4	2
0	3	14	1	0	9	0
1	1	1	1	1	1	1
0	1	1	1	0	1	0
1	1	1	1	1	1	1
	1	1		1	1	1
		1				
			1			
1	1			1		1
		1			1	
1	6			2		2
		5			5	
	1			1		1
		1			1	
	6			2		2
		5			5	
		1				
		5				

For peer review only

			0.0%			
			0.0%			
		35.7%	0.0%			
100.0%				100.0%		100.0%
		35.7%				
				100.0%		100.0%
		35.7%				
1000	14236	31845	20180	625	33966	16010
0	0	23564	20099	0	32665	0
1	9	9	1	2	4	2
0	0	8	1	0	2	0
1	1	1	1	1	1	1
0	0	1	1	0	1	0
1	1	1	1	1	1	1

For peer review only

1	1	1	1	1	1	1
0	0	0	0	0	0	0
1000	14236	8282	81	625	1300	16010
			0.4%		3.8%	
			0.4%		3.8%	
		26.0%	0.4%		3.8%	
100.0%	100.0%			100.0%		100.0%
			0.4%		3.8%	
			0.4%		3.8%	
		26.0%	0.4%		3.8%	
100.0%	100.0%			100.0%		100.0%

For peer review only

For peer review only

Children’s He	Children’s HI\	Chinese Natic	Chronic Lymp	Chronic Myel	Cleft Lip and I	CLIC Sargent (
1	10	1	12	26	2	3
0	13	0	0	16	0	0
1	1	1	1	1	1	1
0	1	0	0	1	0	0
1	1	1	1	1	1	1
	1		1	1	1	1
	1		1	1		
1		1	1	1	1	1
	1					
1		1	12	10	2	3
	3					
			1	1	1	1
	1					
			12	10	2	3
	3					
			1	1		
	1					
			12	10		
	3					

For peer review only

	23.1%			38.5%		
100.0%		100.0%	100.0%		100.0%	100.0%
	23.1%			38.5%		
			100.0%		100.0%	100.0%
	23.1%			38.5%		
			100.0%			
736	49078	253	86711	180581	121	5412
0	0	0	0	185184	0	0
1	10	1	12	25	2	3
0	0	0	0	16	0	0
1	1	1	1	1	1	1
0	0	0	0	1	0	0
1	1	1	1	1	1	1
				1		

For peer review only

1	1	1	1		1	1
				1		
0	0	0	0	0	0	0
736	49078	253	86711		121	5412
				4603		
				1		
				0		
				4603		
				2.5%		
				2.5%		
				2.5%		
100.0%	100.0%	100.0%	100.0%		100.0%	100.0%
				2.5%		
				2.5%		
				2.5%		
100.0%	100.0%	100.0%	100.0%		100.0%	100.0%
				2.5%		
				2.5%		

For peer review only

2.5%

For peer review only

	Coeliac UK	Community C	Community H	Community L	Confederatio	Contact a Fan	Coping with C
	6	1	1	1	2	3	1
	2	0	1	2	2	0	0
	1	1	1	1	1	1	1
	1	0	1	1	1	0	0
	1	1	1	1	1	1	1
	1			1	1	1	
			1		1		
	1	1				1	1
				1			
	4	1				3	1
				1			
					1		
	1					1	
				1			
	4					3	
				1			

For peer review only

For peer review only

		0.0%		0.0%		
		0.0%		0.0%		
		0.0%		0.0%		
	100.0%				100.0%	100.0%
				0.0%		
				0.0%		
				0.0%		
					100.0%	
33029	1029	3042	6060	72000	20369	4187
32003	0	3042	10051	0	0	0
6	1	1	1	2	3	1
2	0	1	2	0	0	0
1	1	1	1	1	1	1
1	0	1	1	0	0	0
1	1	1	1	1	1	1

		1				
1	1			1	1	1
			1			
0	0	0	0	0	0	0
1027	1029			72000	20369	4187
			3991			
3.1%		0.0%				
3.1%		0.0%				
3.1%		0.0%	39.7%			
	100.0%			100.0%	100.0%	100.0%
3.1%		0.0%				
3.1%		0.0%				
3.1%		0.0%	39.7%			
	100.0%			100.0%	100.0%	100.0%

For peer review only

For peer review only

Coppafeel!	Core Charity	Crohn's and C	Crohn's in Chi	Cure and Acti	Cure Parkinso	Cystic Fibrosis
1	5	49	9	2	9	17
1	3	35	0	1	0	0
1	1	1	1	1	1	1
1	1	1	0	1	0	0
1	1	1	1	1	1	1
	1	1	1	1	1	1
		1				1
1						
	1	1	1	1	1	1
	2	14	9	1	9	17
	1	1	1	1	1	1
	2	14	9	1	9	17
		1				1
		14				17

For peer review only

0.0%						
0.0%						
0.0%	40.0%	28.6%				
			100.0%		100.0%	100.0%
	40.0%	28.6%				
			100.0%		100.0%	100.0%
		28.6%				
						100.0%
10000	44609	593275	27581	120	24749	281197
10100	0	0	0	9447	0	0
1	5	49	9	2	9	17
1	0	0	0	1	0	0
1	1	1	1	1	1	1
1	0	0	0	1	0	0
1	1	1	1	1	1	1
		1				1

For peer review only

	1	1	1		1	1
1				1		
0	0	0	0	0	0	0
	44609	593275	27581		24749	281197
100				9327		
		1				1
		0				0
		593275				281197
1.0%						
1.0%						
1.0%						
	100.0%	100.0%	100.0%		100.0%	100.0%
1.0%						
1.0%						
1.0%						
	100.0%	100.0%	100.0%		100.0%	100.0%

For peer review only

100.0% 100.0%

For peer review only

David Lewis C	Deaf Educatic	Delete Blood	Dementia UK	Dermatrust	Diabetes Rese	Diabetes UK
1	1	3	1	1	3	210
0	0	0	0	2	0	11
1	1	1	1	1	1	1
0	0	0	0	1	0	1
1	1	1	1	1	1	1
		1		1	1	1
						1
						1
1	1	1	1		1	1
				1		
1	1	3	1		3	199
				1		
		1			1	1
				1		
		3			3	199
				1		
						1
						199

For peer review only

						1
						199
100.0%	100.0%	100.0%	100.0%		100.0%	
		100.0%			100.0%	
1892	526	14665	2593	29823	6595	3050180
0	0	0	0	45823	0	0
1	1	3	1	1	3	209
0	0	0	0	2	0	0
1	1	1	1	1	1	1
0	0	0	0	1	0	0
1	1	1	1	1	1	1
						1
						1

For peer review only

1	1	1	1	1	1	1
0	0	0	0	0	0	0
1892	526	14665	2593	16000	6595	3050180
						1
						0
						3050180
						1
						0
						3050180
100.0%	100.0%	100.0%	100.0%	34.9%	100.0%	100.0%
100.0%	100.0%	100.0%	100.0%	34.9%	100.0%	100.0%

For peer review only

100.0%

100.0%

For peer review only

Diabetics witl	Diamond Blac	Different Stro	Disabled Livir	Douglas Bade	Edinburgh an	Education for
4	1	5	6	2	2	3
0	0	0	2	0	0	8
1	1	1	1	1	1	1
0	0	0	1	0	0	1
1	1	1	1	1	1	1
1		1	1	1	1	1
1	1	1	1	1	1	
						1
4	1	5	4	2	2	
						5
1		1	1	1	1	
						1
4		5	4	2	2	
						5

For peer review only

100.0%	100.0%	100.0%		100.0%	100.0%	
100.0%		100.0%		100.0%	100.0%	
1547	15150	10787	23840	1384	804	5343
0	0	0	0	0	0	45921
4	1	5	6	2	2	3
0	0	0	0	0	0	3
1	1	1	1	1	1	1
0	0	0	0	0	0	1
1	1	1	1	1	1	1

For peer review only

1	1	1	1	1	1	
						1
0	0	0	0	0	0	0
1547	15150	10787	23840	1384	804	
						40578
100.0%	100.0%	100.0%	100.0%	100.0%	100.0%	
100.0%	100.0%	100.0%	100.0%	100.0%	100.0%	

For peer review only

For peer review only

Encephalitis S	Endometriosi	Epilepsy Actic	Epilepsy Conr	Epilepsy Scotl	Epilepsy Soci	European Par
2	2	9	2	7	18	4
0	5	7	0	0	15	11
1	1	1	1	1	1	1
0	1	1	0	0	1	1
1	1	1	1	1	1	1
1	1	1	1	1	1	1
					1	1
1		1	1	1	1	
	1					1
2		2	2	7	3	
	3					7
1		1	1	1	1	
	1					1
2		2	2	7	3	
	3					7
					1	
						1
					3	
						7

For peer review only

					16.7%	
		22.2%			16.7%	
100.0%			100.0%	100.0%		
					16.7%	
		22.2%			16.7%	
100.0%			100.0%	100.0%		
					16.7%	
					16.7%	
767	21096	92855	4085	6117	188332	64681
0	21096	199382	0	0	111712	239475
2	2	9	2	7	18	4
0	2	6	0	0	3	7
1	1	1	1	1	1	1
0	1	1	0	0	1	1
1	1	1	1	1	1	1
		1			1	1

For peer review only

	1					
1			1	1	1	
		1				1
0	0	0	0	0	0	0
767			4085	6117	76620	
		106527				174794
					1	
		1				1
		0			0	0
					76620	
		106527				174794
	0.0%					
	0.0%					
	0.0%				40.7%	
100.0%			100.0%	100.0%		
	0.0%					
	0.0%					
	0.0%				40.7%	
100.0%			100.0%	100.0%		

For peer review only

40.7%

For peer review only

Family Planni	Fertility Netw	Findacure	Food Chain	FORCE	Forum Link	Foundation fc
12	17	6	3	1	6	1
1	17	7	1	0	0	1
1	1	1	1	1	1	1
1	1	1	1	0	0	1
1	1	1	1	1	1	1
1	1	1	1		1	
1	1					
	1					1
1			1	1	1	
		1				
11			2	1	6	
		1				
	1					
1			1		1	
		1				
11			2		6	
		1				
	1					
1						
11						

For peer review only

	0.0%					0.0%
	0.0%	14.3%				0.0%
	0.0%	14.3%				0.0%
				100.0%	100.0%	
	0.0%					
	0.0%	14.3%				
	0.0%	14.3%				
					100.0%	
	0.0%					
	0.0%					
	0.0%					
107247	189815	27591	13092	507	26164	57537
15150	56317	46176	5000	0	0	0
11	17	6	3	1	6	1
1	5	7	1	0	0	0
1	1	1	1	1	1	1
1	1	1	1	0	0	0
1	1	1	1	1	1	1
1	1					

For peer review only

1	1		1	1	1	1
		1				
0	0	0	0	0	0	0
92097	133497		8092	507	26164	57537
		18585				
1	1					
0	0					
92097	133497					
		40.2%				
				100.0%	100.0%	100.0%
		40.2%				
				100.0%	100.0%	100.0%

For peer review only

For peer review only

Freshwinds	Friends of FO	Fungal Infecti	Gauchers Ass	Gay Men's He	Genetic Allian	George House
2	1	8	29	13	67	2
0	1	11	6	3	86	1
1	1	1	1	1	1	1
0	1	1	1	1	1	1
1	1	1	1	1	1	1
1		1	1	1	1	1
		1	1	1	1	
	1					
1			1	1		1
		1			1	
2			23	10		1
		3			19	
1			1	1		1
		1			1	
2			23	10		1
		3			19	
			1	1		
		1			1	
			23	10		
		3			19	

For peer review only

	0.0%					
	0.0%					
	0.0%	27.3%			22.1%	
100.0%						
		27.3%			22.1%	
100.0%						
		27.3%			22.1%	
11930	0	264961	182227	93592	616355	21000
0	0	307187	0	69319	235212	1000
2	0	8	29	13	66	2
0	0	10	0	3	12	1
1	0	1	1	1	1	1
0	0	1	0	1	1	1
1		1	1	1	1	1
		1	1		1	

For peer review only

	FALSE					
1			1	1	1	1
		1				
0		0	0	0	0	0
11930			182227	24273	381142	20000
		42226				
			1		1	
		1				
		0	0		0	
			182227		381142	
		42226				
		13.7%				
		13.7%		25.9%		
100.0%			100.0%			
		13.7%				
		13.7%		25.9%		
100.0%			100.0%			
		13.7%				

For peer review only

13.7%

100.0%

For peer review only

GIST Support	Groundswell	Group B Stre	Guillain Barre	Haemophilia	Haemophilia	Haemophilia
21	1	1	2	17	57	10
6	1	0	0	2	0	0
1	1	1	1	1	1	1
1	1	0	0	1	0	0
1	1	1	1	1	1	1
1			1	1	1	1
1				1	1	
	1					
1		1	1	1	1	1
15		1	2	15	57	10
1			1	1	1	1
15			2	15	57	10
1				1	1	
15				15	57	

For peer review only

	0.0%					
	0.0%					
	0.0%					
		100.0%	100.0%		100.0%	100.0%
			100.0%		100.0%	100.0%
					100.0%	
103706	3042	154	6400	47143	577522	13721
53906	0	0	0	25605	0	0
21	1	1	2	16	56	10
6	0	0	0	2	0	0
1	1	1	1	1	1	1
1	0	0	0	1	0	0
1	1	1	1	1	1	1
1					1	

1	1	1	1	1	1	1
0	0	0	0	0	0	0
49800	3042	154	6400	21538	577522	13721
1					1	
0					0	
49800					577522	
48.0%				45.7%		
	100.0%	100.0%	100.0%		100.0%	100.0%
48.0%				45.7%		
	100.0%	100.0%	100.0%		100.0%	100.0%

For peer review only

48.0%

100.0%

For peer review only

Hafal	Headway	Health and Sc	Heart of Mers	Heart UK	Heartbeat	Hepatitis B Pc
1	1	1	2	94	1	1
0	0	0	2	5	0	0
1	1	1	1	1	1	1
0	0	0	1	1	0	0
1	1	1	1	1	1	1
			1	1		
				1		
			1			
1	1	1		1	1	1
1	1	1		89	1	1
			1			
				1		
				89		
				1		
				89		

For peer review only

			0.0%			
			0.0%			
			0.0%			
100.0%	100.0%	100.0%			100.0%	100.0%
			0.0%			
			0.0%			
			0.0%			
4000	150	38	31563	909013	758	5070
0	0	0	15781	0	0	0
1	1	1	2	90	1	1
0	0	0	1	0	0	0
1	1	1	1	1	1	1
0	0	0	1	0	0	0
1	1	1	1	1	1	1
				1		

For peer review only

1	1	1	1	1	1	1
0	0	0	0	0	0	0
4000	150	38	15781	909013	758	5070
				1		
				0		
				909013		
100.0%	100.0%	100.0%		100.0%	100.0%	100.0%
100.0%	100.0%	100.0%		100.0%	100.0%	100.0%

For peer review only

100.0%

For peer review only

Hepatitis C Tr	Hereditary Ar	Herts Aid	Hidradenitis S	HIV i-Base	HIV Scotland	Hughes Syndr
84	15	4	7	13	3	1
25	6	2	2	0	3	0
1	1	1	1	1	1	1
1	1	1	1	0	1	0
1	1	1	1	1	1	1
1	1	1	1	1	1	
1	1			1		
					1	
1	1	1	1	1		1
59	9	2	5	13		1
					1	
1	1	1	1	1		
59	9	2	5	13		
1	1			1		
59	9			13		

For peer review only

					0.0%	
					0.0%	
					0.0%	
				100.0%		100.0%
					0.0%	
					0.0%	
					0.0%	
				100.0%		
				100.0%		
1022603	200669	11529	32910	63462	23200	2104
1566080	194617	8050	25750	0	20509	0
83	15	4	7	13	3	1
25	6	2	2	0	3	0
1	1	1	1	1	1	1
1	1	1	1	0	1	0
1	1	1	1	1	1	1
1	1					
1						

	1	1	1	1	1	1
1						
0	0	0	0	0	0	0
	6051	3479	7160	63462	2691	2104
543477						
	1					
1						
0	0					
	6051					
543477						
1						
0						
543477						
	3.0%					
	3.0%				11.6%	
34.7%	3.0%	30.2%	21.8%		11.6%	
				100.0%		100.0%
	3.0%					
	3.0%				11.6%	
34.7%	3.0%	30.2%	21.8%		11.6%	
				100.0%		100.0%
	3.0%					
	3.0%					

For peer review only

34.7%	3.0%
34.7%	

For peer review only

**Huntington's**	**IBS Network**	**ICUsteps**	**Immune Thro**	**Imperial Colle**	**Independent**	**Input Diabete**
1	1	2	15	1	11	1
3	4	0	29	0	0	0
1	1	1	1	1	1	1
1	1	0	1	0	0	0
1	1	1	1	1	1	1
1	1	1	1		1	
			1		1	
		1		1	1	1
1	1		1			
		2		1	11	1
2	3		14			
		1			1	
1	1		1			
		2			11	
2	3		14			
					1	
			1			
					11	
			14			

For peer review only

			48.3%			
		100.0%		100.0%	100.0%	100.0%
			48.3%			
		100.0%			100.0%	
			48.3%			
					100.0%	
30159	8417	4157	67352	4000	29891	30322
0	0	0	44323	0	0	0
1	1	2	15	1	11	1
0	0	0	8	0	0	0
1	1	1	1	1	1	1
0	0	0	1	0	0	0
1	1	1	1	1	1	1

For peer review only

1	1	1	1	1	1	1
0	0	0	0	0	0	0
30159	8417	4157	23028	4000	29891	30322
			34.2%			
100.0%	100.0%	100.0%		100.0%	100.0%	100.0%
			34.2%			
100.0%	100.0%	100.0%		100.0%	100.0%	100.0%

For peer review only

For peer review only

International	International	International	International	International I	Jo's Cervical (	Joint Epilepsy
23	2	9	1	10	2	1
39	1	19	8	31	14	4
1	1	1	1	1	1	1
1	1	1	1	1	1	1
1	1	1	1	1	1	1
1	1	1	1	1	1	1
1		1		1	1	
	1					
1		1	1	1	1	1
	1					
16		10	7	21	12	3
	1					
1		1	1	1	1	1
	1					
16		10	7	21	12	3
1		1		1	1	
16		10		21	12	

For peer review only

41.0%

41.0%

41.0%

652377	73355	24458	18514	222272	25150	12856
873965	0	0	0	1681140	36360	0
23	2	9	1	10	2	1
39	0	0	0	31	2	0
1	1	1	1	1	1	1
1	0	0	0	1	1	0
1	1	1	1	1	1	1
1				1		
				1		

For peer review only

1	1	1	1	1	1	1
0	0	0	0	0	0	0
221588	73355	24458	18514	1458868	11210	12856
1				1		
0				0		
221588				1458868		
				1		
				0		
				1458868		
25.4%	100.0%	100.0%	100.0%		30.8%	100.0%
25.4%	100.0%	100.0%	100.0%		30.8%	100.0%

For peer review only

For peer review only - http://bmjopen.bmj.com/site/about/guidelines.xhtml

25.4%

For peer review only

Patient organisation names						
Juvenile Diab	KAITY	Karen Clifford	Kent Associat	Kidney Cancer	Kidney Cancer	Kidney Care U
19	1	10	1	9	37	2
6	0	0	2	0	0	0
1	1	1	1	1	1	1
1	0	0	1	0	0	0
1	1	1	1	1	1	1
1		1	1	1	1	1
1					1	
1	1	1		1	1	1
			1			
13	1	10		9	37	2
			1			
1		1		1	1	1
			1			
13		10		9	37	2
			1			
1					1	
13					37	

For peer review only

For peer review only

	100.0%	100.0%		100.0%	100.0%	100.0%
		100.0%		100.0%	100.0%	100.0%
					100.0%	
417065	2000	91665	18513	101336	183714	754
10563	0	0	18938	0	0	0
19	1	9	1	8	35	2
1	0	0	1	0	0	0
1	1	1	1	1	1	1
1	0	0	1	0	0	0
1	1	1	1	1	1	1
1				1	1	

1	1	1		1	1	1
			1			
0	0	0	0	0	0	0
406503	2000	91665		101336	183714	754
			424			
1				1	1	
0				0	0	
406503				101336	183714	
			2.2%			
			2.2%			
			2.2%			
	100.0%	100.0%		100.0%	100.0%	100.0%
			2.2%			
			2.2%			
			2.2%			
	100.0%	100.0%		100.0%	100.0%	100.0%

For peer review only

100.0%	100.0%

For peer review only

Kidney Resear	KIDS	KwaAfrica	LATCH Welsh	Laurence Mo	Leeds Teachir	Leicestershire
17	1	1	1	1	1	4
47	1	1	0	1	0	0
1	1	1	1	1	1	1
1	1	1	0	1	0	0
1	1	1	1	1	1	1
1						1
1						
	1	1		1		
			1		1	1
1						
			1		1	4
30						
						1
1						
						4
30						
1						
30						

For peer review only

	0.0%	0.0%		0.0%		
	0.0%	0.0%		0.0%		
	0.0%	0.0%		0.0%		
			100.0%		100.0%	100.0%
						100.0%
178461	7676	5000	1010	1010	354	22216
3144648	7676	5000	0	0	0	0
17	1	1	1	1	1	4
47	1	1	0	0	0	0
1	1	1	1	1	1	1
1	1	1	0	0	0	0
1	1	1	1	1	1	1
1						
1						

For peer review only

	1	1				
			1	1	1	1
1						
0	0	0	0	0	0	0
			1010	1010	354	22216
2966187						
1						
0						
2966187						
1						
0						
2966187						
	0.0%	0.0%				
	0.0%	0.0%				
	0.0%	0.0%				
			100.0%	100.0%	100.0%	100.0%
	0.0%	0.0%				
	0.0%	0.0%				
	0.0%	0.0%				
			100.0%	100.0%	100.0%	100.0%

For peer review only

For peer review only

Leukaemia CA	Life Educatio	Little Hearts	Liver4Life	Lullaby Trust	Lupus Europe	Lupus UK
62	1	1	24	1	4	1
39	0	0	0	0	0	0
1	1	1	1	1	1	1
1	0	0	0	0	0	0
1	1	1	1	1	1	1
1			1		1	
1			1			
1	1	1	1	1	1	1
23	1	1	24	1	4	1
1			1		1	
23			24		4	
1			1			
23			24			

For peer review only

37.1%						
	100.0%	100.0%	100.0%	100.0%	100.0%	100.0%
37.1%						
			100.0%		100.0%	
37.1%						
			100.0%			
472981	519	253	248299	0	55503	707
351393	0	0	0	0	0	0
61	1	1	24	0	4	1
30	0	0	0	0	0	0
1	1	1	1	0	1	1
1	0	0	0	0	0	0
1	1	1	1		1	1
1			1			

For peer review only

				FALSE		
1	1	1	1		1	1
0	0	0	0		0	0
121588	519	253	248299		55503	707
1			1			
0			0			
121588			248299			
25.7%						
	100.0%	100.0%	100.0%		100.0%	100.0%
25.7%						
	100.0%	100.0%	100.0%		100.0%	100.0%

For peer review only

25.7%

100.0%

For peer review only

Lymphoma As	Macmillan Ca	Macular Socie	Maggie's Cen	Make A Wish	Malnutrition	MAMA Acade
58	50	1	3	2	1	1
84	2	0	0	0	0	0
1	1	1	1	1	1	1
1	1	0	0	0	0	0
1	1	1	1	1	1	1
1	1		1	1		
1	1					
	1	1	1	1	1	1
1						
	48	1	3	2	1	1
26						
	1		1	1		
1						
	48		3	2		
26						
	1					
1						
	48					
26						

For peer review only

31.0%						
		100.0%	100.0%	100.0%	100.0%	100.0%
31.0%						
			100.0%	100.0%		
31.0%						
158813	190660	15	3413	765	884	202
31792	0	0	0	0	0	0
57	50	1	3	2	1	1
9	0	0	0	0	0	0
1	1	1	1	1	1	1
1	0	0	0	0	0	0
1	1	1	1	1	1	1
1	1					

For peer review only

1	1	1	1	1	1	1
0	0	0	0	0	0	0
127022	190660	15	3413	765	884	202
1	1					
0	0					
127022	190660					
	100.0%	100.0%	100.0%	100.0%	100.0%	100.0%
	100.0%	100.0%	100.0%	100.0%	100.0%	100.0%

For peer review only

100.0%

For peer review only

Manchester A	Marie Curie C	Martin Fisher	MDS UK Supp	Medical Foun	Melanoma Fo	Melanoma Fu
3	3	1	13	5	4	3
2	0	0	6	3	1	1
1	1	1	1	1	1	1
1	0	0	1	1	1	1
1	1	1	1	1	1	1
1	1		1	1	1	1
			1			
1	1	1	1	1	1	1
1	3	1	7	2	3	2
1	1		1	1	1	1
1	3		7	2	3	2
			1			
			7			

For peer review only

33.3%				40.0%		
	100.0%	100.0%				
33.3%				40.0%		
	100.0%					
19538	643	15000	145332	58641	61418	23080
9438	0	0	131702	63480	0	5050
3	3	1	13	5	4	3
2	0	0	6	3	0	1
1	1	1	1	1	1	1
1	0	0	1	1	0	1
1	1	1	1	1	1	1
			1			

For peer review only

1	1	1	1		1	1
				1		
0	0	0	0	0	0	0
10100	643	15000	13630		61418	18030
				4840		
			1			
			0			
			13630			
			9.4%	7.6%		
			9.4%	7.6%		
			9.4%	7.6%		
	100.0%	100.0%			100.0%	
			9.4%	7.6%		
			9.4%	7.6%		
			9.4%	7.6%		
	100.0%	100.0%			100.0%	
			9.4%			
			9.4%			

For peer review only

9.4%

For peer review only

Melanoma U	Men's Health	Meningitis N	Meningitis R	Mental Healt	Mesotheliom	Migraine Acti
24	14	33	60	8	7	8
0	5	20	13	1	0	1
1	1	1	1	1	1	1
0	1	1	1	1	0	1
1	1	1	1	1	1	1
1	1	1	1	1	1	1
1	1	1	1			
1	1	1	1	1	1	1
24	9	13	47	7	7	7
1	1	1	1	1	1	1
24	9	13	47	7	7	7
1	1	1	1			
24	9	13	47			

For peer review only

For peer review only

		39.4%				
100.0%					100.0%	
		39.4%				
100.0%					100.0%	
		39.4%				
100.0%						
386833	94419	532482	700445	168898	34571	107846
0	64124	266721	216480	5143	0	0
24	11	32	59	8	7	8
0	5	16	8	1	0	0
1	1	1	1	1	1	1
0	1	1	1	1	0	0
1	1	1	1	1	1	1
1		1	1	1		1

1	1	1	1	1	1	1
0	0	0	0	0	0	0
386833	30294	265761	483965	163756	34571	107846
1		1	1	1		1
0		0	0	0		0
386833		265761	483965	163756		107846
	32.1%	49.9%				
100.0%					100.0%	100.0%
	32.1%	49.9%				
100.0%					100.0%	100.0%

For peer review only

49.9%

100.0% 100.0%

For peer review only

Migraine Trus	Motor Neuro	Mouth Cance	Movember Fo	MPN Voice	Multi Organ T	Multiple Birth
9	2	1	1	6	1	1
9	0	2	0	0	0	0
1	1	1	1	1	1	1
1	0	1	0	0	0	0
1	1	1	1	1	1	1
1	1	1		1		
1						
	1		1	1	1	1
		1				
	2		1	6	1	1
		1				
1						
	1			1		
		1				
	2			6		
		1				

For peer review only

0.0%						
0.0%						
0.0%						
	100.0%		100.0%	100.0%	100.0%	100.0%
0.0%						
0.0%						
0.0%						
	100.0%			100.0%		
308512	280	1000	1010	44237	6084	505
0	0	0	0	0	0	0
9	2	1	1	6	1	1
0	0	0	0	0	0	0
1	1	1	1	1	1	1
0	0	0	0	0	0	0
1	1	1	1	1	1	1
1						

For peer review only

1	1	1	1	1	1	1
0	0	0	0	0	0	0
308512	280	1000	1010	44237	6084	505
1						
0						
308512						
100.0%	100.0%	100.0%	100.0%	100.0%	100.0%	100.0%
100.0%	100.0%	100.0%	100.0%	100.0%	100.0%	100.0%

For peer review only

100.0%

For peer review only

Multiple Scler	Multiple Scle	Multiple Scler	Multiple Scle	Muscular Dys	Myasthenia G	Myeloma UK
7	38	62	1	3	1	53
28	24	18	0	10	0	15
1	1	1	1	1	1	1
1	1	1	0	1	0	1
1	1	1	1	1	1	1
1	1	1		1		1
1	1	1				1
	1	1	1		1	1
1				1		
	14	44	1		1	38
21				7		
	1	1				1
1				1		
	14	44				38
21				7		
	1	1				1
1						
	14	44				38
21						

For peer review only

	36.8%					
			100.0%		100.0%	
	36.8%					
	36.8%					
317100	282676	533370	603	17028	2020	7430106
2524665	564884	55200	0	0	0	711530
7	38	62	1	3	1	52
28	24	5	0	0	0	15
1	1	1	1	1	1	1
1	1	1	0	0	0	1
1	1	1	1	1	1	1
1	1	1				1
1						1

For peer review only

		1	1	1	1	1
1	1					
0	0	0	0	0	0	0
		478170	603	17028	2020	6718577
2207565	282208					
		1				1
1	1					
0	0	0				0
		478170				6718577
2207565	282208					
						1
1						
0						0
						6718577
2207565						
	50.0%					
			100.0%	100.0%	100.0%	
	50.0%					
			100.0%	100.0%	100.0%	

For peer review only

50.0%

For peer review only

Myrovlytis Tr	NARA - The B	National AIDS	National AIDS	National Anky	National Assc	National Atte
1	1	41	28	55	1	9
0	0	7	17	33	0	7
1	1	1	1	1	1	1
0	0	1	1	1	0	1
1	1	1	1	1	1	1
		1	1	1		1
		1	1	1		
1	1	1	1	1	1	1
1	1	34	11	22	1	2
		1	1	1		1
		34	11	22		2
		1	1	1		
		34	11	22		

For peer review only

			39.3%	40.0%		22.2%
100.0%	100.0%				100.0%	
			39.3%	40.0%		22.2%
			39.3%	40.0%		
1420	0	454331	284499	902728	152	12006
0	0	198306	316593	527875	0	27951
1	0	41	28	54	1	9
0	0	6	17	18	0	7
1	0	1	1	1	1	1
0	0	1	1	1	0	1
1		1	1	1	1	1
		1	1	1		

For peer review only

	FALSE					
1		1		1	1	
			1			1
0		0	0	0	0	0
1420		256025		374853	152	
			32094			15945
		1		1		
			1			
		0	0	0		
		256025		374853		
			32094			
			10.1%			
			10.1%	41.5%		
100.0%					100.0%	
			10.1%			
			10.1%	41.5%		
100.0%					100.0%	
			10.1%			

For peer review only

10.1%	41.5%

For peer review only

National Canc	National Chil	National Cou	National Ecze	National Kidn	National Obe	National Oste
1	1	1	14	30	3	35
0	1	3	0	0	0	0
1	1	1	1	1	1	1
0	1	1	0	0	0	0
1	1	1	1	1	1	1
		1	1	1	1	1
			1	1		1
	1					
1			1	1	1	1
		1				
1			14	30	3	35
		2				
			1	1	1	1
		1				
			14	30	3	35
		2				
			1	1		1
			14	30		35

For peer review only

	0.0%					
	0.0%					
	0.0%					
100.0%			100.0%	100.0%	100.0%	100.0%
			100.0%	100.0%	100.0%	100.0%
			100.0%	100.0%		100.0%
1000	9600	10521	23676	396718	3508	326741
0	0	23780	0	0	0	0
1	1	1	14	30	3	34
0	0	3	0	0	0	0
1	1	1	1	1	1	1
0	0	1	0	0	0	0
1	1	1	1	1	1	1
				1		1

For peer review only

1	1		1	1	1	1
		1				
0	0	0	0	0	0	0
1000	9600		23676	396718	3508	326741
		13259				
				1		1
				0		0
				396718		326741
100.0%	100.0%		100.0%	100.0%	100.0%	100.0%
100.0%	100.0%		100.0%	100.0%	100.0%	100.0%

For peer review only

100.0%	100.0%

For peer review only

National Rheu	National Voic	NAZ	NET Patient F	Neurological /	Niemann-Pick	NMO Researc
102	12	10	28	15	12	3
20	21	4	14	31	14	0
1	1	1	1	1	1	1
1	1	1	1	1	1	0
1	1	1	1	1	1	1
1	1	1	1	1	1	1
1	1		1	1	1	
1						
1		1	1			1
	1			1	1	
82		6	14			3
	9			16	2	
1		1	1			1
	1			1	1	
82		6	14			3
	9			16	2	
1			1			
	1			1	1	
82			14			
	9			16	2	

For peer review only

1						
82						
					14.3%	
	42.9%				14.3%	
						100.0%
					14.3%	
	42.9%				14.3%	
						100.0%
					14.3%	
	42.9%				14.3%	
688957	87459	105845	196851	147434	129795	6650
0	212771	133957	269645	0	102765	0
100	12	10	27	15	12	3
0	15	4	14	0	12	0
1	1	1	1	1	1	1
0	1	1	1	0	1	0
1	1	1	1	1	1	1
1	1	1	1	1	1	

For peer review only

1				1	1	1
	1	1	1			
0	0	0	0	0	0	0
688957				147434	27030	6650
	125313	28112	72795			
1				1	1	
	1	1	1			
0	0	0	0	0	0	
688957				147434	27030	
	125313	28112	72795			
		21.0%	27.0%		20.8%	
100.0%				100.0%		100.0%
		21.0%	27.0%		20.8%	
100.0%				100.0%		100.0%

21.0% 27.0% 20.8%

100.0% 100.0%

For peer review only

North of Engl	Northern Irel	Northern Irel	Northern Neu	OcuMel UK	Oesophageal	Older People'
4	1	2	2	2	1	2
0	0	0	1	0	1	1
1	1	1	1	1	1	1
0	0	0	1	0	1	1
1	1	1	1	1	1	1
1		1	1	1		1
					1	
1	1	1	1	1		1
4	1	2	1	2		1
1		1	1	1		1
4		2	1	2		1

					0.0%	
					0.0%	
					0.0%	
100.0%	100.0%	100.0%		100.0%		
100.0%		100.0%		100.0%		
5775	1029	4035	21913	2081	1636	30300
0	0	0	21913	0	0	25351
4	1	2	1	2	1	2
0	0	0	1	0	0	1
1	1	1	1	1	1	1
0	0	0	1	0	0	1
1	1	1	1	1	1	1

For peer review only

			1			
1	1	1		1	1	1
0	0	0	0	0	0	0
5775	1029	4035		2081	1636	4949
			0.0%			
			0.0%			16.3%
			0.0%			16.3%
100.0%	100.0%	100.0%		100.0%	100.0%	
			0.0%			
			0.0%			16.3%
			0.0%			16.3%
100.0%	100.0%	100.0%		100.0%	100.0%	

For peer review only

For peer review only

Olive Tree Ca	Oliver King Fo	Orchid	Organisation	Ovacome	Ovarian Canc	Paget's Assoc
1	1	2	2	6	3	3
0	0	0	0	0	3	0
1	1	1	1	1	1	1
0	0	0	0	0	1	0
1	1	1	1	1	1	1
		1	1	1	1	1
					1	
1	1	1	1	1		1
1	1	2	2	6		3
					1	
		1	1	1		1
		2	2	6		3

For peer review only

					0.0%	
					0.0%	
					0.0%	
100.0%	100.0%	100.0%	100.0%	100.0%		100.0%
					0.0%	
					0.0%	
					0.0%	
		100.0%	100.0%	100.0%		100.0%
3400	511	3334	4184	64181	39552	24721
0	0	0	0	0	0	0
1	1	2	2	6	3	3
0	0	0	0	0	0	0
1	1	1	1	1	1	1
0	0	0	0	0	0	0
1	1	1	1	1	1	1

For peer review only

1	1	1	1	1	1	1
0	0	0	0	0	0	0
3400	511	3334	4184	64181	39552	24721
100.0%	100.0%	100.0%	100.0%	100.0%	100.0%	100.0%
100.0%	100.0%	100.0%	100.0%	100.0%	100.0%	100.0%

For peer review only

For peer review only

Pain Associati	Pain Concern	Pain Relief Fo	Pain UK	Pancreatic Ca	Pancreatic Ca	Parkinson’s U
10	11	3	10	7	17	12
0	0	2	0	2	3	8
1	1	1	1	1	1	1
0	0	1	0	1	1	1
1	1	1	1	1	1	1
1	1	1	1	1	1	1
	1				1	1
1	1	1	1	1	1	1
10	11	1	10	5	14	4
1	1	1	1	1	1	1
10	11	1	10	5	14	4
	1				1	1
	11				14	4

For peer review only

		33.3%				33.3%
100.0%	100.0%		100.0%			
		33.3%				33.3%
100.0%	100.0%		100.0%			
						33.3%
	100.0%					
4636	16888	1442	30101	34907	209550	29736
0	0	0	0	0	0	15781
9	9	3	10	7	17	11
0	0	0	0	0	0	2
1	1	1	1	1	1	1
0	0	0	0	0	0	1
1	1	1	1	1	1	1
					1	

For peer review only

1	1	1	1	1	1	1
0	0	0	0	0	0	0
4636	16888	1442	30101	34907	209550	13955
					1	
					0	
					209550	
						46.9%
100.0%	100.0%	100.0%	100.0%	100.0%	100.0%	
						46.9%
100.0%	100.0%	100.0%	100.0%	100.0%	100.0%	

For peer review only

100.0%

For peer review only

Patients Asso	Patients On Ir	Paula Carr Di	PBC Foundati	Pelican Cance	Pelvic Pain Su	Pink Ribbon F
48	5	1	6	1	4	1
0	0	0	3	0	0	0
1	1	1	1	1	1	1
0	0	0	1	0	0	0
1	1	1	1	1	1	1
1	1		1		1	
1						
1	1	1	1	1	1	1
48	5	1	3	1	4	1
1	1		1		1	
48	5		3		4	
1						
48						

For peer review only

100.0%	100.0%	100.0%		100.0%	100.0%	100.0%
100.0%	100.0%				100.0%	
100.0%						
345520	29849	400	17418	8500	2655	10000
0	0	0	15150	0	0	0
45	5	1	6	1	4	1
0	0	0	1	0	0	0
1	1	1	1	1	1	1
0	0	0	1	0	0	0
1	1	1	1	1	1	1
1						

For peer review only

1	1	1	1	1	1	1
0	0	0	0	0	0	0
345520	29849	400	2268	8500	2655	10000
1						
0						
345520						
			13.0%			
			13.0%			
100.0%	100.0%	100.0%		100.0%	100.0%	100.0%
			13.0%			
			13.0%			
100.0%	100.0%	100.0%		100.0%	100.0%	100.0%

For peer review only

100.0%

For peer review only

Pituitary Four	PNH Support	Polycystic Kid	Portsmouth D	Positive Actio	Positive East	Positive Help
23	1	4	1	1	7	1
21	1	0	0	0	2	1
1	1	1	1	1	1	1
1	1	0	0	0	1	1
1	1	1	1	1	1	1
1		1			1	
1						
	1					1
1		1	1	1	1	
2		4	1	1	5	
1		1			1	
2		4			5	
1						
2						

For peer review only

8.7%	0.0%					0.0%
8.7%	0.0%					0.0%
8.7%	0.0%					0.0%
		100.0%	100.0%	100.0%		
8.7%						
8.7%						
8.7%						
		100.0%				
8.7%						
8.7%						
8.7%						
110099	15150	68956	152	0	42960	3042
42209	15150	0	0	0	32097	15261
23	1	4	1	0	7	1
4	1	0	0	0	2	1
1	1	1	1	0	1	1
1	1	0	0	0	1	1
1	1	1	1		1	1
1						

For peer review only

	1			FALSE		
1		1	1		1	
						1
0	0	0	0		0	0
67891		68956	152		10863	
						12218
1						
0						
67891						
	0.0%					
	0.0%					
	0.0%				25.3%	
		100.0%	100.0%			
	0.0%					
	0.0%					
	0.0%				25.3%	
		100.0%	100.0%			

For peer review only

For peer review only

Positively UK	Prader-Willi S	Primary Immu	Progress Edu	Progressive S	Prostate Canc	Prostate Canc
31	1	8	2	3	1	2
20	0	8	10	0	0	4
1	1	1	1	1	1	1
1	0	1	1	0	0	1
1	1	1	1	1	1	1
1		1	1	1		1
1						
		1				
1	1			1	1	
			1			1
11	1			3	1	
			8			2
		1				
1				1		
			1			1
11				3		
			8			2
1						
11						

For peer review only

		0.0%				
		0.0%				
35.5%		0.0%				
	100.0%			100.0%	100.0%	
		0.0%				
		0.0%				
35.5%		0.0%				
				100.0%		
35.5%						
130874	473	73527	1373	564	1052	2088
146722	0	69182	0	0	0	0
31	1	8	2	3	1	2
20	0	8	0	0	0	0
1	1	1	1	1	1	1
1	0	1	0	0	0	0
1	1	1	1	1	1	1
1						

For peer review only

	1	1	1	1	1	1
1						
0	0	0	0	0	0	0
	473	4345	1373	564	1052	2088
15848						
1						
0						
15848						
		5.9%				
10.8%		5.9%				
10.8%		5.9%				
	100.0%		100.0%	100.0%	100.0%	100.0%
		5.9%				
10.8%		5.9%				
10.8%		5.9%				
	100.0%		100.0%	100.0%	100.0%	100.0%
10.8%						

For peer review only - http://bmjopen.bmj.com/site/about/guidelines.xhtml

10.8%

For peer review only

Prostate Canc	Prostate Cym	Psoriasis Asso	Pulmonary Hy	Pumping Mar	QUIT	Rainbow Cent
15	1	37	18	29	13	1
2	0	70	13	9	0	0
1	1	1	1	1	1	1
1	0	1	1	1	0	0
1	1	1	1	1	1	1
1		1	1	1	1	
1		1	1	1	1	
1	1		1	1	1	1
		1				
13	1		5	20	13	1
		33				
1			1	1	1	
		1				
13			5	20	13	
		33				
1			1	1	1	
		1				
13			5	20	13	
		33				

For peer review only

		47.1%	27.8%			
	100.0%				100.0%	100.0%
		47.1%	27.8%			
					100.0%	
		47.1%	27.8%			
					100.0%	
108777	640	427224	274170	307930	79340	579
0	0	0	278788	96358	0	0
14	1	37	18	29	13	1
0	0	0	13	5	0	0
1	1	1	1	1	1	1
0	0	0	1	1	0	0
1	1	1	1	1	1	1
1		1	1	1		

For peer review only

For peer review only

1	1	1		1	1	1
			1			
0	0	0	0	0	0	0
108777	640	427224		211573	79340	579
			4618			
1		1		1		
			1			
0		0	0	0		
108777		427224		211573		
			4618			
			1.7%			
			1.7%			
			1.7%			
100.0%	100.0%	100.0%			100.0%	100.0%
			1.7%			
			1.7%			
			1.7%			
100.0%	100.0%	100.0%			100.0%	100.0%
			1.7%			
			1.7%			

1.7%

100.0% 100.0%

For peer review only

Rainbow Trus	Rapid Effectiv	Rarer Cancers	Raynaud's an	Release	Restless Leg S	Rethink Ment
1	4	62	14	1	5	4
0	2	0	4	1	0	8
1	1	1	1	1	1	1
0	1	0	1	1	0	1
1	1	1	1	1	1	1
	1	1	1		1	1
		1	1			
				1		
1	1	1	1		1	
						1
1	2	62	10		5	
						4
	1	1	1		1	
						1
	2	62	10		5	
						4
		1	1			
		62	10			

For peer review only

For peer review only

				0.0%		
				0.0%		
				0.0%		
100.0%		100.0%			100.0%	
		100.0%			100.0%	
		100.0%				
1010	10915	682971	29651	10063	12940	61642
0	0	0	0	5143	0	12625
1	4	62	13	1	5	4
0	0	0	0	1	0	1
1	1	1	1	1	1	1
0	0	0	0	1	0	1
1	1	1	1	1	1	1
		1				

1	1	1	1	1	1	1
0	0	0	0	0	0	0
1010	10915	682971	29651	4921	12940	49017
		1				
		0				
		682971				
				48.9%		
100.0%	100.0%	100.0%	100.0%		100.0%	
				48.9%		
100.0%	100.0%	100.0%	100.0%		100.0%	

For peer review only

100.0%

For peer review only

Revive Multip	Richmond Fel	River House T	Ronald McDo	Roy Castle Lu	Royal Free Ch	Royal Marsde
3	1	1	1	70	2	1
1	0	0	0	0	3	0
1	1	1	1	1	1	1
1	0	0	0	0	1	0
1	1	1	1	1	1	1
1				1	1	
				1		
1	1	1	1	1		1
					1	
2	1	1	1	70		1
					1	
1				1		
					1	
2				70		
					1	
				1		
				70		

For peer review only

					33.3%	
	100.0%	100.0%	100.0%	100.0%		100.0%
					33.3%	
				100.0%		
				100.0%		
109067	736	253	771	770325	11827	355
29239	0	0	0	0	0	0
3	1	1	1	69	2	1
1	0	0	0	0	0	0
1	1	1	1	1	1	1
1	0	0	0	0	0	0
1	1	1	1	1	1	1
1				1		

For peer review only

1	1	1	1	1	1	1
0	0	0	0	0	0	0
79828	736	253	771	770325	11827	355
1				1		
0				0		
79828				770325		
	100.0%	100.0%	100.0%	100.0%	100.0%	100.0%
	100.0%	100.0%	100.0%	100.0%	100.0%	100.0%

For peer review only

100.0%

For peer review only

Royal Nationa	Salamander T	Samson Cent	SANE	Sarcoma UK	Save Babies T	Saving Lives
43	2	1	7	6	1	3
23	0	0	0	8	1	6
1	1	1	1	1	1	1
1	0	0	0	1	1	1
1	1	1	1	1	1	1
1	1		1	1		1
1						
					1	
1	1	1	1			
				1		1
20	2	1	7			
				2		3
1	1		1			
				1		1
20	2		7			
				2		3
1						
20						

For peer review only

					0.0%	
					0.0%	
46.5%				25.0%	0.0%	
	100.0%	100.0%	100.0%			
46.5%				25.0%		
	100.0%		100.0%			
46.5%						
1236891	721	4500	83687	29998	981	32786
982982	0	0	0	13677	0	70437
42	2	1	7	6	1	3
23	0	0	0	4	0	6
1	1	1	1	1	1	1
1	0	0	0	1	0	1
1	1	1	1	1	1	1
1						
1						

For peer review only

1	1	1	1	1	1	1
0	0	0	0	0	0	0
253909	721	4500	83687	16321	981	37650
1						
0						
253909						
1						
0						
253909						
20.5%						
	100.0%	100.0%	100.0%		100.0%	
20.5%						
	100.0%	100.0%	100.0%		100.0%	

For peer review only

20.5%

20.5%

For peer review only

Scleroderma	Scleroderma	Scoliosis Asso	Scotland Pati	Scottish Drug	Scottish Epile	Scottish Kidn
1	5	1	1	2	2	1
3	1	0	0	0	0	0
1	1	1	1	1	1	1
1	1	0	0	0	0	0
1	1	1	1	1	1	1
1	1			1	1	
	1	1	1	1	1	1
1						
	4	1	1	2	2	1
2						
	1			1	1	
1						
	4			2	2	
2						

For peer review only

		100.0%	100.0%	100.0%	100.0%	100.0%
				100.0%	100.0%	
2000	4513	278	1029	4056	78625	505
11110	634	0	0	0	0	0
1	5	1	1	2	2	1
3	1	0	0	0	0	0
1	1	1	1	1	1	1
1	1	0	0	0	0	0
1	1	1	1	1	1	1

For peer review only

	1	1	1	1	1	1
1						
0	0	0	0	0	0	0
	3879	278	1029	4056	78625	505
9110						
		100.0%	100.0%	100.0%	100.0%	100.0%
		100.0%	100.0%	100.0%	100.0%	100.0%

For peer review only

For peer review only

Scottish Netw	SeeAbility	Self Help Serv	Sense	Service by Em	Sexual Advice	Shift.MS
3	1	2	1	1	9	26
0	0	0	0	0	6	4
1	1	1	1	1	1	1
0	0	0	0	0	1	1
1	1	1	1	1	1	1
1		1			1	1
						1
1	1	1	1	1	1	1
3	1	2	1	1	3	22
1		1			1	1
3		2			3	22
						1
						22

For peer review only

					33.3%	
100.0%	100.0%	100.0%	100.0%	100.0%		
					33.3%	
100.0%		100.0%				
1139	473	514	152	210	115400	279784
0	0	0	0	0	0	0
3	1	2	1	1	8	26
0	0	0	0	0	0	0
1	1	1	1	1	1	1
0	0	0	0	0	0	0
1	1	1	1	1	1	1
					1	1

For peer review only

1	1	1	1	1	1	1
0	0	0	0	0	0	0
1139	473	514	152	210	115400	279784
					1	1
					0	0
					115400	279784
100.0%	100.0%	100.0%	100.0%	100.0%	100.0%	100.0%
100.0%	100.0%	100.0%	100.0%	100.0%	100.0%	100.0%

For peer review only

100.0%	100.0%

For peer review only

Short Bowel S	Sickle Cell an	Sickle Cell Soc	Sign Health	Silver Star	Skin Care Cym	Society for Muc
1	10	18	1	12	8	78
0	5	8	1	0	0	12
1	1	1	1	1	1	1
0	1	1	1	0	0	1
1	1	1	1	1	1	1
	1	1		1	1	1
		1		1		1
			1			
1	1	1		1	1	1
1	5	10		12	8	66
	1	1		1	1	1
	5	10		12	8	66
		1		1		1
		10		12		66

For peer review only

For peer review only

			0.0%			
			0.0%			
			0.0%			
100.0%				100.0%	100.0%	
				100.0%	100.0%	
				100.0%		
224	28443	50877	12625	117959	19925	1031298
0	37172	41925	0	0	0	0
1	10	17	1	12	8	78
0	4	8	0	0	0	0
1	1	1	1	1	1	1
0	1	1	0	0	0	0
1	1	1	1	1	1	1
				1		1
						1

1		1	1	1	1	1
	1					
0	0	0	0	0	0	0
224		8952	12625	117959	19925	1031298
	8728					
				1		1
				0		0
				117959		1031298
						1
						0
						1031298
		17.6%				
	23.5%	17.6%				
100.0%			100.0%	100.0%	100.0%	100.0%
		17.6%				
	23.5%	17.6%				
100.0%			100.0%	100.0%	100.0%	100.0%

For peer review only

100.0% 100.0%

100.0%

For peer review only

Somerville Fo	Sophia Forum	South Asian H	Spectra	Spinal Injuries	Spinal Injuries	Spinal Muscu
3	2	1	1	1	1	2
0	0	1	0	0	0	4
1	1	1	1	1	1	1
0	0	1	0	0	0	1
1	1	1	1	1	1	1
1	1					1
		1				
1	1		1	1	1	
						1
3	2		1	1	1	
						2
1	1					
						1
3	2					
						2

For peer review only

		0.0%				
		0.0%				
		0.0%				
100.0%	100.0%		100.0%	100.0%	100.0%	
100.0%	100.0%					
7328	10120	35998	1010	253	1052	46302
0	0	0	0	0	0	0
3	2	1	1	1	1	2
0	0	0	0	0	0	0
1	1	1	1	1	1	1
0	0	0	0	0	0	0
1	1	1	1	1	1	1

For peer review only

1	1	1	1	1	1	1
0	0	0	0	0	0	0
7328	10120	35998	1010	253	1052	46302
100.0%	100.0%	100.0%	100.0%	100.0%	100.0%	100.0%
100.0%	100.0%	100.0%	100.0%	100.0%	100.0%	100.0%

For peer review only

For peer review only

St Thomas Lu	Steps	Stick N Step	Stroke Associ	SUDEP Action	Surrey Young	Sussex Beaco
4	1	1	39	2	1	1
0	0	1	4	0	2	0
1	1	1	1	1	1	1
0	0	1	1	0	1	0
1	1	1	1	1	1	1
1			1	1	1	
			1			
		1				
1	1		1	1		1
					1	
4	1		35	2		1
					1	
1			1	1		
					1	
4			35	2		
					1	
			1			
			35			

For peer review only

		0.0%				
		0.0%				
		0.0%				
100.0%	100.0%			100.0%		100.0%
100.0%				100.0%		
62950	152	1349	256932	2334	5070	1000
0	0	0	20570	0	10100	0
4	1	1	38	2	1	1
0	0	0	1	0	1	0
1	1	1	1	1	1	1
0	0	0	1	0	1	0
1	1	1	1	1	1	1
			1			

For peer review only

1	1	1	1	1		1
					1	
0	0	0	0	0	0	0
62950	152	1349	236362	2334		1000
					5030	
			1			
			0			
			236362			
					49.8%	
100.0%	100.0%	100.0%		100.0%		100.0%
					49.8%	
100.0%	100.0%	100.0%		100.0%		100.0%

For peer review only

For peer review only

Tackle Africa	Tackle Prostat	Target Ovaria	TB Alert	Team Margot	Teenage Canc	Tenovus Canc
1	13	1	2	1	11	9
2	0	3	3	0	3	0
1	1	1	1	1	1	1
1	0	1	1	0	1	0
1	1	1	1	1	1	1
1	1	1	1		1	1
	1				1	
	1			1	1	1
1		1	1			
	13			1	8	9
1		2	1			
	1				1	1
1		1	1			
	13				8	9
1		2	1			
	1				1	
	13				8	

For peer review only

For peer review only

			33.3%			
	100.0%			100.0%		100.0%
			33.3%			
	100.0%					100.0%
	100.0%					
3042	148470	0	27401	5050	11904	170503
6072	0	0	0	0	0	0
1	13	0	2	1	10	9
2	0	0	0	0	0	0
1	1	0	1	1	1	1
1	0	0	0	0	0	0
1	1		1	1	1	1
	1					1

		FALSE				
	1		1	1	1	1
1						
0	0		0	0	0	0
	148470		27401	5050	11904	170503
3030						
	1					1
	0					0
	148470					170503
49.9%						
	100.0%		100.0%	100.0%	100.0%	100.0%
49.9%						
	100.0%		100.0%	100.0%	100.0%	100.0%

For peer review only

100.0% 100.0%

For peer review only

Terrence Higg	Thames Valle	Thrombosis U	Tiny Tickers	Tommy's	Transplant Lir	Transverse M
13	2	17	2	1	2	1
12	0	0	2	5	0	0
1	1	1	1	1	1	1
1	0	0	1	1	0	0
1	1	1	1	1	1	1
1	1	1	1	1	1	
1		1				
			1			
1	1	1			1	1
				1		
1	2	17			2	1
				4		
			1			
1	1	1			1	
				1		
1	2	17			2	
				4		
1		1				
1		17				

For peer review only

7.7%			0.0%			
7.7%			0.0%			
7.7%			0.0%			
	100.0%	100.0%			100.0%	100.0%
7.7%			0.0%			
7.7%			0.0%			
7.7%			0.0%			
	100.0%	100.0%			100.0%	
7.7%						
7.7%						
7.7%						
		100.0%				
108993	5736	45782	12221	10285	1532	15150
67744	0	0	0	0	0	0
13	2	17	2	1	2	1
8	0	0	0	0	0	0
1	1	1	1	1	1	1
1	0	0	0	0	0	0
1	1	1	1	1	1	1
1						

For peer review only

1	1	1	1	1	1	1
0	0	0	0	0	0	0
41249	5736	45782	12221	10285	1532	15150
1						
0						
41249						
37.8%						
	100.0%	100.0%	100.0%	100.0%	100.0%	100.0%
37.8%						
	100.0%	100.0%	100.0%	100.0%	100.0%	100.0%

For peer review only

37.8%

For peer review only

Tree of Hope	Trigeminal Ne	Tuberous Scle	Turner Syndro	Twins And Mu	UK Chronic Ly	UK Gout Socie
1	3	8	21	6	1	3
0	0	4	11	5	0	0
1	1	1	1	1	1	1
0	0	1	1	1	0	0
1	1	1	1	1	1	1
	1	1	1	1		1
			1			
1	1	1	1	1	1	1
1	3	4	10	1	1	3
	1	1	1	1		1
	3	4	10	1		3
			1			
			10			

For peer review only

				16.7%		
			47.6%	16.7%		
100.0%	100.0%				100.0%	100.0%
				16.7%		
			47.6%	16.7%		
	100.0%					100.0%
			47.6%			
152	4208	91245	39241	42922	4056	38407
0	0	120378	0	0	0	0
1	1	8	20	6	1	3
0	0	4	0	0	0	0
1	1	1	1	1	1	1
0	0	1	0	0	0	0
1	1	1	1	1	1	1
		1				

For peer review only

1	1	1	1	1	1	1
0	0	0	0	0	0	0
152	4208	29133	39241	42922	4056	38407
		1				
		0				
		29133				
100.0%	100.0%	24.2%	100.0%	100.0%	100.0%	100.0%
100.0%	100.0%	24.2%	100.0%	100.0%	100.0%	100.0%

For peer review only

24.2%

For peer review only

UK Primary In	UK Sepsis Tru	UK Thalassae	Urology Foun	Visionary	Waldenstrom	Walk the Wal
15	1	7	3	6	6	1
1	0	12	5	2	3	0
1	1	1	1	1	1	1
1	0	1	1	1	1	0
1	1	1	1	1	1	1
1		1	1	1	1	
1		1				
1	1			1	1	1
		1	1			
14	1			4	3	1
		5	2			
1				1	1	
		1	1			
14				4	3	
		5	2			
1						
		1				
14						
		5				

For peer review only

		41.7%	40.0%			
	100.0%					100.0%
		41.7%	40.0%			
		41.7%				
107005	0	43405	30621	95543	25021	406
0	0	132930	0	15273	0	0
15	0	7	3	6	6	1
0	0	12	0	2	0	0
1	0	1	1	1	1	1
0	0	1	0	1	0	0
1		1	1	1	1	1
1		1				

For peer review only

	FALSE					
1			1	1	1	1
		1				
0		0	0	0	0	0
107005			30621	80270	25021	406
		89525				
1						
		1				
0		0				
107005						
		89525				
100.0%			100.0%		100.0%	100.0%
100.0%			100.0%		100.0%	100.0%

For peer review only

100.0%

For peer review only

Waverley Car	Wellbeing of	WellChild	Willow Found	World Cancer	World Child C	York Air Amb
18	1	2	1	1	15	1
0	1	1	0	0	0	0
1	1	1	1	1	1	1
0	1	1	0	0	0	0
1	1	1	1	1	1	1
1		1			1	
1					1	
	1					
1		1	1	1	1	1
18		1	1	1	15	1
1		1			1	
18		1			15	
1					1	
18					15	

For peer review only

	0.0%					
	0.0%					
	0.0%					
100.0%			100.0%	100.0%	100.0%	100.0%
100.0%					100.0%	
100.0%					100.0%	
103557	4114	262000	258	5000	342276	0
0	0	0	0	0	0	0
18	1	2	1	1	15	0
0	0	0	0	0	0	0
1	1	1	1	1	1	0
0	0	0	0	0	0	0
1	1	1	1	1	1	
1		1			1	

For peer review only

						FALSE
1	1	1	1	1	1	
0	0	0	0	0	0	
103557	4114	262000	258	5000	342276	
1		1			1	
0		0			0	
103557		262000			342276	
100.0%	100.0%	100.0%	100.0%	100.0%	100.0%	
100.0%	100.0%	100.0%	100.0%	100.0%	100.0%	

For peer review only

100.0%	100.0%	100.0%

For peer review only

Young Epilepsy

14
7
1
1

1
1
1

1

7

1

7

1

7

147292
0
13
0
1
0

1
1

For peer review only

1

0
147292

1

0
147292

100.0%

100.0%

For peer review only

100.0%

For peer review only

For peer review only

For peer review only

For peer review only

Web Supplement 6. Recipients - Absolute and relative differences in the number and value of paym

NUMBER OF PAYMENTS - ABSOLUTE DIFFERENCES

Threshold 0: At least 1 one payment in at least one dataset
Total
Number of patient organisations with exact match in both datasets
Number of patient organisations with more paymens in industry data
Number of patient organisations with more payments in patient organisation data

Highest absolute difference between patient organisation and industry data - number of payments hi
Highest absolute difference between patient organisation and industry data - number of payments hi

Threshold 1: More than 1 one payment in at least one datase
Total
Number of patient organisations with exact match in both datasets
Number of patient organisations with more paymens in industry data
Number of patient organisations with more payments in patient organisation data

Highest absolute difference between patient organisation and industry data - number of payments hi
Highest absolute difference between patient organisation and industry data - number of payments hi

Threshold 2: More than 10 payments in at least one dataset
Total
Number of patient organisations with exact match in both datasets
Number of patient organisations with more paymens in industry data
Number of patient organisations with more payments in patient organisation data

Highest absolute difference between patient organisation and industry data - number of payments hi
Highest absolute difference between patient organisation and industry data - number of payments hi

Threshold 3: More than 100 payments in at least one dataset
Total
Number of patient organisations with exact match in both datasets
Number of patient organisations with more paymens in industry data
Number of patient organisations with more payments in patient organisation data

Highest absolute difference between patient organisation and industry data - number of payments hi
Highest absolute difference between patient organisation and industry data - number of payments hi

NUMBER OF PAYMENTS - RELATIVE DIFFERENCES

Threshold 0: At least 1 one payment in at least one dataset

For peer review only

Total

Relative difference <10%

Relative difference <20%

Relative difference <50%

Relative difference = 100%

Threshold 1: More than 1 one payment in at least one datase

Total

Relative difference <10%

Relative difference <20%

Relative difference <50%

Relative difference = 100%

Threshold 2: More than 10 payments in at least one dataset

Total

Relative difference <10%

Relative difference <20%

Relative difference <50%

Relative difference = 100%

Threshold 3: More than 100 payments in at least one dataset

Total

Relative difference <10%

Relative difference <20%

Relative difference <50%

Relative difference = 100%

<u>VALUE OF PAYMENTS - ABSOLUTE DIFFERENCES</u>

Threshold 1: At least one payment with value >£0 in at least one dataset

Total

Number of patient organisations with exact match in both datasets

Number of patient organisations with higher payment value in industry data

Number of patient organisations with higher payment value in patient organisation data

Highest absolute difference between patient organisation and industry data - number of payments hi[illegible]

Highest absolute difference between patient organisation and industry data - number of payments hi[illegible]

Threshold 2: Payments with the value of more than £10,000 in at least one dataset

Total

Number of patient organisations with exact match in both datasets

Number of patient organisations with higher payment value in industry data

Number of patient organisations with higher payment value in patient organisation data

For peer review only

Highest absolute difference between patient organisation and industry data - number of payments hi
Highest absolute difference between patient organisation and industry data - number of payments hi

Threshold 3: Payments with the value of more than £100,000 in at least one dataset
Total
Number of patient organisations with exact match in both datasets
Number of patient organisations with higher payment value in industry data
Number of patient organisations with higher payment value in patient organisation data

Highest absolute difference between patient organisation and industry data - number of payments hi
Highest absolute difference between patient organisation and industry data - number of payments hi

VALUE OF PAYMENTS - RELATIVE DIFFERENCES

Threshold 1: At least one payment with value >£0 in at least one dataset
Total
Relative difference <10%
Relative difference <20%
Relative difference <50%
Relative difference = 100%

Threshold 2: Payments with the value of more than £10,000 in at least one dataset
Total
Relative difference <10%
Relative difference <20%
Relative difference <50%
Relative difference = 100%

Threshold 3: Payments with the value of more than £100,000 in at least one dataset
Total
Relative difference <10%
Relative difference <20%
Relative difference <50%
Relative difference = 100%

For peer review only

All years		2012		2013	
Number of pat	% of all patient c	Number of pat	% of all patient c	Number of pat	% of all patient c
425	100.0%	219	100.0%	225	100.0%
33	7.8%	17	7.8%	18	8.0%
335	78.8%	160	73.1%	157	69.8%
57	13.4%	42	19.2%	50	22.2%
Number of payments		Number of payments		Number of payments	
199		36		35	
33		9		17	
Number of pat	% of all patient c	Number of pat	% of all patient c	Number of pat	% of all patient c
299	100.0%	140	100.0%	137	100.0%
13	4.3%	6	4.3%	9	6.6%
229	76.6%	105	75.0%	95	69.3%
57	19.1%	29	20.7%	33	24.1%
Number of payments		Number of payments		Number of payments	
199		36		35	
33		9		17	
Number of pat	% of all patient c	Number of pat	% of all patient c	Number of pat	% of all patient c
115	100.0%	20	100.0%	13	100.0%
2	1.7%	1	5.0%	0	0.0%
95	82.6%	16	80.0%	9	69.2%
18	15.7%	3	15.0%	4	30.8%
Number of payments		Number of payments		Number of payments	
199		36		35	
33		9		17	
Number of pat	% of all patient c	Number of pat	% of all patient c	Number of pat	% of all patient c
3	100.0%	0	N/A	0	N/A
0	0.0%	N/A	N/A	N/A	N/A
3	100.0%	N/A	N/A	N/A	N/A
0	0.0%	N/A	N/A	N/A	N/A
Number of payments		Number of payments		Number of payments	
199		0		0	
0		0		0	
Number of pat	% of all patient c	Number of pat	% of all patient c	Number of pat	% of all patient c

For peer review only

425	100.0%	219	100.0%	225	100.0%
35	8.2%	17	7.8%	18	8.0%
42	9.9%	18	8.2%	19	8.4%
81	19.1%	30	13.7%	34	15.1%
225	52.9%	138	63.0%	150	66.7%

Number of pat	% of all patient c	Number of pa	% of all patient c	Number of pa	% of all patient c
299	100.0%	140	100.0%	137	100.0%
15	5.0%	6	4.3%	9	6.6%
22	7.4%	7	5.0%	10	7.3%
61	20.4%	19	13.6%	25	18.2%
119	39.8%	70	50.0%	71	51.8%

Number of pat	% of all patient c	Number of pa	% of all patient c	Number of pa	% of all patient c
115	100.0%	20	100.0%	13	100.0%
4	3.5%	1	5.0%	0	0.0%
7	6.1%	2	10.0%	0	0.0%
31	27.0%	4	20.0%	1	7.7%
33	28.7%	7	35.0%	5	38.5%

Number of pat	% of all patient c	Number of pa	% of all patient c	Number of pa	% of all patient c
3	100.0%	0	N/A	0	N/A
0	0.0%	N/A	N/A	N/A	N/A
0	0.0%	N/A	N/A	N/A	N/A
0	0.0%	N/A	N/A	N/A	N/A
1	33.3%	N/A	N/A	N/A	N/A

Number of pat	% of all patient c	Number of pa	% of all patient c	Number of pa	% of all patient c
416	100.0%	206	100.0%	204	100.0%
8	1.9%	4	1.9%	4	2.0%
356	85.6%	168	81.6%	164	80.4%
52	12.5%	34	16.5%	36	17.6%

Value of payments	Value of payments	Value of payments
6,718,576.6	660,846.1	369,657.9
6,493,237.1	283,684.7	829,991.7

Number of pat	% of all patient c	Number of pa	% of all patient c	Number of pa	% of all patient c
104	100.0%	29	100.0%	20	100.0%
0	0.0%	0	0.0%	0	0.0%
80	76.9%	19	65.5%	12	60.0%
24	23.1%	10	34.5%	8	40.0%

Value of payments	Value of payments	Value of payments

For peer review only

For peer review only - http://bmjopen.bmj.com/site/about/guidelines.xhtml

6,718,576.6		660,846.1		369,657.9	
6,493,237.1		283,684.7		829,991.7	

Number of pat	% of all patient c	Number of pa	% of all patient c	Number of pa	% of all patient c
13	100.0%	0	N/A	0	N/A
0	0.0%	N/A	N/A	N/A	N/A
6	46.2%	N/A	N/A	N/A	N/A
7	53.8%	N/A	N/A	N/A	N/A

Value of payments	Value of payments	Value of payments
6,718,576.6	-	-
6,493,237.1	-	-

Number of pat	% of all patient c	Number of pa	% of all patient c	Number of pa	% of all patient c
416	100.0%	206	100.0%	204	100.0%
21	5.0%	6	2.9%	8	3.9%
34	8.2%	11	5.3%	13	6.4%
73	17.5%	20	9.7%	21	10.3%
295	70.9%	158	76.7%	164	80.4%

Number of pat	% of all patient c	Number of pa	% of all patient c	Number of pa	% of all patient c
104	100.0%	29	100.0%	20	100.0%
4	3.8%	0	0.0%	0	0.0%
8	7.7%	2	6.9%	0	0.0%
23	22.1%	4	13.8%	2	10.0%
54	51.9%	17	58.6%	11	55.0%

Number of pat	% of all patient c	Number of pa	% of all patient c	Number of pa	% of all patient c
13	100.0%	0	N/A	0	N/A
0	0.0%	N/A	N/A	N/A	N/A
0	0.0%	N/A	N/A	N/A	N/A
3	23.1%	N/A	N/A	N/A	N/A
4	30.8%	N/A	N/A	N/A	N/A

For peer review only

2014		2015		2016
Number of patie	% of all patient c	Number of patie	% of all patient organisation	Number of patie
247	100.0%	267	100.0%	260
29	11.7%	31	11.6%	21
173	70.0%	186	69.7%	208
45	18.2%	50	18.7%	31
Number of payments		Number of payments		Number of paym
36		44		55
15		10		11
Number of patie	% of all patient c	Number of patie	% of all patient organisation	Number of patie
146	100.0%	149	100.0%	166
6	4.1%	8	5.4%	7
110	75.3%	107	71.8%	138
30	20.5%	34	22.8%	21
Number of payments		Number of payments		Number of paym
36		44		55
15		10		11
Number of patie	% of all patient c	Number of patie	% of all patient organisation	Number of patie
15	100.0%	22	100.0%	29
0	0.0%	0	0.0%	0
11	73.3%	19	86.4%	26
4	26.7%	3	13.6%	3
Number of payments		Number of payments		Number of paym
36		44		55
15		10		11
Number of patie	% of all patient c	Number of patie	% of all patient organisation	Number of patie
0	N/A	0	N/A	0
N/A	N/A	N/A	N/A	N/A
N/A	N/A	N/A	N/A	N/A
N/A	N/A	N/A	N/A	N/A
Number of payments		Number of payments		Number of paym
0		0		0
0		0		0
Number of patie	% of all patient c	Number of patie	% of all patient organisation	Number of patie

For peer review only

247	100.0%	267	100.0%	260
29	11.7%	31	11.6%	21
32	13.0%	33	12.4%	24
48	19.4%	49	18.4%	45
153	61.9%	164	61.4%	159

Number of patie	% of all patient c	Number of patie	% of all patient organisation	Number of patie
146	100.0%	149	100.0%	166
6	4.1%	8	5.4%	7
9	6.2%	10	6.7%	10
25	17.1%	26	17.4%	31
75	51.4%	69	46.3%	79

Number of patie	% of all patient c	Number of patie	% of all patient organisation	Number of patie
15	100.0%	22	100.0%	29
0	0.0%	0	0.0%	0
1	6.7%	1	4.5%	1
3	20.0%	4	18.2%	6
5	33.3%	7	31.8%	13

Number of patie	% of all patient c	Number of patie	% of all patient organisation	Number of patie
0	N/A	0	N/A	0
N/A	N/A	N/A	N/A	N/A
N/A	N/A	N/A	N/A	N/A
N/A	N/A	N/A	N/A	N/A
N/A	N/A	N/A	N/A	N/A

Number of patie	% of all patient c	Number of patie	% of all patient organisation	Number of patie
234	100.0%	249	100.0%	249
11	4.7%	15	6.0%	8
196	83.8%	198	79.5%	211
27	11.5%	36	14.5%	30

Value of payments	Value of payments	Value of paymer
538,969.2	1,473,965.2	6,006,677.7
1,824,997.0	815,956.8	6,493,237.1

Number of patie	% of all patient c	Number of patie	% of all patient organisation	Number of patie
31	100.0%	34	100.0%	36
0	0.0%	0	0.0%	0
24	77.4%	25	73.5%	25
7	22.6%	9	26.5%	11

Value of payments	Value of payments	Value of paymer

For peer review only

538,969.2		1,473,965.2		6,006,677.7
1,824,997.0		815,956.8		6,493,237.1

Number of patie	% of all patient c	Number of patie	% of all patient organisation	Number of patie
1	100.0%	1	100.0%	2
0	0.0%	0	0.0%	0
0	0.0%	1	100.0%	1
1	100.0%	0	0.0%	1

Value of payments		Value of payments		Value of paymer
-		1,473,965.24		6,006,677.66
1,824,996.99		-		6,493,237.05

Number of patie	% of all patient c	Number of patie	% of all patient organisation	Number of patie
234	100.0%	249	100.0%	249
16	6.8%	20	8.0%	18
19	8.1%	25	10.0%	26
28	12.0%	39	15.7%	36
185	79.1%	189	75.9%	197

Number of patie	% of all patient c	Number of patie	% of all patient organisation	Number of patie
31	100.0%	34	100.0%	36
1	3.2%	0	0.0%	2
2	6.5%	0	0.0%	4
5	16.1%	5	14.7%	8
20	64.5%	21	61.8%	20

Number of patie	% of all patient c	Number of patie	% of all patient organisation	Number of patie
1	100.0%	1	100.0%	2
0	0.0%	0	0.0%	0
0	0.0%	0	0.0%	0
0	0.0%	0	0.0%	0
0	0.0%	1	100.0%	0

For peer review only

% of all patient organisationsa given threshold
100.0%
8.1%
80.0%
11.9%

nents

% of all patient organisationsa given threshold
100.0%
4.2%
83.1%
12.7%

nents

% of all patient organisationsa given threshold
100.0%
0.0%
89.7%
10.3%

nents

% of all patient organisationsa given threshold
N/A
N/A
N/A
N/A

nents

% of all patient organisationsa given threshold

For peer review only

100.0%
8.1%
9.2%
17.3%
61.2%

% of all patient organisationsa given threshold
100.0%
4.2%
6.0%
18.7%
47.6%

% of all patient organisationsa given threshold
100.0%
0.0%
3.4%
20.7%
44.8%

% of all patient organisationsa given threshold
N/A
N/A
N/A
N/A
N/A

% of all patient organisationsa given threshold
100.0%
3.2%
84.7%
12.0%

nts

% of all patient organisationsa given threshold
100.0%
0.0%
69.4%
30.6%

nts

For peer review only

% of all patient organisationsa given threshold
100.0%
0.0%
50.0%
50.0%

nts

% of all patient organisationsa given threshold
100.0%
7.2%
10.4%
14.5%
79.1%

% of all patient organisationsa given threshold
100.0%
5.6%
11.1%
22.2%
55.6%

% of all patient organisationsa given threshold
100.0%
0.0%
0.0%
0.0%
0.0%

For peer review only

Web Supplement 7. Donors - summary of payments missing from i

	Year
	**2012**
	**2013**
	**2014**
	**2015**
	**2016**
**Total missing - calculated annually**	
**Total missing - calculated for all years**	

For peer review only

ndustry data

Number of companies missing from industry data

31
30
27
23
0
**N/A**
**24**

For peer review only

Companies missing from industry dataset as a share of companies in industry d

103.3%
78.9%
60.0%
45.1%
0.0%
**N/A**
**38.1%**

For peer review only

Number of payments missing from industry data	Number of payments missing from industry datas
124	16.1%
131	18.6%
74	9.0%
52	5.6%
54	5.0%
**435**	**10.1%**
**128**	**3.0%**

For peer review only

Value of payments missing from industry data (Value of payments missing from industr

1,731,257.8	22.2%
2,666,247.8	43.1%
218,768.4	2.5%
1,367,440.0	12.0%
474,975.0	2.4%
**6,458,689.1**	**11.9%**
**1,610,321.1**	**3.0%**

For peer review only

ry datas as a share of payments in industry datas

For peer review only

Web Supplement 8. Donors - payments missing from industry data (yea

Year	Drug company name	Number of payments
	Alcon	3
	Alexion	1
	ApoPharma	1
	Baxter	7
	Bayer	8
	BioMarin	1
	Biotest	1
	Boehringer Ingelheim	2
	Dermal	3
	Eisai	1
	Flynn Pharma	1
	Fresenius Medical Care	1
	Genzyme	9
	Gilead	8
	GSK	18
2012	Ipsen	3
	Janssen	12
	LEO Pharma	2
	Norgine	1
	Novo Nordisk	3
	Octapharma	1
	Pharma Mar	1
	RB	1
	Roche Products	13
	Sanofi Pasteur MSD	1
	Sigma Tau Rare Diseases	1
	Sobi	1
	Takeda	6
	Teva	5
	UCB	7
	Veriton	1
	Alcon	4
	Alexion	2
	Almirall	2
	Amdipharm	1
	ApoPharma	3
	Bausch and Lomb	2
	Baxter	3
	Bayer	9
	Britannia	1
	Dermal	3
	Gedeon Richter	1
	Genzyme	9
	Gilead	10

For peer review only

	GSK	16
	Ipsen	2
2013	IQVIA	1
	Janssen	13
	LEO Pharma	4
	Lundbeck	1
	Meda	1
	Merck	8
	Norgine	2
	Orphan Europe	1
	PTC	2
	RB	2
	Roche Products	14
	Sigma Tau Rare Diseases	2
	Takeda	9
	Veriton	1
	Vifor Pharma Group	2
	Actavis	1
	Alcon	1
	Alexion	5
	Alliance	1
	Amdipharm	1
	ApoPharma	1
	Baxter	2
	Bayer	4
	Britannia	1
	Dermal	2
	Gedeon Richter	1
	Genzyme	13
	GSK	12
2014	Ipsen	2
	Meda	1
	Mitsubishi Tanabe Pharma	1
	Norgine	2
	Novo Nordisk	4
	Orion Pharma	1
	PTC	2
	Rosemont Pharmaceuticals	1
	Sigma Tau Rare Diseases	2
	Stirling Anglian	1
	Syner-Med	2
	Teva	6
	Veriton	2
	Vifor Pharma Group	2
	Actavis	2
	Aegerion	1

For peer review only

	ALK-Abello	1
	Amdipharm	1
	Bausch and Lomb	1
	Britannia	1
	Dermal	3
	Fresenius Medical Care	1
	Genzyme	9
	GSK	13
	HRA Pharma	1
2015	Norgine	3
	Octapharma	2
	Orphan Europe	1
	Pharma Mar	1
	PTC	1
	RB	1
	Sanofi Pasteur MSD	1
	Santen	1
	Sigma Tau Rare Diseases	2
	Syner-Med	1
	Thea	1
	Vifor Pharma Group	3
	Meda	1
	ALK-Abello	1
	Bausch and Lomb	1
	Genzyme	12
	HRA Pharma	1
	BioMarin	4
	Allergan	5
	Napp Pharmaceuticals	2
	Amdipharm	1
	Alexion	4
	Orphan Europe	1
	Ferring	6
2016	Britannia	1
	Aegerion	1
	Baxalta	1
	Diurnal	1
	PTC	1
	Bio Products Laboratory	1
	Octapharma	2
	Sigma Tau Rare Diseases	1
	Baxter	1
	Fresenius Medical Care	1
	Jazz	1
	Mylan	1
	Dermal	2

For peer review only

rly breakdown)

Value of payments (2016 £)
18937.5
0.0
23145.8
170239.7
205156.3
0.0
22480.9
9468.8
0.0
0.0
2104.2
7890.6
116178.4
50500.0
501114.7
25250.0
50804.1
0.0
0.0
0.0
134882.3
6312.5
83114.6
237981.3
20989.1
0.0
1052.1
23145.8
15248.9
5260.4
0.0
64796.3
5142.6
0.0
0.0
22627.3
22627.3
135138.4
299046.4
0.0
0.0
0.0
278174.8
116016.3

For peer review only

406794.5
20570.3
0.0
42110.4
0.0
0.0
0.0
238799.2
0.0
1028.5
0.0
30855.4
126301.4
514.3
4695.2
0.0
851009.3
0.0
0.0
15210.8
0.0
18253.0
30421.7
70948.4
27301.4
0.0
0.0
0.0
225781.6
2123196.4
34873.4
0.0
5070.3
16884.0
16144.8
0.0
0.0
0.0
28869.2
0.0
0.0
7098.4
0.0
470654.9
24240.0
0.0

For peer review only

0.0
15680.3
0.0
0.0
0.0
5050.0
257424.8
211609.1
0.0
16816.5
64864.2
0.0
0.0
0.0
30300.0
0.0
0.0
11586.7
0.0
0.0
729868.4
0.0
0.0
0.0
348335.0
0.0
5000.0
0.0
23000.0
500.0
27000.0
0.0
0.0
3884.0
0.0
0.0
0.0
0.0
10000.0
24766.0
12706.0
16784.0
3000.0
0.0
0.0
0.0

For peer review only

Web Supplement 9. Donors - payments missing from industry data (breakdown for all years)

Year	Drug company name	Number of payments	Value of payments (2016 £)
2012-2016	RB	4	144270.0
	Genzyme	52	1225894.6
	Sigma Tau Rare Diseases	8	53676.1
	Veriton	4	0.0
	Sanofi Pasteur MSD	2	20989.1
	Fresenius Medical Care	3	15940.6
	Dermal	13	0.0
	Pharma Mar	2	6312.5
	ApoPharma	5	76194.8
	Amdipharm	4	34433.3
	Orphan Europe	3	1028.5
	Gedeon Richter	2	0.0
	PTC	6	0.0
	IQVIA	1	0.0
	Britannia	4	3884.0
	Bausch and Lomb	4	22627.3
	Syner-Med	3	0.0
	Mitsubishi Tanabe Pharma	1	5070.3
	Alliance	1	0.0
	ALK-Abello	2	0.0
	Thea	1	0.0
	Diurnal	1	0.0
	Jazz	1	0.0
	Mylan	1	0.0

For peer review only

Web Supplement 10. Donors - summary of payments missing from patient

Year	Number of companies missing from patient organi
**2012**	5
**2013**	3
**2014**	7
**2015**	5
**2016**	4
**Total missing - calculate**	**N/A**
**Total missing - calculate**	**3**

For peer review only

organisation data

Companies missing from patient organisation data as a share of companies in p

8.9%
4.6%
10.8%
7.1%
6.2%
**N/A**
**3.6%**

For peer review only

Number of payments missing from patient organi	Number of payments missing from patient organi
14	4.8%
6	1.8%
17	4.9%
20	5.5%
10	3.0%
**67**	**4.0%**
**9**	**0.5%**

For peer review only

Value of payments missing from patient organi:	Value of payments missing from patien
113,328.0	3.3%
68,397.8	1.5%
138,893.5	2.6%
52,758.7	1.1%
20,649.1	0.1%
**394,027.2**	**1.2%**
**92,208.2**	**0.3%**

For peer review only

t organisation data as a share of payments in patient organisation data

For peer review only

Web Supplement 11. Donors - payments missing from patient organisation data (yearly breakdo

Year	Drug company name	Number of payments	Value of payments (2016 £)
2012	A. Menarini	2	26302.1
	Consilient Health	2	21041.7
	Daiichi Sankyo	2	16833.3
	Meda	4	30589.3
	Servier Laboratories	4	18561.6
2013	Consilient Health	1	10285.1
	HRA Pharma	3	46797.4
	Servier Laboratories	2	11315.4
2014	A. Menarini	1	7605.4
	Almirall	3	47559.2
	Chugai	2	811.2
	Consilient Health	1	10140.6
	Grünenthal	3	12939.4
	Lundbeck	5	50702.8
	Servier Laboratories	2	9134.9
2015	Almirall	3	12538.1
	Chugai	2	15409.9
	Flynn Pharma	5	13700.7
	Rosemont Pharmaceuticals	7	0.0
	Stirling Anglian	3	11110.0
2016	Almirall	3	2871.6
	Chugai	3	2612.5
	Flynn Pharma	3	5165.0
	Orion Pharma	1	10000.0

For peer review only

own)

For peer review only

Web Supplement 12. Donors - payments missing from patient organisation data (all years

Year	Drug company name	Number of payments	Value of payments (2016 £)
	A. Menarini	3	33907.5
2012-2016	Consilient Health	4	41467.4
	Daiichi Sankyo	2	16833.3

For peer review only

s)

For peer review only

Web Supplement 13. Recipients - summary of payments missing from patient organisation

Year	Number of patient organisations missing from pat
**2012**	116
**2013**	123
**2014**	131
**2015**	141
**2016**	143
**Total missing - calculated annually**	**N/A**
**Total missing - calculated for all years**	**225**

For peer review only

data

Patient organisations missing from patient organisation data as a share of all pa

112.6%
120.6%
112.9%
111.9%
122.2%
**N/A**
**112.5%**

For peer review only

Number of payments missing from patient organ	Number of payments missing from patient organi
399	137.1%
369	113.2%
411	118.8%
462	128.0%
533	158.2%
**2174**	**130.9%**
**1472**	**88.6%**

For peer review only

Value of payments missing from patient organi	Value of payments missing from patien
3,659,293.41	105.8%
2,340,576.29	53.0%
4,427,502.34	83.5%
6,591,354.62	136.6%
4,871,241.58	32.4%
**21,889,968.23**	**66.3%**
**14,023,475.41**	**42.4%**

For peer review only

t organisation data as share of the value of payments in patient organisation data

For peer review only

Web Supplem

Year

For peer review only

2012

For peer review only

For peer review only

2013

For peer review only

For peer review only

For peer review only

2014

For peer review only

For peer review only

For peer review only

2015

For peer review only

For peer review only

For peer review only

2016

For peer review only

For peer review only

For peer review only

nent 14. Recipients - payments missing from patient organisation data (yearly breakdown)

Patient organisation name

Action on Pain
Action on Pre-Eclampsia
African-Caribbean Leukaemia Trust
Age Related Diseases and Health Trust
Allergy Research Foundation
Alzheimer Scotland
Alzheimers Dementia Support
Anaphylaxis Campaign
Anticoagulation UK
Arrhythmia Alliance
Arthritis and Musculoskeletal Alliance
Atrial Fibrillation Association
Beating Bowel Cancer
Bipolar Scotland
Bipolar UK
Bladder and Bowel Foundation (taken over by Bladder and Bowel Community in 2017)
Bladder Health UK
Blood Pressure UK
Bluebell Foundation
Brain Charity
Breakthrough Breast Cancer (current name Breast Cancer Now, following merger with Breast Cancer
Breast Cancer Campaign (current name Breast Cancer Now, following merger with Breakthrough Brea
British Dupuytren's Society
British Heart Foundation
British Obesity Society
Brook
Cancer 52
Cancer Focus Northern Ireland
Cancer Research UK
Child Growth Foundation
Children with Cancer UK
Children’s Heart Unit Fund
Children’s HIV Association
Chinese National Healthy Living Centre
Cleft Lip and Palate Association
Coping with Cancer in Leicestershire and Rutland
Core Charity
Crohn's in Childhood Research Association
Cure Parkinson's Trust
Cystic Fibrosis Trust
Deaf Education through Listening and Talking
Diabetes Research and Wellness Foundation
Diabetes UK
Disabled Living

For peer review only

Epilepsy Scotland
European Parkinson’s Disease Association
Family Planning Association
Forum Link
Gay Men's Health
GIST Support UK
Group B Strep Support UK
Haemophilia Society
Health and Social Care Alliance Scotland
Heartbeat
Hereditary Angioedema UK
HIV i-Base
Hughes Syndrome Foundation
ICUsteps
Independent Cancer Patients' Voice
International Alliance of Patients' Organizations
Karen Clifford Skcin cancer charity
Kidney Cancer UK
Leicestershire AIDS Support Services
Macmillan Cancer Support
Maggie's Centre
Malnutrition and Nutritional Care in the UK
Marie Curie Cancer Care
Medical Foundation for HIV and Sexual Health
Melanoma UK
Mental Health Foundation
Migraine Action
MPN Voice
Myeloma UK
Myrovlytis Trust
National Eczema Society
National Kidney Federation
National Obesity Forum
National Osteoporosis Society
OcuMel UK
Organisation for Sickle Cell Anaemia Research and Thalassaemia Support
Paget's Association
Pain Association Scotland
Pain Concern
Pain Relief Foundation
Pain UK
Pancreatic Cancer UK
Patients Association
Pelvic Pain Support Network
Positive East
Prader-Willi Syndrome Association UK

For peer review only

Prostate Cancer Research Centre
QUIT
Rarer Cancers Foundation
Raynaud's and Scleroderma Association (current name: Scleroderma and Raynauds UK, following the
Richmond Fellowship
Roy Castle Lung Cancer Foundation
Salamander Trust
SANE
SeeAbility
Service by Emergency Response Volunteers - Suffolk and Cambridgeshire
Shift.MS
Silver Star
Society for Mucopolysaccharide Diseases
Somerville Foundation
Spinal Injuries Scotland
Spinal Muscular Atrophy Support UK
Stroke Association
Tackle Prostate Cancer
Tenovus Cancer Care
Thames Valley Positive Support
Thrombosis UK
Trigeminal Neuralgia Association
UK Gout Society
UK Primary Immune-deficiency Patient Support
Waverley Care
World Child Cancer
21 and Co
Action on Pain
Action on Smoking and Health - Scotland
ADHD Foundation
African-Caribbean Leukaemia Trust
African's Getting Involved
Alpha-1 Awareness UK
Alpha-1 UK Support Group
Anaphylaxis Campaign
Anticoagulation UK
Arrhythmia Alliance
Arthritis and Musculoskeletal Alliance
Association for Glycogen Storage Disease
Association for Multiple Endocrine Neoplasia Disorders
Atrial Fibrillation Association
Barts and The London Charity
Beating Bowel Cancer
Bladder and Bowel Foundation (taken over by Bladder and Bowel Community in 2017)
Bladder Health UK
Bloodwise

For peer review only

Bowel Cancer UK
Breakthrough Breast Cancer (current name Breast Cancer Now, following merger with Breast Cancer (
Breast Cancer Haven (The Haven)
British Dupuytren's Society
British Heart Foundation
British Liver Trust
British Polio Fellowship
Brittle Bone Society
Brook
Cancer 52
Cancer Research UK
Cardiac Risk in the Young
Cardiomyopathy UK
Cleft Lip and Palate Association
Coeliac UK
Community Cancer Centre
Contact a Family
Crohn's in Childhood Research Association
Cure Parkinson's Trust
Cystic Fibrosis Trust
David Lewis Centre
Dementia UK
Diabetes Research and Wellness Foundation
Douglas Bader Foundation
Epilepsy Connections
Epilepsy Scotland
Family Planning Association
Forum Link
Gauchers Association
Gay Men's Health
Haemophilia Scotland
Haemophilia Society
Heart UK
Hereditary Angioedema UK
HIV i-Base
ICUsteps
Independent Cancer Patients' Voice
International Alliance of Patients' Organizations
Juvenile Diabetes Research Foundation
Kidney Cancer Support Network
Kidney Cancer UK
Leicestershire AIDS Support Services
Life Education Wessex
Liver4Life
Lupus Europe
Macmillan Cancer Support

For peer review only

Maggie's Centre
Marie Curie Cancer Care
Melanoma UK
Men’s Health Forum
Mesothelioma UK
MPN Voice
Myeloma UK
National AIDS Manual
National Eczema Society
National Kidney Federation
National Obesity Forum
National Osteoporosis Society
Northern Ireland Chest, Heart and Stroke
OcuMel UK
Oliver King Foundation
Orchid
Organisation for Sickle Cell Anaemia Research and Thalassaemia Support
Ovacome
Ovarian Cancer Action
Pain Association Scotland
Pain Concern
Pain UK
Pancreatic Cancer Action
Pancreatic Cancer UK
Parkinson’s UK
Patients Association
PBC Foundation UK
Polycystic Kidney Disease Charity
Positive East
Prostate Cancer Support Organisation
Prostate Cancer UK (merged with Prostate Action in 2012, retaining its original name)
Pumping Marvellous Foundation
QUIT
Rarer Cancers Foundation
Restless Leg Syndrome UK
Ronald McDonald House Charities
Roy Castle Lung Cancer Foundation
Royal Free Charity
SANE
Sarcoma UK
Scoliosis Association UK
Scotland Patients Association
Scottish Epilepsy Initiative
Self Help Services
Shift.MS
Silver Star

For peer review only

South Asian Health Foundation
SUDEP Action
Target Ovarian Cancer
Tenovus Cancer Care
Thrombosis UK
Transplant Links
Trigeminal Neuralgia Association
UK Primary Immune-deficiency Patient Support
UK Sepsis Trust
Waverley Care
World Child Cancer
AADC Research Trust
Action Duchenne
Action for Pulmonary Fibrosis
Action on Pain
Action on Smoking and Health - Wales
Actionplus Foundation
Addaction
Addenbrooks Charitable Trust
African Health Policy Network
African-Caribbean Leukaemia Trust
Alcohol Concern
Alpha-1 UK Support Group
Anaphylaxis Campaign
Anticoagulation UK
Arrhythmia Alliance
Arthritis and Musculoskeletal Alliance
Arthritis Research UK
Association for Glycogen Storage Disease
Asthma and Allergy Foundation
Asthma Relief
Atrial Fibrillation Association
Basil Skyers Myeloma Foundation
Beating Bowel Cancer
Behcets Syndrome Society
Bladder and Bowel Foundation (taken over by Bladder and Bowel Community in 2017)
Bloodwise
Bone Cancer Research Trust
Brain Tumor Charity
Breast Cancer Campaign (current name Breast Cancer Now, following merger with Breakthrough Brea
Breast Cancer Haven (The Haven)
British Dupuytren's Society
British Heart Foundation
British Liver Trust
British Pregnancy Advisory Service
British Skin Foundation

For peer review only

Brook
Building for the Future
Cancer 52
Cancer Focus Northern Ireland
Cancer Research UK
Catholics for AIDS Prevention and Support
Child Growth Foundation
Children with Cancer in Malawi
Chronic Lymphocytic Leukaemia Support Association
Contact a Family
Core Charity
Crohn's in Childhood Research Association
Cure Parkinson's Trust
Cystic Fibrosis Trust
Diabetes Research and Wellness Foundation
Disabled Living
Edinburgh and Lothian Prostate Cancer Support Group
Epilepsy Connections
Epilepsy Scotland
European Parkinson’s Disease Association
Family Planning Association
Food Chain
FORCE
Forum Link
Freshwinds
Gay Men's Health
Haemophilia Society
Heart UK
Hepatitis B Positive Trust
HIV i-Base
Independent Cancer Patients' Voice
Juvenile Diabetes Research Foundation
Karen Clifford Skcin cancer charity
Kidney Cancer Support Network
Kidney Cancer UK
Leukaemia CARE
Liver4Life
Make A Wish Foundation
Melanoma UK
Mental Health Foundation
Mesothelioma UK
Migraine Action
MPN Voice
Multi Organ Transplant Support
National AIDS Manual
National Association for Pre-Menstrual Syndrome

For peer review only

National Eczema Society
National Kidney Federation
National Osteoporosis Society
Niemann-Pick UK
North of England Bone Marrow and Thalassaemia Association
Northern Ireland Rare Disease Partnership
Orchid
Ovacome
Ovarian Cancer Action
Paget's Association
Pain Concern
Pain UK
Pancreatic Cancer UK
Parkinson's UK
Patients Association
PBC Foundation UK
Polycystic Kidney Disease Charity
Positive East
Prostate Cancer Support Organisation
Prostate Cancer UK (merged with Prostate Action in 2012, retaining its original name)
Prostate Cymru
Rainbow Centre for Conductive Education
Rapid Effective Assistance For Children With Potentially Terminal Illness
Rarer Cancers Foundation
Raynaud's and Scleroderma Association (current name: Scleroderma and Raynauds UK, following the
Release
Restless Leg Syndrome UK
Revive Multiple Sclerosis Support
Roy Castle Lung Cancer Foundation
Royal Marsden Cancer Charity
Royal National Institute of Blind People
SANE
Sarcoma UK
Scleroderma Society (current name: Scleroderma and Raynauds UK, following the merger of Raynaud
Scottish Drugs Forum
Scottish Epilepsy Initiative
Silver Star
Skin Care Cymru
Sophia Forum
St Thomas Lupus Trust
Stroke Association
SUDEP Action
Tackle Prostate Cancer
Transplant Links
UK Chronic Lymphocytic Leukaemia Forum
UK Gout Society

For peer review only

UK Primary Immune-deficiency Patient Support
Walk the Walk Worldwide
Waverley Care
World Child Cancer
Action Duchenne
Action on Pain
Addaction
ADHD Foundation
African Health Policy Network
Age UK
aHUS UK
Alcohol Concern
Alpha-1 UK Support Group
Anaphylaxis Campaign
Angelman Syndrome Support Education Research Trust
Anticoagulation UK
Anxiety UK
Aplastic Anaemia Trust
Arrhythmia Alliance
Arthritis and Musculoskeletal Alliance
Association for Glycogen Storage Disease
Atrial Fibrillation Association
BackCare
Basil Skyers Myeloma Foundation
Beat Parkinson's
Beating Bowel Cancer
Bladder and Bowel Foundation (taken over by Bladder and Bowel Community in 2017)
Bloodwise
Bluebell Foundation
Body and Soul
Bone Cancer Research Trust
Breast Cancer Campaign (current name Breast Cancer Now, following merger with Breakthrough Brea
Breast Cancer Haven (The Haven)
Breast Cancer Now (created after the merger of Breast Cancer Campaign and Breakthrough Breast Ca
British Dupuytren's Society
British Heart Foundation
British Liver Trust
British Skin Foundation
Brook
Cambridge Rare Disease Network
Cancer 52
Cancer Focus Northern Ireland
Cancer Research UK
Candlelighters
Children with Cancer UK
Children's Liver Disease Foundation

For peer review only

Chronic Lymphocytic Leukaemia Support Association
CLIC Sargent Cancer Care For Children
Contact a Family
Cure Parkinson's Trust
Cystic Fibrosis Trust
Delete Blood Cancer
Diabetes UK
Diabetics with Eating Disorders
Diamond Blackfan Anaemia UK
Douglas Bader Foundation
Encephalitis Society
Food Chain
Forum Link
Freshwinds
Friends of FOP
Gauchers Association
Gay Men's Health
Haemophilia Scotland
Haemophilia Society
Haemophilia Wales
Hidradenitis Suppurativa Trust
HIV i-Base
Independent Cancer Patients' Voice
Input Diabetes
International Chronic Myeloid Leukemia Foundation
Karen Clifford Skcin cancer charity
Kidney Cancer Support Network
Kidney Cancer UK
Kidney Care UK
LATCH Welsh Children's Cancer Charity
Leeds Teaching Hospital Charitable Foundation
Little Hearts Matter
Liver4Life
Lullaby Trust
Lupus Europe
Lupus UK
Macular Society
Maggie's Centre
MAMA Academy
Melanoma UK
Mental Health Foundation
Mesothelioma UK
Movember Foundation
Multiple Births Foundation
Myasthenia Gravis Association
NARA - The Breathing Charity

For peer review only

National AIDS Manual
National Eczema Society
National Kidney Federation
National Osteoporosis Society
NMO Research Foundation
North of England Bone Marrow and Thalassaemia Association
Older People's Advocacy Alliance
Ovacome
Pancreatic Cancer UK
Patients Association
Patients On Intravenous and Nasogastric Nutrition Therapy
Pelvic Pain Support Network
Portsmouth Down Syndrome Association
Positive Action
Progressive Supranuclear Palsy Association
Prostate Cancer UK (merged with Prostate Action in 2012, retaining its original name)
QUIT
Rainbow Trust Children's Charity
Rapid Effective Assistance For Children With Potentially Terminal Illness
Rarer Cancers Foundation
Raynaud's and Scleroderma Association (current name: Scleroderma and Raynauds UK, following the
Restless Leg Syndrome UK
Rethink Mental Illness
River House Trust
Roy Castle Lung Cancer Foundation
Royal Free Charity
Royal National Institute of Blind People
Salamander Trust
SANE
Scleroderma Society (current name: Scleroderma and Raynauds UK, following the merger of Raynaud
Scottish Kidney Federation
Sense
Silver Star
Skin Care Cymru
Sophia Forum
Spectra
Spinal Injuries Association
St Thomas Lupus Trust
Steps
Stroke Association
Tackle Prostate Cancer
TB Alert
Team Margot
Teenage Cancer Trust
Tenovus Cancer Care
Thrombosis UK

For peer review only

Transverse Myelitis Society
Tree of Hope
UK Primary Immune-deficiency Patient Support
UK Thalassaemia Society
Waverley Care
World Child Cancer
York Air Ambulance
Action Bladder Cancer UK
Action Duchenne
Action on Smoking and Health - Wales
Actionplus Foundation
Addaction
ADHD Foundation
African-Caribbean Leukaemia Trust
ALD Life
Allergy UK
Alpha-1 UK Support Group
Ankylosing Spondylitis International Federation
Anthony Nolan
Anticoagulation UK
Arrhythmia Alliance
Arthritis and Musculoskeletal Alliance
Asyabi UK
Atrial Fibrillation Association
Back on Track
BackCare
Basil Skyers Myeloma Foundation
Beating Bowel Cancer
Birdshot Uveitis Society
Bladder and Bowel Foundation (taken over by Bladder and Bowel Community in 2017)
Body and Soul
Brain Tumour Research
Breast Cancer Haven (The Haven)
British Dupuytren's Society
British Liver Trust
British Skin Foundation
Brook
Cambridge Rare Disease Network
Cancer 52
Cancer Focus Northern Ireland
Cancer Research UK
Cardiac Risk in the Young
Cardiomyopathy UK
Charlie Waller Memorial Trust
Child Growth Foundation
Children's Liver Disease Foundation

For peer review only

Chronic Lymphocytic Leukaemia Support Association
CLIC Sargent Cancer Care For Children
Coppafeel!
Crohn's in Childhood Research Association
Cure and Action for Tay-Sachs
Cure Parkinson's Trust
Cystic Fibrosis Trust
Delete Blood Cancer
Diabetes UK
Diabetics with Eating Disorders
Different Strokes
Disabled Living
Encephalitis Society
Epilepsy Action
Epilepsy Scotland
Family Planning Association
Foundation for Liver Research
GIST Support UK
Guillain Barre and associated inflammatory neuropathies
Haemophilia Scotland
Haemophilia Society
Haemophilia Wales
Hafal
Headway
Heart UK
HIV i-Base
HIV Scotland
Imperial College Healthcare Charity
Independent Cancer Patients' Voice
Juvenile Diabetes Research Foundation
KAITY
Karen Clifford Skcin cancer charity
Kidney Cancer Support Network
Kidney Cancer UK
Kidney Care UK
Liver4Life
Lupus Europe
Macmillan Cancer Support
Make A Wish Foundation
Marie Curie Cancer Care
Martin Fisher Foundation
MDS UK Support Group
Melanoma Focus
Melanoma Fund
Melanoma UK
Mesothelioma UK

For peer review only

Migraine Action
Motor Neurone Disease Association
MPN Voice
Multiple Sclerosis UK
Muscular Dystrophy UK
National Attention Deficit Disorder Information and Support Service
National Cancer Research Institute
National Childbirth Trust
National Eczema Society
National Kidney Federation
National Osteoporosis Society
National Rheumatoid Arthritis Society
NMO Research Foundation
Northern Ireland Rare Disease Partnership
Olive Tree Cancer Support Centre - Crawley
Ovacome
Ovarian Cancer Action
Paget's Association
Pain Concern
Pancreatic Cancer Action
Parkinson’s UK
Patients Association
Patients On Intravenous and Nasogastric Nutrition Therapy
Paula Carr Diabetes Trust
Pelican Cancer Foundation
Pink Ribbon Foundation – Breast Cancer Charity
Polycystic Kidney Disease Charity
Progressive Supranuclear Palsy Association
QUIT
Rarer Cancers Foundation
Restless Leg Syndrome UK
Roy Castle Lung Cancer Foundation
Royal National Institute of Blind People
Samson Centre for MS
SANE
Sarcoma UK
Save Babies Through Screening Foundation UK
Scleroderma and Raynauds UK (established following the merger of Raynaud's and Scleroderma Asso
Scottish Network Children with Arthrities
Sexual Advice Association
Short Bowel Survivor and Friends
Skin Care Cymru
Somerville Foundation
Sussex Beacon
Tackle Prostate Cancer
Teenage Cancer Trust

For peer review only

Tenovus Cancer Care
Thames Valley Positive Support
Thrombosis UK
Tuberous Sclerosis Association
Visionary
Waldenstroms Macroglobulinaemia UK
Waverley Care
WellChild
Willow Foundation
World Cancer Research Fund
World Child Cancer
Young Epilepsy

For peer review only

nber of payme	Value of payments (2016 £)
3	3488.9
1	10520.8
1	15781.3
2	39258.9
2	21041.7
2	563.6
1	1057.3
1	15860.2
12	20260.5
6	7381.0
5	37832.9
36	227017.3
5	36010.8
3	3193.8
3	4145.2
13	415672.2
7	6165.9
1	10520.8
1	473.4
1	981.5
2	5298.0
1	6312.5
2	599.7
1	117.8
1	420.8
4	33929.7
5	33140.6
3	5681.3
10	660846.1
1	1578.1
1	473.4
1	736.5
1	7890.6
1	252.5
1	58.9
1	4187.3
1	8416.7
3	12625.0
1	254.6
2	12650.6
1	526.0
1	2104.2
29	315722.5
1	5260.4

For peer review only

1	37.6
1	21362.0
3	7680.2
2	747.0
3	9153.1
4	12414.6
1	153.6
9	50719.9
1	37.6
1	757.5
3	24923.9
4	16970.1
1	2104.2
1	2099.5
2	4103.1
3	122044.1
1	10520.8
8	24125.3
2	925.8
5	19768.6
1	157.8
1	883.8
1	241.4
1	5260.4
2	21041.7
2	79095.6
2	25776.0
2	18170.9
7	215976.6
1	1420.3
9	20541.9
8	144188.0
2	1157.3
3	13082.8
1	1052.1
1	2104.2
1	2104.2
4	3000.9
4	6689.5
1	631.3
4	16916.6
1	8416.7
10	66089.5
3	1188.0
1	16496.7
1	473.4

1	1052.1
7	19502.5
16	134019.4
5	5946.4
1	736.5
11	136368.8
1	417.7
1	1052.1
1	473.4
1	210.4
1	2865.9
4	40505.2
14	161724.0
2	6327.7
1	1052.1
1	26302.1
3	4771.9
1	5260.4
1	45554.2
1	736.5
5	19805.5
2	4208.3
1	10520.8
2	5643.4
2	9021.6
2	16096.9
1	1028.5
6	5139.5
1	2519.9
1	205.7
1	18513.2
1	129.6
2	16456.2
2	15942.0
1	25712.8
20	73150.1
6	78372.7
5	52570.4
1	2057.0
1	0.0
20	108390.1
1	205.7
5	30968.5
2	21418.9
3	8377.2
1	236.7

For peer review only

1	10285.1
6	29896.9
1	771.4
3	2611.4
3	742.3
2	15633.4
1	0.0
1	15427.7
3	23347.3
4	32912.4
9	33791.8
1	462.8
1	518.7
1	62.0
1	129.6
1	1028.5
1	7713.8
2	7207.8
2	3983.1
3	1016.7
1	1892.5
1	2592.9
1	2057.0
1	778.1
1	2057.0
1	1285.6
4	41449.1
2	5425.4
6	23100.4
2	2879.8
4	6428.2
5	65192.3
25	188350.6
4	40673.6
4	16263.9
1	2057.0
1	3085.5
3	76571.8
4	51906.0
1	48032.0
3	32912.4
2	21290.2
1	518.7
1	360.0
1	8733.1
7	21486.0

For peer review only

1	2809.2
1	251.5
3	19582.9
2	5130.7
2	10626.4
1	1028.5
8	207709.3
4	23005.8
2	2327.5
7	62885.3
1	2351.2
8	22156.9
1	1028.5
1	1028.5
1	510.9
1	3085.5
1	2079.9
2	5656.8
1	10285.1
6	1635.3
5	5471.7
3	6582.5
1	771.4
1	8228.1
3	8964.6
9	61969.6
1	475.1
2	9745.2
2	579.1
1	1626.1
4	3594.9
2	31883.9
3	22722.3
13	122253.9
1	2571.3
1	771.4
14	124200.5
1	11321.9
1	20428.5
1	1028.5
1	277.7
1	1028.5
1	2571.3
2	514.3
3	41140.5
6	48340.1

For peer review only

1	35998.0
1	1563.3
1	0.0
1	2571.3
3	14399.2
1	771.4
1	0.0
4	19688.8
1	0.0
1	4114.1
2	20914.8
1	5070.3
2	3042.2
1	595.8
2	4056.2
1	2028.1
1	2940.8
9	122041.7
1	3042.2
2	20854.0
3	20857.1
4	30421.7
1	60843.4
1	608.4
8	60166.5
6	89084.8
9	85180.7
3	583.1
1	7098.4
1	507.0
1	1014.1
20	86721.7
1	7098.4
8	133200.9
1	20281.1
6	234694.2
3	16934.7
2	10140.6
1	12675.7
5	65913.7
2	12417.3
1	11465.2
2	354.9
2	33463.9
1	507.0
3	3549.2

For peer review only

3	25604.9
1	243.4
5	39041.2
7	10280.6
9	425099.6
1	4089.1
1	1521.1
1	20179.7
1	20281.1
1	7605.4
1	8112.4
2	7098.4
2	342.6
4	7250.5
1	2433.7
3	17827.1
2	804.5
1	2028.1
3	3442.7
2	24984.5
3	22917.7
1	3042.2
1	507.0
1	6084.3
1	3042.2
3	11734.7
15	112989.0
18	178932.5
1	5070.3
2	10647.6
1	3042.2
8	205566.7
5	43874.2
1	507.0
5	31800.8
8	78082.3
5	59736.0
1	507.0
5	133910.2
1	1216.9
1	9944.8
1	33463.9
1	22682.4
1	6084.3
7	82397.2
1	152.1

For peer review only

1	304.2
6	80617.5
3	77459.8
2	16267.8
3	4563.3
1	2535.1
1	248.6
1	10951.8
1	19267.1
1	15210.8
1	3042.2
3	6601.5
9	38939.8
3	6135.0
9	69674.0
2	608.4
1	15210.8
1	4563.3
1	461.8
5	26839.5
1	639.6
1	579.3
1	2738.0
14	194892.6
2	1139.3
1	10063.5
2	3338.3
1	40562.2
13	179410.6
1	354.9
9	282414.7
2	26365.5
1	1014.1
1	671.8
2	4056.2
1	76054.2
1	19013.6
2	1014.1
1	5070.3
2	835.0
9	65132.8
1	770.7
4	9011.9
1	760.5
1	4056.2
2	27886.5

For peer review only

3	21428.0
1	405.6
3	19058.2
7	163600.7
5	32262.1
1	1262.5
9	189819.4
3	16160.0
4	10327.3
2	555.5
1	25250.0
6	52988.6
1	60600.0
4	3030.0
1	303.0
18	118527.0
1	3282.5
1	1616.0
10	108324.5
8	61610.0
4	18555.7
14	107478.3
1	3535.0
1	18852.7
1	5050.0
9	77810.4
11	1473965.2
6	40753.5
1	303.0
4	57945.7
1	2020.0
1	9090.0
1	19705.1
1	0.0
1	31916.0
1	666.6
5	17018.5
1	4155.1
1	8080.0
1	1574.6
5	63630.0
2	12378.2
14	662211.6
1	0.0
1	151.5
1	1010.0

For peer review only

5	47672.0
2	4559.1
1	5050.0
1	10254.5
2	9595.0
2	14402.6
44	899692.9
3	1347.3
1	15150.0
1	606.0
1	505.0
1	5050.0
1	13907.7
1	8888.0
1	0.0
3	19493.0
1	505.0
8	13847.3
14	190024.6
5	5972.6
2	6019.6
1	8080.0
2	4545.0
1	30322.2
1	34105.2
3	27270.0
3	20200.0
10	56661.0
1	526.2
1	1010.0
1	353.5
1	252.5
8	131184.9
1	0.0
1	25668.1
1	707.0
1	15.2
1	445.9
1	202.0
6	124798.6
1	1212.0
1	10100.0
1	1010.0
1	505.0
1	2020.0
1	0.0

For peer review only

7	47908.3
1	202.0
5	61027.2
8	95647.0
1	5050.0
1	1212.0
2	30300.0
2	32572.5
4	122715.0
9	64371.2
1	1520.2
1	1466.7
1	151.5
1	0.0
1	151.5
2	5302.5
2	32115.0
1	1010.0
1	2462.4
10	126755.0
3	20200.0
1	3030.0
1	757.5
1	252.5
14	164788.4
1	505.0
7	230060.8
1	303.0
2	15840.8
2	2473.5
1	505.0
1	151.5
1	10100.0
4	18060.7
1	5050.0
1	1010.0
1	252.5
2	62115.0
1	151.5
9	47571.0
5	83197.3
2	27401.3
1	5050.0
4	3838.0
4	70809.1
2	2525.0

For peer review only

1	15150.0
1	151.5
2	20701.0
1	4040.0
5	15361.1
1	50499.9
1	0.0
1	101.0
5	11772.1
1	5000.0
1	5000.0
2	45000.0
4	6620.0
1	15000.0
1	262.3
3	10160.5
1	1200.0
6	38722.8
1	20000.0
18	103102.5
6	46355.0
5	25000.0
1	1500.0
14	91536.8
1	3809.2
2	4000.0
2	9250.0
15	64682.6
1	10000.0
6	125466.9
2	40000.0
1	262.3
1	15000.0
1	9000.0
8	43810.0
2	2080.2
1	5000.0
3	7745.7
7	80375.0
1	824.2
15	35685.8
6	5808.1
4	1819.2
1	1000.0
2	5937.2
1	15000.0

For peer review only

6	18757.8
1	853.0
1	10000.0
2	650.0
2	120.0
3	9914.0
6	250684.0
1	262.3
55	972606.1
1	200.0
5	10786.9
1	500.0
1	262.3
1	1624.0
2	1351.0
1	15000.0
1	57537.0
5	35143.0
2	6400.0
2	13000.0
14	158595.7
5	7748.3
1	4000.0
1	150.0
15	227311.4
2	11500.0
1	3000.0
1	4000.0
5	15115.4
1	36000.0
1	2000.0
1	10000.0
4	32597.2
11	38214.9
1	228.0
10	57018.2
2	21101.4
14	98362.3
1	257.6
1	150.0
1	15000.0
3	7068.8
3	46207.0
2	15000.0
8	87500.0
3	3900.0

For peer review only

3	1139.7
2	280.0
2	2355.5
1	603.4
1	6000.0
1	45.0
1	1000.0
1	9600.0
1	300.0
4	48000.0
13	118394.2
15	121433.8
2	1600.0
1	1500.0
1	3400.0
1	15000.0
1	10000.0
1	7405.6
1	1685.0
1	1250.0
4	1984.0
11	83416.0
4	28328.7
1	400.0
1	8500.0
1	10000.0
1	44000.0
2	412.3
1	5000.0
9	105050.0
1	4000.0
18	165556.8
9	178153.0
1	4500.0
1	20000.0
1	3000.0
1	981.0
1	2000.0
3	1139.3
1	30000.0
1	224.0
2	850.0
1	1000.0
1	1000.0
3	51000.0
2	2538.0

For peer review only

3	51568.0
1	5000.0
7	9052.8
1	23754.0
2	15000.0
3	9851.0
7	56002.3
2	262000.0
1	257.6
1	5000.0
3	91163.6
1	18000.0

For peer review only

Web Supplement 15. Recipients - payments missing from patient organisation data (all years)

Patient organisation name

21 and Co
AADC Research Trust
Action Bladder Cancer UK
Action Duchenne
Action on Pain
Action on Smoking and Health - Scotland
Actionplus Foundation
Addenbrooks Charitable Trust
African-Caribbean Leukaemia Trust
African's Getting Involved
Age Related Diseases and Health Trust
aHUS UK
ALD Life
Allergy Research Foundation
Alpha-1 Awareness UK
Alpha-1 UK Support Group
Alzheimer Scotland
Alzheimers Dementia Support
Angelman Syndrome Support Education Research Trust
Ankylosing Spondylitis International Federation
Anticoagulation UK
Aplastic Anaemia Trust
Arrhythmia Alliance
Arthritis and Musculoskeletal Alliance
Asthma and Allergy Foundation
Asthma Relief
Asyabi UK
Atrial Fibrillation Association
Back on Track
Barts and The London Charity
Basil Skyers Myeloma Foundation
Beat Parkinson’s
Beating Bowel Cancer
Behcets Syndrome Society
Bipolar Scotland
Bipolar UK
Birdshot Uveitis Society
Bladder and Bowel Foundation (taken over by Bladder and Bowel Community in 2017)
Blood Pressure UK
Bluebell Foundation
Body and Soul
Bone Cancer Research Trust
Brain Charity
Brain Tumor Charity

For peer review only

Brain Tumour Research
Breast Cancer Campaign (current name Breast Cancer Now, following merger with Breakthrough Brea
Breast Cancer Haven (The Haven)
British Dupuytren's Society
British Heart Foundation
British Obesity Society
British Polio Fellowship
British Skin Foundation
Brook
Building for the Future
Cambridge Rare Disease Network
Cancer 52
Cancer Focus Northern Ireland
Cancer Research UK
Candlelighters
Cardiac Risk in the Young
Cardiomyopathy UK
Catholics for AIDS Prevention and Support
Charlie Waller Memorial Trust
Children with Cancer UK
Children's Liver Disease Foundation
Children’s Heart Unit Fund
Chinese National Healthy Living Centre
Chronic Lymphocytic Leukaemia Support Association
Cleft Lip and Palate Association
CLIC Sargent Cancer Care For Children
Community Cancer Centre
Contact a Family
Coping with Cancer in Leicestershire and Rutland
Crohn's in Childhood Research Association
Cure Parkinson's Trust
Cystic Fibrosis Trust
David Lewis Centre
Deaf Education through Listening and Talking
Delete Blood Cancer
Dementia UK
Diabetes Research and Wellness Foundation
Diabetics with Eating Disorders
Diamond Blackfan Anaemia UK
Different Strokes
Douglas Bader Foundation
Edinburgh and Lothian Prostate Cancer Support Group
Encephalitis Society
Epilepsy Connections
Epilepsy Scotland
FORCE

For peer review only

Forum Link
Freshwinds
Group B Strep Support UK
Guillain Barre and associated inflammatory neuropathies
Haemophilia Society
Haemophilia Wales
Hafal
Headway
Health and Social Care Alliance Scotland
Heartbeat
Hepatitis B Positive Trust
HIV i-Base
Hughes Syndrome Foundation
ICUsteps
Imperial College Healthcare Charity
Independent Cancer Patients' Voice
Input Diabetes
KAITY
Karen Clifford Skcin cancer charity
Kidney Cancer Support Network
Kidney Cancer UK
Kidney Care UK
LATCH Welsh Children's Cancer Charity
Leeds Teaching Hospital Charitable Foundation
Leicestershire AIDS Support Services
Life Education Wessex
Little Hearts Matter
Liver4Life
Lullaby Trust
Lupus Europe
Lupus UK
Macular Society
Maggie's Centre
Make A Wish Foundation
Malnutrition and Nutritional Care in the UK
MAMA Academy
Marie Curie Cancer Care
Martin Fisher Foundation
Melanoma UK
Mesothelioma UK
Motor Neurone Disease Association
Movember Foundation
MPN Voice
Multi Organ Transplant Support
Multiple Births Foundation
Multiple Sclerosis UK

For peer review only

Myasthenia Gravis Association
Myrovlytis Trust
NARA - The Breathing Charity
National Association for Pre-Menstrual Syndrome
National Cancer Research Institute
National Eczema Society
National Kidney Federation
National Obesity Forum
National Osteoporosis Society
NMO Research Foundation
North of England Bone Marrow and Thalassaemia Association
Northern Ireland Chest, Heart and Stroke
Northern Ireland Rare Disease Partnership
OcuMel UK
Olive Tree Cancer Support Centre - Crawley
Oliver King Foundation
Orchid
Organisation for Sickle Cell Anaemia Research and Thalassaemia Support
Ovacome
Paget's Association
Pain Association Scotland
Pain Concern
Pain UK
Patients Association
Patients On Intravenous and Nasogastric Nutrition Therapy
Paula Carr Diabetes Trust
Pelican Cancer Foundation
Pelvic Pain Support Network
Pink Ribbon Foundation – Breast Cancer Charity
Polycystic Kidney Disease Charity
Portsmouth Down Syndrome Association
Positive Action
Prader-Willi Syndrome Association UK
Progressive Supranuclear Palsy Association
Prostate Cancer Research Centre
Prostate Cymru
QUIT
Rainbow Centre for Conductive Education
Rainbow Trust Children's Charity
Rarer Cancers Foundation
Restless Leg Syndrome UK
Richmond Fellowship
River House Trust
Ronald McDonald House Charities
Roy Castle Lung Cancer Foundation
Royal Marsden Cancer Charity

For peer review only

Salamander Trust
Samson Centre for MS
SANE
Scoliosis Association UK
Scotland Patients Association
Scottish Drugs Forum
Scottish Epilepsy Initiative
Scottish Kidney Federation
Scottish Network Children with Arthrities
SeeAbility
Self Help Services
Sense
Service by Emergency Response Volunteers - Suffolk and Cambridgeshire
Short Bowel Survivor and Friends
Silver Star
Skin Care Cymru
Somerville Foundation
Sophia Forum
Spectra
Spinal Injuries Association
Spinal Injuries Scotland
St Thomas Lupus Trust
Steps
SUDEP Action
Sussex Beacon
Tackle Prostate Cancer
Team Margot
Tenovus Cancer Care
Thames Valley Positive Support
Thrombosis UK
Transplant Links
Transverse Myelitis Society
Tree of Hope
Trigeminal Neuralgia Association
UK Chronic Lymphocytic Leukaemia Forum
UK Gout Society
UK Sepsis Trust
Walk the Walk Worldwide
Waverley Care
Willow Foundation
World Cancer Research Fund
World Child Cancer
York Air Ambulance

For peer review only

Number of payments	Value of payments (2016 £)
1	1028.5
1	5070.3
1	101.0
12	47076.4
12	13947.1
1	2519.9
2	7940.8
1	3042.2
6	70151.6
1	129.6
2	39258.9
1	25250.0
1	262.3
2	21041.7
2	16456.2
5	138585.3
2	563.6
1	1057.3
1	303.0
6	38722.8
76	375206.6
1	1616.0
34	329518.1
32	262194.0
1	507.0
1	1014.1
1	1500.0
104	621144.3
1	3809.2
1	205.7
4	35201.1
1	5050.0
42	342673.1
1	20281.1
3	3193.8
3	4145.2
1	10000.0
38	2271217.4
1	10520.8
2	776.4
6	97945.7
3	12160.6
1	981.5
1	12675.7

For peer review only

1	262.3
7	81316.2
5	47893.8
8	55592.3
7	1881.6
1	420.8
1	0.0
6	9784.6
12	95961.9
1	243.4
4	9320.3
26	249099.2
13	29164.3
57	1817634.9
1	0.0
7	6270.9
5	2338.0
1	4089.1
1	1000.0
2	624.9
2	16010.0
1	736.5
1	252.5
12	86711.0
2	120.9
3	5412.1
1	1028.5
3	20369.3
1	4187.3
9	27581.2
9	24748.9
17	281196.7
1	1892.5
1	526.0
3	14664.9
1	2592.9
3	6594.9
4	1547.3
1	15150.0
5	10786.9
2	1384.1
2	804.5
2	767.3
2	4085.1
7	6116.9
1	507.0

For peer review only

6	26164.4
2	11930.2
1	153.6
2	6400.0
57	577521.6
10	13720.9
1	4000.0
1	150.0
1	37.6
1	757.5
1	5070.3
13	63461.6
1	2104.2
2	4156.5
1	4000.0
11	29891.2
1	30322.2
1	2000.0
10	91665.0
9	101336.2
37	183714.4
2	754.3
1	1010.0
1	353.5
4	22216.1
1	518.7
1	252.5
24	248299.0
1	0.0
4	55502.5
1	707.0
1	15.2
3	3412.9
2	764.6
1	883.8
1	202.0
3	643.0
1	15000.0
24	386833.4
7	34571.2
2	280.0
1	1010.0
6	44237.3
1	6084.3
1	505.0
1	603.4

For peer review only

1	2020.0
1	1420.3
1	0.0
1	152.1
1	1000.0
14	23675.7
30	396718.0
3	3508.5
35	326740.6
3	6650.0
4	5775.3
1	1028.5
2	4035.1
2	2080.6
1	3400.0
1	510.9
2	3334.2
2	4184.1
6	64181.1
3	24720.6
10	4636.2
11	16888.4
10	30100.6
48	345520.4
5	29848.9
1	400.0
1	8500.0
4	2654.7
1	10000.0
4	68956.0
1	151.5
1	0.0
1	473.4
3	563.8
1	1052.1
1	639.6
13	79339.8
1	579.3
1	1010.0
62	682970.9
5	12939.6
1	736.5
1	252.5
1	771.4
70	770325.1
1	354.9

For peer review only

2	720.7
1	4500.0
7	83686.8
1	277.7
1	1028.5
2	4056.2
2	78625.5
1	505.0
3	1139.3
1	473.4
2	514.3
1	151.5
1	210.4
1	224.0
12	117958.9
8	19924.8
3	7327.7
2	10120.3
1	1010.0
1	252.5
1	1052.1
4	62950.0
1	151.5
2	2334.0
1	1000.0
13	148469.6
1	5050.0
9	170502.5
2	5736.5
17	45782.5
2	1531.9
1	15150.0
1	151.5
3	4208.3
1	4056.2
3	38407.4
1	0.0
1	405.6
18	103557.2
1	257.6
1	5000.0
15	342275.9
1	0.0

For peer review only

Web Supplement 16. Shared ties in industry and patient organisation data based on the number an

Ties calculated based on the number of payments

Total number of ties reported in industry data involving at least one payment
Total number of ties reported in patient organisation data involving at least one payment

Number of shared ties (donors and recipients are the same in industry and patient organisation data)
Shared ties as a share of all ties in industry data
Shared ties as a share of all ties in patient organisation data

Number of ties involving the same number of payments
Number of ties involving the same number of payments as a share of shared ties

Number of payments forming ties with the equal number of payments in both datasets
Payments forming ties with the equal number of payments in both datasets as a a share of all paymer
Payments forming ties with the equal number of payments in both datasets as a a share of all paymer

Ties calculated based on the value of payments

Total number of ties reported in industry data involving at least one payment with value >£0
Total number of ties reported in patient data involving at least one payment with value >£0

Number of shared ties (donors and recipients are the same in industry and patient organisation data)
Shared ties as a share of all ties in industry data
Shared ties as a share of all ties in patient organisation data

Number of shared ties involving the same value of payments
Shared ties involving the same alue of payments as a share of shared ties

Value of payments in which donors and recipients provided the same value (2016 £)
Value of shared payments as a share of payments with values >£0 in industry data
Value of shared payments as a share of payments with values >£0 in patient organisation data

For peer review only

All years	2012	2013	2014	2015	2016
1101	369	366	432	479	523
626	252	276	293	319	287
428	110	122	146	168	160
38.9%	29.8%	33.3%	33.8%	35.1%	30.6%
68.4%	43.7%	44.2%	49.8%	52.7%	55.7%
87	45	66	69	89	64
20.3%	40.9%	54.1%	47.3%	53.0%	40.0%
162	47	69	79	97	73
3.8%	6.1%	9.8%	9.6%	10.4%	6.7%
9.8%	16.2%	21.2%	22.8%	26.9%	21.7%
1088	366	357	431	470	522
326	127	122	119	150	140
34	21	24	28	32	24
3.1%	5.7%	6.7%	6.5%	6.8%	4.6%
10.4%	16.5%	19.7%	23.5%	21.3%	17.1%
20	17	22	26	30	23
58.8%	81.0%	91.7%	92.9%	93.8%	95.8%
325,108.4	216,055.8	201,362.3	251,964.6	517,139.2	482,216.0
0.6%	2.8%	3.3%	2.9%	4.5%	2.4%
1.0%	6.2%	4.6%	4.8%	10.7%	3.2%

For peer review only

Web Supplement 17. Overlap between recipient lists reported by donors and patient organisa

	Total number of donors with at least one payment	Minimum overlap with patient organisation data	Number of donors with minium overlap with patient organisation data	Donors with minium overlap with patient organisation data as a share of all donors	Number of donors with less than 50% overlap with patient organisation data	Donors with less than 50% overlap with patient organisation data as a share of all donors
All years	63	0.0%	6	9.5%	40	63.5%
2012	30	0.0%	7	23.3%	23	76.7%
2013	38	0.0%	9	23.7%	25	65.8%
2014	45	0.0%	12	26.7%	35	77.8%
2015	51	0.0%	11	21.6%	33	64.7%
2016	44	0.0%	10	22.7%	32	72.7%

For peer review only

ation data - yearly breakdown based on the number of payments

Maximum overlap with patient organisation data	Number of donors with maximum overlap with patient organisation data	Donors with maximum overlap with patient organisation data as a share of all donors
100.0%	3	4.8%
100.0%	1	3.3%
100.0%	4	10.5%
100.0%	3	6.7%
100.0%	3	5.9%
100.0%	3	6.8%

For peer review only

Web Supplement 18. Overlap between recipient lists reported by donors and patient organisatio

	Number of donors with at least one payemnt with the value >£0	Minimum overlap with patient organisation data	Number of donors with minium overlap with patient organisation data	Donors with minium overlap with patient organisation data as a share of all donors	Number of donors with less than 50% overlap with patient organisation data	Donors with less than 50% overlap with patient organisation data as a share of all donors
All years	62	0.0%	17	27.4%	56	90.3%
2012	30	0.0%	13	43.3%	29	96.7%
2013	38	0.0%	18	47.4%	36	94.7%
2014	45	0.0%	26	57.8%	45	100.0%
2015	50	0.0%	21	42.0%	42	84.0%
2016	44	0.0%	15	34.1%	39	88.6%

For peer review only

on data - yearly breakdown based on the value of payments

Maximum overlap with patient organisation data	Number of donors with maximum overlap with patient organisation data	Donors with maximum overlap with patient organisation data as a share of all donors
100.0%	2	3.2%
50.0%	1	3.3%
66.7%	1	2.6%
40.0%	1	2.2%
100.0%	1	2.0%
66.7%	1	2.3%

For peer review only

Web Supplement 19. Overlap between donor lists reported by recipeints and industry data - y

	Number of recipients with at least 1 payment	Minimum overlap with industry data	Number of recipients with minium overlap with industry data	Recipients with minium overlap with industry data as a share of all recipients	Number of recipients with less than 50% overlap with industry data	Recipients with less than 50% overlap with industry data as a share of all recipients
All years	200	0.0%	14	7.0%	32	16.0%
2012	103	0.0%	37	35.9%	51	49.5%
2013	102	0.0%	38	37.3%	50	49.0%
2014	116	0.0%	34	29.3%	49	42.2%
2015	126	0.0%	37	29.4%	47	37.3%
2016	117	0.0%	28	23.9%	40	34.2%

For peer review only

yearly breakdown based on the number of payments

Maximum overlap with industry data	Number of recipients with maximum overlap with industry data	Recipeints with maximum overlap with industry data as a share of all recipients
100.0%	102	51.0%
100.0%	29	28.2%
100.0%	24	23.5%
100.0%	41	35.3%
100.0%	49	38.9%
100.0%	50	42.7%

For peer review only

Web Supplement 20. Overlap between donor lists eported by recipeints and industry data - yearl

	Number of patient organisations with at least one payemnt with the value >0	Minimum overlap with industry data	Number of recipients with minium overlap with industry data	Recipients with minium overlap with industry data as a share of all recipients	Number of recipients with less than 50% overlap with industry data	Recipients with less than 50% overlap with industry data as a share of all recipients
All years	121	0.0%	12	9.9%	18	14.9%
2012	57	0.0%	22	38.6%	30	52.6%
2013	51	0.0%	19	37.3%	26	51.0%
2014	58	0.0%	17	29.3%	21	36.2%
2015	72	0.0%	22	30.6%	26	36.1%
2016	57	0.0%	10	17.5%	15	26.3%

For peer review only

ly breakdown based on the value of payments

Maximum overlap with industry data	Number of recipients with maximum overlap with industry data	Recipeints with maximum overlap with industry data as a share of all recipients
100.0%	71	58.7%
100.0%	16	28.1%
100.0%	14	27.5%
100.0%	27	46.6%
100.0%	35	48.6%
100.0%	31	54.4%

For peer review only

STROBE Statement—Checklist of items that should be included in reports of ***cohort studies***

	Item No	Recommendation	Page No
**Title and abstract**	1	(*a*) Indicate the study's design with a commonly used term in the title or the abstract	4
		(*b*) Provide in the abstract an informative and balanced summary of what was done and what was found	4
**Introduction**			
Background/rationale	2	Explain the scientific background and rationale for the investigation being reported	5
Objectives	3	State specific objectives, including any prespecified hypotheses	5
**Methods**			
Study design	4	Present key elements of study design early in the paper	6
Setting	5	Describe the setting, locations, and relevant dates, including periods of recruitment, exposure, follow-up, and data collection	6
Participants	6	(*a*) Give the eligibility criteria, and the sources and methods of selection of participants. Describe methods of follow-up	6
		(*b*) For matched studies, give matching criteria and number of exposed and unexposed	N/A
Variables	7	Clearly define all outcomes, exposures, predictors, potential confounders, and effect modifiers. Give diagnostic criteria, if applicable	7
Data sources/ measurement	8*	For each variable of interest, give sources of data and details of methods of assessment (measurement). Describe comparability of assessment methods if there is more than one group	7
Bias	9	Describe any efforts to address potential sources of bias	6
Study size	10	Explain how the study size was arrived at	6
Quantitative variables	11	Explain how quantitative variables were handled in the analyses. If applicable, describe which groupings were chosen and why	7
Statistical methods	12	(*a*) Describe all statistical methods, including those used to control for confounding	7
		(*b*) Describe any methods used to examine subgroups and interactions	N/A
		(*c*) Explain how missing data were addressed	6
		(*d*) If applicable, explain how loss to follow-up was addressed	N/A
		(*e*) Describe any sensitivity analyses	N/A
**Results**			
Participants	13*	(a) Report numbers of individuals at each stage of study—eg numbers potentially eligible, examined for eligibility, confirmed eligible, included in the study, completing follow-up, and analysed	7
		(b) Give reasons for non-participation at each stage	N/A
		(c) Consider use of a flow diagram	Web supplement 1 and 2
Descriptive data	14*	(a) Give characteristics of study participants (eg demographic, clinical, social) and information on exposures and potential confounders	7-8
		(b) Indicate number of participants with missing data for each variable of interest	6,16
		(c) Summarise follow-up time (eg, average and total amount)	Table 1
Outcome data	15*	Report numbers of outcome events or summary measures over time	7-20

For peer review only - http://bmjopen.bmj.com/site/about/guidelines.xhtml

For peer review only

For peer review only - http://bmjopen.bmj.com/site/about/guidelines.xhtml

Main results	16	(*a*) Give unadjusted estimates and, if applicable, confounder-adjusted estimates and their precision (eg, 95% confidence interval). Make clear which confounders were adjusted for and why they were included	7-20
		(*b*) Report category boundaries when continuous variables were categorized	10-15
		(*c*) If relevant, consider translating estimates of relative risk into absolute risk for a meaningful time period	N/A
Other analyses	17	Report other analyses done—eg analyses of subgroups and interactions, and sensitivity analyses	N/A
**Discussion**			
Key results	18	Summarise key results with reference to study objectives	20-22
Limitations	19	Discuss limitations of the study, taking into account sources of potential bias or imprecision. Discuss both direction and magnitude of any potential bias	22-23
Interpretation	20	Give a cautious overall interpretation of results considering objectives, limitations, multiplicity of analyses, results from similar studies, and other relevant evidence	23
Generalisability	21	Discuss the generalisability (external validity) of the study results	22
**Other information**			
Funding	22	Give the source of funding and the role of the funders for the present study and, if applicable, for the original study on which the present article is based	2

*Give information separately for exposed and unexposed groups.

Note: An Explanation and Elaboration article discusses each checklist item and gives methodological background and published examples of transparent reporting. The STROBE checklist is best used in conjunction with this article (freely available on the Web sites of PLoS Medicine at http://www.plosmedicine.org/, Annals of Internal Medicine at http://www.annals.org/, and Epidemiology at http://www.epidem.com/). Information on the STROBE Initiative is available at http://www.strobe-statement.org.

For peer review only

BMJ Open

An underreported relationship: a comparative study of pharmaceutical industry and patient organisation payment disclosures in the UK (2012-2016)

Journal:	*BMJ Open*
Manuscript ID	bmjopen-2020-037351.R1
Article Type:	Original research
Date Submitted by the Author:	08-Apr-2020
Complete List of Authors:	Ozieranski, Piotr; University of Bath, Social and Policy Sciences<br>Csanádi, Marcell ; Syreon Research Institute<br>Rickard, Emily; University of Bath, Social and Policy Sciences<br>Mulinari, Shai; Lunds Universitet, Sociology
**Primary Subject Heading**:	Health policy
Secondary Subject Heading:	Ethics, Sociology
Keywords:	Health policy < HEALTH SERVICES ADMINISTRATION & MANAGEMENT, Protocols & guidelines < HEALTH SERVICES ADMINISTRATION & MANAGEMENT, MEDICAL ETHICS

SCHOLARONE™
Manuscripts

I, the Submitting Author has the right to grant and does grant on behalf of all authors of the Work (as defined in the below author licence), an exclusive licence and/or a non-exclusive licence for contributions from authors who are: i) UK Crown employees; ii) where BMJ has agreed a CC-BY licence shall apply, and/or iii) in accordance with the terms applicable for US Federal Government officers or employees acting as part of their official duties; on a worldwide, perpetual, irrevocable, royalty-free basis to BMJ Publishing Group Ltd ("BMJ") its licensees and where the relevant Journal is co-owned by BMJ to the co-owners of the Journal, to publish the Work in this journal and any other BMJ products and to exploit all rights, as set out in our licence.

The Submitting Author accepts and understands that any supply made under these terms is made by BMJ to the Submitting Author unless you are acting as an employee on behalf of your employer or a postgraduate student of an affiliated institution which is paying any applicable article publishing charge ("APC") for Open Access articles. Where the Submitting Author wishes to make the Work available on an Open Access basis (and intends to pay the relevant APC), the terms of reuse of such Open Access shall be governed by a Creative Commons licence – details of these licences and which Creative Commons licence will apply to this Work are set out in our licence referred to above.

Other than as permitted in any relevant BMJ Author's Self Archiving Policies, I confirm this Work has not been accepted for publication elsewhere, is not being considered for publication elsewhere and does not duplicate material already published. I confirm all authors consent to publication of this Work and authorise the granting of this licence.

For peer review only

An underreported relationship: a comparative study of pharmaceutical industry and patient organisation payment disclosures in the UK (2012-2016)

Piotr Ozieranski,[1*] Marcell Csanadi,[2] Emily Rickard,[3] Shai Mulinari [4]

[1] – Department of Social and Policy Sciences, University of Bath, Claverton Down, Bath, UK

[2] – Syreon Research Institute, Budapest, Hungary

[3] – Department of Social and Policy Sciences, University of Bath, Claverton Down, Bath, UK

[4] – Department of Sociology, Lund University, Lund, Sweden

*Corresponding author email: p.ozieranski@bath.ac.uk

Key words: pharmaceutical industry, patient organisations, conflicts of interest, financial disclosures, transparency

Wordcount: 4,455

For peer review only

Abstract

Objectives: To examine the underreporting of pharmaceutical company payments to patient organisations by donors and recipients.

Design: Comparative descriptive analysis of payments disclosed on drug company and charity regulator websites.

Setting: UK.

Participants: 87 donors (drug companies) and 425 recipients (patient organisations) reporting payments in 2012-2016.

Main outcome measures: Number and value of payments reported by donors and recipients; differences in reported payments from/to the same donors and recipients; payments reported in either dataset but not the other one; agreement between donor-recipient ties established by payments; overlap between donor and recipient lists and, respectively, industry and patient organisation data.

Results: Of 87 donors, 63 (72.4%) reported payments but 84 (96.6%) were mentioned by recipients. Although donors listed 425 recipients, only 200 (47.1%) reported payments. The number and value of payments reported by donors were 259.8% and 163.7% greater than those reported by recipients, respectively. The number of donors with matching payment numbers and values in both datasets were 3.4% and 0%, respectively; for recipients these figures were 7.8% and 1.9%. There were 24 and 3 donors missing from industry and patient organisation data during the entire study period, representing 38.1% and 3.6% of those in the respective datasets. The share of donor-recipient ties in which industry and patient organisation data agreed about donors and recipients was 38.9% and 68.4% in each dataset, respectively. Of 63 donors reporting payments, only 3 (4.8%) had their recipient lists fully overlapping with patient organisation data. Of 200 recipients reporting industry funding, 102 (51.0%) had their donor lists fully overlapping with industry data.

Conclusions: Both donors and recipients underreported payments. Existing donor and recipient disclosure systems cannot manage potential conflicts of interest associated with industry payments. Increased standardisation could limit the underreporting by each side but only an integrated donor-recipient database could eliminate it.

Strengths and limitations

- We examine the underreporting of pharmaceutical industry payments to patient organisations using large samples of donors and recipients over a period of time
- We systematically compare the underreporting by donors and recipients using five complementary measures at different levels of analysis
- One key limitation is that the samples of donors and recipients were not exhaustive
- The full extent of underreporting remains unknown as no definitive list of payments exists

For peer review only - http://bmjopen.bmj.com/site/about/guidelines.xhtml

Introduction

Many patient organisations accept funding from drug companies. A recent UK analysis showed that the industry donated over £57m (€65m; $73m) to 508 patient organisations, with the annual sum more than doubling from 2012 to 2016.[1] Another study of 289 US patient organisations found that 156 (67%) received funding from for-profit companies, with a median proportion of 45% of their income coming from drug, medical device, or biotechnology companies.[2] Although industry funding may benefit patient organisations,[3 4] it raises concerns about potential conflict of interests (COIs) compromising patient organisations' independence and credibility.[5 6 7 8 9 10]

Both donors[11] and recipients[4] assert that any COIs can be managed by careful disclosure of funding. On the donor side, since 2012 members of pharmaceutical industry trade groups in European countries,[12] such as the Association of the British Pharmaceutical Industry (ABPI), have disclosed payments to patient organisations annually on each company's website, including their monetary value and purpose.[13] In the UK, the number of companies subscribing to the ABPI Code also includes over sixty non-members of the ABPI; hence, nearly every relevant company is covered.[14] However, the self-regulatory approach to payment disclosure has shortcomings, including absent reports, unclear or inadequate payment descriptions, and unstandardised reporting.[1 15] Likewise, on the recipient side, disclosures published on patient organisations' websites have been criticised as incomplete or uninformative.[16 17 18 19 20] In the UK, a possibly more reliable, yet rarely examined,[15 21] source of disclosures are mandatory annual accounts of patient organisations registered as charities with an annual income over £25,000. These accounts serve a dual-purpose of detailing the charity's activities and ensuring financial transparency.

Notwithstanding the shortcomings of each side's disclosures, and the potentially complementary information they provide, donor and recipient disclosures are rarely compared. One exception is a recent UK study which identified companies not disclosing payments to patient organisations being mentioned in their annual accounts as donors.[15] Another UK study found discrepancies between annual accounts of some patient organisations contributing to health technology assessment in England and drug company payment disclosures.[21] Similarly, discrepancies were found between sponsorships reported on patient organisation and drug company websites in Italy.[19] More broadly, cross-interrogation of different data sources has revealed undisclosed industry ties among treatment guideline panellists,[22 23 24] clinical trialists, authors of medical journal articles,[25 26 27 28 29] and some clinical commissioning groups[30] and NHS trusts in England.[31]

We examine the underreporting of payments to UK patient organisations from 2012 to 2016 by comparing payment disclosures made by 87 companies to 425 patient organisations with the annual accounts of the same set of patient organisations. Specifically, by considering the extent to which the industry and patient organisation disclosures differ regarding who provided and received funding, and how much was paid and received, we examine the reliability of the two disclosures systems said to neutralise concerns about COIs.

For peer review only - http://bmjopen.bmj.com/site/about/guidelines.xhtml

Methods

Data sources and extraction

Absent of a complete list of non-ABPI member companies that subscribe to the ABPI Code, our sample of drug companies comprised all 108 participants of Disclosure UK in 2015, including 53 ABPI members and 55 non-members (web supplement 1). Disclosure UK is a self-regulatory initiative run by the ABPI, covering payments to healthcare organisations[32] and professionals,[33] but because its participants subscribe to the ABPI Code, they should also separately disclose, on their websites, payments to patient organisations once a year.[1 15]

In June 2017 and January 2018, ER searched the websites of the 108 companies, identifying 220 disclosure reports published between 2012 and 2016. ER downloaded them and extracted payment data into a single Excel spreadsheet, converting all payment values to 2016 sterling using inflation data from the Office for National Statistics.[34] There were 66 (61.1%) companies disclosing payments in at least one year during this period. Following the exclusion of ineligible payments based on their timing or recipient characteristics, we established that 64 companies reported 4,572 payments, worth £57,305,289.2 to 489 UK patient organisations (web supplement 1).

In September 2017 and July 2018, ER searched for these patient organisations on the websites of the UK charity regulators – the Charity Commission for England and Wales, the Scottish Charity Regulator and the Northern Irish Charity Regulator – to which registered charities must submit their annual accounts. We considered accounts from financial years ending in 2012 to 2017 to cover all calendar year data from drug company reports. We found that 425 (86.9%) patient organisations drug companies listed as recipients published their annual accounts at least once with at least one charity regulator, with the total number of annual accounts of 1,428. In industry data, 63 (98.4%) companies reported 4,316 (94.4%) payments, worth £54,071,454.21 (94.4%) to these patient organisations.

ER read all annual accounts and extracted any information pertaining to drug company payments to an Excel spreadsheet. As with industry data, if several values were mentioned in a single payment description they were considered as separate payments. As patient organisation yearly accounts lack specific monthly end date,[21] we turned those covering months up to June into the previous calendar year, and the rest into the current calendar year. The 425 patient organisations registered as charities reported 4,372 payments, worth £58,668,293.6, from 167 donors. Following exclusion criteria referring to donor and recipient characteristics as well as the conversion of financial into calendar years (web supplement 2), we established that 200 (47.1%) organisations reported 1,661 (38.0%) payments, worth £33,037,955.8 (56.3%), from 84 (47.1%) drug companies. Notably, we excluded 2,014 payments, worth £1,992,147.3, made by 61 companies found in patient organisation data but missing from the original sample of Disclosure UK participants.

One researcher, ER, collected drug company disclosure reports and patient organisation annual acccounts. Drug company and charity regulator websites are well-structured and ER searched the websites at two points in time, with the second phase of data collection being used to check the accuracy of the initial one. Therefore, we are confident that no relevant

For peer review only - http://bmjopen.bmj.com/site/about/guidelines.xhtml

documents were missed. When extracting payment data from drug company and patient organisation documents into Excel spreadsheets, ER checked twice that each payment was transferred correctly. As these documents follow a similar format we deemed it sufficient for another researcher, PO, to check 20% randomly selected drug company payment disclosure reports and patient organisation annual accounts with no discrepancies found.

The ethical implications of this study article were approved via a peer ethics review process at the Department of Social and Policy Sciences, University of Bath in April 2016. This study did not require a full ethical approval as it relied on publicly available data aggregated at the organisational level.

Analysis

MC and PO converted the data into donor-recipient matrices and analysed it descriptively in Excel. We examined the overall pattern of the number and value of payments in each dataset and its changes over time.

Outcome measures

Absent an exhaustive list of payments, enabling a direct comparison of industry and patient organisation data, we studied underreporting with five complementary measures. Our calculations can be verified by consulting web supplements 3-20 signposted throughout the Results. The web supplements are accessible via the University of Bath Research Data Archive in the Excel format.[35]

First, we compared the overall volume of payments reported in industry and patient organisation data, including payments lacking specific values.

Second, we examined the absolute and relative differences in payments reported in relation to the same donors and recipients in industry and patient organisation data. We calculated the absolute difference as the difference between the number/value of payments reported in the two datasets; the relative difference was the absolute difference divided by the number/value of payments in the dataset with the higher number/value of payments. To compare larger and smaller donors and recipients we considered absolute and relative differences at different thresholds of the overall number (i.e. ≥1, >1, >10, >100 in at least one dataset) and value of payments (i.e. >£0, >£10,000, >£100,000 in at least one dataset).

Third, focusing on the highest relative differences, in which donors or recipients were present in one but absent from the other dataset, we calculated the number and value of payments related to donors and recipients missing from each dataset.

Fourth, we compared the extent to which industry and patient organisation data conveyed the same pattern of connections between donors and recipients. Specifically, we analysed the overlap of ties (or connections) between donors and recipients – established by the presence of payments – in each dataset.

For peer review only - http://bmjopen.bmj.com/site/about/guidelines.xhtml

Finally, building on the analysis of donor-industry ties at the level of the two datasets, we moved to the donor and recipient level by examining the extent of overlap between recipient and donor lists in patient organisation and industry data, respectively.

Patient and public involvement

Neither patient groups nor the public were involved in this study. We plan to disseminate key findings in an accessible format using a blog post.

Results

The Results follow the measures of underrporting of payments outlined in the Methods. We summarise relationships between these measures in Figure 1.

[Figure 1]

Comparison of payment patterns in industry and patient organisation data

Although 63 donors reported payments, 84 were mentioned by recipients. In industry data, donors disclosed payments to 425 recipients but only 200 (47.1%) of those reported payments during the study period (Table 1).

The number of payments disclosed by donors was 259.8% greater than those disclosed by recipients (Table 1). Each side was disclosing more payments over time, but the increase from 2012 to 2016 was greater in industry data (141.2% v 115.8%, respectively). In industry data, 4,235 (98.1%) payments reported by 62 (98.4%) donors to 416 (97.9%) recipients had values greater than 0. In patient organisation data, only 772 (46.5%) payments reported by 121 (60.5%) recipients from 62 (73.8%) donors had values greater than 0. The value of payments disclosed by donors was 163.7% higher than those disclosed by recipients. Over the five-year period the value of payments reported by donors increased by 257.6%, while those by recipients – by 434.7%.

For peer review only

Table 1 Patterns in payments reported by donors and recipients*

	All years	2012	2013	2014	2015	2016
**Calculations based on the number of payments**						
Number of payments - industry data	4316	769	706	821	934	1086
Number of payments - patient organisation data	1661	291	326	346	361	337
Number of drug companies with at least one payment - industry data	63	30	38	45	51	44
Number of drug companies with at least one payment - patient organisation data	84	56	65	65	69	65
Number of patient organisations with at least one payment - industry data	425	197	198	225	244	244
Number of patient organisations with at least one payment - patient organisation data	200	103	102	116	126	117
**Calculations based on the value of payments**						
Number of payments with value >£0 - industry data	4235	748	682	813	915	1077
Number of payments with value >£0 - patient organisation data	772	144	148	144	173	163
Value of payments - industry data (2016 £)	54,071,454.2	7,797,600.4	6,189,152.7	8,583,101.6	11,415,293.2	20,086,306.3
Value of payments - patient organisation data (2016 £)	33,037,955.8	3,459,605.6	4,414,328.4	5,301,728.3	4,825,091.2	15,037,202.3
Number of drug companies with at least one payment with the value >£0 - industry data	62	30	38	45	50	44
Number of drug companies with at least one payment with the value >£0 - patient organisation data	62	42	44	42	46	44
Number of patient organisations with at least one payment with the value >£0 - industry data	416	197	193	225	237	244
Number of patient organisations with at least one payment with the value >£0 - patient organisation data	121	57	51	58	72	57

For peer review only - http://bmjopen.bmj.com/site/about/guidelines.xhtml

* We used the following values of the Consumer Price Index obtained from Office for National Statistics to adjust payment values for inflation: 2012=96, 2013=98.2, 2014=99.6, 2015=100, 2016=101.

For peer review only

Payments reported in relation to the same donors and recipients in industry and patient organisation data

Having considered the overall pattern of payments, we now compare those reported in relation to the same donors and recipients in each dataset.

Donors

Of the 87 donors identified in industry and patient organisation data, three (3.4%) had matching payment numbers, and 49 (56.3%) had more payments in patient organisation data (Table 2; web supplement 3). In relative terms, only 21 (24.1%) donors had a difference in the number of payments less than 50%, and 27 (31.0%) had a difference of 100%, equivalent to only either donors or recipients reporting payments.

In both datasets, 74 (85.1%) donors had payment values greater than 0 (web supplement 3). Of those, 48 (64.9%) had a higher payment value in industry data and the remaining ones – in patient organisation data. Only 14 (18.9%) donors with payment values greater than 0 had a relative difference lower than 50%, while 24 (32.4%) donors had a relative difference of 100%.

Overall, donors with a high overall number and value of payments in either dataset typically had high absolute differences (web supplement 3). Contrastingly, donors with a small number or value of payments usually had higher relative differences, that is, high relative differences often resulted from few payments or payments of small value in one dataset but none in the other.

Over time, there was little improvement in the shares of donors with matching numbers of payments (from 1.6% in 2012 to 5.5% in 2016); the share of donors with matching payment values showed no improvement (0%). However, the shares of those with relative differences in payment numbers or values lower than 50% increased (from 11.5 to 17.4%; and from 7.7% to 25.5%, respectively) (web supplement 4).

For peer review only

Table 2 Donors – absolute and relative differences in the number and value of payments

	All years	%
**Calculations based on the number of payments - absolute differences**		
Number of drug companies with at least one payment in at least one dataset - industry and patient organisation data	87	
Number of drug companies with exact match in both datasets	3	3.4%
Number of drug companies with more payments in industry data	49	56.3%
Number of drug companies with more payments in patient organisation data	35	40.2%
Highest absolute difference between patient organisation and industry data - number of payments higher in industry data	757	
Highest absolute difference between patient organisation and industry data - number of payments higher in patient organisation data	53	
**Calculations based on the number of payments - relative differences**		
Number of drug companies with relative difference <10%	5	5.7%
Number of drug companies with relative difference <20%	9	10.3%
Number of drug companies with relative difference <50%	21	24.1%
Number of drug companies with relative difference = 100%	27	31.0%
**Calculations based on the value of payments - absolute differences**		
Number of drug companies with at least one payment with value >£0 in at least one dataset	74	100%
Number of drug companies with exact match in both datasets	0	0.0%
Number of drug companies with higher payment value in industry data	48	64.9%
Number of drug companies with higher payment value in patient organisation data	26	35.1%

For peer review only

Highest absolute difference between patient organisation and industry data - number of payments higher in industry data (2016 £)	6,406,351.2	
Highest absolute difference between patient organisation and industry data - number of payments higher in patient organisation data (2016 £)	2,960,716.0	
**Calculations based on the value of payments - relative differences**		
Number of drug companies with relative difference <10%	3	4.1%
Number of drug companies with relative difference <20%	9	12.2%
Number of drug companies with relative difference <50%	14	18.9%
Number of drug companies with relative difference = 100%	24	32.4%

For peer review only

Recipients

Of the 425 recipients identified in industry data, 33 (7.8%) had matching payments numbers in both datasets, while 335 (78.8%) had a greater number in industry data (Table 3, web supplement 5). In relative terms, 225 (52.9%) recipients had payments reported only in industry data (the relative difference of 100%), while 81 (19.1%) had a relative difference lower than 50%.

Of 425 recipients, 416 (97.9%) had payments with a value greater than 0 in at least one dataset (web supplement 5). Of those, 356 (85.6%) had a higher value of payments in industry data, and 8 (1.9%) had matching payment values. In relative terms, 295 (70.9%) recipients had payments reported exclusively in industry data, and 73 (17.5%) recipients had a relative difference lower than 50%.

Overall, recipients with a high overall number and value of payments usually had high absolute differences (web supplement 5). Relative differences were lower for recipients with higher overall number and value of payments.

The shares of recipients with matching numbers and values of payments showed no improvement over time, but the shares of those with relative differences lower than 50% increased (from 13.7% to 17.3% and from 9.7% to 14.5%, respectively) (web supplement 6).

For peer review only

Table 3 – Recipients – absolute and relative differences in the number and value of payments

	All years	%
**Calculations based on the number of payments - absolute differences**		
Number of patient organisations with at least one payment in at least one dataset - industry and patient organisation data	425	
Number of patient organisations with exact match in both datasets	33	7.8%
Number of patient organisations with more payments in industry data	335	78.8%
Number of patient organisations with more payments in patient organisation data	57	13.4%
Highest absolute difference between patient organisation and industry data - number of payments higher in industry data	199	
Highest absolute difference between patient organisation and industry data - number of payments higher in patient organisation data	33	
**Calculations based on the number of payments - relative differences**		
Number of patient organisations with relative difference <10%	35	8.2%
Number of patient organisations with relative difference <20%	42	9.9%
Number of patient organisations with relative difference <50%	81	19.1%
Number of patient organisations with relative difference = 100%	225	52.9%
**Calculations based on the value of payments - absolute differences**		
Number of patient organisations with at least one payment with value >£0 in at least one dataset	416	100%
Number of patient organisations with exact match in both datasets	8	1.9%
Number of patient organisations with higher payment value in industry data	356	85.6%
Number of patient organisations with higher payment value in patient organisation data	52	12.5%

For peer review only

For peer review only - http://bmjopen.bmj.com/site/about/guidelines.xhtml

Highest absolute difference between patient organisation and industry data - number of payments higher in industry data (2016 £)	6,718,576.6
Highest absolute difference between patient organisation and industry data - number of payments higher in patient organisation data (2016 £)	6,493,237.1

Calculations based on the value of payments - relative differences

Number of patient organisations with relative difference <10%	21	5.0%
Number of patient organisations with relative difference <20%	34	8.2%
Number of patient organisations with relative difference <50%	73	17.5%
Number of patient organisations with relative difference = 100%	295	70.9%

For peer review only

For peer review only - http://bmjopen.bmj.com/site/about/guidelines.xhtml

Missing payments

After analysing the pattern of absolute and relative differences we now focus on donors and recipients with the relative difference of 100%, that is, those with payments reported only in either industry or patient organisation data.

Industry data

There were 24 donors, representing 38.1% of those in industry data, which seemed not to comply with the ABPI Code[13] as their payments were reported exclusively in patient organisation data over the entire period of observation (web supplement 7, 8, 9). The number and value of payments associated with these donors (in patient organisation data) were 128 and £1,610,321.1, equivalent to 3% of the number and value of payments in industry data. The annual shares of missing donors and their payments were considerably higher as some companies did not report payments only in some years. The annual shares of missing payments were decreasing over time.

Patient organisation data

There were 3 donors, constituting 3.6% of those in patient organisation data, whose payments were only reported in industry data (web supplement 10, 11, 12). The number and value of payments associated with these donors (in industry data) were 9 and £92.208.2, constituting 0.5% and 0.3% of the number and value of payments in patient organisation data. As with industry data, the annual shares of missing donors and their payments were higher as some donors were not reported in patient organisation data only in some years. The annual shares of missing payments were decreasing during the study period.

There were 225 recipients missing from patient organisation data, representing 112.5% of recipients in this dataset (web supplement 13, 14, 15). The number and value of missing payments were 1,472 and £14,023,475.41 (88.6% and 42.4% of the respective figures in patient organisation data). The yearly share of missing recipients and their payments increased, while the yearly share of missing payment values decreased.

Agreement between donor-recipient ties reported by industry and patient organisations

After describing the underreporting as a donor/recipient characteristic we now consider it by examining the distribution of ties between donors and recipients in each dataset

There were 1101 ties between donors and recipients formed by at least one payment in industry data and 626 in patient organisation data (Web Supplement 16). In 428 instances both datasets agreed about the donors and recipients, representing 38.9% and 68.4% of ties in each dataset, respectively. Of these, in 87 (20.3%) instances both donors and recipients reported the same number of payments. The number of payments constituting those matching ties was 162, equivalent to 3.8% and 9.8% of the total in industry and patient organisation data, respectively.

For peer review only - http://bmjopen.bmj.com/site/about/guidelines.xhtml

There were 1,088 ties involving payments with a value greater than zero in industry data and 326 in patient organisation data. In 34 instances, both datasets agreed about donors and recipients (3.1% and 10.4% of ties in each dataset). Of these, in 20 instances (58.8%) instances both sides provided matching payment values. The ties with matching values were worth £325,108.4, representing 0.6% and 1.0.% of the value of payments in industry and patient organisation data, respectively.

The proportion of shared ties remained stable across time (web supplement 16).

Overlap between recipient and donor lists in the two datasets

Having compared the distribution of ties in each dataset, we now consider ties as a characteristic of donors and recipients. Specifically, we analyse underreporting by comparing donor and recipient lists in each dataset against the other dataset.

Figure 2 Overlap between recipient and donor lists

Overlap between recipient lists and patient organisation data

Of 63 donors reporting at least one payment in the industry dataset, only 3 (4.8%) had all their recipients mentioning them as donors, the situation representing the full overlap between recipient lists and patient organisation data (Figure 2A). Conversely, the recipient lists of 6 (9.5%) donors had zero overlap with patient organisation data. The recipient lists of the remaining donors were situated between the two extremes, with 40 (63.5%) donors having less than 50% overlap, meaning that only half of the recipients were reflected in patient organisation data.

Considering the recipient lists calculated using the value of payments (62 donors reporting payment values greater than zero), the full and zero overlap occurred for 2 (3.2%) and 17 (27.0%) donors, respectively (Figure 2B). Overall, 56 (90.3%) donors had less than 50% overlap with patient organisation data.

Overlap between donors lists and industry data

Of the 200 recipients in patient organisation data, 102 (51.0%) had all donors mentioning them as recipients, which represented the full overlap between donor lists and industry data (Figure 2C). Contrastingly, the donor lists of 14 (7.4%) recipients had no overlap with industry data. Overall, 32 (16.0%) recipients had less than 50% overlap with industry data.

Based on the value of payments (121 recipients reporting payment values greater than zero), the full and zero overlap occurred for 71 (58.7%) and 12 (9.9%) recipients, respectively (Figure 2D). There were 18 (14.9%) donors with less than 50% overlap with industry data.

Over time, there was little improvement in the share of patient organisations reporting funding from drug companies listing them as recipients (web supplement 17 and 18). On the other hand, donors were reporting an increasing share of patient organisations mentioning their funding (web supplement 19 and 20).

For peer review only - http://bmjopen.bmj.com/site/about/guidelines.xhtml

Discussion

Principal findings

Our study confirms that earlier concerns about the underreporting of payments in relation to specific drug companies[15] and charities[36 37] in the UK were not isolated instances. It demonstrates that neither the industry self-regulatory system for disclosure of payments to patient organisations[1] nor a state-run system built for broader financial transparency purposes prevented a high extent of underreporting. Importantly, the underreporting occurred despite the media and research salience of the transparency of financial relationships between the industry and patient organisations.[19 38 39 40]

Although some yearly variation might be expected given differences between when a payment was made and when it was received or spent, the overall large and seemingly increasing discrepancies between industry and patient organisation records are concerning. The number and value of payments disclosed by drug companies were likely to be higher because the ABPI Code has a formal definition of a payment and stipulates that payment values should be recorded whenever possible.[13] Charity regulators lack similar specific requirements.[41] Further, some patient organisations may redistribute money to other collaborators and therefore they may report smaller amounts than donating drug companies.[42] Nevertheless, any differences between donor and recipient records should be minimised by the requirement for a written contract between the parties, introduced by the ABPI Code, implying a shared understanding of how much is paid, to whom, and how.[13]

What also indicates underreporting is that few companies and patient organisations had matching – or even broadly similar – records in the two datasets, with many having differences exceeding hundreds of payments or millions of pounds. Both drug companies and patient organisations had a larger number of payments in the other dataset, but for both sides the value of payments was greater in industry data. The contrast between the datasets was higher for patient organisations, with a majority having a greater number and value of payments reported in industry data. This pattern suggests inadequacies of the existing charity regulator governance of the reporting of corporate payments. It further corresponds with the results of a recent journalistic investigation into the underreporting of payments by a major charity,[36 37] prompting a rebuke from a charity regulator.[43]

We also unearthed unreported payments associated with companies missing from either dataset. The underreporting of payments is further indicated by the fact that donor-recipient ties reported in both datasets were in minority. This was particularly the case for ties involving matching payment numbers or values. The extent of the overlap between donor and recipient lists reported in the two datasets was similarly limited. Therefore, donors and recipients rarely disclosed payments reported by the other side.

As drug companies from our sample signed the ABPI Code, missing payments indicate that they either did not meet the obligation to disclose or removed their online disclosure reports. We could not find any evidence of specific obligations for UK charities to name funders in their accounts.[41] Their absence is motivated by concerns that “the loss of donor anonymity would result in a decrease in voluntary income reported by charities. There were

For peer review only

also concerns about the practical implications and about how much interest 'general users' of charity accounts would have in this disclosure."[44] Nevertheless, any unreported payments could be misleading for patient organisation members, supporters, expert bodies relying on patient testimonies,[21] policymakers, and the public.[36 43] Unreported payments may indicate a culture of corporate manipulation of patient organisations.[45 46] Indeed, an oft-used argument for increasing transparency of payments is that "sunshine" mitigates against misbehaviour and undue influence.[47] Crucially, despite challenges in achieving financial sustainability,[48] some patient organisations do not accept industry funding.[49 50]

Finally, underreporting should be minimised by codes and reporting standards seeking to ensure faithful disclosure. The ABPI's self-regulatory authority, the PMCPA, is tasked with ensuring compliance under the ABPI Code, including through guidance and training, occasional active monitoring, and with the possibility to sanction companies that breach their obligations.[51 52] Likewise, the UK Charity regulators monitor a selection of annual accounts annually and, in instancies of inaccurate reporting, will contact the charity directly to provide advice or request the accounts be resubmitted.[53 54 55]

More broadly, a similarly high extent of underreporting was found in Italy, with less than a third of patient organisations identified as funding recipients disclosing industry funding.[19] High levels of underreporting have also been identified internationally in relation to other recipients of industry payments, including authors of clinical practice guidelines [23 24] and scientific publications.[26 27 29]

Strengths and limitations

This is the first study examining the underreporting of payments to patient organisations using large samples of donors and recipients over a period of time. It systematically compares the underreporting by the two sides using five complementary measures. These measures account for the varying nature of different types of payment data and therefore can be replicated elsewhere.

Our study has several limitations. First, while Disclosure UK covers a vast majority of the UK pharmaceutical industry,[14] our sample was not exhaustive, as demonstrated by additional companies found in patient organisation accounts. Second, excluding companies outside of our sample is likely to have underestimated the underreporting but reach data saturation would require several further rounds of data scrapping . Third, we identified patient organisations using drug company disclosure reports, but sampling starting from patient organisations could have produced different results. Fourth, patient organisations registered with charity regulators but with yearly income lower than £25,000 are not required to submit annual accounts. Therefore, we might have excluded, entirely or in specific years, some of the patient organisations identified as recipients of industry funding, if their yearly incomes were below that threshold. Fifth, some patient organisations were excluded due to the conversion of financial years into calendar years, which might have increased discrepancies with drug company disclosure reports. Sixth, some companies might have reported payments from 2016 in subsequent years, but the extent of delayed reporting is likely to have been minimal,[1] and there was no delayed reporting in patient organisation annual accounts. Seventh, we only considered annual accounts submitted to charity regulators, while funders might have also (or instead) been disclosed on the charity's

For peer review only - http://bmjopen.bmj.com/site/about/guidelines.xhtml

website. However, website disclosures are likely to undergo frequent changes, and often lack transparency.[16 17 18 19 20] Eight, evaluating the extent of underreporting precisely is impossible because no definitive list of payments exists; consequently, there could be payments undisclosed by both sides. Finally, considering payment descriptions could reveal further discrepancies, including different payment goals reported by donors and recipients; it could also identify payments made via third parties (e.g. public relations companies[43]) or benefitting patient organisations indirectly.[15]

Conclusions and policy implications

Although the full scale of underreporting of industry payments to patient organisations remains unknown, it concerns both donors and recipients, and involves a considerable number and value of payments. Our findings put a question mark over the key claim that – at least in their current form – publicly available payment disclosures effectively address concerns about COIs resulting from industry payments.

We provide evidence for developing easily achievable improvements in the reporting of payments by both donors and recipients. Consistent with the ABPI Code, the ABPI's self-regulatory authority, the PMCPA, should investigate any instances of missing payments among the companies that have ratified the Code (data never published or prematurely removed from the public domain), and, if appropriate, penalise companies concerned in accordance with its mandate and available financial (so-called administrative charges) and non-financial (mainly naming and shaming) sanctions.[51 52] Separately, drug companies should improve data presentation following earlier recommendations,[15] especially standardisation of reporting and elimination of payments with no assigned values. Companies should store data for at least as long as is required by the Charity Commission for England and Wales, that is five years. Reduction in underreporting could also be achieved if the ABPI started to publish yearly reports summarising payments to patient organisations disclosed by companies, as the ABPI currently does with payments to healthcare professionals and organisations in the Disclosure UK database.[33 32] Indeed, at least one European country – Sweden – has an industry-run, centralised disclosure database of payments to patient organisations,[56] and there is no reason why the ABPI should not have the same.

Given the shortcomings of the industry self-regulatory system, charity regulators should introduce tailored solutions related to reporting corporate funding in annual accounts, including a standardised template comprising a short payment description (including payment form and goal), its value, and donor name. Patient organisation websites should report this information separately or include clear signposting to the annual accounts. A key step in refining these solutions would involve in-depth exploration of perspectives of patient organisations. However, ultimately, only a single state-run permanent database integrating payments reported by drug companies and patient organisations could eliminate underreporting.

For peer review only

References

1. Ozieranski P, Rickard E, Mulinari, Shai. Exposing drug industry funding of UK patient organisations. *BMJ* 2019;365:l1806. doi: 10.1136/bmj.l1806
2. Rose SL, Highland J, Karafa MT, et al. Patient advocacy organizations, industry funding, and conflicts of interest. *JAMA internal medicine* 2017;177(3):344-50.
3. Taylor J, Denegri S. Industry links with patient organisations. *BMJ* 2017
4. Kent A. Should patient groups accept money from drug companies? Yes. *BMJ* 2007;334(7600):934. doi: 10.1136/bmj.39185.461968.AD
5. Hemminki E, Toiviainen HK, Vuorenkoski L. Co-operation between patient organisations and the drug industry in Finland. *Social science & medicine* 2010;70(8):1171-75.
6. McCoy MS, Carniol M, Chockley K, et al. Conflicts of interest for patient-advocacy organizations. *New England Journal of Medicine* 2017;376(9):880-85.
7. Fabbri A, Swandari S, Lau E, et al. Pharmaceutical Industry Funding of Health Consumer Groups in Australia: A Cross-sectional Analysis. *International Journal of Health Services* 2019:0020731418823376.
8. Lexchin J. Association between commercial funding of Canadian patient groups and their views about funding of medicines: An observational study. *PLOS ONE* 2019;14(2):e0212399. doi: 10.1371/journal.pone.0212399
9. Kang S-Y, Bai G, Karas L, et al. Pharmaceutical Industry Support of US Patient Advocacy Organizations: An International Context. *American journal of public health* 2019;109(4):559-61.
10. Fabbri A, Parker L, Colombo C, et al. Industry funding of patient and health consumer organisations: systematic review with meta-analysis. *BMJ* 2020;368:l6925. doi: 10.1136/bmj.l6925
11. Pearcy J. ABPI guidance on drug companies and patient organisations. *BMJ* 2019;364:l740. doi: 10.1136/bmj.l740
12. EFPIA. EFPIA Code of Practice on relationships between the pharmaceutical industry and Patient Organisations. 2011
13. ABPI. ABPI Code of Practice for the Pharmaceutical Industry. 2016
14. PMCPA. Annual report 2016. 2016
15. Rickard E, Ozieranski P, Mulinari S. Evaluating the transparency of pharmaceutical company disclosure of payments to patient organisations in the UK. *Health Policy* 2019
16. Mosconi P. Industry funding of patients' support groups: Declaration of competing interests is rare in Italian breast cancer associations. *BMJ: British Medical Journal* 2003;327(7410):344.
17. Ball DE, Tisocki K, Herxheimer A. Advertising and disclosure of funding on patient organisation websites: a cross-sectional survey. *BMC Public Health* 2006;6(1):201.
18. Rothman SM, Raveis VH, Friedman A, et al. Health advocacy organizations and the pharmaceutical industry: an analysis of disclosure practices. *American Journal of Public Health* 2011;101(4):602-09.
19. Colombo C, Mosconi P, Villani W, et al. Patient organizations' funding from pharmaceutical companies: is disclosure clear, complete and accessible to the public? An Italian survey. *PLoS One* 2012;7(5):e34974.

For peer review only - http://bmjopen.bmj.com/site/about/guidelines.xhtml

20. Lau E, Fabbri A, Mintzes B. How do health consumer organisations in Australia manage pharmaceutical industry sponsorship? A cross-sectional study. *Australian Health Review* 2018
21. Mandeville KL, Barker R, Packham A, et al. Financial interests of patient organisations contributing to technology assessment at England's National Institute for Health and Care Excellence: policy review. *bmj* 2019;364:k5300.
22. Neuman J, Korenstein D, Ross JS, et al. Prevalence of financial conflicts of interest among panel members producing clinical practice guidelines in Canada and United States: cross sectional study. *BMJ* 2011;343:d5621. doi: 10.1136/bmj.d5621
23. Bindslev JBB, Schroll J, Gøtzsche PC, et al. Underreporting of conflicts of interest in clinical practice guidelines: cross sectional study. *BMC Medical Ethics* 2013;14(1):19. doi: 10.1186/1472-6939-14-19
24. Moynihan R, Lai A, Jarvis H, et al. Undisclosed financial ties between guideline writers and pharmaceutical companies: a cross-sectional study across 10 disease categories. *BMJ Open* 2019;9(2):e025864. doi: 10.1136/bmjopen-2018-025864
25. Rasmussen K, Schroll J, Gøtzsche PC, et al. Under-reporting of conflicts of interest among trialists: a cross-sectional study. *J R Soc Med* 2014;108 doi: 10.1177/0141076814557878
26. Olavarria OA, Holihan JL, Cherla D, et al. Comparison of Conflicts of Interest among Published Hernia Researchers Self-Reported with the Centers for Medicare and Medicaid Services Open Payments Database. *Journal of the American College of Surgeons* 2017;224(5):800-04. doi: https://doi.org/10.1016/j.jamcollsurg.2017.01.052
27. Wayant C, Turner E, Meyer C, et al. Financial conflicts of interest among oncologist authors of reports of clinical drug trials. *JAMA oncology* 2018;4(10):1426-28.
28. Kesselheim AS, Wang B, Studdert DM, et al. Conflict of Interest Reporting by Authors Involved in Promotion of Off-Label Drug Use: An Analysis of Journal Disclosures. *PLOS Medicine* 2012;9(8):e1001280. doi: 10.1371/journal.pmed.1001280
29. Cherla DV, Olavarria OA, Holihan JL, et al. Discordance of conflict of interest self-disclosure and the Centers of Medicare and Medicaid Services. *Journal of Surgical Research* 2017;218:18-22.
30. Moberly T. The pharma deals that CCGs fail to declare. *BMJ* 2018;360:j5915. doi: 10.1136/bmj.j5915
31. Moberly T. NHS joint working with industry is out of public sight. *BMJ* 2019;364:l1353. doi: 10.1136/bmj.l1353
32. Ozieranski P, Csanadi M, Rickard E, et al. Analysis of Pharmaceutical Industry Payments to UK Health Care Organizations in 2015. *JAMA Network Open* 2019;2(6):e196253-e53.
33. Mulinari S, Ozieranski P. Disclosure of payments by pharmaceutical companies to healthcare professionals in the UK: analysis of the Association of the British Pharmaceutical Industry's Disclosure UK database, 2015 and 2016 cohorts. *BMJ open* 2018;8(10):e023094.
34. Office for National Statistics. CPIH Index 2019 [Available from: https://www.ons.gov.uk/economy/inflationandpriceindices/timeseries/l522/mm23 accessed 22nd November 2019.

For peer review only

35. Ozieranski P, Csanadi M, Rickard E, et al. Underreporting of drug industry payments to patient organisations in the UK (2012-2016) Bath2020 [Available from: https://doi.org/10.15125/BATH-00784.
36. Gregory A. Watchdog: patient charities must come clean about Big Pharma cash. *The Sunday Times* 2019.
37. Gregory A. Patients' charity Pain UK took cash from Big Pharma. *The Sunday Times* 2019.
38. Vitry A, Löfgren H. 16. Health consumer groups and the pharmaceutical industry: is transparency the answer? *Democratizing Health: Consumer groups in the policy process* 2011:239.
39. Rose SL. Patient advocacy organizations: institutional conflicts of interest, trust, and trustworthiness: SAGE Publications Sage CA: Los Angeles, CA, 2013.
40. Moynihan R, Bero L. Toward a Healthier Patient Voice: More Independence, Less Industry Funding. *JAMA internal medicine* 2017;177(3):350-51.
41. Charity Commission for England and Wales. Charities SORP (FRS 102) Accounting and Reporting by Charities: Statement of Recommended Practice. 2014
42. Ridley SH. Rapid response to: Exposing drug industry funding of UK patient organisations. *BMJ* 2019;365:l1806.
43. Charity Commission for England and Wales. Pain UK: regulatory compliance case conclusions. 2019
44. Jones G. Charities will not have to name donors, says SORP Committee. *Civil Society News* 2017.
45. Kanter GP. Extending the Sunshine Act From Physicians to Patient Advocacy Organizations. *American Journal of Public Health* 2018;108(8):978-79. doi: 10.2105/AJPH.2018.304520
46. Lin DH, Lucas E, Murimi IB, et al. Financial Conflicts of Interest and the Centers for Disease Control and Prevention's 2016 Guideline for Prescribing Opioids for Chronic Pain. *JAMA Internal Medicine* 2017;177(3):427-28. doi: 10.1001/jamainternmed.2016.8471
47. Rosenthal MB, Mello MM. Sunlight as Disinfectant — New Rules on Disclosure of Industry Payments to Physicians. *New England Journal of Medicine* 2013;368(22):2052-54. doi: 10.1056/NEJMp1305090
48. Baggott R, Jones K. The Big Society in an age of austerity: threats and opportunities for Health Consumer and Patients' Organizations in England. *Health Expectations* 2015;18(6):2164-73. doi: 10.1111/hex.12185
49. Parker L, Fabbri A, Grundy Q, et al. "Asset exchange"—interactions between patient groups and pharmaceutical industry: Australian qualitative study. *BMJ* 2019;367:l6694. doi: 10.1136/bmj.l6694
50. Just Treatment. About 2020 [30th March 2020]. Available from: https://justtreatment.org/about.
51. Zetterqvist AV, Merlo J, Mulinari S. Complaints, Complainants, and Rulings Regarding Drug Promotion in the United Kingdom and Sweden 2004–2012: A Quantitative and Qualitative Study of Pharmaceutical Industry Self-Regulation. *PLOS Medicine* 2015;12(2):e1001785. doi: 10.1371/journal.pmed.1001785
52. PMCPA. About us 2020 [30th March 2020]. Available from: https://www.pmcpa.org.uk/about-us/.
53. Charity Commission for England and Wales. Do charity annual reports and accounts meet the reader's needs? Accounts Monitoring Review, 2017.

For peer review only - http://bmjopen.bmj.com/site/about/guidelines.xhtml

54. OSCR. Annual monitoring 2020 [Available from: https://www.oscr.org.uk/managing-a-charity/annual-monitoring/ accessed 30th March 2020.
55. Charity Commission for Northern Ireland. Getting it right. Guidance to assist charities in complying with their legal obligations and explain how the Charity Commission for Northern Ireland identifies and addresses non-compliance, 2019.
56. LIF. Etik: LER 2020 [30th March 2020]. Available from: https://www.lif.se/etik/samarbetsdatabaser/.

For peer review only

Figure 1

The Venn Diagram illustrates the relationships between the mesures of payment underreporting employed in this paper by showing the overlap between the industry and patient organization datasets. Please note that the size of specific parts of the diagram does not reflect the size of either of the datasets or the overlap between them. All outcome measures were reported for the number and value of payments.

Figure 2

The bars in Figure 2A-1B show the number of donors with varying level of overlap between their recipient lists and patient organisation data based on the number (Figure 2A) and value (Figure 2B) of payments. For example, based on the number of payments there were 13 companies with 20-29.9% recipients also being reported in patient organisation data (Figure 2A).

The bars in Figures 2C-1D show the number of recipients with varying level of overlap between their donor lists and industry data based on the number (Figure 2C) and value (Figure 2D) of payments. For example, based on the number of payments there were 24 recipients with 50-59.9% of donors also being reported in industry data.

For peer review only

Contributors

PO conceived and designed the study, managed, analysed and interpreted the data, as well as drafted the article MC analysed the data and drafted the article. ER collected and managed the data, and drafted the article. SM designed the study, contributed to interpretation, drafted the article

Funding

Shai Mulinari and Piotr Ozieranski's work on this study was supported by the grant "What can be learnt from the new pharmaceutical industry payment disclosures?" awarded by the Swedish Research Council for Health, Working Life and Welfare (FORTE #2016-00875). Emily Rickard's work was funded through the above grant. Marcell Csanadi did not receive funding for this work.

Details of the role of study sponsors

The funding body has played no part in the design or conduct of this study

Statement of independence of researchers from funders

All researchers working on this study are independent from the funding body.

Competing interests

We have read and understood the BMJ Group policy on declaration of interests and declare the following interests:

Shai Mulinari and Piotr Ozieranski's work on the study reported in this paper was supported by the grant "What can be learnt from the new pharmaceutical industry payment disclosures?" awarded by the Swedish Research Council for Health, Working Life and Welfare (FORTE #2016-00875). Emily Rickard's work was funded through the above grant. Marcell Csanadi did not receive funding for this work. We have no other competing interests to declare.

Patient consent

None required.

For peer review only

Ethical approval

No ethical approval was needed. The ethical implications of this study article were approved via a peer ethics review process at the Department of Social and Policy Sciences, University of Bath in April 2016. This study did not require a full ethical approval as it relied on publicly available data aggregated at the organisational level

Data sharing

The authors of this study agree to share data underpinning this study in the form of web supplements 3-20 available from the University of Bath Research Data Archive. The raw data poses no risk to anonymity of individuals as it draws on publicly available reports concerning financial transfers between organisations.

The reference for this dataset is: Ozieranski, P., Csanadi, M., Rickard, E., Mulinari, S., 2020. *Underreporting of drug industry payments to patient organisations in the UK (2012-2016).* Bath: University of Bath Research Data Archive. https://doi.org/10.15125/BATH-00784

Exclusive license

The Corresponding Author has the right to grant on behalf of all authors and does grant on behalf of all authors, a worldwide licence to the Publishers and its licensees in perpetuity, in all forms, formats and media (whether known now or created in the future), to i) publish, reproduce, distribute, display and store the Contribution, ii) translate the Contribution into other languages, create adaptations, reprints, include within collections and create summaries, extracts and/or, abstracts of the Contribution, iii) create any other derivative work(s) based on the Contribution, iv) to exploit all subsidiary rights in the Contribution, v) the inclusion of electronic links from the Contribution to third party material where-ever it may be located; and, vi) licence any third party to do any or all of the above."

Transparency declaration

The guarantor (Piotr Ozieranski) affirms that this manuscript is an honest, accurate, and transparent account of the study being reported; that no important aspects of the study have been omitted; and that any discrepancies from the study as planned (and, if relevant, registered) have been explained.

For peer review only

Figure 1: Relationships between the outcome measures of underreporting of drug company payments in industry and patient organisation data

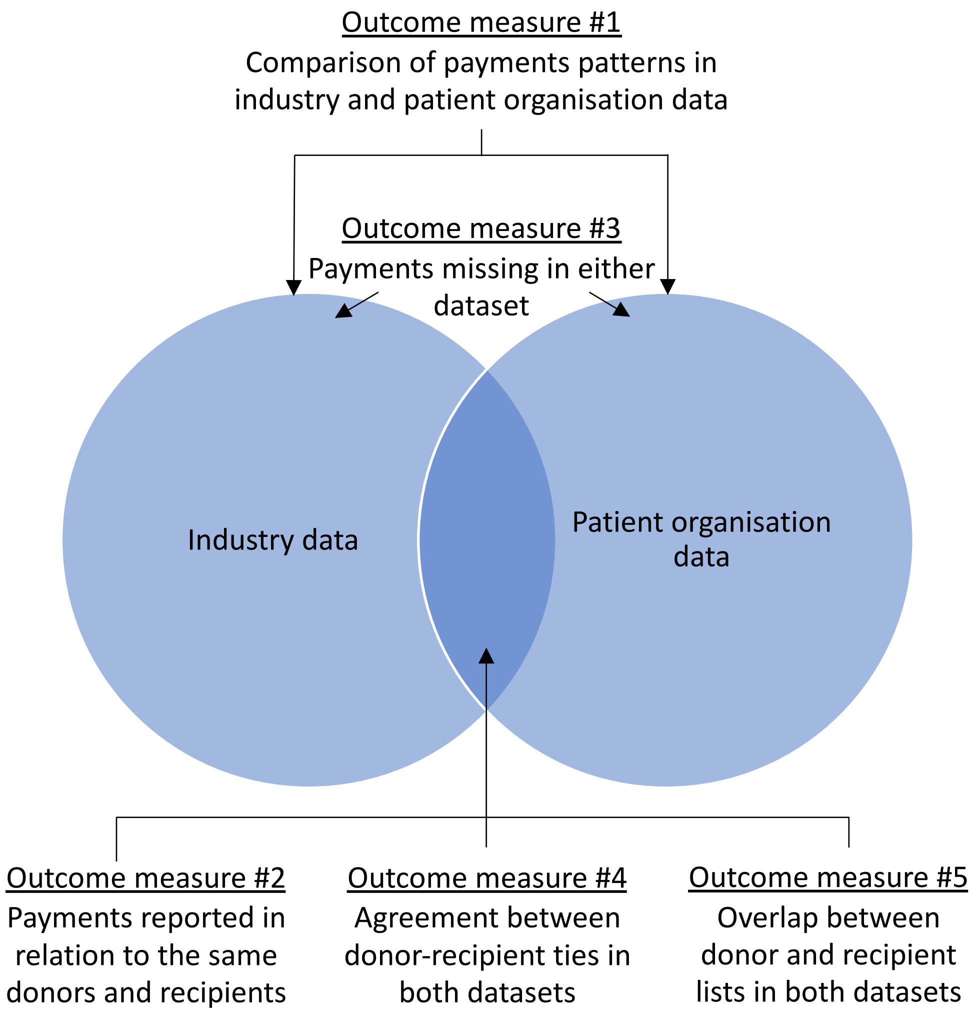

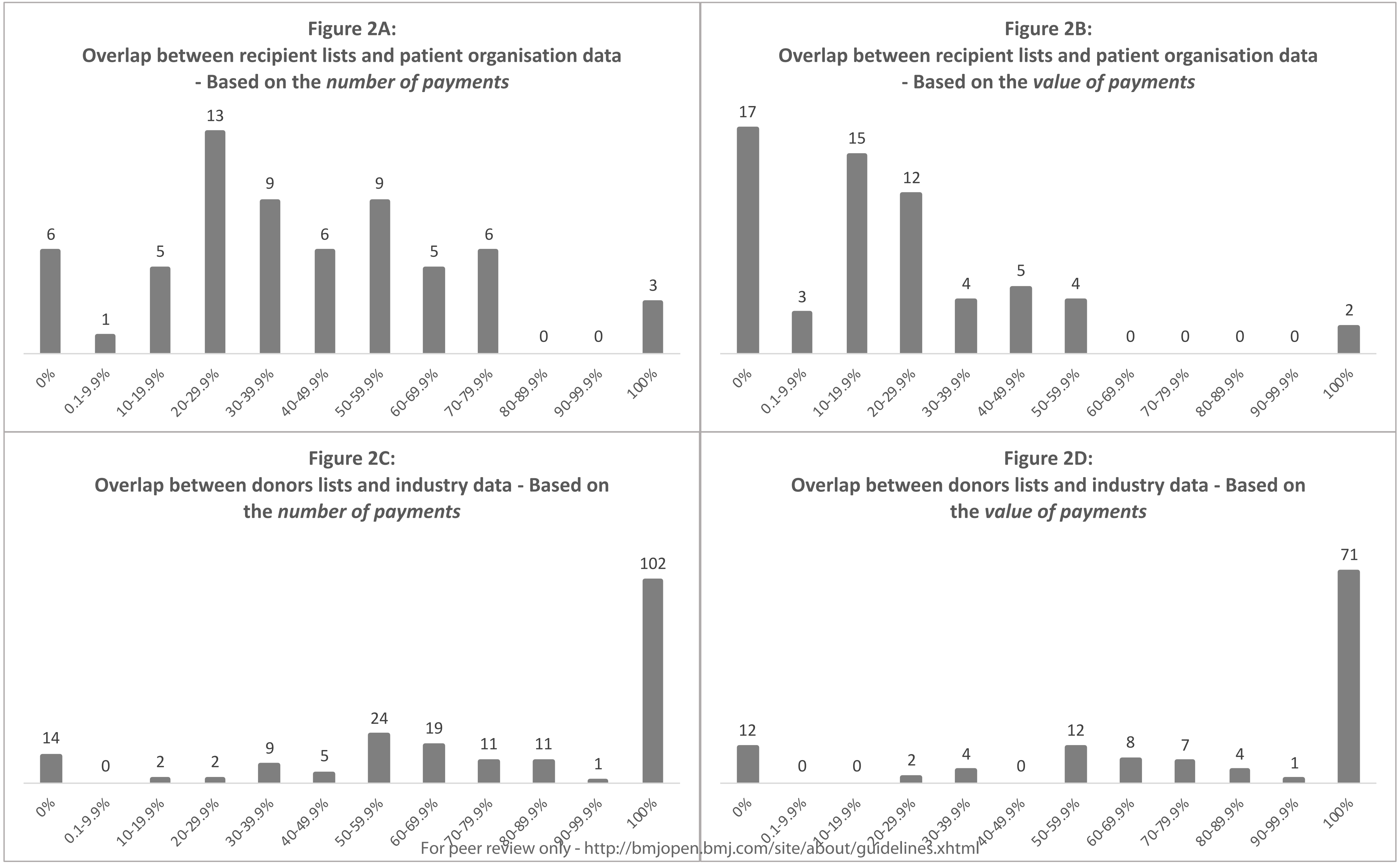
Figure 2A:
Overlap between recipient lists and patient organisation data
- Based on the *number of payments*
6 1 5 13 9 6 9 5 6 0 0 3
0% 0.1-9.9% 10-19.9% 20-29.9% 30-39.9% 40-49.9% 50-59.9% 60-69.9% 70-79.9% 80-89.9% 90-99.9% 100%
Figure 2B:
Overlap between recipient lists and patient organisation data
- Based on the *value of payments*
17 3 15 12 4 5 4 0 0 0 0 2
0% 0.1-9.9% 10-19.9% 20-29.9% 30-39.9% 40-49.9% 50-59.9% 60-69.9% 70-79.9% 80-89.9% 90-99.9% 100%
Figure 2C:
Overlap between donors lists and industry data - Based on
the *number of payments*
14 0 2 2 9 5 24 19 11 11 1 102
0% 0.1-9.9% 10-19.9% 20-29.9% 30-39.9% 40-49.9% 50-59.9% 60-69.9% 70-79.9% 80-89.9% 90-99.9% 100%
Figure 2D:
Overlap between donors lists and industry data - Based on
the *value of payments*
12 0 0 2 4 0 12 8 7 4 1 71
0% 0.1-9.9% 10-19.9% 20-29.9% 30-39.9% 40-49.9% 50-59.9% 60-69.9% 70-79.9% 80-89.9% 90-99.9% 100%

Web supplement 1 – Research process: pharmaceutical industry data

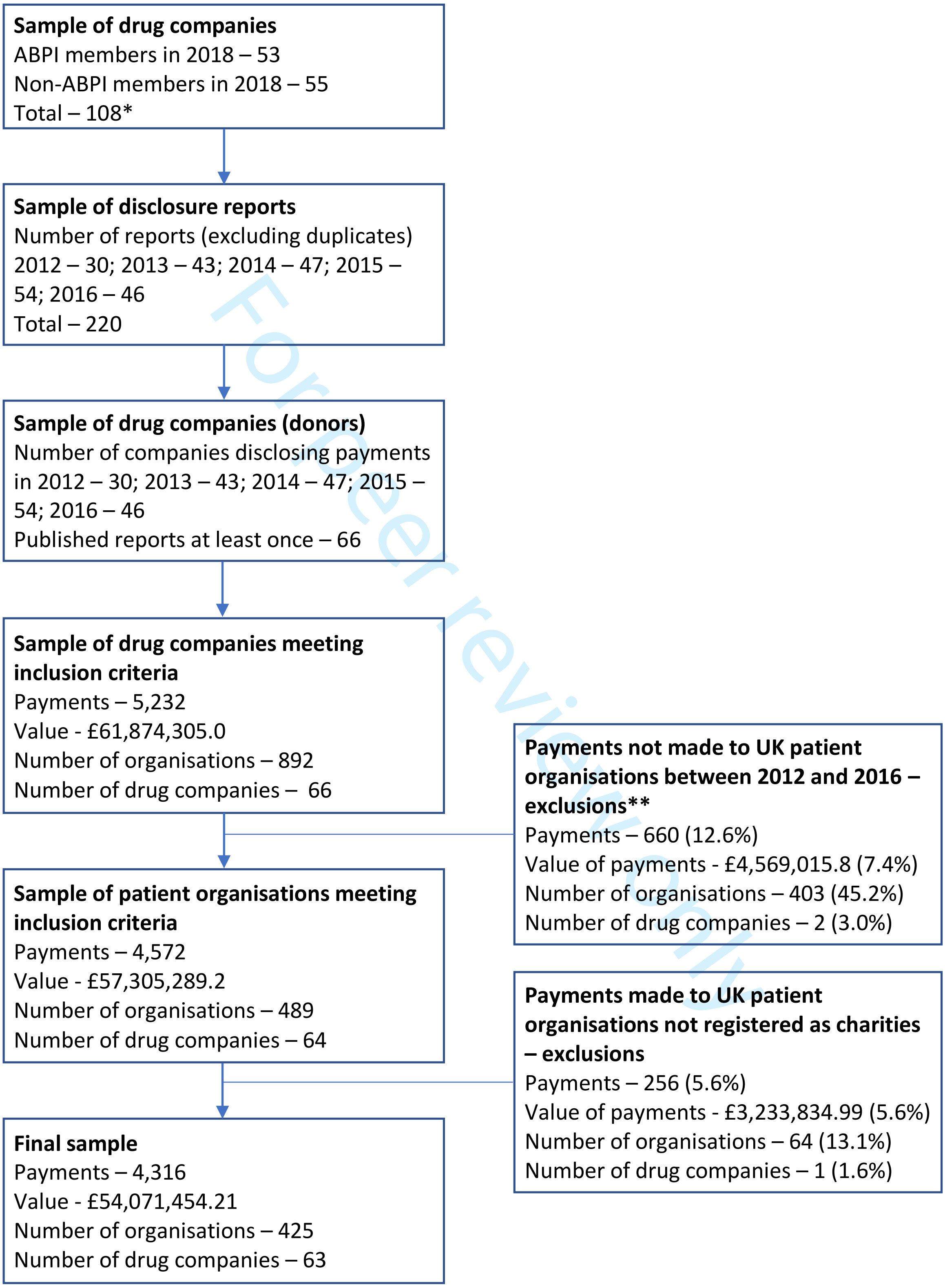

*Complete lists of companies provided in Table 1

**Detailed breakdown provided in Table 2

For peer review only

Table 1 Sample of drug companies – ABPI and non-ABPI members

ABPI member in 2018 – n=53	Non-ABPI member in 2018 – n=55
A. Menarini	Accretio
Abbvie	Actavis
Actelion	Aegerion
Alexion	Aguettant
Alimera Sciences	Alcon
ALK-Abelo	AMCo
Allergan	ApoPharma
Alliance	Astellas
Almirall	Baxalta
Amgen	Baxter
AstraZeneca	Besins
Bausch and Lomb	BGP Products
Bayer	Bio Products Laboratory
Biogen	Biotest
BioMarin	Bracco
Bristol-Myers Squibb	Britannia
Boehringer Ingelheim	CEB Pharma
Celgene	Consilient Health
Chiesi	CSL Behring
Chugai	Dermal
Daiichi Sankyo	Diurnal
Eisai	Ferring
Lilly	Flynn Pharma
Fresenius Medical Care	Fresenius Kabi
Grünenthal	Galen
GSK	Gedeon Richter UK
Ipsen	Genzyme Therapeutics
Janssen	Gilead
LEO Pharma	Guerbet
Lundbeck	Hospira UK
Merck	HRA Pharma
Mitsubishi Tanabe Pharma	Jazz Pharmaceuticals
MSD	Martindale Pharma
Napp Pharmaceuticals	Meda Pharmaceuticals
Norgine	Merz Pharma
Novartis	Mundipharma International
Novo Nordisk	Nicovations
Orion Pharma	Octapharma
Otsuka Pharmaceuticals	Orphan Europe
Pfizer	Profile Pharma

For peer review only

Pharma Mar	PTC Therapeutics
Pierre Fabre Ltd	RB
Quintiles Commercial (Novex)	Rosemont Pharmaceuticals
Roche Products	Sandoz
Sanofi	Sanofi Pasteur
Santen	Sigma Tau Rare Diseases
Servier Laboratories	Sobi
Shionogi Europe	Special Products
Shire	STD
Sunovion Pharmaceuticals	Stirling Anglian
Takeda	Syner-Med
UCB	Teva
Vifor Fresenius Medical Care Renal Pharma	Thea Pharmaceuticals
	Tillotts
	Vifor Pharma Group

Table 2 Breakdown of exclusions - payments not made to UK patient organisations between 2012 and 2016

Reason for exclusion	Number of payments (%)	Value of payments - £ (%)	Number of organisations (%)
Outside period of observation	4 (0.6)	10,039.8 (0.2)	0 (0.0)
Not an organisation	14 (2.1)	94,736.1 (2.1)	0 (0.0)
More than one organisation	17 (2.6)	354,390.0 (7.8)	0 (0.0)
Organisation - nature unclear	57 (8.6)	397,240.6 (8.7)	39 (9.7)
Not UK organisation	54 (8.2)	716,044.0 (15.7)	30 (7.4)
Excluded UK organisations			
Public sector – state organisation	12 (1.8)	18,629.7 (0.4)	7 (1.7)
Public healthcare provider	242 (36.7)	726,401.4 (15.9)	176 (43.7)
Private sector organisation	47 (7.1)	981,886.2 (21.5)	33 (8.2)
University	14 (2.1)	86,670.3 (1.9)	7 (1.7)
Professional organisation (third sector)	143 (21.7)	984,935.1 (21.6)	73 (18.1)
Community interest company or social enterprise - healthcare provider (third sector)	3 (0.5)	2,690.7 (0.1)	3 (0.7)
Healthcare provider (third sector)	20 (3.0)	46,522.0 (1.0)	10 (2.5)
Not focusing directly on health (third sector)	30 (4.5)	148,396.5 (3.2)	24 (6.0)
Community interest company or social enterprise - not focusing directly on health (third sector)	3 (0.5)	433.4 (0.0)	1 (0.2)
Total	660 (100)	4,569,015.8	403 (100)

For peer review only

Web supplement 2 Research process: patient organisation data

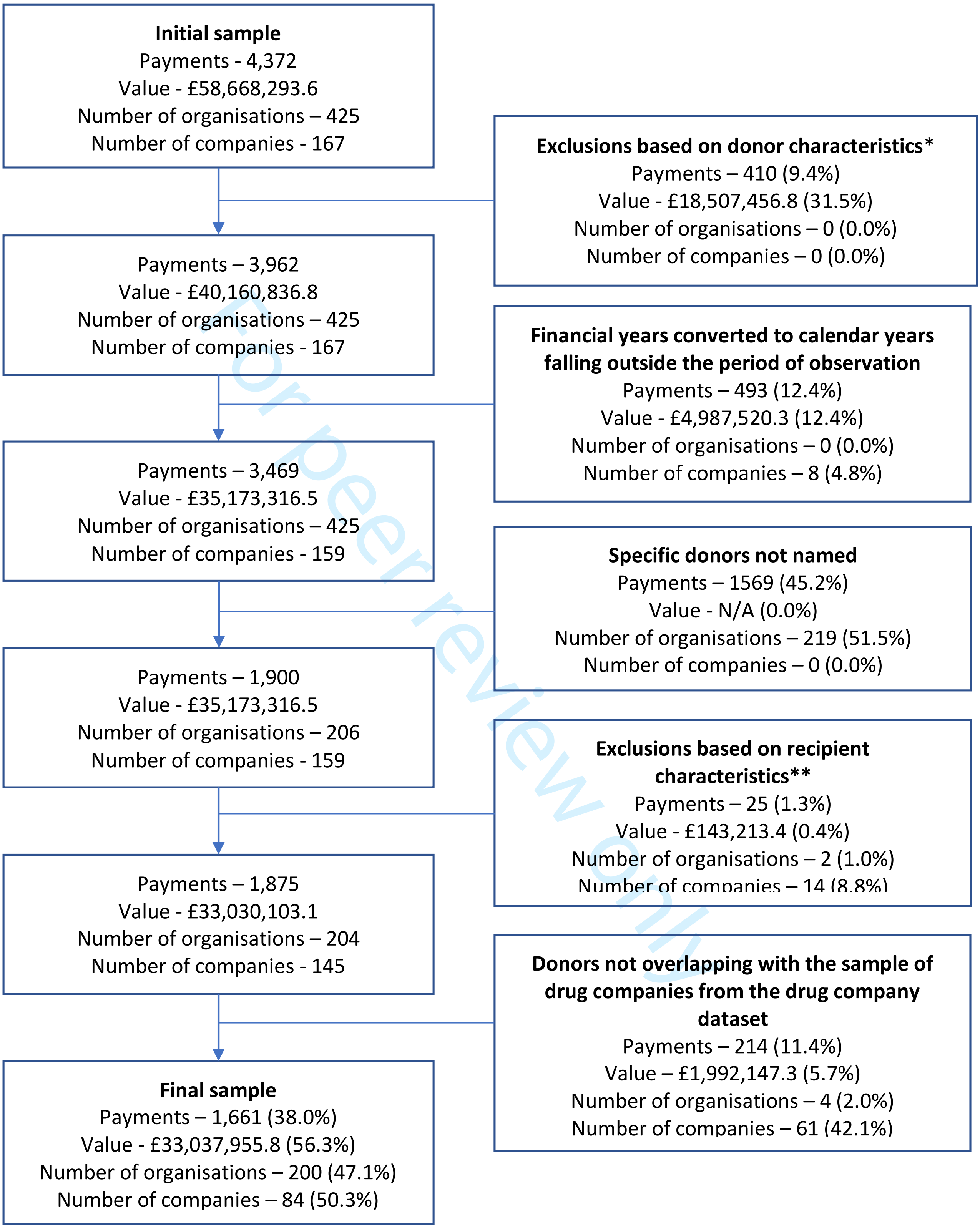

*More detailed breakdown in Table 3
**More detailed breakdown in Table 4

For peer review only

Table 3 Exclusions based on donor characteristics – detailed breakdown

Reason for exclusion	Number of Payments excluded	Value of payments excluded (£)
Exclusions unique to patient organisation dataset		
Regional branch (same charity number as national branch)	229	13,213,514.2
Subsumed under a larger organisation (same charity number as larger organisation)	125	291,862.9
Years before merger	14	1,515.0
Years after merger	9	-
Exclusions resulting from exclusions in drug company dataset		
Third sector - healthcare provider	6	-
Third sector - professional organisation	6	-
Third sector - not focusing directly on health	15	5,000,564.8
Not a charity	6	-
Total	410	18,507,456.8

Table 4 Exclusions based on recipient characteristics – detailed breakdown

	Number of Payments excluded	Value of payments excluded (£)	Number of drug companies excluded	Number of patient organisations excluded
More than one organisation	8	58,485.5	7	0
Not a pharmaceutical company	17	84,727.8	7	2
Total	25	143,213.4	14	2

For peer review only

This online dataset comprises web supplements (WS) accompanying the paper entitled “An underreported re

For peer review only

Web Supplement 3. Donors - Absolute and relative differences in the number and value of payments (all ye

NUMBER OF PAYMENTS

Number of payments - industry data
Number of payments - patient organisation data
Number of drug companies with at least one payment - industry data
Number of drug companies with at least one payment - patient organisation data

NUMBER OF PAYMENTS - THRESHOLDS

Threshold 0: Number of drug companies with at least 1 one payment in at least one dataset (1 if condition me
Threshold 1: Number of drug companies with more than 1 one payment in at least one dataset (1 if condition
Threshold 2: Number of drug companies with more than 10 payments in at least one dataset (1 if condition m
Threshold 3: Number of drug companies with more than 100 payments in at least one dataset (1 if condition

NUMBER OF PAYMENTS - ABSOLUTE DIFFERENCES

Number of payments - threshold 0
Number of drug companies with exact match in both datasets
Number of drug companies with more paymens t in industry data
Number of drug companies with more payments in patient organisation data

Highest absolute difference between patient organisation and industry data - number of payments higher in i
Highest absolute difference between patient organisation and industry data - number of payments higher in p

Number of payments - threshold 1
Number of drug companies with exact match in both datasets
Number of drug companies with more paymens t in industry data
Number of drug companies with more payments in patient organisation data

Highest absolute difference between patient organisation and industry data - number of payments higher in i
Highest absolute difference between patient organisation and industry data - number of payments higher in p

Number of payments - threshold 2
Number of drug companies with exact match in both datasets
Number of drug companies with more paymens t in industry data
Number of drug companies with more payments in patient organisation data

Highest absolute difference between patient organisation and industry data - number of payments higher in i
Highest absolute difference between patient organisation and industry data - number of payments higher in p

Number of payments - threshold 3
Number of drug companies with exact match in both datasets
Number of drug companies with more paymens t in industry data

For peer review only

Number of drug companies with more payments in patient organisation data

Highest absolute difference between patient organisation and industry data - number of payments higher in i
Highest absolute difference between patient organisation and industry data - number of payments higher in p

NUMBER OF PAYMENTS - RELATIVE DIFFERENCES

Relative difference <10%
Relative difference <20%
Relative difference <50%
Relative difference = 100%

Number of payments - threshold 1
Relative difference <10%
Relative difference <20%
Relative difference <50%
Relative difference = 100%

Number of payments - threshold 2
Relative difference <10%
Relative difference <20%
Relative difference <50%
Relative difference = 100%

Number of payments - threshold 3
Relative difference <10%
Relative difference <20%
Relative difference <50%
Relative difference = 100%

VALUE OF PAYMENTS - ABSOLUTE DIFFERENCES

Value of payments - industry data (2016 £)
Value of payments - patient organisation data (2016 £)
Number of payments with value >£0 - industry data
Number of payments with value >£0 - patient organisation data
Number of drug companies with at least one payment with the value >£0 - industry data
Number of drug companies with at least one payment with the value >£0 - patient organisation data

VALUE OF PAYMENTS - THRESHOLDS

Threshold 1: Number of drug companies with at least one payment with value >£0 in at least one dataset (1 i
Threshold 2: Number of drug companies with payments with the value of more than £10,000 in at least one c
Threshold 3: Number of drug companies with payments with the value of more than £100,000 in at least one

VALUE OF PAYMENTS - ABSOLUTE DIFFERENCES

Number of payments - threshold 1

For peer review only

Number of drug companies with exact match in both datasets
Number of drug companies with higher payment value in industry data
Number of drug companies with higher payment value in patient organisation data

Highest absolute difference between patient organisation and industry data - number of payments higher in i
Highest absolute difference between patient organisation and industry data - number of payments higher in [illegible]

Number of payments - threshold 2
Number of drug companies with exact match in both datasets
Number of drug companies with higher payment value in industry data
Number of drug companies with higher payment value in patient organisation data

Highest absolute difference between patient organisation and industry data - number of payments higher in i
Highest absolute difference between patient organisation and industry data - number of payments higher in [illegible]

Number of payments - threshold 3
Number of drug companies with exact match in both datasets
Number of drug companies with higher payment value in industry data
Number of drug companies with higher payment value in patient organisation data

Highest absolute difference between patient organisation and industry data - number of payments higher in i
Highest absolute difference between patient organisation and industry data - number of payments higher in [illegible]

VALUE OF PAYMENTS - RELATIVE DIFFERENCES

Relative difference <10%
Relative difference <20%
Relative difference <50%
Relative difference = 100%

Number of payments - threshold 1
Relative difference <10%
Relative difference <20%
Relative difference <50%
Relative difference = 100%

Number of payments - threshold 2
Relative difference <10%
Relative difference <20%
Relative difference <50%
Relative difference = 100%

Number of payments - threshold 3
Relative difference <10%
Relative difference <20%

For peer review only

Relative difference <50%
Relative difference = 100%

For peer review only

		A. Menarini	Abbvie	Actavis	Actelion
		1	1	0	1
Total					
4316		3	281	5	38
1661		0	84	5	23
63		1	1	1	1
84		0	1	1	1
Number of drug c	% of all drug companies with at least one payment in at least one dataset				
87	100.0%	1	1	1	1
80	92.0%	1	1	1	1
47	54.0%		1		1
10	11.5%		1		
Number of drug c	% of all drug companies at a given threshold				
3	3.4%			1	
49	56.3%	1	1		1
35	40.2%				
Number of payments					
757		3	197		15
53					
Number of drug c	% of all drug companies at a given threshold				
3	3.8%			1	
49	61.3%	1	1		1
28	35.0%				
Number of payments					
757		3	197		15
53					
Number of drug c	% of all drug companies at a given threshold				
1	2.1%				
36	76.6%		1		1
10	21.3%				
Number of payments					
757			197		15
53					
Number of drug c	% of all drug companies at a given threshold				
0	0.0%				
10	100.0%		1		

For peer review only

0	0.0%				
Number of payments					
757			197		
0					
Number of drug c	% of all drug companies				
5	5.7%			0.0%	
9	10.3%			0.0%	
21	24.1%			0.0%	39.5%
27	31.0%	100.0%			
Number of drug c	% of all companies at a given threshold				
5	6.3%			0.0%	
9	11.3%			0.0%	
21	26.3%			0.0%	39.5%
20	25.0%	100.0%			
Number of drug c	% of all companies at a given threshold				
3	6.4%				
5	10.6%				
15	31.9%				39.5%
2	4.3%				
Number of drug c	% of all companies at a given threshold				
0	0.0%				
0	0.0%				
1	10.0%				
0	0.0%				
Total					
£54,071,454.2		33,907.5	3,584,604.6	49,000.0	364,970.8
£33,037,955.8		-	1,412,340.9	49,240.0	217,382.1
4235		3	281	5	38
772		0	40	2	16
62		1	1	1	1
62		0	1	1	1
Number of drug c	% of all drug companies with at least one payment with the value of at lea				
74	85.1%	1	1	1	1
45	51.7%		1		1
17	19.5%		1		
Number of drug c	% of all drug companies at a given threshold				

For peer review only

0	0.0%				
48	64.9%	1	1		1
26	35.1%			1	
Vallue of payments					
6,406,351.1		33908	2172264		147589
2,960,716.0				240	
Number of drug c	% of all drug companies at a given threshold				
0	0.0%				
32	71.1%		1		1
13	28.9%				
Vallue of payments					
6,406,351.1			2172264		147589
2,960,716.0					
Number of drug c	% of all drug companies at a given threshold				
0	0.0%				
13	76.5%		1		
4	23.5%				
Vallue of payments			0		
6,406,351.1			2172264		
2,960,716.0					
Number of drug c	% of all drug companies with at least one payment with value >£0 in at lea				
3	4.1%			0.5%	
9	12.2%			0.5%	
14	18.9%			0.5%	40.4%
24	32.4%	100.0%			
Number of drug c	% of all drug companies at a given threshold				
3	4.1%			0.5%	
9	12.2%			0.5%	
14	18.9%			0.5%	40.4%
24	32.4%	100.0%			
Number of drug c	% of all drug companies at a given threshold				
1	2.2%				
5	11.1%				
9	20.0%				40.4%
4	8.9%				
Number of drug c	% of all drug companies at a given threshold				
0	0.0%				
2	11.8%				

For peer review only

	2	11.8%
	1	5.9%

For peer review only

Aegerion	Alcon	Alexion	ALK-Abello	Allergan	Alliance	Almirall
0	0	1	1	1	1	1
5	1	5	0	25	0	9
4	9	17	2	26	1	2
1	1	1	0	1	0	1
1	1	1	1	1	1	1
1	1	1	1	1	1	1
1	1	1	1	1		1
		1		1		
1						1
	1	1	1	1	1	
1						7
	8	12	2	1	1	
1						1
	1	1	1	1		
1						7
	8	12	2	1		
		1		1		
		12		1		

For peer review only

				3.8%		
				3.8%		
20.0%				3.8%		
			100.0%		100.0%	
				3.8%		
				3.8%		
20.0%				3.8%		
			100.0%			
				3.8%		
				3.8%		
				3.8%		
35,040.1	15,150.0	60,600.0	-	678,030.8	-	62,968.9
-	83,733.8	70,583.4	-	148,240.9	-	-
5	1	5	0	25	0	9
0	5	8	0	4	0	0
1	1	1	0	1	0	1
0	1	1	0	1	0	0
st >£0 in at least one dataset						
1	1	1		1		1
				1		

For peer review only

1			1	1
	1	1		
35040			529790	62969
	68584	9983		
			1	
			529790	
ıst one dataset				
		14.1%		
		14.1%		
100.0%				100.0%
		14.1%		
		14.1%		
100.0%				100.0%

For peer review only

For peer review only

Amdipharm	Amgen	ApoPharma	Astellas	AstraZeneca	ausch and Lon
0	1	0	0	1	1
0	82	0	74	55	0
4	41	5	8	24	4
0	1	0	1	1	0
1	1	1	1	1	1
1	1	1	1	1	1
1	1	1	1	1	1
	1		1	1	
	1		1	1	
1		1			1
	41		66	31	
4		5			4
	1		1	1	
1		1			1
	41		66	31	
4		5			4
	1		1	1	
	41		66	31	

For peer review only

100.0%		100.0%			100.0%
100.0%		100.0%			100.0%
-	699,274.6	-	2,783,709.6	725,306.3	-
34,433.3	324,720.9	76,194.8	80,417.4	42,450.6	22,627.3
0	82	0	74	53	0
3	22	5	4	6	2
0	1	0	1	1	0
1	1	1	1	1	1
1	1	1	1	1	1
	1		1	1	
			1		

For peer review only

	1		1	1	
1		1			1
	374554		2703292	682856	
34433		76195			22627
	1		1	1	
	374554		2703292	682856	
			1		
			0		
			2703292		
100.0%		100.0%			100.0%
100.0%		100.0%			100.0%

For peer review only

For peer review only

Baxalta	Baxter	Bayer	Products Labor	Biogen	BioMarin
0	0	1	0	1	1
4	4	49	7	50	42
3	15	30	6	24	16
1	1	1	1	1	1
1	1	1	1	1	1
1	1	1	1	1	1
1	1	1	1	1	1
	1	1		1	1
1		1	1	1	1
	1				
1		19	1	26	26
	11				
1		1	1	1	1
	1				
1		19	1	26	26
	11				
		1		1	1
	1				
		19		26	26
	11				

For peer review only

			14.3%		
25.0%		38.8%	14.3%		
			14.3%		
25.0%		38.8%	14.3%		
		38.8%			
39,289.0	22,142.2	675,838.0	37,492.0	566,033.3	575,345.3
129,431.5	411,356.2	583,949.1	32,172.7	676,372.1	11,685.3
4	4	49	7	50	42
1	10	18	4	15	3
1	1	1	1	1	1
1	1	1	1	1	1
1	1	1	1	1	1
1	1	1		1	1

For peer review only

		1	1		1
1	1			1	
		91889	5319		563660
90143	389214			110339	
		1			1
1	1			1	
		91889			563660
90143	389214			110339	
		13.6%	14.2%	16.3%	
		13.6%	14.2%	16.3%	
		13.6%	14.2%	16.3%	
		13.6%	14.2%	16.3%	
		13.6%		16.3%	
		13.6%		16.3%	

For peer review only

For peer review only

Biotest	ehringer Ingelh	istol-Myers Squil	Britannia	Celgene	Chiesi
0	1	1	0	1	1
15	15	231	0	125	19
8	15	27	4	27	9
1	1	1	0	1	1
1	1	1	1	1	1
1	1	1	1	1	1
1	1	1	1	1	1
1	1	1		1	1
		1		1	
	1				
1		1		1	1
			1		
7		204		98	10
			4		
	1				
1		1		1	1
			1		
7		204		98	10
			4		
	1				
1		1		1	1
7		204		98	10
		1		1	

For peer review only

		204		98	
	0.0%				
	0.0%				
46.7%	0.0%				
			100.0%		
	0.0%				
	0.0%				
46.7%	0.0%				
			100.0%		
	0.0%				
	0.0%				
46.7%	0.0%				
88,420.4	133,153.7	1,759,705.1	-	1,828,014.0	188,262.1
131,337.0	134,736.3	281,373.4	3,884.0	493,270.0	57,591.4
15	15	230	0	125	19
7	7	17	1	11	2
1	1	1	0	1	1
1	1	1	1	1	1
1	1	1	1	1	1
1	1	1		1	1
		1		1	

For peer review only

		1		1	1
1	1		1		
		1478332		1334744	130671
42917	1583		3884		
		1		1	1
1	1				
		1478332		1334744	130671
42917	1583				
		1		1	
		0		0	
		1478332		1334744	
	1.2%				
	1.2%				
32.7%	1.2%				
			100.0%		
	1.2%				
	1.2%				
32.7%	1.2%				
			100.0%		
	1.2%				
	1.2%				
32.7%	1.2%				

For peer review only

For peer review only

Chugai	onsilient Healt	CSL Behring	Daiichi Sankyc	Dermal	Diurnal	Eisai
1	0	0	1	0	0	1
10	4	33	2	0	0	27
3	0	19	0	13	1	7
1	1	1	1	0	0	1
1	0	1	0	1	1	1
1	1	1	1	1	1	1
1	1	1	1	1		1
		1		1		1
1	1	1	1			1
				1	1	
7	4	14	2			20
				13	1	
1	1	1	1			1
				1		
7	4	14	2			20
				13		
		1				1
				1		
		14				20
				13		

For peer review only

		42.4%				
	100.0%		100.0%	100.0%	100.0%	
		42.4%				
	100.0%		100.0%	100.0%		
		42.4%				
				100.0%		
19,784.9	41,467.4	627,645.1	16,833.3	-	-	187,422.6
-	-	501,753.7	-	-	-	-
9	4	33	2	0	0	27
0	0	13	0	0	0	0
1	1	1	1	0	0	1
0	0	1	0	0	0	0
1	1	1	1			1
		1				1

For peer review only

1	1	1	1	1
19785	41467	125891	16833	187423
		1		1
		125891		187423
100.0%	100.0%	20.1%	100.0%	100.0%
100.0%	100.0%	20.1%	100.0%	100.0%
		20.1%		100.0%

For peer review only

For peer review only

Ferring	Flynn Pharma	enius Medical	edeon Richte	Genzyme	Gilead
0	0	1	0	0	0
23	19	0	0	0	174
29	4	3	2	52	90
1	1	0	0	0	1
1	1	1	1	1	1
1	1	1	1	1	1
1	1	1	1	1	1
1	1			1	1
					1
	1				1
1		1	1	1	
	15				84
6		3	2	52	
	1				1
1		1	1	1	
	15				84
6		3	2	52	
	1				1
1				1	
	15				84
6				52	
					1

For peer review only

					84
20.7%					48.3%
		100.0%	100.0%	100.0%	
20.7%					48.3%
		100.0%	100.0%	100.0%	
20.7%					48.3%
				100.0%	
					48.3%
180,131.8	38,577.9	-	-	-	1,616,738.8
44,886.0	5,175.2	15,940.6	-	1,225,894.6	1,306,716.1
23	19	0	0	0	174
3	3	3	0	25	69
1	1	0	0	0	1
1	1	1	0	1	1
1	1	1		1	1
1				1	1
				1	1

For peer review only

1	1			1
		1	1	
135246	33403			310023
		15941	1225895	
1				1
			1	
135246				310023
			1225895	
				1
			1	
			0	0
				310023
			1225895	
				19.2%
				19.2%
		100.0%	100.0%	
				19.2%
				19.2%
		100.0%	100.0%	
				19.2%
				19.2%
			100.0%	
				19.2%

For peer review only

19.2%

100.0%

For peer review only

Drug company names					
**Grünenthal**	**GSK**	**HRA Pharma**	**Ipsen**	**IQVIA**	**Janssen**
1	1	0	1	1	1
17	20	6	5	0	88
3	73	3	11	1	63
1	1	1	1	0	1
1	1	1	1	1	1
1	1	1	1	1	1
1	1	1	1		1
1	1		1		1
1		1			1
	1		1	1	
14		3			25
	53		6	1	
1		1			1
	1		1		
14		3			25
	53		6		
1					1
	1		1		
14					25
	53		6		

For peer review only

					28.4%
				100.0%	
					28.4%
					28.4%
63,036.7	501,099.7	92,429.9	37,342.0	-	1,017,042.6
8,728.1	3,461,815.7	-	148,100.2	-	376,373.5
17	20	6	5	0	88
2	36	0	7	0	25
1	1	1	1	0	1
1	1	0	1	0	1
1	1	1	1		1
	1		1		1
	1				1

For peer review only

1		1		1
	1		1	
54309		92430		640669
	2960716		110758	
				1
	1		1	
				640669
	2960716		110758	
				1
	1			
	0			0
				640669
	2960716			
		100.0%		
		100.0%		

For peer review only

For peer review only

Jazz	LEO Pharma	Lilly	Lundbeck	Meda	Merck
0	1	1	1	0	1
0	35	239	33	12	50
1	20	32	3	4	46
0	1	1	1	1	1
1	1	1	1	1	1
1	1	1	1	1	1
	1	1	1	1	1
	1	1	1	1	1
		1			
	1	1	1	1	1
1					
	15	207	30	8	4
1					
	1	1	1	1	1
	15	207	30	8	4
	1	1	1	1	1
	15	207	30	8	4
		1			

For peer review only

		207			
					8.0%
					8.0%
	42.9%				8.0%
100.0%					
					8.0%
					8.0%
	42.9%				8.0%
					8.0%
					8.0%
	42.9%				8.0%
-	151,378.2	2,825,173.2	223,802.5	77,182.6	455,388.5
-	10,000.0	143,038.3	-	75,750.0	905,077.7
0	34	237	33	12	50
0	1	12	0	1	19
0	1	1	1	1	1
0	1	1	0	1	1
	1	1	1	1	1
	1	1	1		1
		1			

1	1	1	1	
				1
141378	2682135	223803	1433	
				449689
1	1	1		
				1
141378	2682135	223803		
				449689
	1			
	0			
	2682135			
			1.9%	
			1.9%	
			1.9%	49.7%
		100.0%		
			1.9%	
			1.9%	
			1.9%	49.7%
		100.0%		
				49.7%
		100.0%		

For peer review only

For peer review only

bishi Tanabe P	MSD	Mylan	pp Pharmaceuti	Norgine	Novartis
1	1	0	1	1	1
0	235	0	60	10	435
1	55	1	24	10	154
0	1	0	1	1	1
1	1	1	1	1	1
1	1	1	1	1	1
	1		1	1	1
	1		1		1
	1				1
				1	
	1		1		1
1		1			
	180		36		281
1		1			
				1	
	1		1		1
	180		36		281
	1		1		1
	180		36		281
	1				1

For peer review only

	180				281
				0.0%	
				0.0%	
				0.0%	
100.0%		100.0%			
				0.0%	
				0.0%	
				0.0%	
-	1,750,167.8	-	173,152.9	102,297.0	5,195,484.0
5,070.3	837,180.0	-	82,633.4	33,700.5	2,102,441.4
0	233	0	60	10	433
1	29	0	10	2	104
0	1	0	1	1	1
1	1	0	1	1	1
1	1		1	1	1
	1		1	1	1
	1				1

For peer review only

1	1	1	1	1
5070	912988	90519	68596	3093043
	1	1	1	1
	912988	90519	68596	3093043
	1			1
	0			0
	912988			3093043
100.0%				
100.0%				

For peer review only

For peer review only

Novo Nordisk	Octapharma	Orion Pharma	Orphan Europe	ka Pharmaceut	Pfizer
1	0	1	0	1	1
47	2	8	0	13	886
17	7	4	3	11	129
1	1	1	0	1	1
1	1	1	1	1	1
1	1	1	1	1	1
1	1	1	1	1	1
1				1	1
					1
1		1		1	1
	1		1		
30		4		2	757
	5		3		
1		1		1	1
	1		1		
30		4		2	757
	5		3		
1				1	1
30				2	757
					1

For peer review only

					757
				15.4%	
				15.4%	
			100.0%		
				15.4%	
				15.4%	
			100.0%		
				15.4%	
				15.4%	
753,861.7	5,276.0	49,682.0	-	184,272.2	9,304,016.7
49,315.6	435,156.4	-	1,028.5	88,840.6	10,713,090.3
47	2	8	0	13	831
3	7	0	1	6	57
1	1	1	0	1	1
1	1	0	1	1	1
1	1	1	1	1	1
1	1			1	1
					1

For peer review only

1		1		1	
	1		1		1
704546		49682		95432	
	429880		1029		1409074
1				1	
	1				1
704546				95432	
	429880				1409074
					1
					0
					1409074
					13.2%
					13.2%
		100.0%	100.0%		
					13.2%
					13.2%
		100.0%	100.0%		
					13.2%
					13.2%
					13.2%

For peer review only

13.2%

For peer review only

Pharma Mar	PTC	RB	Roche Products	ont Pharmace	Sanofi
1	0	0	1	0	1
0	0	0	99	7	224
2	6	4	70	1	37
0	0	0	1	1	1
1	1	1	1	1	1
1	1	1	1	1	1
1	1	1	1	1	1
			1		1
					1
			1	1	1
1	1	1			
			29	6	187
2	6	4			
			1	1	1
1	1	1			
			29	6	187
2	6	4			
			1		1
			29		187
					1

For peer review only

					187
			29.3%		
100.0%	100.0%	100.0%			
			29.3%		
100.0%	100.0%	100.0%			
			29.3%		
-	-	-	1,369,625.3	-	2,562,336.3
6,312.5	-	144,270.0	660,704.8	-	189,271.9
0	0	0	99	0	219
1	0	4	27	0	10
0	0	0	1	0	1
1	0	1	1	0	1
1		1	1		1
		1	1		1
			1		1

For peer review only

		1	1
1	1		
		708920	2373064
6313	144270		
		1	1
	1		
		708920	2373064
	144270		
		1	1
		0	0
		708920	2373064
100.0%	100.0%		
100.0%	100.0%		
	100.0%		

For peer review only

For peer review only

nofi Pasteur M	Santen	rvier Laborato	Shire	Tau Rare Dis	Sobi
0	1	1	1	0	1
0	1	20	115	0	36
2	2	3	37	8	13
0	1	1	1	0	1
1	1	1	1	1	1
1	1	1	1	1	1
1	1	1	1	1	1
		1	1		1
			1		
		1	1		1
1	1			1	
		17	78		23
2	1			8	
		1	1		1
1	1			1	
		17	78		23
2	1			8	
		1	1		1
		17	78		23
			1		

For peer review only

78

100.0%				100.0%	
100.0%				100.0%	
-	4,000.0	76,304.9	1,266,644.8	-	224,819.9
20,989.1	-	10,857.5	417,018.3	53,676.1	20,701.1
0	1	20	115	0	36
1	0	2	18	4	6
0	1	1	1	0	1
1	0	1	1	1	1
1	1	1	1	1	1
			1		1
			1		

For peer review only

	1	1	1		1
1				1	
	4000	65447	849626		204119
20989				53676	
			1		1
			849626		204119
			1		
			0		
			849626		
100.0%	100.0%			100.0%	
100.0%	100.0%			100.0%	

For peer review only

For peer review only

Stirling Anglia	Syner-Med	Takeda	Teva	Thea	Tillotts
0	0	1	0	0	0
3	0	54	24	0	7
1	3	42	28	1	6
1	0	1	1	0	1
1	1	1	1	1	1
1	1	1	1	1	1
1	1	1	1		1
		1	1		
1		1			1
	1		1	1	
2		12			1
	3		4	1	
1		1			1
	1		1		
2		12			1
	3		4		
		1			
			1		
		12			
			4		

For peer review only

			14.3%		14.3%
		22.2%	14.3%		14.3%
	100.0%			100.0%	
			14.3%		14.3%
		22.2%	14.3%		14.3%
	100.0%				
			14.3%		
		22.2%	14.3%		
11,110.0	-	6,614,214.3	642,199.4	-	48,612.7
-	-	207,863.1	274,284.3	-	32,002.9
3	0	54	23	0	7
0	0	16	13	0	2
1	0	1	1	0	1
0	0	1	1	0	1
1		1	1		1
		1	1		
		1			

For peer review only

1	1	1	1
11110	6406351	367915	16610
	1	1	
	6406351	367915	
	1		
	0		
	6406351		
			34.2%
100.0%			
			34.2%
100.0%			

For peer review only

For peer review only

UCB	Veriton	ifor Pharma Group
1	0	0
86	0	3
37	4	12
1	0	1
1	1	1
1	1	1
1	1	1
1		1
1		
	1	1
49		
	4	9
1		
	1	1
49		
	4	9
1		
		1
49		
		9

For peer review only

100.0%

100.0%

534,290.2	-	20,350.0
84,069.3	-	2,828,703.7
84	0	3
10	0	6
1	0	1
1	0	1
1		1
1		1
		1

For peer review only

1	1
450221	2808354
1	1
450221	2808354
	1
	0
	2808354

For peer review only

For peer review only

Web Supplement 4. Donors - Absolute and relative differences in the number and value of payments (indiv

NUMBER OF PAYMENTS - ABSOLUTE DIFFERENCES

Threshold 0: At least 1 one payment in at least one dataset

Total
Number of drug companies with exact match in both datasets
Number of drug companies with more paymens t in industry data
Number of drug companies with more payments in patient organisation data

Highest absolute difference between patient organisation and industry data - number of payments higher in i
Highest absolute difference between patient organisation and industry data - number of payments higher in p

Threshold 1: More than 1 one payment in at least one datase

Total
Number of drug companies with exact match in both datasets
Number of drug companies with more paymens t in industry data
Number of drug companies with more payments in patient organisation data

Highest absolute difference between patient organisation and industry data - number of payments higher in i
Highest absolute difference between patient organisation and industry data - number of payments higher in p

Threshold 2: More than 10 payments in at least one dataset

Total
Number of drug companies with exact match in both datasets
Number of drug companies with more paymens t in industry data
Number of drug companies with more payments in patient organisation data

Highest absolute difference between patient organisation and industry data - number of payments higher in i
Highest absolute difference between patient organisation and industry data - number of payments higher in p

Threshold 3: More than 100 payments in at least one dataset

Total
Number of drug companies with exact match in both datasets
Number of drug companies with more paymens t in industry data
Number of drug companies with more payments in patient organisation data

Highest absolute difference between patient organisation and industry data - number of payments higher in i
Highest absolute difference between patient organisation and industry data - number of payments higher in p

NUMBER OF PAYMENTS - RELATIVE DIFFERENCES

Threshold 0: At least 1 one payment in at least one dataset

Total
Relative difference <10%

For peer review only

Relative difference <20%
Relative difference <50%
Relative difference = 100%

Threshold 1: More than 1 one payment in at least one datase
Total
Relative difference <10%
Relative difference <20%
Relative difference <50%
Relative difference = 100%

Threshold 2: More than 10 payments in at least one dataset
Total
Relative difference <10%
Relative difference <20%
Relative difference <50%
Relative difference = 100%

Threshold 3: More than 100 payments in at least one dataset
Total
Relative difference <10%
Relative difference <20%
Relative difference <50%
Relative difference = 100%

VALUE OF PAYMENTS - ABSOLUTE DIFFERENCES

Threshold 1: At least one payment with value >£0 in at least one dataset
Total
Number of drug companies with exact match in both datasets
Number of drug companies with higher payment value in industry data
Number of drug companies with higher payment value in patient organisation data

Highest absolute difference between patient organisation and industry data - number of payments higher in i
Highest absolute difference between patient organisation and industry data - number of payments higher in p

Threshold 2: Payments with the value of more than £10,000 in at least one dataset
Total
Number of drug companies with exact match in both datasets
Number of drug companies with higher payment value in industry data
Number of drug companies with higher payment value in patient organisation data

Highest absolute difference between patient organisation and industry data - number of payments higher in i
Highest absolute difference between patient organisation and industry data - number of payments higher in p

Threshold 3: Payments with the value of more than £100,000 in at least one dataset

For peer review only

Total
Number of drug companies with exact match in both datasets
Number of drug companies with higher payment value in industry data
Number of drug companies with higher payment value in patient organisation data

Highest absolute difference between patient organisation and industry data - number of payments higher in i
Highest absolute difference between patient organisation and industry data - number of payments higher in p

VALUE OF PAYMENTS- RELATIVE DIFFERENCES

Threshold 1: At least one payment with value >£0 in at least one dataset
Total
Relative difference <10%
Relative difference <20%
Relative difference <50%
Relative difference = 100%

Threshold 2: Payments with the value of at least £10,000 in at least one dataset
Total
Relative difference <10%
Relative difference <20%
Relative difference <50%
Relative difference = 100%

Threshold 3: Payments with the value of at least 100,000 in at least one dataset
Total
Relative difference <10%
Relative difference <20%
Relative difference <50%
Relative difference = 100%

For peer review only

All years			2012			2013		
Number of drug c	% of all drug	0	Number of drug	% of all drug	0	Number of dr	% of all drug	0
87	100.0%		61	100.0%		68	100.0%	
3	3.4%		1	1.6%		7	10.3%	
49	56.3%		28	45.9%		28	41.2%	
35	40.2%		32	52.5%		33	48.5%	
Number of payments			Number of payments			Number of payments		
757			216			135		
53			18			16		
Number of drug c	% of all drug	0	Number of drug	% of all drug	0	Number of dr	% of all drug	0
80	100.0%		46	100.0%		55	100.0%	
3	3.8%		1	2.2%		3	5.5%	
49	61.3%		28	60.9%		27	49.1%	
28	35.0%		17	37.0%		25	45.5%	
Number of payments			Number of payments			Number of payments		
757			216			135		
53			18			16		
Number of drug c	% of all drug	0	Number of drug	% of all drug	0	Number of dr	% of all drug	0
47	100.0%		16	100.0%		17	100.0%	
1	2.1%		0	0.0%		0	0.0%	
36	76.6%		13	81.3%		14	82.4%	
10	21.3%		3	18.8%		3	17.6%	
Number of payments			Number of payments			Number of payments		
757			216			135		
53			18			16		
Number of drug c	% of all drug	0	Number of drug	% of all drug	0	Number of dr	% of all drug	0
10	100.0%		1	100.0%		1	100.0%	
0	0.0%		0	0.0%		0	0.0%	
10	100.0%		1	100.0%		1	100.0%	
0	0.0%		0	0.0%		0	0.0%	
Number of payments			Number of payments			Number of payments		
757			216			135		
0			0			0		
Number of drug c	% of all drug co		Number of drug	% of all drug co		Number of dr	% of all drug co	
87	100.0%		61	100.0%		68	100.0%	
5	5.7%		1	1.6%		7	10.3%	

For peer review only

9	10.3%		2	3.3%		7	10.3%	
21	24.1%		7	11.5%		10	14.7%	
27	31.0%		36	59.0%		33	48.5%	

Number of drug c	% of all drug	0	Number of drug	% of all drug	0	Number of dr	% of all drug	0
80	100.0%		46	100.0%		55	100.0%	
5	6.3%		1	2.2%		3	5.5%	
9	11.3%		2	4.3%		3	5.5%	
21	26.3%		7	15.2%		6	10.9%	
20	25.0%		21	45.7%		24	43.6%	

Number of drug c	% of all drug	0	Number of drug	% of all drug	0	Number of dr	% of all drug	0
47	100.0%		16	100.0%		17	100.0%	
3	6.4%		0	0.0%		0	0.0%	
5	10.6%		0	0.0%		0	0.0%	
15	31.9%		1	6.3%		0	0.0%	
2	4.3%		3	18.8%		3	17.6%	

Number of drug c	% of all drug	0	Number of drug	% of all drug	0	Number of dr	% of all drug	0
10	100.0%		1	100.0%		1	100.0%	
0	0.0%		0	0.0%		0	0.0%	
0	0.0%		0	0.0%		0	0.0%	
1	10.0%		0	0.0%		0	0.0%	
0	0.0%		0	0.0%		0	0.0%	

Number of drug c	% of all drug	0	Number of drug	% of all drug	0	Number of dr	% of all drug	0
74	100.0%		52	100.0%		56	100.0%	
0	0.0%		0	0.0%		0	0.0%	
48	64.9%		26	50.0%		35	62.5%	
26	35.1%		26	50.0%		21	37.5%	

Value of payments	Value of payments	Value of payments
6,406,351.2	1,576,293.48	666,171.11
2,960,716.0	501,114.66	851,009.34

Number of drug c	% of all drug	0	Number of drug	% of all drug	0	Number of dr	% of all drug	0
45	100.0%		20	100.0%		24	100.0%	
0	0.0%		0	0.0%		0	0.0%	
32	71.1%		13	65.0%		14	58.3%	
13	28.9%		7	35.0%		10	41.7%	

Value of payments	Value of payments	Value of payments
6,406,351.2	1,576,293.5	666,171.1
2,960,716.0	501,114.7	851,009.3

Number of drug c	% of all drug	0	Number of drug	% of all drug	0	Number of dr	% of all drug	0

For peer review only

17	100.0%		2	100.0%		1	100.0%	
0	0.0%		0	0.0%		0	0.0%	
13	76.5%		2	100.0%		1	100.0%	
4	23.5%		0	0.0%		0	0.0%	
Value of payments			Value of payments			Value of payments		
6,406,351.2			1,576,293.5			604,556.0		
2,960,716.0			-			-		
Number of drug c	% of all drug	0	Number of drug	% of all drug	0	Number of dr	% of all drug	0
74	100.0%		52	100.0%		56	100.0%	
3	4.1%		1	1.9%		0	0.0%	
9	12.2%		1	1.9%		4	7.1%	
14	18.9%		4	7.7%		7	12.5%	
24	32.4%		32	61.5%		30	53.6%	
Number of drug c	% of all drug	0	Number of drug	% of all drug	0	Number of dr	% of all drug	0
45	100.0%		20	100.0%		24	100.0%	
1	2.2%		0	0.0%		0	0.0%	
5	11.1%		0	0.0%		1	4.2%	
9	20.0%		1	5.0%		3	12.5%	
4	8.9%		8	40.0%		9	37.5%	
Number of drug c	% of all drug	0	Number of drug	% of all drug	0	Number of dr	% of all drug	0
17	100.0%		2	100.0%		1	100.0%	
0	0.0%		0	0.0%		0	0.0%	
2	11.8%		0	0.0%		0	0.0%	
2	11.8%		0	0.0%		1	100.0%	
1	5.9%		0	0.0%		0	0.0%	

For peer review only

2014		2015		2016	
Number of drug	% of all drug 0	Number of drug	% of all drug 0	Number of drug	% of all drug
72	100.0%	74	100.0%	69	100.0%
4	5.6%	7	9.5%	4	5.8%
38	52.8%	42	56.8%	39	56.5%
30	41.7%	25	33.8%	26	37.7%
Number of payments		Number of payments		Number of payments	
108		125		173	
13		13		12	
Number of drug	% of all drug 0	Number of drug	% of all drug 0	Number of drug	% of all drug
55	100.0%	57	100.0%	50	100.0%
1	1.8%	5	8.8%	3	6.0%
36	65.5%	42	73.7%	38	76.0%
18	32.7%	10	17.5%	9	18.0%
Number of payments		Number of payments		Number of payments	
108		125		173	
13		13		12	
Number of drug	% of all drug 0	Number of drug	% of all drug 0	Number of drug	% of all drug
21	100.0%	23	100.0%	23	100.0%
0	0.0%	0	0.0%	0	0.0%
18	85.7%	22	95.7%	22	95.7%
3	14.3%	1	4.3%	1	4.3%
Number of payments		Number of payments		Number of payments	
108		125		173	
13		13		12	
Number of drug	% of all drug 0	Number of drug	% of all drug 0	Number of drug	% of all drug
2	100.0%	1	100.0%	100.0%	100.0%
0	0.0%	0	0.0%	0	0.0%
2	100.0%	1	100.0%	1	100.0%
0	0.0%	0	0.0%	0	0.0%
Number of payments		Number of payments		Number of payments	
108		125		173	
0		0		0	
Number of drug	% of all drug co	Number of drug	% of all drug co	Number of drug	% of all drug
72	100.0%	74	100.0%	69	100.0%
4	5.6%	7	9.5%	4	5.8%

For peer review only

5	6.9%		9	12.2%		5	7.2%
12	16.7%		17	23.0%		12	17.4%
34	47.2%		28	37.8%		29	42.0%
Number of drug	% of all drug	0	Number of drug	% of all drug	0	Number of drug	% of all drug
55	100.0%		57	100.0%		50	100.0%
1	1.8%		5	8.8%		3	6.0%
2	3.6%		7	12.3%		4	8.0%
9	16.4%		15	26.3%		11	22.0%
20	36.4%		13	22.8%		11	22.0%
Number of drug	% of all drug	0	Number of drug	% of all drug	0	Number of drug	% of all drug
21	100.0%		23	100.0%		23	100.0%
0	0.0%		0	0.0%		0	0.0%
1	4.8%		0	0.0%		0	0.0%
3	14.3%		4	17.4%		2	8.7%
2	9.5%		1	4.3%		1	4.3%
Number of drug	% of all drug	0	Number of drug	% of all drug	0	Number of drug	% of all drug
2	100.0%		1	100.0%		100.0%	100.0%
0	0.0%		0	0.0%		0	0.0%
0	0.0%		0	0.0%		0	0.0%
0	0.0%		0	0.0%		0	0.0%
0	0.0%		0	0.0%		0	0.0%
Number of drug	% of all drug	0	Number of drug	% of all drug	0	Number of drug	% of all drug
59	100.0%		60	100.0%		55	100.0%
0	0.0%		1	1.7%		0	0.0%
39	66.1%		41	68.3%		38	69.1%
20	33.9%		18	30.0%		17	30.9%
Value of payments			Value of payments			Value of payments	
902,858.00			1,637,974.69			5,986,743.37	
2,123,196.45			729,868.42			5,541,099.82	
Number of drug	% of all drug	0	Number of drug	% of all drug	0	Number of drug	% of all drug
28	100.0%		30	100.0%		28	100.0%
0	0.0%		0	0.0%		0	0.0%
21	75.0%		25	83.3%		23	82.1%
7	25.0%		5	16.7%		5	17.9%
Value of payments			Value of payments			Value of payments	
902,858.0			1,637,974.7			5,986,743.4	
2,123,196.4			729,868.4			5,541,099.8	
Number of drug	% of all drug	0	Number of drug	% of all drug	0	Number of drug	% of all drug

For peer review only

3	100.0%	2	100.0%	2	100.0%
0	0.0%	0	0.0%	0	0.0%
2	66.7%	2	100.0%	1	50.0%
1	33.3%	0	0.0%	1	50.0%

Value of payments	Value of payments	Value of payments
902,858.0	1,637,974.7	5,986,743.4
2,123,196.4	-	5,541,099.8

Number of drug	% of all drug	0	Number of drug	% of all drug	0	Number of drug	% of all drug
59	100.0%		60	100.0%		55	100.0%
1	1.7%		5	8.3%		0	0.0%
1	1.7%		8	13.3%		4	7.3%
8	13.6%		17	28.3%		14	25.5%
31	52.5%		24	40.0%		22	40.0%

Number of drug	% of all drug	0	Number of drug	% of all drug	0	Number of drug	% of all drug
28	100.0%		30	100.0%		28	100.0%
1	3.6%		2	6.7%		0	0.0%
1	3.6%		2	6.7%		3	10.7%
5	17.9%		8	26.7%		10	35.7%
8	28.6%		6	20.0%		4	14.3%

Number of drug	% of all drug	0	Number of drug	% of all drug	0	Number of drug	% of all drug
3	100.0%		2	100.0%		2	100.0%
0	0.0%		0	0.0%		0	0.0%
0	0.0%		0	0.0%		0	0.0%
0	0.0%		0	0.0%		0	0.0%
1	33.3%		0	0.0%		0	0.0%

For peer review only

companies at a given threshold

companies at a given threshold

companies at a given threshold

companies at a given threshold

companies

For peer review only

companies at a given threshold

companies at a given threshold

companies at a given threshold

companies at a given threshold

companies at a given threshold

companies at a given threshold

For peer review only

companies at a given threshold

companies at a given threshold

companies at a given threshold

For peer review only

Web Supplement 5. Recipients - Absolute and relative differences in the number and value of payments (al

NUMBER OF PAYMENTS

Number of payments - industry data
Number of payments - patient organisation data
Number of patient organisations with at least 1 payment - industry data
Number of patient organisations with at least 1 payment - patient organisation data

NUMBER OF PAYMENTS - THRESHOLDS

Threshold 0: Number of patient organisations with at least 1 one payment in at least one dataset
Threshold 1: Number of patient organisations with more than 1 one payment in at least one dataset
Threshold 2: Number of patient organisations with more than 10 payments in at least one dataset
Threshold 3: Number of patient organisations with more than 100 payments in at least one dataset

NUMBER OF PAYMENTS - ABSOLUTE DIFFERENCES

Number of payments - threshold 0
Number of patient organisations with exact match in both datasets
Number of patient organisations with more paymens in industry data
Number of patient organisations with more payments in patient organisation data

Highest absolute difference between patient organisation and industry data - number of payments higher in i
Highest absolute difference between patient organisation and industry data - number of payments higher in p

Number of payments - threshold 1
Number of patient organisations with exact match in both datasets
Number of patient organisations with more paymens in industry data
Number of patient organisations with more payments in patient organisation data

Highest absolute difference between patient organisation and industry data - number of payments higher in i
Highest absolute difference between patient organisation and industry data - number of payments higher in p

Number of payments - threshold 2
Number of patient organisations with exact match in both datasets
Number of patient organisations with more paymens in industry data
Number of patient organisations with more payments in patient organisation data

Highest absolute difference between patient organisation and industry data - number of payments higher in i
Highest absolute difference between patient organisation and industry data - number of payments higher in p

Number of payments - threshold 3
Number of patient organisations with exact match in both datasets
Number of patient organisations with more paymens in industry data

For peer review only

Number of patient organisations with more payments in patient organisation data

Highest absolute difference between patient organisation and industry data - number of payments higher in i
Highest absolute difference between patient organisation and industry data - number of payments higher in p

NUMBER OF PAYMENTS - RELATIVE DIFFERENCES

Relative difference <10%
Relative difference <20%
Relative difference <50%
Relative difference = 100%

Number of payments - threshold 1
Relative difference <10%
Relative difference <20%
Relative difference <50%
Relative difference = 100%

Number of payments - threshold 2
Relative difference <10%
Relative difference <20%
Relative difference <50%
Relative difference = 100%

Number of payments - threshold 3
Relative difference <10%
Relative difference <20%
Relative difference <50%
Relative difference = 100%

VALUE OF PAYMENTS - ABSOLUTE DIFFERENCES

Value of payments - industry data (2016 £)
Value of payments - patient organisation data (2016 £)
Number of payments with value >£0 - industry data
Number of payments with value >£0 - patient organisation data
Number of patient organisations with at least one payemnt with the value >£0 - industry data
Number of patient organisations with at least one payemnt with the value >£0 - patient organisation data

VALUE OF PAYMENTS - THRESHOLDS

Threshold 1: Number of patient organisations with at least one payment with value >0 in at least one dataset
Threshold 2: Number of patient organisations with payments with the value of more than £10,000 in at least
Threshold 3: Number of patient organisations with payments with the value of more than £100,000 in at leas

VALUE OF PAYMENTS - ABSOLUTE DIFFERENCES

Number of payments - threshold 1

For peer review only

Number of patient organisations with exact match in both datasets
Number of patient organisations with higher payment value in industry data
Number of patient organisations with higher payment value in patient organisation data

Highest absolute difference between patient organisation and industry data - number of payments higher in i
Highest absolute difference between patient organisation and industry data - number of payments higher in [illegible]

Number of payments - threshold 2

Number of patient organisations with exact match in both datasets
Number of patient organisations with higher payment value in industry data
Number of patient organisations with higher payment value in patient organisation data

Highest absolute difference between patient organisation and industry data - number of payments higher in i
Highest absolute difference between patient organisation and industry data - number of payments higher in [illegible]

Number of payments - threshold 3

Number of patient organisations with exact match in both datasets
Number of patient organisations with higher payment value in industry data
Number of patient organisations with higher payment value in patient organisation data

Highest absolute difference between patient organisation and industry data - number of payments higher in i
Highest absolute difference between patient organisation and industry data - number of payments higher in [illegible]

VALUE OF PAYMENTS - RELATIVE DIFFERENCES

Relative difference <10%
Relative difference <20%
Relative difference <50%
Relative difference = 100%

Number of payments - threshold 1

Relative difference <10%
Relative difference <20%
Relative difference <50%
Relative difference = 100%

Number of payments - threshold 2

Relative difference <10%
Relative difference <20%
Relative difference <50%
Relative difference = 100%

Number of payments - threshold 3

Relative difference <10%
Relative difference <20%

For peer review only

Relative difference <50%

Relative difference = 100%

For peer review only

		21 and Co	AADC Resear	Action Bladd	Action Duche
Total					
4316		1	1	1	12
1661		0	0	0	0
425		1	1	1	1
200		0	0	0	0
Number of patien	% of all patient organisations with at least one payment in at least one				
425	100.0%	1	1	1	1
299	70.4%				1
115	27.1%				1
3	0.7%				
Number of patien	% of all patient organisations at a given threshold				
33	7.8%				
335	78.8%	1	1	1	1
57	13.4%				
Number of payments					
199		1	1	1	12
33					
Number of patien	% of all patient organisations at a given threshold				
13	4.3%				
229	76.6%				1
57	19.1%				
Number of payments					
199					12
33					
Number of patien	% of all patient organisations at a given threshold				
2	1.7%				
95	82.6%				1
18	15.7%				
Number of payments					
199					12
33					
Number of patien	% of all patient organisations at a given threshold				
0	0.0%				
3	100.0%				

For peer review only

0	0.0%				
Number of payments					
199					
0					
Number of patien	% of all patient organisations with at least one payment in at least one				
35	8.2%				
42	9.9%				
81	19.1%				
225	52.9%	100.0%	100.0%	100.0%	100.0%
Number of patien	% of all patient organisations at a given threshold				
15	5.0%				
22	7.4%				
61	20.4%				
119	39.8%				100.0%
Number of patien	% of all patient organisations at a given threshold				
4	3.5%				
7	6.1%				
31	27.0%				
33	28.7%				100.0%
Number of patien	% of all patient organisations at a given threshold				
0	0.0%				
0	0.0%				
0	0.0%				
1	33.3%				
£54,071,454.2		1029	5070	101	47076
£33,037,955.8		0	0	0	0
4235		1	1	1	12
772		0	0	0	0
416		1	1	1	1
121		0	0	0	0
416		1	1	1	1
104					
13					
Number of patien	% of all patient organisations at a given threshold				

For peer review only

8	1.9%				
356	85.6%	1	1	1	1
52	12.5%				
Vallue of payments		0	0	0	0
6,718,576.6		1029	5070	101	47076
6,493,237.1					
Number of patien	% of all patient organisations at a given threshold				
0	0.0%				
80	76.9%				
24	23.1%				
Vallue of payments					
6,718,576.6					
6,493,237.1					
Number of patien	% of all patient organisations at a given threshold				
0	0.0%				
6	46.2%				
7	53.8%				
Vallue of payments					
6,718,576.6					
6,493,237.1					
Number of patien	% of all patient organisations with at least one payment with value >£0				
21	5.0%				
34	8.2%				
73	17.5%				
295	70.9%	100.0%	100.0%	100.0%	100.0%
21	5.0%				
34	8.2%				
73	17.5%				
295	70.9%	100.0%	100.0%	100.0%	100.0%
4	3.8%				
8	7.7%				
23	22.1%				
54	51.9%				
0	0.0%				
0	0.0%				

For peer review only

3	23.1%
4	30.8%

For peer review only

	Action for Pu	Action on Pa	Action on Pr	Action on Sm	Action on Sm	Actionplus F	Add+Up
	3	12	1	1	5	2	3
	2	0	1	0	5	0	5
	1	1	1	1	1	1	1
	1	0	1	0	1	0	1
dataset							
	1	1	1	1	1	1	1
	1	1			1	1	1
		1					
			1		1		
	1	1		1		1	
							1
	1	12		1		2	
							2
					1		
	1	1				1	
							1
	1	12				2	
							2
		1					
		12					

For peer review only

dataset							
			0.0%		0.0%		
			0.0%		0.0%		
	33.3%		0.0%		0.0%		40.0%
		100.0%		100.0%		100.0%	
					0.0%		
					0.0%		
	33.3%				0.0%		40.0%
		100.0%				100.0%	
		100.0%					
	14053	13947	10521	2520	28933	7941	30629
	18300	0	10285	0	33478	0	37297
	3	10	1	1	5	2	3
	2	0	1	0	5	0	5
	1	1	1	1	1	1	1
	1	0	1	0	1	0	1
	1	1	1	1	1	1	1

For peer review only

	1	1	1		1	
1				1		1
0	0	0	0	0	0	0
	13947	236	2520		7941	
4247				4545		6667

in at least one dataset

		2.2%				
		2.2%		13.6%		17.9%
23.2%		2.2%		13.6%		17.9%
	100.0%		100.0%		100.0%	
		2.2%				
		2.2%		13.6%		17.9%
23.2%		2.2%		13.6%		17.9%
	100.0%		100.0%		100.0%	

For peer review only

Addaction	Addenbrooke:	Addison's Di:	Adfam	ADHD Found	Africa Advoc:	African Healt
20	1	3	1	9	8	12
2	0	1	2	1	7	6
1	1	1	1	1	1	1
1	0	1	1	1	1	1
1	1	1	1	1	1	1
1		1	1	1	1	1
1						1
1	1	1		1	1	1
			1			
18	1	2		8	1	6
			1			
1		1		1	1	1
			1			
18		2		8	1	6
			1			
1						1
18						6

For peer review only

					12.5%	
					12.5%	
	100.0%					
					12.5%	
					12.5%	
356861	3042	25000	270	27042	40023	57095
98542	0	25000	35800	20281	35149	23988
20	1	3	1	9	8	12
2	0	1	2	1	6	5
1	1	1	1	1	1	1
1	0	1	1	1	1	1
1	1	1	1	1	1	1
1						

For peer review only

		1				
1	1			1	1	1
			1			
0	0	0	0	0	0	0
258319	3042			6761	4874	33107
			35530			
1						
0						
258319						
		0.0%				
		0.0%			12.2%	
		0.0%		25.0%	12.2%	
	100.0%					
		0.0%				
		0.0%			12.2%	
		0.0%		25.0%	12.2%	
	100.0%					

For peer review only

For peer review only

African-Carib	African's Get	Age Related	Age UK	Age UK - Cym	AGE UK - Isle	aHUS UK
6	1	2	12	2	1	1
0	0	0	4	4	2	0
1	1	1	1	1	1	1
0	0	0	1	1	1	0
1	1	1	1	1	1	1
1		1	1	1	1	
			1			
1	1	1	1			1
				1	1	
6	1	2	8			1
				2	1	
1		1	1			
				1	1	
6		2	8			
				2	1	
			1			
			8			

For peer review only

100.0%	100.0%	100.0%				100.0%
100.0%		100.0%				
70152	130	39259	97127	11210	42574	25250
0	0	0	1825301	0	24278	0
6	1	2	12	2	1	1
0	0	0	1	0	1	0
1	1	1	1	1	1	1
0	0	0	1	0	1	0
1	1	1	1	1	1	1
			1			
			1			

For peer review only

1	1	1		1	1	1
			1			
0	0	0	0	0	0	0
70152	130	39259		11210	18296	25250
			1728174			
			1			
			0			
			1728174			
			1			
			0			
			1728174			
					43.0%	
100.0%	100.0%	100.0%		100.0%		100.0%
					43.0%	
100.0%	100.0%	100.0%		100.0%		100.0%

For peer review only

For peer review only

Airedale Volu	Alcohol Conc	ALD Life	Allergy Resea	Allergy UK	Alpha-1 Awa	Alpha-1 UK S
3	18	1	2	23	2	5
3	2	0	0	8	0	0
1	1	1	1	1	1	1
1	1	0	0	1	0	0
1	1	1	1	1	1	1
1	1		1	1	1	1
	1			1		
1						
	1	1	1	1	1	1
	16	1	2	15	2	5
1						
	1		1	1	1	1
	16		2	15	2	5
	1			1		
	16			15		

For peer review only

0.0%						
0.0%						
0.0%						
		100.0%	100.0%		100.0%	100.0%
0.0%						
0.0%						
0.0%						
			100.0%		100.0%	100.0%
34921	128034	262	21042	280169	16456	138585
34921	0	0	0	86271	0	0
3	18	1	2	23	2	5
3	0	0	0	2	0	0
1	1	1	1	1	1	1
1	0	0	0	1	0	0
1	1	1	1	1	1	1
	1			1		1

For peer review only

1						
	1	1	1	1	1	1
0	0	0	0	0	0	0
	128034	262	21042	193898	16456	138585
	1			1		1
	0			0		0
	128034			193898		138585
0.0%						
0.0%						
0.0%						
	100.0%	100.0%	100.0%		100.0%	100.0%
0.0%						
0.0%						
0.0%						
	100.0%	100.0%	100.0%		100.0%	100.0%
	100.0%					100.0%

For peer review only

For peer review only

Alzheimer Sc	Alzheimer's F	Alzheimer’s S	Alzheimers D	Anaphylaxis (	Angelman Sy	Ankylosing S
2	7	18	1	8	1	6
0	10	5	0	3	0	0
1	1	1	1	1	1	1
0	1	1	0	1	0	0
1	1	1	1	1	1	1
1	1	1		1		1
		1				
1		1	1	1	1	1
	1					
2		13	1	5	1	6
	3					
1		1		1		1
	1					
2		13		5		6
	3					
		1				
		13				

	30.0%					
100.0%			100.0%		100.0%	100.0%
	30.0%					
100.0%						100.0%
564	443860	253057	1057	45474	303	38723
0	0	0	0	0	0	0
2	7	18	1	8	1	6
0	0	0	0	0	0	0
1	1	1	1	1	1	1
0	0	0	0	0	0	0
1	1	1	1	1	1	1
	1	1				

For peer review only

1	1	1	1	1	1	1
0	0	0	0	0	0	0
564	443860	253057	1057	45474	303	38723
	1	1				
	0	0				
	443860	253057				
100.0%	100.0%	100.0%	100.0%	100.0%	100.0%	100.0%
100.0%	100.0%	100.0%	100.0%	100.0%	100.0%	100.0%
	100.0%	100.0%				

For peer review only

For peer review only

Anthony Nol	Anticoagulati	Anxiety UK	Aplastic Ana	Arrhythmia A	Arthritis and	Arthritis Care
3	76	8	1	34	32	50
2	0	2	0	0	0	10
1	1	1	1	1	1	1
1	0	1	0	0	0	1
1	1	1	1	1	1	1
1	1	1		1	1	1
	1			1	1	1
1	1	1	1	1	1	1
1	76	6	1	34	32	40
1	1	1		1	1	1
1	76	6		34	32	40
	1			1	1	1
	76			34	32	40

For peer review only

33.3%						
	100.0%		100.0%	100.0%	100.0%	
33.3%						
	100.0%			100.0%	100.0%	
	100.0%			100.0%	100.0%	
30494	375207	21339	1616	329518	262194	391558
0	0	0	0	0	0	0
3	76	7	1	34	32	49
0	0	0	0	0	0	0
1	1	1	1	1	1	1
0	0	0	0	0	0	0
1	1	1	1	1	1	1
	1			1	1	1

For peer review only

1	1	1	1	1	1	1
0	0	0	0	0	0	0
30494	375207	21339	1616	329518	262194	391558
	1			1	1	1
	0			0	0	0
	375207			329518	262194	391558
100.0%	100.0%	100.0%	100.0%	100.0%	100.0%	100.0%
100.0%	100.0%	100.0%	100.0%	100.0%	100.0%	100.0%
	100.0%			100.0%	100.0%	100.0%

For peer review only

For peer review only

Arthritis Rese	Association f	Association f	Asthma and /	Asthma Relie	Asthma UK	Asyabi UK
6	13	6	1	1	34	1
3	1	5	0	0	34	0
1	1	1	1	1	1	1
1	1	1	0	0	1	0
1	1	1	1	1	1	1
1	1	1			1	
	1				1	
					1	
1	1	1	1	1		1
3	12	1	1	1		1
					1	
1	1	1				
3	12	1				
					1	
	1					
	12					

For peer review only

					0.0%	
		16.7%			0.0%	
		16.7%			0.0%	
			100.0%	100.0%		100.0%
					0.0%	
		16.7%			0.0%	
		16.7%			0.0%	
					0.0%	
					0.0%	
					0.0%	
248103	71616	35059	507	1014	301952	1500
0	0	20100	0	0	0	0
5	13	5	1	1	33	1
0	0	2	0	0	0	0
1	1	1	1	1	1	1
0	0	1	0	0	0	0
1	1	1	1	1	1	1
1					1	

For peer review only

1	1	1	1	1	1	1
0	0	0	0	0	0	0
248103	71616	14959	507	1014	301952	1500
1					1	
0					0	
248103					301952	
		42.7%				
100.0%	100.0%		100.0%	100.0%	100.0%	100.0%
		42.7%				
100.0%	100.0%		100.0%	100.0%	100.0%	100.0%
100.0%					100.0%	

For peer review only

Ataxia UK	Atrial Fibrilla	Back on Tracl	BackCare	Barts and Th	Basil Skyers I	Batten Disea
8	104	1	11	1	4	10
8	0	0	4	0	0	4
1	1	1	1	1	1	1
1	0	0	1	0	0	1
1	1	1	1	1	1	1
1	1		1		1	1
	1		1			
	1					
1						
	1	1	1	1	1	1
	104	1	7	1	4	6
1						
	1		1		1	1
	104		7		4	6
	1		1			
	104		7			
	1					

For peer review only

	104					
0.0%						
0.0%						
0.0%						
	100.0%	100.0%		100.0%	100.0%	
0.0%						
0.0%						
0.0%						
	100.0%				100.0%	
	100.0%					
	100.0%					
33556	621144	3809	43576	206	35201	73762
36402	0	0	9127	0	0	0
8	102	1	8	1	4	10
2	0	0	2	0	0	0
1	1	1	1	1	1	1
1	0	0	1	0	0	0
1	1	1	1	1	1	1
	1					

For peer review only

	1	1	1	1	1	1
1						
0	0	0	0	0	0	0
	621144	3809	34450	206	35201	73762
2846						
	1					
	0					
	621144					
7.8%						
7.8%						
7.8%						
	100.0%	100.0%		100.0%	100.0%	100.0%
7.8%						
7.8%						
7.8%						
	100.0%	100.0%		100.0%	100.0%	100.0%
	100.0%					

For peer review only

For peer review only

Beat Parkins	Beating Bowe	Behcets Synd	Bipolar Scotla	Bipolar UK	Birdshot Uve	Bladder and Bo
1	42	1	3	3	1	38
0	0	0	0	0	0	0
1	1	1	1	1	1	1
0	0	0	0	0	0	0
1	1	1	1	1	1	1
	1		1	1		1
	1					1
1	1	1	1	1	1	1
1	42	1	3	3	1	38
	1		1	1		1
	42		3	3		38
	1					1
	42					38

For peer review only

100.0%	100.0%	100.0%	100.0%	100.0%	100.0%	100.0%
	100.0%		100.0%	100.0%		100.0%
	100.0%					100.0%
5050	342673	20281	3194	4145	10000	2271217
0	0	0	0	0	0	0
1	42	1	3	3	1	38
0	0	0	0	0	0	0
1	1	1	1	1	1	1
0	0	0	0	0	0	0
1	1	1	1	1	1	1
	1					1
						1

For peer review only

1	1	1	1	1	1	1
0	0	0	0	0	0	0
5050	342673	20281	3194	4145	10000	2271217
	1					1
	0					0
	342673					2271217
						1
						0
						2271217
100.0%	100.0%	100.0%	100.0%	100.0%	100.0%	100.0%
100.0%	100.0%	100.0%	100.0%	100.0%	100.0%	100.0%
	100.0%					100.0%

For peer review only

100.0%

For peer review only

Bladder Heal	Bliss	Blood Pressu	Bloodwise	Bluebell Fou	Body and So	Bone Cancer
17	13	1	19	2	6	3
13	6	0	1	0	0	0
1	1	1	1	1	1	1
1	1	0	1	0	0	0
1	1	1	1	1	1	1
1	1		1	1	1	1
1	1		1			
1	1	1	1	1	1	1
4	7	1	18	2	6	3
1	1		1	1	1	1
4	7		18	2	6	3
1	1		1			
4	7		18			

For peer review only

23.5%						
		100.0%		100.0%	100.0%	100.0%
23.5%						
				100.0%	100.0%	100.0%
23.5%						
76682	204923	10521	197710	776	97946	12161
0	0	0	0	0	0	0
16	13	1	19	2	6	3
0	0	0	0	0	0	0
1	1	1	1	1	1	1
0	0	0	0	0	0	0
1	1	1	1	1	1	1
	1		1			

For peer review only

1	1	1	1	1	1	1
0	0	0	0	0	0	0
76682	204923	10521	197710	776	97946	12161
	1		1			
	0		0			
	204923		197710			
100.0%	100.0%	100.0%	100.0%	100.0%	100.0%	100.0%
100.0%	100.0%	100.0%	100.0%	100.0%	100.0%	100.0%
	100.0%		100.0%			

For peer review only

For peer review only

Bowel Cancer	Brain Charity	Brain Tumor	Brain Tumou	Breakthrougl	Breast Cance	Breast Cance
16	1	1	1	16	7	28
13	0	0	0	3	0	19
1	1	1	1	1	1	1
1	0	0	0	1	0	1
1	1	1	1	1	1	1
1				1	1	1
1				1		1
1	1	1	1	1	1	1
3	1	1	1	13	7	9
1				1	1	1
3				13	7	9
1				1		1
3				13		9

For peer review only

18.8%						
18.8%						32.1%
	100.0%	100.0%	100.0%		100.0%	
18.8%						
18.8%						32.1%
					100.0%	
18.8%						
18.8%						32.1%
109272	981	12676	262	109107	81316	332296
0	0	0	0	0	0	369592
16	1	1	1	16	6	25
0	0	0	0	0	0	14
1	1	1	1	1	1	1
0	0	0	0	0	0	1
1	1	1	1	1	1	1
1				1		1

For peer review only

1	1	1	1	1	1	
						1
0	0	0	0	0	0	0
109272	981	12676	262	109107	81316	
						37296
1				1		
						1
0				0		0
109272				109107		
						37296
						10.1%
						10.1%
100.0%	100.0%	100.0%	100.0%	100.0%	100.0%	
						10.1%
						10.1%
100.0%	100.0%	100.0%	100.0%	100.0%	100.0%	
						10.1%
						10.1%
100.0%				100.0%		

For peer review only

For peer review only

Breast Cance	Breast Cancer I	British Dupuy	British Heart	British Liver T	British Lung F	British Obesi
5	4	8	7	29	50	1
0	1	0	0	5	23	0
1	1	1	1	1	1	1
0	1	0	0	1	1	0
1	1	1	1	1	1	1
1	1	1	1	1	1	
				1	1	
1	1	1	1	1	1	1
5	3	8	7	24	27	1
1	1	1	1	1	1	
5	3	8	7	24	27	
				1	1	
				24	27	

For peer review only

100.0%		100.0%	100.0%			100.0%
100.0%		100.0%	100.0%			
47894	3506763	55592	1882	229800	574095	421
0	10000000	0	0	0	1139917	0
5	2	8	7	29	47	1
0	1	0	0	0	22	0
1	1	1	1	1	1	1
0	1	0	0	0	1	0
1	1	1	1	1	1	1
	1			1	1	
	1				1	

For peer review only

1		1	1	1		1
	1				1	
0	0	0	0	0	0	0
47894		55592	1882	229800		421
	6493237				565821	
				1		
	1				1	
	0			0	0	
				229800		
	6493237				565821	
	1				1	
	0				0	
	6493237				565821	
					49.6%	
100.0%		100.0%	100.0%	100.0%		100.0%
					49.6%	
100.0%		100.0%	100.0%	100.0%		100.0%
					49.6%	
				100.0%		

For peer review only

49.6%

For peer review only

British Polio	British Pregn	British Skin F	British Thyro	Brittle Bone	Brook	Building for t
1	5	6	1	1	12	1
0	2	0	5	2	0	0
1	1	1	1	1	1	1
0	1	0	1	1	0	0
1	1	1	1	1	1	1
	1	1	1	1	1	
					1	
1	1	1			1	1
			1	1		
1	3	6			12	1
			4	1		
	1	1			1	
			1	1		
	3	6			12	
			4	1		
					1	
					12	

For peer review only

100.0%		100.0%			100.0%	100.0%
		100.0%			100.0%	
					100.0%	
0	127999	9785	250	15428	95962	243
0	302001	0	44574	20000	0	0
0	5	6	1	1	12	1
0	2	0	4	1	0	0
0	1	1	1	1	1	1
0	1	0	1	1	0	0
	1	1	1	1	1	1
	1					

For peer review only

FALSE						
		1			1	1
	1		1	1		
	0	0	0	0	0	0
		9785			95962	243
	174002		44324	4572		
	1					
	0					
	174002					
				22.9%		
		100.0%			100.0%	100.0%
				22.9%		
		100.0%			100.0%	100.0%

For peer review only

For peer review only

Butterfly Thy	Cambridge C	Cambridge R	Cancer 52	Cancer Black	Cancer Focus	Cancer Resear
3	1	4	26	2	13	57
9	2	0	0	4	0	0
1	1	1	1	1	1	1
1	1	0	0	1	0	0
1	1	1	1	1	1	1
1	1	1	1	1	1	1
			1		1	1
		1	1		1	1
1	1			1		
		4	26		13	57
6	1			2		
		1	1		1	1
1	1			1		
		4	26		13	57
6	1			2		
			1		1	1
			26		13	57

For peer review only

		100.0%	100.0%		100.0%	100.0%
		100.0%	100.0%		100.0%	100.0%
			100.0%		100.0%	100.0%
32775	507	9320	249099	9615	29164	1817635
115983	610	0	0	14876	0	0
3	1	4	26	2	12	56
9	2	0	0	3	0	0
1	1	1	1	1	1	1
1	1	0	0	1	0	0
1	1	1	1	1	1	1
1			1			1
						1

For peer review only

		1	1		1	1
1	1			1		
0	0	0	0	0	0	0
		9320	249099		29164	1817635
83208	103			5260		
			1			1
1						
0			0			0
			249099			1817635
83208						
						1
						0
						1817635
	16.9%					
	16.9%			35.4%		
		100.0%	100.0%		100.0%	100.0%
	16.9%					
	16.9%			35.4%		
		100.0%	100.0%		100.0%	100.0%
			100.0%			100.0%

For peer review only

100.0%

For peer review only

Candlelighter	Cara Trust	Cardiac Risk i	Cardiomyopa	Catholics for	Centre for Al	Changing Fac
1	5	7	5	1	1	8
0	5	0	0	0	4	3
1	1	1	1	1	1	1
0	1	0	0	0	1	1
1	1	1	1	1	1	1
	1	1	1		1	1
	1					
1		1	1	1		1
					1	
1		7	5	1		5
					3	
	1					
		1	1			1
					1	
		7	5			5
					3	

For peer review only

	0.0%					
	0.0%					
	0.0%					
100.0%		100.0%	100.0%	100.0%		
	0.0%					
	0.0%					
	0.0%					
		100.0%	100.0%			
0	15570	6271	2338	4089	253	32753
0	20523	0	0	0	303	10000
0	5	7	5	1	1	8
0	4	0	0	0	1	1
0	1	1	1	1	1	1
0	1	0	0	0	1	1
	1	1	1	1	1	1

For peer review only

FALSE						
		1	1	1		1
	1				1	
	0	0	0	0	0	0
		6271	2338	4089		22753
	4953				51	
					16.7%	
	24.1%				16.7%	
		100.0%	100.0%	100.0%		
					16.7%	
	24.1%				16.7%	
		100.0%	100.0%	100.0%		

For peer review only

For peer review only

Charlie Walle	Child Growth	Children Livir	Children with	Children with	Children's Bo	Children's Liv
1	9	9	1	2	4	2
0	3	14	1	0	9	0
1	1	1	1	1	1	1
0	1	1	1	0	1	0
1	1	1	1	1	1	1
	1	1		1	1	1
		1				
			1			
1	1			1		1
		1			1	
1	6			2		2
		5			5	
	1			1		1
		1			1	
	6			2		2
		5			5	
		1				
		5				

For peer review only

			0.0%			
			0.0%			
		35.7%	0.0%			
100.0%				100.0%		100.0%
		35.7%				
				100.0%		100.0%
		35.7%				
1000	14236	31845	20180	625	33966	16010
0	0	23564	20099	0	32665	0
1	9	9	1	2	4	2
0	0	8	1	0	2	0
1	1	1	1	1	1	1
0	0	1	1	0	1	0
1	1	1	1	1	1	1

For peer review only

1	1	1	1	1	1	1
0	0	0	0	0	0	0
1000	14236	8282	81	625	1300	16010
			0.4%		3.8%	
			0.4%		3.8%	
		26.0%	0.4%		3.8%	
100.0%	100.0%			100.0%		100.0%
			0.4%		3.8%	
			0.4%		3.8%	
		26.0%	0.4%		3.8%	
100.0%	100.0%			100.0%		100.0%

For peer review only

For peer review only

Children's He	Children's HI	Chinese Nati	Chronic Lymp	Chronic Myel	Cleft Lip and	CLIC Sargent
1	10	1	12	26	2	3
0	13	0	0	16	0	0
1	1	1	1	1	1	1
0	1	0	0	1	0	0
1	1	1	1	1	1	1
	1		1	1	1	1
	1		1	1		
1		1	1	1	1	1
	1					
1		1	12	10	2	3
	3					
			1	1	1	1
	1					
			12	10	2	3
	3					
			1	1		
	1					
			12	10		
	3					

For peer review only

	23.1%			38.5%		
100.0%		100.0%	100.0%		100.0%	100.0%
	23.1%			38.5%		
			100.0%		100.0%	100.0%
	23.1%			38.5%		
			100.0%			
736	49078	253	86711	180581	121	5412
0	0	0	0	185184	0	0
1	10	1	12	25	2	3
0	0	0	0	16	0	0
1	1	1	1	1	1	1
0	0	0	0	1	0	0
1	1	1	1	1	1	1
				1		

For peer review only

For peer review only

1	1	1	1		1	1
				1		
0	0	0	0	0	0	0
736	49078	253	86711		121	5412
				4603		
				1		
				0		
				4603		
				2.5%		
				2.5%		
				2.5%		
100.0%	100.0%	100.0%	100.0%		100.0%	100.0%
				2.5%		
				2.5%		
				2.5%		
100.0%	100.0%	100.0%	100.0%		100.0%	100.0%
				2.5%		
				2.5%		
				2.5%		

For peer review only

Coeliac UK	Community (	Community I	Community L	Confederatio	Contact a Far	Coping with (
6	1	1	1	2	3	1
2	0	1	2	2	0	0
1	1	1	1	1	1	1
1	0	1	1	1	0	0
1	1	1	1	1	1	1
1			1	1	1	
		1		1		
1	1				1	1
			1			
4	1				3	1
			1			
				1		
1					1	
			1			
4					3	
			1			

For peer review only

		0.0%		0.0%		
		0.0%		0.0%		
		0.0%		0.0%		
	100.0%				100.0%	100.0%
				0.0%		
				0.0%		
				0.0%		
					100.0%	
33029	1029	3042	6060	72000	20369	4187
32003	0	3042	10051	0	0	0
6	1	1	1	2	3	1
2	0	1	2	0	0	0
1	1	1	1	1	1	1
1	0	1	1	0	0	0
1	1	1	1	1	1	1

For peer review only

		1				
1	1			1	1	1
			1			
0	0	0	0	0	0	0
1027	1029			72000	20369	4187
			3991			
3.1%		0.0%				
3.1%		0.0%				
3.1%		0.0%	39.7%			
	100.0%			100.0%	100.0%	100.0%
3.1%		0.0%				
3.1%		0.0%				
3.1%		0.0%	39.7%			
	100.0%			100.0%	100.0%	100.0%

For peer review only

For peer review only

Coppafeel!	Core Charity	Crohn's and (	Crohn's in Ch	Cure and Act	Cure Parkins	Cystic Fibrosi
1	5	49	9	2	9	17
1	3	35	0	1	0	0
1	1	1	1	1	1	1
1	1	1	0	1	0	0
1	1	1	1	1	1	1
	1	1	1	1	1	1
		1				1
1						
	1	1	1	1	1	1
	2	14	9	1	9	17
	1	1	1	1	1	1
	2	14	9	1	9	17
		1				1
		14				17

For peer review only

0.0%						
0.0%						
0.0%	40.0%	28.6%				
			100.0%		100.0%	100.0%
	40.0%	28.6%				
			100.0%		100.0%	100.0%
		28.6%				
						100.0%
10000	44609	593275	27581	120	24749	281197
10100	0	0	0	9447	0	0
1	5	49	9	2	9	17
1	0	0	0	1	0	0
1	1	1	1	1	1	1
1	0	0	0	1	0	0
1	1	1	1	1	1	1
		1				1

For peer review only

	1	1	1		1	1
1				1		
0	0	0	0	0	0	0
	44609	593275	27581		24749	281197
100				9327		
		1				1
		0				0
		593275				281197
1.0%						
1.0%						
1.0%						
	100.0%	100.0%	100.0%		100.0%	100.0%
1.0%						
1.0%						
1.0%						
	100.0%	100.0%	100.0%		100.0%	100.0%
		100.0%				100.0%

For peer review only

For peer review only

David Lewis (	Deaf Educati	Delete Blood	Dementia UK	Dermatrust	Diabetes Res	Diabetes UK
1	1	3	1	1	3	210
0	0	0	0	2	0	11
1	1	1	1	1	1	1
0	0	0	0	1	0	1
1	1	1	1	1	1	1
		1		1	1	1
						1
						1
1	1	1	1		1	1
				1		
1	1	3	1		3	199
				1		
		1			1	1
				1		
		3			3	199
				1		
						1
						199
						1

For peer review only

For peer review only

						199
100.0%	100.0%	100.0%	100.0%		100.0%	
		100.0%			100.0%	
1892	526	14665	2593	29823	6595	3050180
0	0	0	0	45823	0	0
1	1	3	1	1	3	209
0	0	0	0	2	0	0
1	1	1	1	1	1	1
0	0	0	0	1	0	0
1	1	1	1	1	1	1
						1
						1

1	1	1	1		1	1
				1		
0	0	0	0	0	0	0
1892	526	14665	2593		6595	3050180
				16000		
						1
						0
						3050180
						1
						0
						3050180
				34.9%		
100.0%	100.0%	100.0%	100.0%		100.0%	100.0%
				34.9%		
100.0%	100.0%	100.0%	100.0%		100.0%	100.0%
						100.0%

For peer review only

100.0%

For peer review only

Diabetics wit	Diamond Bla	Different Stre	Disabled Livi	Douglas Bad	Edinburgh an	Education fo
4	1	5	6	2	2	3
0	0	0	2	0	0	8
1	1	1	1	1	1	1
0	0	0	1	0	0	1
1	1	1	1	1	1	1
1		1	1	1	1	1
1	1	1	1	1	1	
						1
4	1	5	4	2	2	
						5
1		1	1	1	1	
						1
4		5	4	2	2	
						5

For peer review only

100.0%	100.0%	100.0%		100.0%	100.0%	
100.0%		100.0%		100.0%	100.0%	
1547	15150	10787	23840	1384	804	5343
0	0	0	0	0	0	45921
4	1	5	6	2	2	3
0	0	0	0	0	0	3
1	1	1	1	1	1	1
0	0	0	0	0	0	1
1	1	1	1	1	1	1

For peer review only

1	1	1	1	1	1	
						1
0	0	0	0	0	0	0
1547	15150	10787	23840	1384	804	
						40578
100.0%	100.0%	100.0%	100.0%	100.0%	100.0%	
100.0%	100.0%	100.0%	100.0%	100.0%	100.0%	

For peer review only

For peer review only

Encephalitis S	Endometrios	Epilepsy Acti	Epilepsy Con	Epilepsy Scot	Epilepsy Soci	European Pa
2	2	9	2	7	18	4
0	5	7	0	0	15	11
1	1	1	1	1	1	1
0	1	1	0	0	1	1
1	1	1	1	1	1	1
1	1	1	1	1	1	1
					1	1
1		1	1	1	1	
	1					1
2		2	2	7	3	
	3					7
1		1	1	1	1	
	1					1
2		2	2	7	3	
	3					7
					1	
						1
					3	
						7

For peer review only

					16.7%	
		22.2%			16.7%	
100.0%			100.0%	100.0%		
					16.7%	
		22.2%			16.7%	
100.0%			100.0%	100.0%		
					16.7%	
					16.7%	
767	21096	92855	4085	6117	188332	64681
0	21096	199382	0	0	111712	239475
2	2	9	2	7	18	4
0	2	6	0	0	3	7
1	1	1	1	1	1	1
0	1	1	0	0	1	1
1	1	1	1	1	1	1
		1			1	1

For peer review only

	1					
1			1	1	1	
		1				1
0	0	0	0	0	0	0
767			4085	6117	76620	
		106527				174794
					1	
		1				1
		0			0	0
					76620	
		106527				174794
	0.0%					
	0.0%					
	0.0%				40.7%	
100.0%			100.0%	100.0%		
	0.0%					
	0.0%					
	0.0%				40.7%	
100.0%			100.0%	100.0%		
					40.7%	

For peer review only

For peer review only - http://bmjopen.bmj.com/site/about/guidelines.xhtml

For peer review only

Family Plann	Fertility Netw	Findacure	Food Chain	FORCE	Forum Link	Foundation f
12	17	6	3	1	6	1
1	17	7	1	0	0	1
1	1	1	1	1	1	1
1	1	1	1	0	0	1
1	1	1	1	1	1	1
1	1	1	1		1	
1	1					
	1					1
1			1	1	1	
		1				
11			2	1	6	
		1				
	1					
1			1		1	
		1				
11			2		6	
		1				
	1					
1						
11						

For peer review only

	0.0%					0.0%
	0.0%	14.3%				0.0%
	0.0%	14.3%				0.0%
				100.0%	100.0%	
	0.0%					
	0.0%	14.3%				
	0.0%	14.3%				
					100.0%	
	0.0%					
	0.0%					
	0.0%					
107247	189815	27591	13092	507	26164	57537
15150	56317	46176	5000	0	0	0
11	17	6	3	1	6	1
1	5	7	1	0	0	0
1	1	1	1	1	1	1
1	1	1	1	0	0	0
1	1	1	1	1	1	1
1	1					

For peer review only

1	1		1	1	1	1
		1				
0	0	0	0	0	0	0
92097	133497		8092	507	26164	57537
		18585				
1	1					
0	0					
92097	133497					
		40.2%				
				100.0%	100.0%	100.0%
		40.2%				
				100.0%	100.0%	100.0%

For peer review only

For peer review only

Freshwinds	Friends of FO	Fungal Infect	Gauchers Ass	Gay Men's H	Genetic Allia	George Hous
2	1	8	29	13	67	2
0	1	11	6	3	86	1
1	1	1	1	1	1	1
0	1	1	1	1	1	1
1	1	1	1	1	1	1
1		1	1	1	1	1
		1	1	1	1	
	1					
1			1	1		1
		1			1	
2			23	10		1
		3			19	
1			1	1		1
		1			1	
2			23	10		1
		3			19	
			1	1		
		1			1	
			23	10		
		3			19	

For peer review only

	0.0%					
	0.0%					
	0.0%	27.3%			22.1%	
100.0%						
		27.3%			22.1%	
100.0%						
		27.3%			22.1%	
11930	0	264961	182227	93592	616355	21000
0	0	307187	0	69319	235212	1000
2	0	8	29	13	66	2
0	0	10	0	3	12	1
1	0	1	1	1	1	1
0	0	1	0	1	1	1
1		1	1	1	1	1
		1	1		1	

For peer review only

	FALSE					
1			1	1	1	1
		1				
0		0	0	0	0	0
11930			182227	24273	381142	20000
		42226				
			1		1	
		1				
		0	0		0	
			182227		381142	
		42226				
		13.7%				
		13.7%		25.9%		
100.0%			100.0%			
		13.7%				
		13.7%		25.9%		
100.0%			100.0%			
		13.7%				
		13.7%				
			100.0%			

For peer review only

For peer review only

GIST Support	Groundswell	Group B Stre	Guillain Barr	Haemophilia	Haemophilia	Haemophilia
21	1	1	2	17	57	10
6	1	0	0	2	0	0
1	1	1	1	1	1	1
1	1	0	0	1	0	0
1	1	1	1	1	1	1
1			1	1	1	1
1				1	1	
	1					
1		1	1	1	1	1
15		1	2	15	57	10
1			1	1	1	1
15			2	15	57	10
1				1	1	
15				15	57	

For peer review only

	0.0%					
	0.0%					
	0.0%					
		100.0%	100.0%		100.0%	100.0%
			100.0%		100.0%	100.0%
					100.0%	
103706	3042	154	6400	47143	577522	13721
53906	0	0	0	25605	0	0
21	1	1	2	16	56	10
6	0	0	0	2	0	0
1	1	1	1	1	1	1
1	0	0	0	1	0	0
1	1	1	1	1	1	1
1					1	

For peer review only

1	1	1	1	1	1	1
0	0	0	0	0	0	0
49800	3042	154	6400	21538	577522	13721
1					1	
0					0	
49800					577522	
48.0%				45.7%		
	100.0%	100.0%	100.0%		100.0%	100.0%
48.0%				45.7%		
	100.0%	100.0%	100.0%		100.0%	100.0%
48.0%						
					100.0%	

For peer review only

For peer review only

Hafal	Headway	Health and S	Heart of Mer	Heart UK	Heartbeat	Hepatitis B P
1	1	1	2	94	1	1
0	0	0	2	5	0	0
1	1	1	1	1	1	1
0	0	0	1	1	0	0
1	1	1	1	1	1	1
			1	1		
				1		
			1			
1	1	1		1	1	1
1	1	1		89	1	1
			1			
				1		
				89		
				1		
				89		

For peer review only

			0.0%			
			0.0%			
			0.0%			
100.0%	100.0%	100.0%			100.0%	100.0%
			0.0%			
			0.0%			
			0.0%			
4000	150	38	31563	909013	758	5070
0	0	0	15781	0	0	0
1	1	1	2	90	1	1
0	0	0	1	0	0	0
1	1	1	1	1	1	1
0	0	0	1	0	0	0
1	1	1	1	1	1	1
				1		

For peer review only

1	1	1	1	1	1	1
0	0	0	0	0	0	0
4000	150	38	15781	909013	758	5070
				1		
				0		
				909013		
100.0%	100.0%	100.0%		100.0%	100.0%	100.0%
100.0%	100.0%	100.0%		100.0%	100.0%	100.0%
				100.0%		

For peer review only

For peer review only

Hepatitis C Tr	Hereditary A	Herts Aid	Hidradenitis	HIV i-Base	HIV Scotland	Hughes Synd
84	15	4	7	13	3	1
25	6	2	2	0	3	0
1	1	1	1	1	1	1
1	1	1	1	0	1	0
1	1	1	1	1	1	1
1	1	1	1	1	1	
1	1			1		
					1	
1	1	1	1	1		1
59	9	2	5	13		1
					1	
1	1	1	1	1		
59	9	2	5	13		
1	1			1		
59	9			13		

For peer review only

					0.0%	
					0.0%	
					0.0%	
				100.0%		100.0%
					0.0%	
					0.0%	
					0.0%	
				100.0%		
				100.0%		
1022603	200669	11529	32910	63462	23200	2104
1566080	194617	8050	25750	0	20509	0
83	15	4	7	13	3	1
25	6	2	2	0	3	0
1	1	1	1	1	1	1
1	1	1	1	0	1	0
1	1	1	1	1	1	1
1	1					
1						

For peer review only

	1	1	1	1	1	1
1						
0	0	0	0	0	0	0
	6051	3479	7160	63462	2691	2104
543477						
	1					
1						
0	0					
	6051					
543477						
1						
0						
543477						
	3.0%					
	3.0%				11.6%	
34.7%	3.0%	30.2%	21.8%		11.6%	
				100.0%		100.0%
	3.0%					
	3.0%				11.6%	
34.7%	3.0%	30.2%	21.8%		11.6%	
				100.0%		100.0%
	3.0%					
	3.0%					
34.7%	3.0%					

34.7%

For peer review only

Huntington's	IBS Network	ICUsteps	Immune Thr	Imperial Coll	Independent	Input Diabet
1	1	2	15	1	11	1
3	4	0	29	0	0	0
1	1	1	1	1	1	1
1	1	0	1	0	0	0
1	1	1	1	1	1	1
1	1	1	1		1	
			1		1	
		1		1	1	1
1	1		1			
		2		1	11	1
2	3		14			
		1			1	
1	1		1			
		2			11	
2	3		14			
					1	
			1			
					11	
			14			

For peer review only

			48.3%			
		100.0%		100.0%	100.0%	100.0%
			48.3%			
		100.0%			100.0%	
			48.3%			
					100.0%	
30159	8417	4157	67352	4000	29891	30322
0	0	0	44323	0	0	0
1	1	2	15	1	11	1
0	0	0	8	0	0	0
1	1	1	1	1	1	1
0	0	0	1	0	0	0
1	1	1	1	1	1	1

For peer review only

1	1	1	1	1	1	1
0	0	0	0	0	0	0
30159	8417	4157	23028	4000	29891	30322
			34.2%			
100.0%	100.0%	100.0%		100.0%	100.0%	100.0%
			34.2%			
100.0%	100.0%	100.0%		100.0%	100.0%	100.0%

For peer review only

For peer review only

International	International	International	International	International	Jo's Cervical (	Joint Epilepsy
23	2	9	1	10	2	1
39	1	19	8	31	14	4
1	1	1	1	1	1	1
1	1	1	1	1	1	1
1	1	1	1	1	1	1
1	1	1	1	1	1	1
1		1		1	1	
	1					
1		1	1	1	1	1
	1					
16		10	7	21	12	3
	1					
1		1	1	1	1	1
	1					
16		10	7	21	12	3
1		1		1	1	
16		10		21	12	

For peer review only

41.0%						
41.0%						
41.0%						
652377	73355	24458	18514	222272	25150	12856
873965	0	0	0	1681140	36360	0
23	2	9	1	10	2	1
39	0	0	0	31	2	0
1	1	1	1	1	1	1
1	0	0	0	1	1	0
1	1	1	1	1	1	1
1				1		
				1		

For peer review only

	1	1	1			1
1				1	1	
0	0	0	0	0	0	0
	73355	24458	18514			12856
221588				1458868	11210	
1				1		
0				0		
221588				1458868		
				1		
				0		
				1458868		
25.4%					30.8%	
	100.0%	100.0%	100.0%			100.0%
25.4%					30.8%	
	100.0%	100.0%	100.0%			100.0%
25.4%						

For peer review only

For peer review only

				Patient organisation names		
**Juvenile Diab**	**KAITY**	**Karen Cliffor**	**Kent Associa**	**Kidney Cance**	**Kidney Cance**	**Kidney Care U**
19	1	10	1	9	37	2
6	0	0	2	0	0	0
1	1	1	1	1	1	1
1	0	0	1	0	0	0
1	1	1	1	1	1	1
1		1	1	1	1	1
1					1	
1	1	1		1	1	1
			1			
13	1	10		9	37	2
			1			
1		1		1	1	1
			1			
13		10		9	37	2
			1			
1					1	
13					37	

For peer review only

	100.0%	100.0%		100.0%	100.0%	100.0%
		100.0%		100.0%	100.0%	100.0%
					100.0%	
417065	2000	91665	18513	101336	183714	754
10563	0	0	18938	0	0	0
19	1	9	1	8	35	2
1	0	0	1	0	0	0
1	1	1	1	1	1	1
1	0	0	1	0	0	0
1	1	1	1	1	1	1
1				1	1	

For peer review only

1	1	1		1	1	1
			1			
0	0	0	0	0	0	0
406503	2000	91665		101336	183714	754
			424			
1				1	1	
0				0	0	
406503				101336	183714	
			2.2%			
			2.2%			
			2.2%			
	100.0%	100.0%		100.0%	100.0%	100.0%
			2.2%			
			2.2%			
			2.2%			
	100.0%	100.0%		100.0%	100.0%	100.0%
				100.0%	100.0%	

For peer review only

For peer review only

Kidney Resea	KIDS	KwaAfrica	LATCH Welsh	Laurence Mo	Leeds Teachi	Leicestershir
17	1	1	1	1	1	4
47	1	1	0	1	0	0
1	1	1	1	1	1	1
1	1	1	0	1	0	0
1	1	1	1	1	1	1
1						1
1						
	1	1		1		
			1		1	1
1						
			1		1	4
30						
						1
1						
						4
30						
1						
30						

For peer review only

	0.0%	0.0%		0.0%		
	0.0%	0.0%		0.0%		
	0.0%	0.0%		0.0%		
			100.0%		100.0%	100.0%
						100.0%
178461	7676	5000	1010	1010	354	22216
3144648	7676	5000	0	0	0	0
17	1	1	1	1	1	4
47	1	1	0	0	0	0
1	1	1	1	1	1	1
1	1	1	0	0	0	0
1	1	1	1	1	1	1
1						
1						

For peer review only

	1	1				
			1	1	1	1
1						
0	0	0	0	0	0	0
			1010	1010	354	22216
2966187						
1						
0						
2966187						
1						
0						
2966187						
	0.0%	0.0%				
	0.0%	0.0%				
	0.0%	0.0%				
			100.0%	100.0%	100.0%	100.0%
	0.0%	0.0%				
	0.0%	0.0%				
	0.0%	0.0%				
			100.0%	100.0%	100.0%	100.0%

For peer review only

For peer review only

Leukaemia C	Life Educatio	Little Hearts	Liver4Life	Lullaby Trust	Lupus Europ	Lupus UK
62	1	1	24	1	4	1
39	0	0	0	0	0	0
1	1	1	1	1	1	1
1	0	0	0	0	0	0
1	1	1	1	1	1	1
1			1		1	
1			1			
1	1	1	1	1	1	1
23	1	1	24	1	4	1
1			1		1	
23			24		4	
1			1			
23			24			

For peer review only

37.1%						
	100.0%	100.0%	100.0%	100.0%	100.0%	100.0%
37.1%						
			100.0%		100.0%	
37.1%						
			100.0%			
472981	519	253	248299	0	55503	707
351393	0	0	0	0	0	0
61	1	1	24	0	4	1
30	0	0	0	0	0	0
1	1	1	1	0	1	1
1	0	0	0	0	0	0
1	1	1	1		1	1
1			1			

For peer review only

				FALSE		
1	1	1	1		1	1
0	0	0	0		0	0
121588	519	253	248299		55503	707
1			1			
0			0			
121588			248299			
25.7%						
	100.0%	100.0%	100.0%		100.0%	100.0%
25.7%						
	100.0%	100.0%	100.0%		100.0%	100.0%
25.7%						
			100.0%			

For peer review only

For peer review only

Lymphoma A	Macmillan Ca	Macular Soci	Maggie's Cen	Make A Wish	Malnutrition	MAMA Acad
58	50	1	3	2	1	1
84	2	0	0	0	0	0
1	1	1	1	1	1	1
1	1	0	0	0	0	0
1	1	1	1	1	1	1
1	1		1	1		
1	1					
	1	1	1	1	1	1
1						
	48	1	3	2	1	1
26						
	1		1	1		
1						
	48		3	2		
26						
	1					
1						
	48					
26						

For peer review only

31.0%						
		100.0%	100.0%	100.0%	100.0%	100.0%
31.0%						
			100.0%	100.0%		
31.0%						
158813	190660	15	3413	765	884	202
31792	0	0	0	0	0	0
57	50	1	3	2	1	1
9	0	0	0	0	0	0
1	1	1	1	1	1	1
1	0	0	0	0	0	0
1	1	1	1	1	1	1
1	1					

For peer review only

1	1	1	1	1	1	1
0	0	0	0	0	0	0
127022	190660	15	3413	765	884	202
1	1					
0	0					
127022	190660					
	100.0%	100.0%	100.0%	100.0%	100.0%	100.0%
	100.0%	100.0%	100.0%	100.0%	100.0%	100.0%
	100.0%					

For peer review only

For peer review only

Manchester /	Marie Curie (	Martin Fishe	MDS UK Sup	Medical Four	Melanoma F	Melanoma F
3	3	1	13	5	4	3
2	0	0	6	3	1	1
1	1	1	1	1	1	1
1	0	0	1	1	1	1
1	1	1	1	1	1	1
1	1		1	1	1	1
			1			
1	1	1	1	1	1	1
1	3	1	7	2	3	2
1	1		1	1	1	1
1	3		7	2	3	2
			1			
			7			

For peer review only

33.3%				40.0%		
	100.0%	100.0%				
33.3%				40.0%		
	100.0%					
19538	643	15000	145332	58641	61418	23080
9438	0	0	131702	63480	0	5050
3	3	1	13	5	4	3
2	0	0	6	3	0	1
1	1	1	1	1	1	1
1	0	0	1	1	0	1
1	1	1	1	1	1	1
			1			

For peer review only

1	1	1	1		1	1
				1		
0	0	0	0	0	0	0
10100	643	15000	13630		61418	18030
				4840		
			1			
			0			
			13630			
			9.4%	7.6%		
			9.4%	7.6%		
			9.4%	7.6%		
	100.0%	100.0%			100.0%	
			9.4%	7.6%		
			9.4%	7.6%		
			9.4%	7.6%		
	100.0%	100.0%			100.0%	
			9.4%			
			9.4%			
			9.4%			

For peer review only

For peer review only

Melanoma U	Men's Healtl	Meningitis N	Meningitis Re	Mental Healt	Mesotheliom	Migraine Acti
24	14	33	60	8	7	8
0	5	20	13	1	0	1
1	1	1	1	1	1	1
0	1	1	1	1	0	1
1	1	1	1	1	1	1
1	1	1	1	1	1	1
1	1	1	1			
1	1	1	1	1	1	1
24	9	13	47	7	7	7
1	1	1	1	1	1	1
24	9	13	47	7	7	7
1	1	1	1			
24	9	13	47			

For peer review only

		39.4%				
100.0%					100.0%	
		39.4%				
100.0%					100.0%	
		39.4%				
100.0%						
386833	94419	532482	700445	168898	34571	107846
0	64124	266721	216480	5143	0	0
24	11	32	59	8	7	8
0	5	16	8	1	0	0
1	1	1	1	1	1	1
0	1	1	1	1	0	0
1	1	1	1	1	1	1
1		1	1	1		1

For peer review only

1	1	1	1	1	1	1
0	0	0	0	0	0	0
386833	30294	265761	483965	163756	34571	107846
1		1	1	1		1
0		0	0	0		0
386833		265761	483965	163756		107846
	32.1%	49.9%				
100.0%					100.0%	100.0%
	32.1%	49.9%				
100.0%					100.0%	100.0%
		49.9%				
100.0%						100.0%

For peer review only

For peer review only

Migraine Tru	Motor Neuro	Mouth Cance	Movember F	MPN Voice	Multi Organ	Multiple Birt
9	2	1	1	6	1	1
9	0	2	0	0	0	0
1	1	1	1	1	1	1
1	0	1	0	0	0	0
1	1	1	1	1	1	1
1	1	1		1		
1						
	1		1	1	1	1
		1				
	2		1	6	1	1
		1				
1						
	1			1		
		1				
	2			6		
		1				

For peer review only

0.0%						
0.0%						
0.0%						
	100.0%		100.0%	100.0%	100.0%	100.0%
0.0%						
0.0%						
0.0%						
	100.0%			100.0%		
308512	280	1000	1010	44237	6084	505
0	0	0	0	0	0	0
9	2	1	1	6	1	1
0	0	0	0	0	0	0
1	1	1	1	1	1	1
0	0	0	0	0	0	0
1	1	1	1	1	1	1
1						

For peer review only

1	1	1	1	1	1	1
0	0	0	0	0	0	0
308512	280	1000	1010	44237	6084	505
1						
0						
308512						
100.0%	100.0%	100.0%	100.0%	100.0%	100.0%	100.0%
100.0%	100.0%	100.0%	100.0%	100.0%	100.0%	100.0%
100.0%						

For peer review only

For peer review only

Multiple Scle	Multiple Scle	Multiple Scle	Multiple Scle	Muscular Dy	Myasthenia (	Myeloma UK
7	38	62	1	3	1	53
28	24	18	0	10	0	15
1	1	1	1	1	1	1
1	1	1	0	1	0	1
1	1	1	1	1	1	1
1	1	1		1		1
1	1	1				1
	1	1	1		1	1
1				1		
	14	44	1		1	38
21				7		
	1	1				1
1				1		
	14	44				38
21				7		
	1	1				1
1						
	14	44				38
21						

For peer review only

	36.8%					
			100.0%		100.0%	
	36.8%					
	36.8%					
317100	282676	533370	603	17028	2020	7430106
2524665	564884	55200	0	0	0	711530
7	38	62	1	3	1	52
28	24	5	0	0	0	15
1	1	1	1	1	1	1
1	1	1	0	0	0	1
1	1	1	1	1	1	1
1	1	1				1
1						1

For peer review only

		1	1	1	1	1
1	1					
0	0	0	0	0	0	0
		478170	603	17028	2020	6718577
2207565	282208					
		1				1
1	1					
0	0	0				0
		478170				6718577
2207565	282208					
						1
1						
0						0
						6718577
2207565						
	50.0%					
			100.0%	100.0%	100.0%	
	50.0%					
			100.0%	100.0%	100.0%	
	50.0%					

For peer review only

For peer review only

Myrovlytis T	NARA - The E	National AID	National AID	National Ank	National Ass	National Att
1	1	41	28	55	1	9
0	0	7	17	33	0	7
1	1	1	1	1	1	1
0	0	1	1	1	0	1
1	1	1	1	1	1	1
		1	1	1		1
		1	1	1		
1	1	1	1	1	1	1
1	1	34	11	22	1	2
		1	1	1		1
		34	11	22		2
		1	1	1		
		34	11	22		

For peer review only

			39.3%	40.0%		22.2%
100.0%	100.0%				100.0%	
			39.3%	40.0%		22.2%
			39.3%	40.0%		
1420	0	454331	284499	902728	152	12006
0	0	198306	316593	527875	0	27951
1	0	41	28	54	1	9
0	0	6	17	18	0	7
1	0	1	1	1	1	1
0	0	1	1	1	0	1
1		1	1	1	1	1
		1	1	1		

For peer review only

	FALSE					
1		1		1	1	
			1			1
0		0	0	0	0	0
1420		256025		374853	152	
			32094			15945
		1		1		
			1			
		0	0	0		
		256025		374853		
			32094			
			10.1%			
			10.1%	41.5%		
100.0%					100.0%	
			10.1%			
			10.1%	41.5%		
100.0%					100.0%	
			10.1%			
			10.1%	41.5%		

For peer review only

For peer review only

National Can	National Chil	National Cou	National Eczє	National Kidr	National Obє	National Ostє
1	1	1	14	30	3	35
0	1	3	0	0	0	0
1	1	1	1	1	1	1
0	1	1	0	0	0	0
1	1	1	1	1	1	1
		1	1	1	1	1
			1	1		1
	1					
1			1	1	1	1
		1				
1			14	30	3	35
		2				
			1	1	1	1
		1				
			14	30	3	35
		2				
			1	1		1
			14	30		35

For peer review only

	0.0%					
	0.0%					
	0.0%					
100.0%			100.0%	100.0%	100.0%	100.0%
			100.0%	100.0%	100.0%	100.0%
			100.0%	100.0%		100.0%
1000	9600	10521	23676	396718	3508	326741
0	0	23780	0	0	0	0
1	1	1	14	30	3	34
0	0	3	0	0	0	0
1	1	1	1	1	1	1
0	0	1	0	0	0	0
1	1	1	1	1	1	1
				1		1

For peer review only

1	1		1	1	1	1
		1				
0	0	0	0	0	0	0
1000	9600		23676	396718	3508	326741
		13259				
				1		1
				0		0
				396718		326741
100.0%	100.0%		100.0%	100.0%	100.0%	100.0%
100.0%	100.0%		100.0%	100.0%	100.0%	100.0%
				100.0%		100.0%

For peer review only

For peer review only

National Rhe	National Voi	NAZ	NET Patient I	Neurological	Niemann-Pic	NMO Resear
102	12	10	28	15	12	3
20	21	4	14	31	14	0
1	1	1	1	1	1	1
1	1	1	1	1	1	0
1	1	1	1	1	1	1
1	1	1	1	1	1	1
1	1		1	1	1	
1						
1		1	1			1
	1			1	1	
82		6	14			3
	9			16	2	
1		1	1			1
	1			1	1	
82		6	14			3
	9			16	2	
1			1			
	1			1	1	
82			14			
	9			16	2	
1						

For peer review only

82						
					14.3%	
	42.9%				14.3%	
						100.0%
					14.3%	
	42.9%				14.3%	
						100.0%
					14.3%	
	42.9%				14.3%	
688957	87459	105845	196851	147434	129795	6650
0	212771	133957	269645	0	102765	0
100	12	10	27	15	12	3
0	15	4	14	0	12	0
1	1	1	1	1	1	1
0	1	1	1	0	1	0
1	1	1	1	1	1	1
1	1	1	1	1	1	

For peer review only

1				1	1	1
	1	1	1			
0	0	0	0	0	0	0
688957				147434	27030	6650
	125313	28112	72795			
1				1	1	
	1	1	1			
0	0	0	0	0	0	
688957				147434	27030	
	125313	28112	72795			
		21.0%	27.0%		20.8%	
100.0%				100.0%		100.0%
		21.0%	27.0%		20.8%	
100.0%				100.0%		100.0%
		21.0%	27.0%		20.8%	
100.0%				100.0%		

For peer review only

For peer review only

North of Engl	Northern Irel	Northern Irel	Northern Ne	OcuMel UK	Oesophageal	Older People
4	1	2	2	2	1	2
0	0	0	1	0	1	1
1	1	1	1	1	1	1
0	0	0	1	0	1	1
1	1	1	1	1	1	1
1		1	1	1		1
					1	
1	1	1	1	1		1
4	1	2	1	2		1
1		1	1	1		1
4		2	1	2		1

For peer review only

					0.0%	
					0.0%	
					0.0%	
100.0%	100.0%	100.0%		100.0%		
100.0%		100.0%		100.0%		
5775	1029	4035	21913	2081	1636	30300
0	0	0	21913	0	0	25351
4	1	2	1	2	1	2
0	0	0	1	0	0	1
1	1	1	1	1	1	1
0	0	0	1	0	0	1
1	1	1	1	1	1	1

For peer review only

			1			
1	1	1		1	1	1
0	0	0	0	0	0	0
5775	1029	4035		2081	1636	4949
			0.0%			
			0.0%			16.3%
			0.0%			16.3%
100.0%	100.0%	100.0%		100.0%	100.0%	
			0.0%			
			0.0%			16.3%
			0.0%			16.3%
100.0%	100.0%	100.0%		100.0%	100.0%	

For peer review only

Olive Tree Ca	Oliver King Fo	Orchid	Organisation	Ovacome	Ovarian Canc	Paget's Assoc
1	1	2	2	6	3	3
0	0	0	0	0	3	0
1	1	1	1	1	1	1
0	0	0	0	0	1	0
1	1	1	1	1	1	1
		1	1	1	1	1
					1	
1	1	1	1	1		1
1	1	2	2	6		3
					1	
		1	1	1		1
		2	2	6		3

For peer review only

					0.0%	
					0.0%	
					0.0%	
100.0%	100.0%	100.0%	100.0%	100.0%		100.0%
					0.0%	
					0.0%	
					0.0%	
		100.0%	100.0%	100.0%		100.0%
3400	511	3334	4184	64181	39552	24721
0	0	0	0	0	0	0
1	1	2	2	6	3	3
0	0	0	0	0	0	0
1	1	1	1	1	1	1
0	0	0	0	0	0	0
1	1	1	1	1	1	1

For peer review only

1	1	1	1	1	1	1
0	0	0	0	0	0	0
3400	511	3334	4184	64181	39552	24721
100.0%	100.0%	100.0%	100.0%	100.0%	100.0%	100.0%
100.0%	100.0%	100.0%	100.0%	100.0%	100.0%	100.0%

For peer review only

For peer review only

Pain Associat	Pain Concern	Pain Relief Fo	Pain UK	Pancreatic Ca	Pancreatic Ca	Parkinson's U
10	11	3	10	7	17	12
0	0	2	0	2	3	8
1	1	1	1	1	1	1
0	0	1	0	1	1	1
1	1	1	1	1	1	1
1	1	1	1	1	1	1
	1				1	1
1	1	1	1	1	1	1
10	11	1	10	5	14	4
1	1	1	1	1	1	1
10	11	1	10	5	14	4
	1				1	1
	11				14	4

For peer review only

		33.3%				33.3%
100.0%	100.0%		100.0%			
		33.3%				33.3%
100.0%	100.0%		100.0%			
						33.3%
	100.0%					
4636	16888	1442	30101	34907	209550	29736
0	0	0	0	0	0	15781
9	9	3	10	7	17	11
0	0	0	0	0	0	2
1	1	1	1	1	1	1
0	0	0	0	0	0	1
1	1	1	1	1	1	1
					1	

For peer review only

1	1	1	1	1	1	1
0	0	0	0	0	0	0
4636	16888	1442	30101	34907	209550	13955
					1	
					0	
					209550	
						46.9%
100.0%	100.0%	100.0%	100.0%	100.0%	100.0%	
						46.9%
100.0%	100.0%	100.0%	100.0%	100.0%	100.0%	
					100.0%	

For peer review only

For peer review only

Patients Asso	Patients On I	Paula Carr Di	PBC Foundat	Pelican Canc	Pelvic Pain S	Pink Ribbon I
48	5	1	6	1	4	1
0	0	0	3	0	0	0
1	1	1	1	1	1	1
0	0	0	1	0	0	0
1	1	1	1	1	1	1
1	1		1		1	
1						
1	1	1	1	1	1	1
48	5	1	3	1	4	1
1	1		1		1	
48	5		3		4	
1						
48						

For peer review only

100.0%	100.0%	100.0%		100.0%	100.0%	100.0%
100.0%	100.0%				100.0%	
100.0%						
345520	29849	400	17418	8500	2655	10000
0	0	0	15150	0	0	0
45	5	1	6	1	4	1
0	0	0	1	0	0	0
1	1	1	1	1	1	1
0	0	0	1	0	0	0
1	1	1	1	1	1	1
1						

For peer review only

1	1	1	1	1	1	1
0	0	0	0	0	0	0
345520	29849	400	2268	8500	2655	10000
1						
0						
345520						
			13.0%			
			13.0%			
100.0%	100.0%	100.0%		100.0%	100.0%	100.0%
			13.0%			
			13.0%			
100.0%	100.0%	100.0%		100.0%	100.0%	100.0%
100.0%						

For peer review only

For peer review only

	Pituitary Fou	PNH Support	Polycystic Ki	Portsmouth I	Positive Acti	Positive East	Positive Help
	23	1	4	1	1	7	1
	21	1	0	0	0	2	1
	1	1	1	1	1	1	1
	1	1	0	0	0	1	1
	1	1	1	1	1	1	1
	1		1			1	
	1						
		1					1
	1		1	1	1	1	
	2		4	1	1	5	
	1		1			1	
	2		4			5	
	1						
	2						

For peer review only

8.7%	0.0%					0.0%
8.7%	0.0%					0.0%
8.7%	0.0%					0.0%
		100.0%	100.0%	100.0%		
8.7%						
8.7%						
8.7%						
		100.0%				
8.7%						
8.7%						
8.7%						
110099	15150	68956	152	0	42960	3042
42209	15150	0	0	0	32097	15261
23	1	4	1	0	7	1
4	1	0	0	0	2	1
1	1	1	1	0	1	1
1	1	0	0	0	1	1
1	1	1	1		1	1
1						

For peer review only

	1			FALSE		
1		1	1		1	
						1
0	0	0	0		0	0
67891		68956	152		10863	
						12218
1						
0						
67891						
	0.0%					
	0.0%					
	0.0%				25.3%	
		100.0%	100.0%			
	0.0%					
	0.0%					
	0.0%				25.3%	
		100.0%	100.0%			

For peer review only

For peer review only

Positively UK	Prader-Willi S	Primary Imm	Progress Edu	Progressive S	Prostate Can	Prostate Can
31	1	8	2	3	1	2
20	0	8	10	0	0	4
1	1	1	1	1	1	1
1	0	1	1	0	0	1
1	1	1	1	1	1	1
1		1	1	1		1
1						
		1				
1	1			1	1	
			1			1
11	1			3	1	
			8			2
		1				
1				1		
			1			1
11				3		
			8			2
1						
11						

For peer review only

		0.0%				
		0.0%				
35.5%		0.0%				
	100.0%			100.0%	100.0%	
		0.0%				
		0.0%				
35.5%		0.0%				
				100.0%		
35.5%						
130874	473	73527	1373	564	1052	2088
146722	0	69182	0	0	0	0
31	1	8	2	3	1	2
20	0	8	0	0	0	0
1	1	1	1	1	1	1
1	0	1	0	0	0	0
1	1	1	1	1	1	1
1						

For peer review only

	1	1	1	1	1	1
1						
0	0	0	0	0	0	0
	473	4345	1373	564	1052	2088
15848						
1						
0						
15848						
		5.9%				
10.8%		5.9%				
10.8%		5.9%				
	100.0%		100.0%	100.0%	100.0%	100.0%
		5.9%				
10.8%		5.9%				
10.8%		5.9%				
	100.0%		100.0%	100.0%	100.0%	100.0%
10.8%						
10.8%						

For peer review only

For peer review only

Prostate Can	Prostate Cym	Psoriasis Ass	Pulmonary H	Pumping Ma	QUIT	Rainbow Cen
15	1	37	18	29	13	1
2	0	70	13	9	0	0
1	1	1	1	1	1	1
1	0	1	1	1	0	0
1	1	1	1	1	1	1
1		1	1	1	1	
1		1	1	1	1	
1	1		1	1	1	1
		1				
13	1		5	20	13	1
		33				
1			1	1	1	
		1				
13			5	20	13	
		33				
1			1	1	1	
		1				
13			5	20	13	
		33				

For peer review only

		47.1%	27.8%			
	100.0%				100.0%	100.0%
		47.1%	27.8%			
					100.0%	
		47.1%	27.8%			
					100.0%	
108777	640	427224	274170	307930	79340	579
0	0	0	278788	96358	0	0
14	1	37	18	29	13	1
0	0	0	13	5	0	0
1	1	1	1	1	1	1
0	0	0	1	1	0	0
1	1	1	1	1	1	1
1		1	1	1		

For peer review only

1	1	1		1	1	1
			1			
0	0	0	0	0	0	0
108777	640	427224		211573	79340	579
			4618			
1		1		1		
			1			
0		0	0	0		
108777		427224		211573		
			4618			
			1.7%			
			1.7%			
			1.7%			
100.0%	100.0%	100.0%			100.0%	100.0%
			1.7%			
			1.7%			
			1.7%			
100.0%	100.0%	100.0%			100.0%	100.0%
			1.7%			
			1.7%			
			1.7%			
100.0%		100.0%				

For peer review only

For peer review only

Rainbow Tru	Rapid Effecti	Rarer Cancer	Raynaud's ar	Release	Restless Leg	Rethink Men
1	4	62	14	1	5	4
0	2	0	4	1	0	8
1	1	1	1	1	1	1
0	1	0	1	1	0	1
1	1	1	1	1	1	1
	1	1	1		1	1
		1	1			
				1		
1	1	1	1		1	
						1
1	2	62	10		5	
						4
	1	1	1		1	
						1
	2	62	10		5	
						4
		1	1			
		62	10			

For peer review only

				0.0%		
				0.0%		
				0.0%		
100.0%		100.0%			100.0%	
		100.0%			100.0%	
		100.0%				
1010	10915	682971	29651	10063	12940	61642
0	0	0	0	5143	0	12625
1	4	62	13	1	5	4
0	0	0	0	1	0	1
1	1	1	1	1	1	1
0	0	0	0	1	0	1
1	1	1	1	1	1	1
		1				

For peer review only

1	1	1	1	1	1	1
0	0	0	0	0	0	0
1010	10915	682971	29651	4921	12940	49017
		1				
		0				
		682971				
				48.9%		
100.0%	100.0%	100.0%	100.0%		100.0%	
				48.9%		
100.0%	100.0%	100.0%	100.0%		100.0%	
		100.0%				

For peer review only

For peer review only

Revive Multi	Richmond Fe	River House	Ronald McDc	Roy Castle Lu	Royal Free Cl	Royal Marsd
3	1	1	1	70	2	1
1	0	0	0	0	3	0
1	1	1	1	1	1	1
1	0	0	0	0	1	0
1	1	1	1	1	1	1
1				1	1	
				1		
1	1	1	1	1		1
					1	
2	1	1	1	70		1
					1	
1				1		
					1	
2				70		
					1	
				1		
				70		

For peer review only

					33.3%	
	100.0%	100.0%	100.0%	100.0%		100.0%
					33.3%	
				100.0%		
				100.0%		
109067	736	253	771	770325	11827	355
29239	0	0	0	0	0	0
3	1	1	1	69	2	1
1	0	0	0	0	0	0
1	1	1	1	1	1	1
1	0	0	0	0	0	0
1	1	1	1	1	1	1
1				1		

For peer review only

1	1	1	1	1	1	1
0	0	0	0	0	0	0
79828	736	253	771	770325	11827	355
1				1		
0				0		
79828				770325		
	100.0%	100.0%	100.0%	100.0%	100.0%	100.0%
	100.0%	100.0%	100.0%	100.0%	100.0%	100.0%
				100.0%		

For peer review only

For peer review only

Royal Nationa	Salamander	Samson Cent	SANE	Sarcoma UK	Save Babies	Saving Lives
43	2	1	7	6	1	3
23	0	0	0	8	1	6
1	1	1	1	1	1	1
1	0	0	0	1	1	1
1	1	1	1	1	1	1
1	1		1	1		1
1						
					1	
1	1	1	1			
				1		1
20	2	1	7			
				2		3
1	1		1			
				1		1
20	2		7			
				2		3
1						
20						

For peer review only

					0.0%	
					0.0%	
46.5%				25.0%	0.0%	
	100.0%	100.0%	100.0%			
46.5%				25.0%		
	100.0%		100.0%			
46.5%						
1236891	721	4500	83687	29998	981	32786
982982	0	0	0	13677	0	70437
42	2	1	7	6	1	3
23	0	0	0	4	0	6
1	1	1	1	1	1	1
1	0	0	0	1	0	1
1	1	1	1	1	1	1
1						
1						

For peer review only

1	1	1	1	1	1	
						1
0	0	0	0	0	0	0
253909	721	4500	83687	16321	981	
						37650
1						
0						
253909						
1						
0						
253909						
20.5%						
	100.0%	100.0%	100.0%		100.0%	
20.5%						
	100.0%	100.0%	100.0%		100.0%	
20.5%						

For peer review only

20.5%

For peer review only

Scleroderma	Scleroderma	Scoliosis Ass	Scotland Pati	Scottish Drug	Scottish Epile	Scottish Kidn
1	5	1	1	2	2	1
3	1	0	0	0	0	0
1	1	1	1	1	1	1
1	1	0	0	0	0	0
1	1	1	1	1	1	1
1	1			1	1	
	1	1	1	1	1	1
1						
	4	1	1	2	2	1
2						
	1			1	1	
1						
	4			2	2	
2						

For peer review only

		100.0%	100.0%	100.0%	100.0%	100.0%
				100.0%	100.0%	
2000	4513	278	1029	4056	78625	505
11110	634	0	0	0	0	0
1	5	1	1	2	2	1
3	1	0	0	0	0	0
1	1	1	1	1	1	1
1	1	0	0	0	0	0
1	1	1	1	1	1	1

For peer review only

	1	1	1	1	1	1
1						
0	0	0	0	0	0	0
	3879	278	1029	4056	78625	505
9110						
		100.0%	100.0%	100.0%	100.0%	100.0%
		100.0%	100.0%	100.0%	100.0%	100.0%

For peer review only

For peer review only

Scottish Netw	SeeAbility	Self Help Ser	Sense	Service by En	Sexual Advic	Shift.MS
3	1	2	1	1	9	26
0	0	0	0	0	6	4
1	1	1	1	1	1	1
0	0	0	0	0	1	1
1	1	1	1	1	1	1
1		1			1	1
						1
1	1	1	1	1	1	1
3	1	2	1	1	3	22
1		1			1	1
3		2			3	22
						1
						22

For peer review only

					33.3%	
100.0%	100.0%	100.0%	100.0%	100.0%		
					33.3%	
100.0%		100.0%				
1139	473	514	152	210	115400	279784
0	0	0	0	0	0	0
3	1	2	1	1	8	26
0	0	0	0	0	0	0
1	1	1	1	1	1	1
0	0	0	0	0	0	0
1	1	1	1	1	1	1
					1	1

For peer review only

1	1	1	1	1	1	1
0	0	0	0	0	0	0
1139	473	514	152	210	115400	279784
					1	1
					0	0
					115400	279784
100.0%	100.0%	100.0%	100.0%	100.0%	100.0%	100.0%
100.0%	100.0%	100.0%	100.0%	100.0%	100.0%	100.0%
					100.0%	100.0%

For peer review only

For peer review only

Short Bowel	Sickle Cell an	Sickle Cell So	Sign Health	Silver Star	Skin Care Cyr	Society for Mu
1	10	18	1	12	8	78
0	5	8	1	0	0	12
1	1	1	1	1	1	1
0	1	1	1	0	0	1
1	1	1	1	1	1	1
	1	1		1	1	1
		1		1		1
			1			
1	1	1		1	1	1
1	5	10		12	8	66
	1	1		1	1	1
	5	10		12	8	66
		1		1		1
		10		12		66

For peer review only

			0.0%			
			0.0%			
			0.0%			
100.0%				100.0%	100.0%	
				100.0%	100.0%	
				100.0%		
224	28443	50877	12625	117959	19925	1031298
0	37172	41925	0	0	0	0
1	10	17	1	12	8	78
0	4	8	0	0	0	0
1	1	1	1	1	1	1
0	1	1	0	0	0	0
1	1	1	1	1	1	1
				1		1
						1

1		1	1	1	1	1
	1					
0	0	0	0	0	0	0
224		8952	12625	117959	19925	1031298
	8728					
				1		1
				0		0
				117959		1031298
						1
						0
						1031298
		17.6%				
	23.5%	17.6%				
100.0%			100.0%	100.0%	100.0%	100.0%
		17.6%				
	23.5%	17.6%				
100.0%			100.0%	100.0%	100.0%	100.0%
				100.0%		100.0%

For peer review only

100.0%

For peer review only

Somerville Fc	Sophia Forun	South Asian I	Spectra	Spinal Injurie	Spinal Injurie	Spinal Muscu
3	2	1	1	1	1	2
0	0	1	0	0	0	4
1	1	1	1	1	1	1
0	0	1	0	0	0	1
1	1	1	1	1	1	1
1	1					1
		1				
1	1		1	1	1	
						1
3	2		1	1	1	
						2
1	1					
						1
3	2					
						2

For peer review only

		0.0%				
		0.0%				
		0.0%				
100.0%	100.0%		100.0%	100.0%	100.0%	
100.0%	100.0%					
7328	10120	35998	1010	253	1052	46302
0	0	0	0	0	0	0
3	2	1	1	1	1	2
0	0	0	0	0	0	0
1	1	1	1	1	1	1
0	0	0	0	0	0	0
1	1	1	1	1	1	1

For peer review only

1	1	1	1	1	1	1
0	0	0	0	0	0	0
7328	10120	35998	1010	253	1052	46302
100.0%	100.0%	100.0%	100.0%	100.0%	100.0%	100.0%
100.0%	100.0%	100.0%	100.0%	100.0%	100.0%	100.0%

For peer review only

For peer review only

St Thomas Lu	Steps	Stick N Step	Stroke Associ	SUDEP Actioı	Surrey Young	Sussex Beaco
4	1	1	39	2	1	1
0	0	1	4	0	2	0
1	1	1	1	1	1	1
0	0	1	1	0	1	0
1	1	1	1	1	1	1
1			1	1	1	
			1			
		1				
1	1		1	1		1
					1	
4	1		35	2		1
					1	
1			1	1		
					1	
4			35	2		
					1	
			1			
			35			

For peer review only

		0.0%				
		0.0%				
		0.0%				
100.0%	100.0%			100.0%		100.0%
100.0%				100.0%		
62950	152	1349	256932	2334	5070	1000
0	0	0	20570	0	10100	0
4	1	1	38	2	1	1
0	0	0	1	0	1	0
1	1	1	1	1	1	1
0	0	0	1	0	1	0
1	1	1	1	1	1	1
			1			

For peer review only

1	1	1	1	1		1
					1	
0	0	0	0	0	0	0
62950	152	1349	236362	2334		1000
					5030	
			1			
			0			
			236362			
					49.8%	
100.0%	100.0%	100.0%		100.0%		100.0%
					49.8%	
100.0%	100.0%	100.0%		100.0%		100.0%

For peer review only

For peer review only

Tackle Africa	Tackle Prosta	Target Ovari	TB Alert	Team Margo	Teenage Can	Tenovus Canc
1	13	1	2	1	11	9
2	0	3	3	0	3	0
1	1	1	1	1	1	1
1	0	1	1	0	1	0
1	1	1	1	1	1	1
1	1	1	1		1	1
	1				1	
	1			1	1	1
1		1	1			
	13			1	8	9
1		2	1			
	1				1	1
1		1	1			
	13				8	9
1		2	1			
	1				1	
	13				8	

For peer review only

			33.3%			
	100.0%			100.0%		100.0%
			33.3%			
	100.0%					100.0%
	100.0%					
3042	148470	0	27401	5050	11904	170503
6072	0	0	0	0	0	0
1	13	0	2	1	10	9
2	0	0	0	0	0	0
1	1	0	1	1	1	1
1	0	0	0	0	0	0
1	1		1	1	1	1
	1					1

For peer review only

		FALSE				
	1		1	1	1	1
1						
0	0		0	0	0	0
	148470		27401	5050	11904	170503
3030						
	1					1
	0					0
	148470					170503
49.9%						
	100.0%		100.0%	100.0%	100.0%	100.0%
49.9%						
	100.0%		100.0%	100.0%	100.0%	100.0%
	100.0%					100.0%

For peer review only

For peer review only

Terrence Hig	Thames Valle	Thrombosis U	Tiny Tickers	Tommy’s	Transplant Li	Transverse M
13	2	17	2	1	2	1
12	0	0	2	5	0	0
1	1	1	1	1	1	1
1	0	0	1	1	0	0
1	1	1	1	1	1	1
1	1	1	1	1	1	
1		1				
			1			
1	1	1			1	1
				1		
1	2	17			2	1
				4		
			1			
1	1	1			1	
				1		
1	2	17			2	
				4		
1		1				
1		17				

For peer review only

7.7%			0.0%			
7.7%			0.0%			
7.7%			0.0%			
	100.0%	100.0%			100.0%	100.0%
7.7%			0.0%			
7.7%			0.0%			
7.7%			0.0%			
	100.0%	100.0%			100.0%	
7.7%						
7.7%						
7.7%						
		100.0%				
108993	5736	45782	12221	10285	1532	15150
67744	0	0	0	0	0	0
13	2	17	2	1	2	1
8	0	0	0	0	0	0
1	1	1	1	1	1	1
1	0	0	0	0	0	0
1	1	1	1	1	1	1
1						

For peer review only

1	1	1	1	1	1	1
0	0	0	0	0	0	0
41249	5736	45782	12221	10285	1532	15150
1						
0						
41249						
37.8%						
	100.0%	100.0%	100.0%	100.0%	100.0%	100.0%
37.8%						
	100.0%	100.0%	100.0%	100.0%	100.0%	100.0%
37.8%						

For peer review only

For peer review only

Tree of Hope	Trigeminal N	Tuberous Scl	Turner Syndr	Twins And M	UK Chronic L	UK Gout Soci
1	3	8	21	6	1	3
0	0	4	11	5	0	0
1	1	1	1	1	1	1
0	0	1	1	1	0	0
1	1	1	1	1	1	1
	1	1	1	1		1
			1			
1	1	1	1	1	1	1
1	3	4	10	1	1	3
	1	1	1	1		1
	3	4	10	1		3
			1			
			10			

For peer review only

				16.7%		
			47.6%	16.7%		
100.0%	100.0%				100.0%	100.0%
				16.7%		
			47.6%	16.7%		
	100.0%					100.0%
			47.6%			
152	4208	91245	39241	42922	4056	38407
0	0	120378	0	0	0	0
1	1	8	20	6	1	3
0	0	4	0	0	0	0
1	1	1	1	1	1	1
0	0	1	0	0	0	0
1	1	1	1	1	1	1
		1				

For peer review only

1	1		1	1	1	1
		1				
0	0	0	0	0	0	0
152	4208		39241	42922	4056	38407
		29133				
		1				
		0				
		29133				
		24.2%				
100.0%	100.0%		100.0%	100.0%	100.0%	100.0%
		24.2%				
100.0%	100.0%		100.0%	100.0%	100.0%	100.0%
		24.2%				

For peer review only

For peer review only

UK Primary Ir	UK Sepsis Trι	UK Thalassae	Urology Four	Visionary	Waldenstron	Walk the Wa
15	1	7	3	6	6	1
1	0	12	5	2	3	0
1	1	1	1	1	1	1
1	0	1	1	1	1	0
1	1	1	1	1	1	1
1		1	1	1	1	
1		1				
1	1			1	1	1
		1	1			
14	1			4	3	1
		5	2			
1				1	1	
		1	1			
14				4	3	
		5	2			
1						
		1				
14						
		5				

For peer review only

		41.7%	40.0%			
	100.0%					100.0%
		41.7%	40.0%			
		41.7%				
107005	0	43405	30621	95543	25021	406
0	0	132930	0	15273	0	0
15	0	7	3	6	6	1
0	0	12	0	2	0	0
1	0	1	1	1	1	1
0	0	1	0	1	0	0
1		1	1	1	1	1
1		1				

For peer review only

	FALSE					
1			1	1	1	1
		1				
0		0	0	0	0	0
107005			30621	80270	25021	406
		89525				
1						
		1				
0		0				
107005						
		89525				
100.0%			100.0%		100.0%	100.0%
100.0%			100.0%		100.0%	100.0%
100.0%						

For peer review only

For peer review only

Waverley Ca	Wellbeing of	WellChild	Willow Foun	World Cance	World Child (	York Air Amb
18	1	2	1	1	15	1
0	1	1	0	0	0	0
1	1	1	1	1	1	1
0	1	1	0	0	0	0
1	1	1	1	1	1	1
1		1			1	
1					1	
	1					
1		1	1	1	1	1
18		1	1	1	15	1
1		1			1	
18		1			15	
1					1	
18					15	

For peer review only

	0.0%					
	0.0%					
	0.0%					
100.0%			100.0%	100.0%	100.0%	100.0%
100.0%					100.0%	
100.0%					100.0%	
103557	4114	262000	258	5000	342276	0
0	0	0	0	0	0	0
18	1	2	1	1	15	0
0	0	0	0	0	0	0
1	1	1	1	1	1	0
0	0	0	0	0	0	0
1	1	1	1	1	1	
1		1			1	

For peer review only

						FALSE
1	1	1	1	1	1	
0	0	0	0	0	0	
103557	4114	262000	258	5000	342276	
1		1			1	
0		0			0	
103557		262000			342276	
100.0%	100.0%	100.0%	100.0%	100.0%	100.0%	
100.0%	100.0%	100.0%	100.0%	100.0%	100.0%	
100.0%		100.0%			100.0%	

For peer review only

For peer review only

Young Epilepsy

14
7
1
1

1
1
1

1

7

1

7

1

7

147292
0
13
0
1
0

1
1

For peer review only

1

0
147292

1

0
147292

100.0%

100.0%

100.0%

For peer review only

For peer review only

For peer review only

For peer review only

For peer review only

Web Supplement 6. Recipients - Absolute and relative differences in the number and value of payments (in

NUMBER OF PAYMENTS - ABSOLUTE DIFFERENCES

Threshold 0: At least 1 one payment in at least one dataset
Total
Number of patient organisations with exact match in both datasets
Number of patient organisations with more paymens in industry data
Number of patient organisations with more payments in patient organisation data

Highest absolute difference between patient organisation and industry data - number of payments higher in i
Highest absolute difference between patient organisation and industry data - number of payments higher in p

Threshold 1: More than 1 one payment in at least one datase
Total
Number of patient organisations with exact match in both datasets
Number of patient organisations with more paymens in industry data
Number of patient organisations with more payments in patient organisation data

Highest absolute difference between patient organisation and industry data - number of payments higher in i
Highest absolute difference between patient organisation and industry data - number of payments higher in p

Threshold 2: More than 10 payments in at least one dataset
Total
Number of patient organisations with exact match in both datasets
Number of patient organisations with more paymens in industry data
Number of patient organisations with more payments in patient organisation data

Highest absolute difference between patient organisation and industry data - number of payments higher in i
Highest absolute difference between patient organisation and industry data - number of payments higher in p

Threshold 3: More than 100 payments in at least one dataset
Total
Number of patient organisations with exact match in both datasets
Number of patient organisations with more paymens in industry data
Number of patient organisations with more payments in patient organisation data

Highest absolute difference between patient organisation and industry data - number of payments higher in i
Highest absolute difference between patient organisation and industry data - number of payments higher in p

NUMBER OF PAYMENTS - RELATIVE DIFFERENCES

Threshold 0: At least 1 one payment in at least one dataset
Total
Relative difference <10%

For peer review only

Relative difference <20%
Relative difference <50%
Relative difference = 100%

Threshold 1: More than 1 one payment in at least one datase
Total
Relative difference <10%
Relative difference <20%
Relative difference <50%
Relative difference = 100%

Threshold 2: More than 10 payments in at least one dataset
Total
Relative difference <10%
Relative difference <20%
Relative difference <50%
Relative difference = 100%

Threshold 3: More than 100 payments in at least one dataset
Total
Relative difference <10%
Relative difference <20%
Relative difference <50%
Relative difference = 100%

VALUE OF PAYMENTS - ABSOLUTE DIFFERENCES

Threshold 1: At least one payment with value >£0 in at least one dataset
Total
Number of patient organisations with exact match in both datasets
Number of patient organisations with higher payment value in industry data
Number of patient organisations with higher payment value in patient organisation data

Highest absolute difference between patient organisation and industry data - number of payments higher in i
Highest absolute difference between patient organisation and industry data - number of payments higher in [illegible]

Threshold 2: Payments with the value of more than £10,000 in at least one dataset
Total
Number of patient organisations with exact match in both datasets
Number of patient organisations with higher payment value in industry data
Number of patient organisations with higher payment value in patient organisation data

Highest absolute difference between patient organisation and industry data - number of payments higher in i
Highest absolute difference between patient organisation and industry data - number of payments higher in [illegible]

Threshold 3: Payments with the value of more than £100,000 in at least one dataset

For peer review only

Total
Number of patient organisations with exact match in both datasets
Number of patient organisations with higher payment value in industry data
Number of patient organisations with higher payment value in patient organisation data

Highest absolute difference between patient organisation and industry data - number of payments higher in i
Highest absolute difference between patient organisation and industry data - number of payments higher in [illegible]

VALUE OF PAYMENTS - RELATIVE DIFFERENCES

Threshold 1: At least one payment with value >£0 in at least one dataset
Total
Relative difference <10%
Relative difference <20%
Relative difference <50%
Relative difference = 100%

Threshold 2: Payments with the value of more than £10,000 in at least one dataset
Total
Relative difference <10%
Relative difference <20%
Relative difference <50%
Relative difference = 100%

Threshold 3: Payments with the value of more than £100,000 in at least one dataset
Total
Relative difference <10%
Relative difference <20%
Relative difference <50%
Relative difference = 100%

For peer review only

**All years**		**2012**		**2013**	
Number of pat	% of all patient	Number of pa	% of all patient	Number of pa	% of all patient
425	100.0%	219	100.0%	225	100.0%
33	7.8%	17	7.8%	18	8.0%
335	78.8%	160	73.1%	157	69.8%
57	13.4%	42	19.2%	50	22.2%
Number of payments		Number of payments		Number of payments	
199		36		35	
33		9		17	
Number of pat	% of all patient	Number of pa	% of all patient	Number of pa	% of all patient
299	100.0%	140	100.0%	137	100.0%
13	4.3%	6	4.3%	9	6.6%
229	76.6%	105	75.0%	95	69.3%
57	19.1%	29	20.7%	33	24.1%
Number of payments		Number of payments		Number of payments	
199		36		35	
33		9		17	
Number of pat	% of all patient	Number of pa	% of all patient	Number of pa	% of all patient
115	100.0%	20	100.0%	13	100.0%
2	1.7%	1	5.0%	0	0.0%
95	82.6%	16	80.0%	9	69.2%
18	15.7%	3	15.0%	4	30.8%
Number of payments		Number of payments		Number of payments	
199		36		35	
33		9		17	
Number of pat	% of all patient	Number of pa	% of all patient	Number of pa	% of all patient
3	100.0%	0	N/A	0	N/A
0	0.0%	N/A	N/A	N/A	N/A
3	100.0%	N/A	N/A	N/A	N/A
0	0.0%	N/A	N/A	N/A	N/A
Number of payments		Number of payments		Number of payments	
199		0		0	
0		0		0	
Number of pat	% of all patient	Number of pa	% of all patient	Number of pa	% of all patient
425	100.0%	219	100.0%	225	100.0%
35	8.2%	17	7.8%	18	8.0%

For peer review only

42	9.9%	18	8.2%	19	8.4%
81	19.1%	30	13.7%	34	15.1%
225	52.9%	138	63.0%	150	66.7%

Number of pat	% of all patient	Number of pa	% of all patient	Number of pa	% of all patient
299	100.0%	140	100.0%	137	100.0%
15	5.0%	6	4.3%	9	6.6%
22	7.4%	7	5.0%	10	7.3%
61	20.4%	19	13.6%	25	18.2%
119	39.8%	70	50.0%	71	51.8%

Number of pat	% of all patient	Number of pa	% of all patient	Number of pa	% of all patient
115	100.0%	20	100.0%	13	100.0%
4	3.5%	1	5.0%	0	0.0%
7	6.1%	2	10.0%	0	0.0%
31	27.0%	4	20.0%	1	7.7%
33	28.7%	7	35.0%	5	38.5%

Number of pat	% of all patient	Number of pa	% of all patient	Number of pa	% of all patient
3	100.0%	0	N/A	0	N/A
0	0.0%	N/A	N/A	N/A	N/A
0	0.0%	N/A	N/A	N/A	N/A
0	0.0%	N/A	N/A	N/A	N/A
1	33.3%	N/A	N/A	N/A	N/A

Number of pat	% of all patient	Number of pa	% of all patient	Number of pa	% of all patient
416	100.0%	206	100.0%	204	100.0%
8	1.9%	4	1.9%	4	2.0%
356	85.6%	168	81.6%	164	80.4%
52	12.5%	34	16.5%	36	17.6%

Value of payments	Value of payments	Value of payments
6,718,576.6	660,846.1	369,657.9
6,493,237.1	283,684.7	829,991.7

Number of pat	% of all patient	Number of pa	% of all patient	Number of pa	% of all patient
104	100.0%	29	100.0%	20	100.0%
0	0.0%	0	0.0%	0	0.0%
80	76.9%	19	65.5%	12	60.0%
24	23.1%	10	34.5%	8	40.0%

Value of payments	Value of payments	Value of payments
6,718,576.6	660,846.1	369,657.9
6,493,237.1	283,684.7	829,991.7

Number of pat	% of all patient	Number of pa	% of all patient	Number of pa	% of all patient

For peer review only

13	100.0%		0	N/A		0	N/A
0	0.0%	N/A	N/A	N/A	N/A
6	46.2%	N/A	N/A	N/A	N/A
7	53.8%	N/A	N/A	N/A	N/A

Value of payments	Value of payments	Value of payments
6,718,576.6	-	-
6,493,237.1	-	-

Number of pat	% of all patient	Number of pa	% of all patient	Number of pa	% of all patient
416	100.0%	206	100.0%	204	100.0%
21	5.0%	6	2.9%	8	3.9%
34	8.2%	11	5.3%	13	6.4%
73	17.5%	20	9.7%	21	10.3%
295	70.9%	158	76.7%	164	80.4%

Number of pat	% of all patient	Number of pa	% of all patient	Number of pa	% of all patient
104	100.0%	29	100.0%	20	100.0%
4	3.8%	0	0.0%	0	0.0%
8	7.7%	2	6.9%	0	0.0%
23	22.1%	4	13.8%	2	10.0%
54	51.9%	17	58.6%	11	55.0%

Number of pat	% of all patient	Number of pa	% of all patient	Number of pa	% of all patient
13	100.0%	0	N/A	0	N/A
0	0.0%	N/A	N/A	N/A	N/A
0	0.0%	N/A	N/A	N/A	N/A
3	23.1%	N/A	N/A	N/A	N/A
4	30.8%	N/A	N/A	N/A	N/A

For peer review only

2014		2015		2016
Number of pati	% of all patient	Number of pati	% of all patient organisatic	Number of pati
247	100.0%	267	100.0%	260
29	11.7%	31	11.6%	21
173	70.0%	186	69.7%	208
45	18.2%	50	18.7%	31
Number of payments		Number of payments		Number of payr
36		44		55
15		10		11
Number of pati	% of all patient	Number of pati	% of all patient organisatic	Number of pati
146	100.0%	149	100.0%	166
6	4.1%	8	5.4%	7
110	75.3%	107	71.8%	138
30	20.5%	34	22.8%	21
Number of payments		Number of payments		Number of payr
36		44		55
15		10		11
Number of pati	% of all patient	Number of pati	% of all patient organisatic	Number of pati
15	100.0%	22	100.0%	29
0	0.0%	0	0.0%	0
11	73.3%	19	86.4%	26
4	26.7%	3	13.6%	3
Number of payments		Number of payments		Number of payr
36		44		55
15		10		11
Number of pati	% of all patient	Number of pati	% of all patient organisatic	Number of pati
0	N/A	0	N/A	0
N/A	N/A	N/A	N/A	N/A
N/A	N/A	N/A	N/A	N/A
N/A	N/A	N/A	N/A	N/A
Number of payments		Number of payments		Number of payr
0		0		0
0		0		0
Number of pati	% of all patient	Number of pati	% of all patient organisatic	Number of pati
247	100.0%	267	100.0%	260
29	11.7%	31	11.6%	21

For peer review only

32	13.0%	33	12.4%	24
48	19.4%	49	18.4%	45
153	61.9%	164	61.4%	159

Number of pati	% of all patient	Number of pati	% of all patient organisatic	Number of pati
146	100.0%	149	100.0%	166
6	4.1%	8	5.4%	7
9	6.2%	10	6.7%	10
25	17.1%	26	17.4%	31
75	51.4%	69	46.3%	79

Number of pati	% of all patient	Number of pati	% of all patient organisatic	Number of pati
15	100.0%	22	100.0%	29
0	0.0%	0	0.0%	0
1	6.7%	1	4.5%	1
3	20.0%	4	18.2%	6
5	33.3%	7	31.8%	13

Number of pati	% of all patient	Number of pati	% of all patient organisatic	Number of pati
0	N/A	0	N/A	0
N/A	N/A	N/A	N/A	N/A
N/A	N/A	N/A	N/A	N/A
N/A	N/A	N/A	N/A	N/A
N/A	N/A	N/A	N/A	N/A

Number of pati	% of all patient	Number of pati	% of all patient organisatic	Number of pati
234	100.0%	249	100.0%	249
11	4.7%	15	6.0%	8
196	83.8%	198	79.5%	211
27	11.5%	36	14.5%	30

Value of payments	Value of payments	Value of payme
538,969.2	1,473,965.2	6,006,677.7
1,824,997.0	815,956.8	6,493,237.1

Number of pati	% of all patient	Number of pati	% of all patient organisatic	Number of pati
31	100.0%	34	100.0%	36
0	0.0%	0	0.0%	0
24	77.4%	25	73.5%	25
7	22.6%	9	26.5%	11

Value of payments	Value of payments	Value of payme
538,969.2	1,473,965.2	6,006,677.7
1,824,997.0	815,956.8	6,493,237.1

Number of pati	% of all patient	Number of pati	% of all patient organisatic	Number of pati

For peer review only

1	100.0%	1	100.0%	2
0	0.0%	0	0.0%	0
0	0.0%	1	100.0%	1
1	100.0%	0	0.0%	1

Value of payments	Value of payments	Value of payme
-	1,473,965.24	6,006,677.66
1,824,996.99	-	6,493,237.05

Number of pati	% of all patient	Number of pati	% of all patient organisatic	Number of pati
234	100.0%	249	100.0%	249
16	6.8%	20	8.0%	18
19	8.1%	25	10.0%	26
28	12.0%	39	15.7%	36
185	79.1%	189	75.9%	197

Number of pati	% of all patient	Number of pati	% of all patient organisatic	Number of pati
31	100.0%	34	100.0%	36
1	3.2%	0	0.0%	2
2	6.5%	0	0.0%	4
5	16.1%	5	14.7%	8
20	64.5%	21	61.8%	20

Number of pati	% of all patient	Number of pati	% of all patient organisatic	Number of pati
1	100.0%	1	100.0%	2
0	0.0%	0	0.0%	0
0	0.0%	0	0.0%	0
0	0.0%	0	0.0%	0
0	0.0%	1	100.0%	0

For peer review only

% of all patient organisationsa given threshold
100.0%
8.1%
80.0%
11.9%

nents

% of all patient organisationsa given threshold
100.0%
4.2%
83.1%
12.7%

nents

% of all patient organisationsa given threshold
100.0%
0.0%
89.7%
10.3%

nents

% of all patient organisationsa given threshold
N/A
N/A
N/A
N/A

nents

% of all patient organisationsa given threshold
100.0%
8.1%

For peer review only

9.2%
17.3%
61.2%

% of all patient organisationsa given threshold
100.0%
4.2%
6.0%
18.7%
47.6%

% of all patient organisationsa given threshold
100.0%
0.0%
3.4%
20.7%
44.8%

% of all patient organisationsa given threshold
N/A
N/A
N/A
N/A
N/A

% of all patient organisationsa given threshold
100.0%
3.2%
84.7%
12.0%

nts

% of all patient organisationsa given threshold
100.0%
0.0%
69.4%
30.6%

nts

% of all patient organisationsa given threshold

For peer review only

100.0%
0.0%
50.0%
50.0%

nts

% of all patient organisationsa given threshold
100.0%
7.2%
10.4%
14.5%
79.1%

% of all patient organisationsa given threshold
100.0%
5.6%
11.1%
22.2%
55.6%

% of all patient organisationsa given threshold
100.0%
0.0%
0.0%
0.0%
0.0%

For peer review only

Web Supplement 7. Donors - summary of payments missing from

	Year
	**2012**
	**2013**
	**2014**
	**2015**
	**2016**
**Total missing - calculated annually**	
**Total missing - calculated for all years**	

For peer review only

industry data

Number of companies missing from industry data

31
30
27
23
0
**N/A**
**24**

For peer review only

Companies missing from industry dataset as a share of companies in industry

103.3%
78.9%
60.0%
45.1%
0.0%
**N/A**
**38.1%**

For peer review only

Number of payments missing from industry data	Number of payments missing from industry data
124	16.1%
131	18.6%
74	9.0%
52	5.6%
54	5.0%
**435**	**10.1%**
**128**	**3.0%**

For peer review only

Value of payments missing from industry data	Value of payments missing from industry datas as a
1,731,257.8	22.2%
2,666,247.8	43.1%
218,768.4	2.5%
1,367,440.0	12.0%
474,975.0	2.4%
**6,458,689.1**	**11.9%**
**1,610,321.1**	**3.0%**

For peer review only

share of payments in industry datas

For peer review only

Web Supplement 8. Donors - payments missing from industry data (yearly breakdown)

Year	Drug company name	Number of payments	Value of payments (2016 £)
	Alcon	3	18937.5
	Alexion	1	0.0
	ApoPharma	1	23145.8
	Baxter	7	170239.7
	Bayer	8	205156.3
	BioMarin	1	0.0
	Biotest	1	22480.9
	Boehringer Ingelheim	2	9468.8
	Dermal	3	0.0
	Eisai	1	0.0
	Flynn Pharma	1	2104.2
	Fresenius Medical Care	1	7890.6
	Genzyme	9	116178.4
	Gilead	8	50500.0
	GSK	18	501114.7
2012	Ipsen	3	25250.0
	Janssen	12	50804.1
	LEO Pharma	2	0.0
	Norgine	1	0.0
	Novo Nordisk	3	0.0
	Octapharma	1	134882.3
	Pharma Mar	1	6312.5
	RB	1	83114.6
	Roche Products	13	237981.3
	Sanofi Pasteur MSD	1	20989.1
	Sigma Tau Rare Diseases	1	0.0
	Sobi	1	1052.1
	Takeda	6	23145.8
	Teva	5	15248.9
	UCB	7	5260.4
	Veriton	1	0.0
	Alcon	4	64796.3
	Alexion	2	5142.6
	Almirall	2	0.0
	Amdipharm	1	0.0
	ApoPharma	3	22627.3
	Bausch and Lomb	2	22627.3
	Baxter	3	135138.4
	Bayer	9	299046.4
	Britannia	1	0.0
	Dermal	3	0.0
	Gedeon Richter	1	0.0
	Genzyme	9	278174.8
	Gilead	10	116016.3
	GSK	16	406794.5
2013	Ipsen	2	20570.3

For peer review only

2013	IQVIA	1	0.0
	Janssen	13	42110.4
	LEO Pharma	4	0.0
	Lundbeck	1	0.0
	Meda	1	0.0
	Merck	8	238799.2
	Norgine	2	0.0
	Orphan Europe	1	1028.5
	PTC	2	0.0
	RB	2	30855.4
	Roche Products	14	126301.4
	Sigma Tau Rare Diseases	2	514.3
	Takeda	9	4695.2
	Veriton	1	0.0
	Vifor Pharma Group	2	851009.3
	Actavis	1	0.0
	Alcon	1	0.0
	Alexion	5	15210.8
	Alliance	1	0.0
	Amdipharm	1	18253.0
	ApoPharma	1	30421.7
	Baxter	2	70948.4
	Bayer	4	27301.4
	Britannia	1	0.0
	Dermal	2	0.0
	Gedeon Richter	1	0.0
	Genzyme	13	225781.6
	GSK	12	2123196.4
2014	Ipsen	2	34873.4
	Meda	1	0.0
	Mitsubishi Tanabe Pharma	1	5070.3
	Norgine	2	16884.0
	Novo Nordisk	4	16144.8
	Orion Pharma	1	0.0
	PTC	2	0.0
	Rosemont Pharmaceuticals	1	0.0
	Sigma Tau Rare Diseases	2	28869.2
	Stirling Anglian	1	0.0
	Syner-Med	2	0.0
	Teva	6	7098.4
	Veriton	2	0.0
	Vifor Pharma Group	2	470654.9
	Actavis	2	24240.0
	Aegerion	1	0.0
	ALK-Abello	1	0.0
	Amdipharm	1	15680.3
	Bausch and Lomb	1	0.0
	Britannia	1	0.0

For peer review only

	Dermal	3	0.0
	Fresenius Medical Care	1	5050.0
	Genzyme	9	257424.8
	GSK	13	211609.1
	HRA Pharma	1	0.0
2015	Norgine	3	16816.5
	Octapharma	2	64864.2
	Orphan Europe	1	0.0
	Pharma Mar	1	0.0
	PTC	1	0.0
	RB	1	30300.0
	Sanofi Pasteur MSD	1	0.0
	Santen	1	0.0
	Sigma Tau Rare Diseases	2	11586.7
	Syner-Med	1	0.0
	Thea	1	0.0
	Vifor Pharma Group	3	729868.4
	Meda	1	0.0
	ALK-Abello	1	0.0
	Bausch and Lomb	1	0.0
	Genzyme	12	348335.0
	HRA Pharma	1	0.0
	BioMarin	4	5000.0
	Allergan	5	0.0
	Napp Pharmaceuticals	2	23000.0
	Amdipharm	1	500.0
	Alexion	4	27000.0
	Orphan Europe	1	0.0
	Ferring	6	0.0
2016	Britannia	1	3884.0
	Aegerion	1	0.0
	Baxalta	1	0.0
	Diurnal	1	0.0
	PTC	1	0.0
	Bio Products Laboratory	1	10000.0
	Octapharma	2	24766.0
	Sigma Tau Rare Diseases	1	12706.0
	Baxter	1	16784.0
	Fresenius Medical Care	1	3000.0
	Jazz	1	0.0
	Mylan	1	0.0
	Dermal	2	0.0

For peer review only

Web Supplement 9. Donors - payments missing from industry data (breakdown for all years)

Year	Drug company name	Number of payments	Value of payments (2016 £)
2012-2016	RB	4	144270.0
	Genzyme	52	1225894.6
	Sigma Tau Rare Diseases	8	53676.1
	Veriton	4	0.0
	Sanofi Pasteur MSD	2	20989.1
	Fresenius Medical Care	3	15940.6
	Dermal	13	0.0
	Pharma Mar	2	6312.5
	ApoPharma	5	76194.8
	Amdipharm	4	34433.3
	Orphan Europe	3	1028.5
	Gedeon Richter	2	0.0
	PTC	6	0.0
	IQVIA	1	0.0
	Britannia	4	3884.0
	Bausch and Lomb	4	22627.3
	Syner-Med	3	0.0
	Mitsubishi Tanabe Pharma	1	5070.3
	Alliance	1	0.0
	ALK-Abello	2	0.0
	Thea	1	0.0
	Diurnal	1	0.0
	Jazz	1	0.0
	Mylan	1	0.0

For peer review only

Web Supplement 10. Donors - summary of payments missing from patier

Year	Number of companies missing from patient orgaı
**2012**	5
**2013**	3
**2014**	7
**2015**	5
**2016**	4
**Total missing - calculate**	**N/A**
**Total missing - calculate**	**3**

For peer review only

ıt organisation data

Companies missing from patient organisation data as a share of companies in

8.9%
4.6%
10.8%
7.1%
6.2%
**N/A**
**3.6%**

For peer review only

Number of payments missing from patient orga	Number of payments missing from patient orga
14	4.8%
6	1.8%
17	4.9%
20	5.5%
10	3.0%
**67**	**4.0%**
**9**	**0.5%**

For peer review only

Value of payments missing from patient organ	Value of payments missing from patient organisatic
113,328.0	3.3%
68,397.8	1.5%
138,893.5	2.6%
52,758.7	1.1%
20,649.1	0.1%
**394,027.2**	**1.2%**
**92,208.2**	**0.3%**

For peer review only

ɔn data as a share of payments in patient organisation data

For peer review only

Web Supplement 11. Donors - payments missing from patient organisation data (yearly break

Year	Drug company name	Number of payments	Value of payments (2016 £)
2012	A. Menarini	2	26302.1
	Consilient Health	2	21041.7
	Daiichi Sankyo	2	16833.3
	Meda	4	30589.3
	Servier Laboratories	4	18561.6
2013	Consilient Health	1	10285.1
	HRA Pharma	3	46797.4
	Servier Laboratories	2	11315.4
2014	A. Menarini	1	7605.4
	Almirall	3	47559.2
	Chugai	2	811.2
	Consilient Health	1	10140.6
	Grünenthal	3	12939.4
	Lundbeck	5	50702.8
	Servier Laboratories	2	9134.9
2015	Almirall	3	12538.1
	Chugai	2	15409.9
	Flynn Pharma	5	13700.7
	Rosemont Pharmaceuticals	7	0.0
	Stirling Anglian	3	11110.0
2016	Almirall	3	2871.6
	Chugai	3	2612.5
	Flynn Pharma	3	5165.0
	Orion Pharma	1	10000.0

For peer review only

:down)

For peer review only

Web Supplement 12. Donors - payments missing from patient organisation data (all yea

Year	Drug company name	Number of payments	Value of payments (2016 £)
2012-2016	A. Menarini	3	33907.5
	Consilient Health	4	41467.4
	Daiichi Sankyo	2	16833.3

For peer review only

ars)

For peer review only

Web Supplement 13. Recipients - summary of payments missing from patient organisatio

	Year	Number of patient organisations missing from pa
	**2012**	116
	**2013**	123
	**2014**	131
	**2015**	141
	**2016**	143
**Total missing - calculated annually**		**N/A**
**Total missing - calculated for all years**		**225**

For peer review only

ɔn data

Patient organisations missing from patient organisation data as a share of all

112.6%
120.6%
112.9%
111.9%
122.2%
**N/A**
**112.5%**

For peer review only

Number of payments missing from patient orga	Number of payments missing from patient orga
399	137.1%
369	113.2%
411	118.8%
462	128.0%
533	158.2%
**2174**	**130.9%**
**1472**	**88.6%**

For peer review only

Value of payments missing from patient organ	Value of payments missing from patient organisatic
3,659,293.41	105.8%
2,340,576.29	53.0%
4,427,502.34	83.5%
6,591,354.62	136.6%
4,871,241.58	32.4%
**21,889,968.23**	**66.3%**
**14,023,475.41**	**42.4%**

For peer review only

on data as share of the value of payments in patient organisation data

For peer review only

Web Suppler
Year

For peer review only

2012

For peer review only

For peer review only

For peer review only

2013

For peer review only

For peer review only

2014

For peer review only

For peer review only

For peer review only

2015

For peer review only

For peer review only

For peer review only

2016

For peer review only

For peer review only

nent 14. Recipients - payments missing from patient organisation data (yearly breakdown)

Patient organisation name

Action on Pain
Action on Pre-Eclampsia
African-Caribbean Leukaemia Trust
Age Related Diseases and Health Trust
Allergy Research Foundation
Alzheimer Scotland
Alzheimers Dementia Support
Anaphylaxis Campaign
Anticoagulation UK
Arrhythmia Alliance
Arthritis and Musculoskeletal Alliance
Atrial Fibrillation Association
Beating Bowel Cancer
Bipolar Scotland
Bipolar UK
Bladder and Bowel Foundation (taken over by Bladder and Bowel Community in 2017)
Bladder Health UK
Blood Pressure UK
Bluebell Foundation
Brain Charity
Breakthrough Breast Cancer (current name Breast Cancer Now, following merger with Breast Cancer Campaig
Breast Cancer Campaign (current name Breast Cancer Now, following merger with Breakthrough Breast Canc
British Dupuytren's Society
British Heart Foundation
British Obesity Society
Brook
Cancer 52
Cancer Focus Northern Ireland
Cancer Research UK
Child Growth Foundation
Children with Cancer UK
Children’s Heart Unit Fund
Children’s HIV Association
Chinese National Healthy Living Centre
Cleft Lip and Palate Association
Coping with Cancer in Leicestershire and Rutland
Core Charity
Crohn's in Childhood Research Association
Cure Parkinson's Trust
Cystic Fibrosis Trust
Deaf Education through Listening and Talking
Diabetes Research and Wellness Foundation
Diabetes UK
Disabled Living
Epilepsy Scotland
European Parkinson’s Disease Association

For peer review only

Family Planning Association
Forum Link
Gay Men's Health
GIST Support UK
Group B Strep Support UK
Haemophilia Society
Health and Social Care Alliance Scotland
Heartbeat
Hereditary Angioedema UK
HIV i-Base
Hughes Syndrome Foundation
ICUsteps
Independent Cancer Patients' Voice
International Alliance of Patients' Organizations
Karen Clifford Skcin cancer charity
Kidney Cancer UK
Leicestershire AIDS Support Services
Macmillan Cancer Support
Maggie's Centre
Malnutrition and Nutritional Care in the UK
Marie Curie Cancer Care
Medical Foundation for HIV and Sexual Health
Melanoma UK
Mental Health Foundation
Migraine Action
MPN Voice
Myeloma UK
Myrovlytis Trust
National Eczema Society
National Kidney Federation
National Obesity Forum
National Osteoporosis Society
OcuMel UK
Organisation for Sickle Cell Anaemia Research and Thalassaemia Support
Paget's Association
Pain Association Scotland
Pain Concern
Pain Relief Foundation
Pain UK
Pancreatic Cancer UK
Patients Association
Pelvic Pain Support Network
Positive East
Prader-Willi Syndrome Association UK
Prostate Cancer Research Centre
QUIT
Rarer Cancers Foundation
Raynaud's and Scleroderma Association (current name: Scleroderma and Raynauds UK, following the merger

For peer review only

Richmond Fellowship
Roy Castle Lung Cancer Foundation
Salamander Trust
SANE
SeeAbility
Service by Emergency Response Volunteers - Suffolk and Cambridgeshire
Shift.MS
Silver Star
Society for Mucopolysaccharide Diseases
Somerville Foundation
Spinal Injuries Scotland
Spinal Muscular Atrophy Support UK
Stroke Association
Tackle Prostate Cancer
Tenovus Cancer Care
Thames Valley Positive Support
Thrombosis UK
Trigeminal Neuralgia Association
UK Gout Society
UK Primary Immune-deficiency Patient Support
Waverley Care
World Child Cancer
21 and Co
Action on Pain
Action on Smoking and Health - Scotland
ADHD Foundation
African-Caribbean Leukaemia Trust
African's Getting Involved
Alpha-1 Awareness UK
Alpha-1 UK Support Group
Anaphylaxis Campaign
Anticoagulation UK
Arrhythmia Alliance
Arthritis and Musculoskeletal Alliance
Association for Glycogen Storage Disease
Association for Multiple Endocrine Neoplasia Disorders
Atrial Fibrillation Association
Barts and The London Charity
Beating Bowel Cancer
Bladder and Bowel Foundation (taken over by Bladder and Bowel Community in 2017)
Bladder Health UK
Bloodwise
Bowel Cancer UK
Breakthrough Breast Cancer (current name Breast Cancer Now, following merger with Breast Cancer [illegible]
Breast Cancer Haven (The Haven)
British Dupuytren's Society
British Heart Foundation
British Liver Trust

For peer review only

British Polio Fellowship
Brittle Bone Society
Brook
Cancer 52
Cancer Research UK
Cardiac Risk in the Young
Cardiomyopathy UK
Cleft Lip and Palate Association
Coeliac UK
Community Cancer Centre
Contact a Family
Crohn's in Childhood Research Association
Cure Parkinson's Trust
Cystic Fibrosis Trust
David Lewis Centre
Dementia UK
Diabetes Research and Wellness Foundation
Douglas Bader Foundation
Epilepsy Connections
Epilepsy Scotland
Family Planning Association
Forum Link
Gauchers Association
Gay Men's Health
Haemophilia Scotland
Haemophilia Society
Heart UK
Hereditary Angioedema UK
HIV i-Base
ICUsteps
Independent Cancer Patients' Voice
International Alliance of Patients' Organizations
Juvenile Diabetes Research Foundation
Kidney Cancer Support Network
Kidney Cancer UK
Leicestershire AIDS Support Services
Life Education Wessex
Liver4Life
Lupus Europe
Macmillan Cancer Support
Maggie's Centre
Marie Curie Cancer Care
Melanoma UK
Men’s Health Forum
Mesothelioma UK
MPN Voice
Myeloma UK
National AIDS Manual

For peer review only

National Eczema Society
National Kidney Federation
National Obesity Forum
National Osteoporosis Society
Northern Ireland Chest, Heart and Stroke
OcuMel UK
Oliver King Foundation
Orchid
Organisation for Sickle Cell Anaemia Research and Thalassaemia Support
Ovacome
Ovarian Cancer Action
Pain Association Scotland
Pain Concern
Pain UK
Pancreatic Cancer Action
Pancreatic Cancer UK
Parkinson's UK
Patients Association
PBC Foundation UK
Polycystic Kidney Disease Charity
Positive East
Prostate Cancer Support Organisation
Prostate Cancer UK (merged with Prostate Action in 2012, retaining its original name)
Pumping Marvellous Foundation
QUIT
Rarer Cancers Foundation
Restless Leg Syndrome UK
Ronald McDonald House Charities
Roy Castle Lung Cancer Foundation
Royal Free Charity
SANE
Sarcoma UK
Scoliosis Association UK
Scotland Patients Association
Scottish Epilepsy Initiative
Self Help Services
Shift.MS
Silver Star
South Asian Health Foundation
SUDEP Action
Target Ovarian Cancer
Tenovus Cancer Care
Thrombosis UK
Transplant Links
Trigeminal Neuralgia Association
UK Primary Immune-deficiency Patient Support
UK Sepsis Trust
Waverley Care

For peer review only

World Child Cancer
AADC Research Trust
Action Duchenne
Action for Pulmonary Fibrosis
Action on Pain
Action on Smoking and Health - Wales
Actionplus Foundation
Addaction
Addenbrooks Charitable Trust
African Health Policy Network
African-Caribbean Leukaemia Trust
Alcohol Concern
Alpha-1 UK Support Group
Anaphylaxis Campaign
Anticoagulation UK
Arrhythmia Alliance
Arthritis and Musculoskeletal Alliance
Arthritis Research UK
Association for Glycogen Storage Disease
Asthma and Allergy Foundation
Asthma Relief
Atrial Fibrillation Association
Basil Skyers Myeloma Foundation
Beating Bowel Cancer
Behcets Syndrome Society
Bladder and Bowel Foundation (taken over by Bladder and Bowel Community in 2017)
Bloodwise
Bone Cancer Research Trust
Brain Tumor Charity
Breast Cancer Campaign (current name Breast Cancer Now, following merger with Breakthrough Breast Canc
Breast Cancer Haven (The Haven)
British Dupuytren's Society
British Heart Foundation
British Liver Trust
British Pregnancy Advisory Service
British Skin Foundation
Brook
Building for the Future
Cancer 52
Cancer Focus Northern Ireland
Cancer Research UK
Catholics for AIDS Prevention and Support
Child Growth Foundation
Children with Cancer in Malawi
Chronic Lymphocytic Leukaemia Support Association
Contact a Family
Core Charity
Crohn's in Childhood Research Association

For peer review only

Cure Parkinson's Trust
Cystic Fibrosis Trust
Diabetes Research and Wellness Foundation
Disabled Living
Edinburgh and Lothian Prostate Cancer Support Group
Epilepsy Connections
Epilepsy Scotland
European Parkinson’s Disease Association
Family Planning Association
Food Chain
FORCE
Forum Link
Freshwinds
Gay Men's Health
Haemophilia Society
Heart UK
Hepatitis B Positive Trust
HIV i-Base
Independent Cancer Patients' Voice
Juvenile Diabetes Research Foundation
Karen Clifford Skcin cancer charity
Kidney Cancer Support Network
Kidney Cancer UK
Leukaemia CARE
Liver4Life
Make A Wish Foundation
Melanoma UK
Mental Health Foundation
Mesothelioma UK
Migraine Action
MPN Voice
Multi Organ Transplant Support
National AIDS Manual
National Association for Pre-Menstrual Syndrome
National Eczema Society
National Kidney Federation
National Osteoporosis Society
Niemann-Pick UK
North of England Bone Marrow and Thalassaemia Association
Northern Ireland Rare Disease Partnership
Orchid
Ovacome
Ovarian Cancer Action
Paget's Association
Pain Concern
Pain UK
Pancreatic Cancer UK
Parkinson’s UK

For peer review only

Patients Association
PBC Foundation UK
Polycystic Kidney Disease Charity
Positive East
Prostate Cancer Support Organisation
Prostate Cancer UK (merged with Prostate Action in 2012, retaining its original name)
Prostate Cymru
Rainbow Centre for Conductive Education
Rapid Effective Assistance For Children With Potentially Terminal Illness
Rarer Cancers Foundation
Raynaud's and Scleroderma Association (current name: Scleroderma and Raynauds UK, following the merger
Release
Restless Leg Syndrome UK
Revive Multiple Sclerosis Support
Roy Castle Lung Cancer Foundation
Royal Marsden Cancer Charity
Royal National Institute of Blind People
SANE
Sarcoma UK
Scleroderma Society (current name: Scleroderma and Raynauds UK, following the merger of Raynaud's and S
Scottish Drugs Forum
Scottish Epilepsy Initiative
Silver Star
Skin Care Cymru
Sophia Forum
St Thomas Lupus Trust
Stroke Association
SUDEP Action
Tackle Prostate Cancer
Transplant Links
UK Chronic Lymphocytic Leukaemia Forum
UK Gout Society
UK Primary Immune-deficiency Patient Support
Walk the Walk Worldwide
Waverley Care
World Child Cancer
Action Duchenne
Action on Pain
Addaction
ADHD Foundation
African Health Policy Network
Age UK
aHUS UK
Alcohol Concern
Alpha-1 UK Support Group
Anaphylaxis Campaign
Angelman Syndrome Support Education Research Trust
Anticoagulation UK

For peer review only

Anxiety UK
Aplastic Anaemia Trust
Arrhythmia Alliance
Arthritis and Musculoskeletal Alliance
Association for Glycogen Storage Disease
Atrial Fibrillation Association
BackCare
Basil Skyers Myeloma Foundation
Beat Parkinson’s
Beating Bowel Cancer
Bladder and Bowel Foundation (taken over by Bladder and Bowel Community in 2017)
Bloodwise
Bluebell Foundation
Body and Soul
Bone Cancer Research Trust
Breast Cancer Campaign (current name Breast Cancer Now, following merger with Breakthrough Breast Canc
Breast Cancer Haven (The Haven)
Breast Cancer Now (created after the merger of Breast Cancer Campaign and Breakthrough Breast Cancer in
British Dupuytren's Society
British Heart Foundation
British Liver Trust
British Skin Foundation
Brook
Cambridge Rare Disease Network
Cancer 52
Cancer Focus Northern Ireland
Cancer Research UK
Candlelighters
Children with Cancer UK
Children's Liver Disease Foundation
Chronic Lymphocytic Leukaemia Support Association
CLIC Sargent Cancer Care For Children
Contact a Family
Cure Parkinson's Trust
Cystic Fibrosis Trust
Delete Blood Cancer
Diabetes UK
Diabetics with Eating Disorders
Diamond Blackfan Anaemia UK
Douglas Bader Foundation
Encephalitis Society
Food Chain
Forum Link
Freshwinds
Friends of FOP
Gauchers Association
Gay Men's Health
Haemophilia Scotland

For peer review only

Haemophilia Society
Haemophilia Wales
Hidradenitis Suppurativa Trust
HIV i-Base
Independent Cancer Patients' Voice
Input Diabetes
International Chronic Myeloid Leukemia Foundation
Karen Clifford Skcin cancer charity
Kidney Cancer Support Network
Kidney Cancer UK
Kidney Care UK
LATCH Welsh Children's Cancer Charity
Leeds Teaching Hospital Charitable Foundation
Little Hearts Matter
Liver4Life
Lullaby Trust
Lupus Europe
Lupus UK
Macular Society
Maggie's Centre
MAMA Academy
Melanoma UK
Mental Health Foundation
Mesothelioma UK
Movember Foundation
Multiple Births Foundation
Myasthenia Gravis Association
NARA - The Breathing Charity
National AIDS Manual
National Eczema Society
National Kidney Federation
National Osteoporosis Society
NMO Research Foundation
North of England Bone Marrow and Thalassaemia Association
Older People’s Advocacy Alliance
Ovacome
Pancreatic Cancer UK
Patients Association
Patients On Intravenous and Nasogastric Nutrition Therapy
Pelvic Pain Support Network
Portsmouth Down Syndrome Association
Positive Action
Progressive Supranuclear Palsy Association
Prostate Cancer UK (merged with Prostate Action in 2012, retaining its original name)
QUIT
Rainbow Trust Children's Charity
Rapid Effective Assistance For Children With Potentially Terminal Illness
Rarer Cancers Foundation

For peer review only

Raynaud's and Scleroderma Association (current name: Scleroderma and Raynauds UK, following the merger
Restless Leg Syndrome UK
Rethink Mental Illness
River House Trust
Roy Castle Lung Cancer Foundation
Royal Free Charity
Royal National Institute of Blind People
Salamander Trust
SANE
Scleroderma Society (current name: Scleroderma and Raynauds UK, following the merger of Raynaud's and Sc
Scottish Kidney Federation
Sense
Silver Star
Skin Care Cymru
Sophia Forum
Spectra
Spinal Injuries Association
St Thomas Lupus Trust
Steps
Stroke Association
Tackle Prostate Cancer
TB Alert
Team Margot
Teenage Cancer Trust
Tenovus Cancer Care
Thrombosis UK
Transverse Myelitis Society
Tree of Hope
UK Primary Immune-deficiency Patient Support
UK Thalassaemia Society
Waverley Care
World Child Cancer
York Air Ambulance
Action Bladder Cancer UK
Action Duchenne
Action on Smoking and Health - Wales
Actionplus Foundation
Addaction
ADHD Foundation
African-Caribbean Leukaemia Trust
ALD Life
Allergy UK
Alpha-1 UK Support Group
Ankylosing Spondylitis International Federation
Anthony Nolan
Anticoagulation UK
Arrhythmia Alliance
Arthritis and Musculoskeletal Alliance

For peer review only

Asyabi UK
Atrial Fibrillation Association
Back on Track
BackCare
Basil Skyers Myeloma Foundation
Beating Bowel Cancer
Birdshot Uveitis Society
Bladder and Bowel Foundation (taken over by Bladder and Bowel Community in 2017)
Body and Soul
Brain Tumour Research
Breast Cancer Haven (The Haven)
British Dupuytren's Society
British Liver Trust
British Skin Foundation
Brook
Cambridge Rare Disease Network
Cancer 52
Cancer Focus Northern Ireland
Cancer Research UK
Cardiac Risk in the Young
Cardiomyopathy UK
Charlie Waller Memorial Trust
Child Growth Foundation
Children's Liver Disease Foundation
Chronic Lymphocytic Leukaemia Support Association
CLIC Sargent Cancer Care For Children
Coppafeel!
Crohn's in Childhood Research Association
Cure and Action for Tay-Sachs
Cure Parkinson's Trust
Cystic Fibrosis Trust
Delete Blood Cancer
Diabetes UK
Diabetics with Eating Disorders
Different Strokes
Disabled Living
Encephalitis Society
Epilepsy Action
Epilepsy Scotland
Family Planning Association
Foundation for Liver Research
GIST Support UK
Guillain Barre and associated inflammatory neuropathies
Haemophilia Scotland
Haemophilia Society
Haemophilia Wales
Hafal
Headway

For peer review only

Heart UK
HIV i-Base
HIV Scotland
Imperial College Healthcare Charity
Independent Cancer Patients' Voice
Juvenile Diabetes Research Foundation
KAITY
Karen Clifford Skcin cancer charity
Kidney Cancer Support Network
Kidney Cancer UK
Kidney Care UK
Liver4Life
Lupus Europe
Macmillan Cancer Support
Make A Wish Foundation
Marie Curie Cancer Care
Martin Fisher Foundation
MDS UK Support Group
Melanoma Focus
Melanoma Fund
Melanoma UK
Mesothelioma UK
Migraine Action
Motor Neurone Disease Association
MPN Voice
Multiple Sclerosis UK
Muscular Dystrophy UK
National Attention Deficit Disorder Information and Support Service
National Cancer Research Institute
National Childbirth Trust
National Eczema Society
National Kidney Federation
National Osteoporosis Society
National Rheumatoid Arthritis Society
NMO Research Foundation
Northern Ireland Rare Disease Partnership
Olive Tree Cancer Support Centre - Crawley
Ovacome
Ovarian Cancer Action
Paget's Association
Pain Concern
Pancreatic Cancer Action
Parkinson’s UK
Patients Association
Patients On Intravenous and Nasogastric Nutrition Therapy
Paula Carr Diabetes Trust
Pelican Cancer Foundation
Pink Ribbon Foundation – Breast Cancer Charity

For peer review only

Polycystic Kidney Disease Charity
Progressive Supranuclear Palsy Association
QUIT
Rarer Cancers Foundation
Restless Leg Syndrome UK
Roy Castle Lung Cancer Foundation
Royal National Institute of Blind People
Samson Centre for MS
SANE
Sarcoma UK
Save Babies Through Screening Foundation UK
Scleroderma and Raynauds UK (established following the merger of Raynaud's and Scleroderma Association a
Scottish Network Children with Arthrities
Sexual Advice Association
Short Bowel Survivor and Friends
Skin Care Cymru
Somerville Foundation
Sussex Beacon
Tackle Prostate Cancer
Teenage Cancer Trust
Tenovus Cancer Care
Thames Valley Positive Support
Thrombosis UK
Tuberous Sclerosis Association
Visionary
Waldenstroms Macroglobulinaemia UK
Waverley Care
WellChild
Willow Foundation
World Cancer Research Fund
World Child Cancer
Young Epilepsy

For peer review only

Number of payments	Value of payments (2016 £)
3	3488.9
1	10520.8
1	15781.3
2	39258.9
2	21041.7
2	563.6
1	1057.3
1	15860.2
12	20260.5
6	7381.0
5	37832.9
36	227017.3
5	36010.8
3	3193.8
3	4145.2
13	415672.2
7	6165.9
1	10520.8
1	473.4
1	981.5
2	5298.0
1	6312.5
2	599.7
1	117.8
1	420.8
4	33929.7
5	33140.6
3	5681.3
10	660846.1
1	1578.1
1	473.4
1	736.5
1	7890.6
1	252.5
1	58.9
1	4187.3
1	8416.7
3	12625.0
1	254.6
2	12650.6
1	526.0
1	2104.2
29	315722.5
1	5260.4
1	37.6
1	21362.0

For peer review only

3	7680.2
2	747.0
3	9153.1
4	12414.6
1	153.6
9	50719.9
1	37.6
1	757.5
3	24923.9
4	16970.1
1	2104.2
1	2099.5
2	4103.1
3	122044.1
1	10520.8
8	24125.3
2	925.8
5	19768.6
1	157.8
1	883.8
1	241.4
1	5260.4
2	21041.7
2	79095.6
2	25776.0
2	18170.9
7	215976.6
1	1420.3
9	20541.9
8	144188.0
2	1157.3
3	13082.8
1	1052.1
1	2104.2
1	2104.2
4	3000.9
4	6689.5
1	631.3
4	16916.6
1	8416.7
10	66089.5
3	1188.0
1	16496.7
1	473.4
1	1052.1
7	19502.5
16	134019.4
5	5946.4

For peer review only

1	736.5
11	136368.8
1	417.7
1	1052.1
1	473.4
1	210.4
1	2865.9
4	40505.2
14	161724.0
2	6327.7
1	1052.1
1	26302.1
3	4771.9
1	5260.4
1	45554.2
1	736.5
5	19805.5
2	4208.3
1	10520.8
2	5643.4
2	9021.6
2	16096.9
1	1028.5
6	5139.5
1	2519.9
1	205.7
1	18513.2
1	129.6
2	16456.2
2	15942.0
1	25712.8
20	73150.1
6	78372.7
5	52570.4
1	2057.0
1	0.0
20	108390.1
1	205.7
5	30968.5
2	21418.9
3	8377.2
1	236.7
1	10285.1
6	29896.9
1	771.4
3	2611.4
3	742.3
2	15633.4

For peer review only

1	0.0
1	15427.7
3	23347.3
4	32912.4
9	33791.8
1	462.8
1	518.7
1	62.0
1	129.6
1	1028.5
1	7713.8
2	7207.8
2	3983.1
3	1016.7
1	1892.5
1	2592.9
1	2057.0
1	778.1
1	2057.0
1	1285.6
4	41449.1
2	5425.4
6	23100.4
2	2879.8
4	6428.2
5	65192.3
25	188350.6
4	40673.6
4	16263.9
1	2057.0
1	3085.5
3	76571.8
4	51906.0
1	48032.0
3	32912.4
2	21290.2
1	518.7
1	360.0
1	8733.1
7	21486.0
1	2809.2
1	251.5
3	19582.9
2	5130.7
2	10626.4
1	1028.5
8	207709.3
4	23005.8

For peer review only

2	2327.5
7	62885.3
1	2351.2
8	22156.9
1	1028.5
1	1028.5
1	510.9
1	3085.5
1	2079.9
2	5656.8
1	10285.1
6	1635.3
5	5471.7
3	6582.5
1	771.4
1	8228.1
3	8964.6
9	61969.6
1	475.1
2	9745.2
2	579.1
1	1626.1
4	3594.9
2	31883.9
3	22722.3
13	122253.9
1	2571.3
1	771.4
14	124200.5
1	11321.9
1	20428.5
1	1028.5
1	277.7
1	1028.5
1	2571.3
2	514.3
3	41140.5
6	48340.1
1	35998.0
1	1563.3
1	0.0
1	2571.3
3	14399.2
1	771.4
1	0.0
4	19688.8
1	0.0
1	4114.1

For peer review only

2	20914.8
1	5070.3
2	3042.2
1	595.8
2	4056.2
1	2028.1
1	2940.8
9	122041.7
1	3042.2
2	20854.0
3	20857.1
4	30421.7
1	60843.4
1	608.4
8	60166.5
6	89084.8
9	85180.7
3	583.1
1	7098.4
1	507.0
1	1014.1
20	86721.7
1	7098.4
8	133200.9
1	20281.1
6	234694.2
3	16934.7
2	10140.6
1	12675.7
5	65913.7
2	12417.3
1	11465.2
2	354.9
2	33463.9
1	507.0
3	3549.2
3	25604.9
1	243.4
5	39041.2
7	10280.6
9	425099.6
1	4089.1
1	1521.1
1	20179.7
1	20281.1
1	7605.4
1	8112.4
2	7098.4

For peer review only

2	342.6
4	7250.5
1	2433.7
3	17827.1
2	804.5
1	2028.1
3	3442.7
2	24984.5
3	22917.7
1	3042.2
1	507.0
1	6084.3
1	3042.2
3	11734.7
15	112989.0
18	178932.5
1	5070.3
2	10647.6
1	3042.2
8	205566.7
5	43874.2
1	507.0
5	31800.8
8	78082.3
5	59736.0
1	507.0
5	133910.2
1	1216.9
1	9944.8
1	33463.9
1	22682.4
1	6084.3
7	82397.2
1	152.1
1	304.2
6	80617.5
3	77459.8
2	16267.8
3	4563.3
1	2535.1
1	248.6
1	10951.8
1	19267.1
1	15210.8
1	3042.2
3	6601.5
9	38939.8
3	6135.0

For peer review only

9	69674.0
2	608.4
1	15210.8
1	4563.3
1	461.8
5	26839.5
1	639.6
1	579.3
1	2738.0
14	194892.6
2	1139.3
1	10063.5
2	3338.3
1	40562.2
13	179410.6
1	354.9
9	282414.7
2	26365.5
1	1014.1
1	671.8
2	4056.2
1	76054.2
1	19013.6
2	1014.1
1	5070.3
2	835.0
9	65132.8
1	770.7
4	9011.9
1	760.5
1	4056.2
2	27886.5
3	21428.0
1	405.6
3	19058.2
7	163600.7
5	32262.1
1	1262.5
9	189819.4
3	16160.0
4	10327.3
2	555.5
1	25250.0
6	52988.6
1	60600.0
4	3030.0
1	303.0
18	118527.0

For peer review only

1	3282.5
1	1616.0
10	108324.5
8	61610.0
4	18555.7
14	107478.3
1	3535.0
1	18852.7
1	5050.0
9	77810.4
11	1473965.2
6	40753.5
1	303.0
4	57945.7
1	2020.0
1	9090.0
1	19705.1
1	0.0
1	31916.0
1	666.6
5	17018.5
1	4155.1
1	8080.0
1	1574.6
5	63630.0
2	12378.2
14	662211.6
1	0.0
1	151.5
1	1010.0
5	47672.0
2	4559.1
1	5050.0
1	10254.5
2	9595.0
2	14402.6
44	899692.9
3	1347.3
1	15150.0
1	606.0
1	505.0
1	5050.0
1	13907.7
1	8888.0
1	0.0
3	19493.0
1	505.0
8	13847.3

For peer review only

14	190024.6
5	5972.6
2	6019.6
1	8080.0
2	4545.0
1	30322.2
1	34105.2
3	27270.0
3	20200.0
10	56661.0
1	526.2
1	1010.0
1	353.5
1	252.5
8	131184.9
1	0.0
1	25668.1
1	707.0
1	15.2
1	445.9
1	202.0
6	124798.6
1	1212.0
1	10100.0
1	1010.0
1	505.0
1	2020.0
1	0.0
7	47908.3
1	202.0
5	61027.2
8	95647.0
1	5050.0
1	1212.0
2	30300.0
2	32572.5
4	122715.0
9	64371.2
1	1520.2
1	1466.7
1	151.5
1	0.0
1	151.5
2	5302.5
2	32115.0
1	1010.0
1	2462.4
10	126755.0

For peer review only

3	20200.0
1	3030.0
1	757.5
1	252.5
14	164788.4
1	505.0
7	230060.8
1	303.0
2	15840.8
2	2473.5
1	505.0
1	151.5
1	10100.0
4	18060.7
1	5050.0
1	1010.0
1	252.5
2	62115.0
1	151.5
9	47571.0
5	83197.3
2	27401.3
1	5050.0
4	3838.0
4	70809.1
2	2525.0
1	15150.0
1	151.5
2	20701.0
1	4040.0
5	15361.1
1	50499.9
1	0.0
1	101.0
5	11772.1
1	5000.0
1	5000.0
2	45000.0
4	6620.0
1	15000.0
1	262.3
3	10160.5
1	1200.0
6	38722.8
1	20000.0
18	103102.5
6	46355.0
5	25000.0

For peer review only

1	1500.0
14	91536.8
1	3809.2
2	4000.0
2	9250.0
15	64682.6
1	10000.0
6	125466.9
2	40000.0
1	262.3
1	15000.0
1	9000.0
8	43810.0
2	2080.2
1	5000.0
3	7745.7
7	80375.0
1	824.2
15	35685.8
6	5808.1
4	1819.2
1	1000.0
2	5937.2
1	15000.0
6	18757.8
1	853.0
1	10000.0
2	650.0
2	120.0
3	9914.0
6	250684.0
1	262.3
55	972606.1
1	200.0
5	10786.9
1	500.0
1	262.3
1	1624.0
2	1351.0
1	15000.0
1	57537.0
5	35143.0
2	6400.0
2	13000.0
14	158595.7
5	7748.3
1	4000.0
1	150.0

For peer review only

15	227311.4
2	11500.0
1	3000.0
1	4000.0
5	15115.4
1	36000.0
1	2000.0
1	10000.0
4	32597.2
11	38214.9
1	228.0
10	57018.2
2	21101.4
14	98362.3
1	257.6
1	150.0
1	15000.0
3	7068.8
3	46207.0
2	15000.0
8	87500.0
3	3900.0
3	1139.7
2	280.0
2	2355.5
1	603.4
1	6000.0
1	45.0
1	1000.0
1	9600.0
1	300.0
4	48000.0
13	118394.2
15	121433.8
2	1600.0
1	1500.0
1	3400.0
1	15000.0
1	10000.0
1	7405.6
1	1685.0
1	1250.0
4	1984.0
11	83416.0
4	28328.7
1	400.0
1	8500.0
1	10000.0

For peer review only

1	44000.0
2	412.3
1	5000.0
9	105050.0
1	4000.0
18	165556.8
9	178153.0
1	4500.0
1	20000.0
1	3000.0
1	981.0
1	2000.0
3	1139.3
1	30000.0
1	224.0
2	850.0
1	1000.0
1	1000.0
3	51000.0
2	2538.0
3	51568.0
1	5000.0
7	9052.8
1	23754.0
2	15000.0
3	9851.0
7	56002.3
2	262000.0
1	257.6
1	5000.0
3	91163.6
1	18000.0

For peer review only

Web Supplement 15. Recipients - payments missing from patient organisation data (all years)

Patient organisation name

21 and Co
AADC Research Trust
Action Bladder Cancer UK
Action Duchenne
Action on Pain
Action on Smoking and Health - Scotland
Actionplus Foundation
Addenbrooks Charitable Trust
African-Caribbean Leukaemia Trust
African's Getting Involved
Age Related Diseases and Health Trust
aHUS UK
ALD Life
Allergy Research Foundation
Alpha-1 Awareness UK
Alpha-1 UK Support Group
Alzheimer Scotland
Alzheimers Dementia Support
Angelman Syndrome Support Education Research Trust
Ankylosing Spondylitis International Federation
Anticoagulation UK
Aplastic Anaemia Trust
Arrhythmia Alliance
Arthritis and Musculoskeletal Alliance
Asthma and Allergy Foundation
Asthma Relief
Asyabi UK
Atrial Fibrillation Association
Back on Track
Barts and The London Charity
Basil Skyers Myeloma Foundation
Beat Parkinson’s
Beating Bowel Cancer
Behcets Syndrome Society
Bipolar Scotland
Bipolar UK
Birdshot Uveitis Society
Bladder and Bowel Foundation (taken over by Bladder and Bowel Community in 2017)
Blood Pressure UK
Bluebell Foundation
Body and Soul
Bone Cancer Research Trust
Brain Charity
Brain Tumor Charity
Brain Tumour Research
Breast Cancer Campaign (current name Breast Cancer Now, following merger with Breakthrough Breast Canc

For peer review only

Breast Cancer Haven (The Haven)
British Dupuytren's Society
British Heart Foundation
British Obesity Society
British Polio Fellowship
British Skin Foundation
Brook
Building for the Future
Cambridge Rare Disease Network
Cancer 52
Cancer Focus Northern Ireland
Cancer Research UK
Candlelighters
Cardiac Risk in the Young
Cardiomyopathy UK
Catholics for AIDS Prevention and Support
Charlie Waller Memorial Trust
Children with Cancer UK
Children's Liver Disease Foundation
Children’s Heart Unit Fund
Chinese National Healthy Living Centre
Chronic Lymphocytic Leukaemia Support Association
Cleft Lip and Palate Association
CLIC Sargent Cancer Care For Children
Community Cancer Centre
Contact a Family
Coping with Cancer in Leicestershire and Rutland
Crohn's in Childhood Research Association
Cure Parkinson's Trust
Cystic Fibrosis Trust
David Lewis Centre
Deaf Education through Listening and Talking
Delete Blood Cancer
Dementia UK
Diabetes Research and Wellness Foundation
Diabetics with Eating Disorders
Diamond Blackfan Anaemia UK
Different Strokes
Douglas Bader Foundation
Edinburgh and Lothian Prostate Cancer Support Group
Encephalitis Society
Epilepsy Connections
Epilepsy Scotland
FORCE
Forum Link
Freshwinds
Group B Strep Support UK
Guillain Barre and associated inflammatory neuropathies

For peer review only

Haemophilia Society
Haemophilia Wales
Hafal
Headway
Health and Social Care Alliance Scotland
Heartbeat
Hepatitis B Positive Trust
HIV i-Base
Hughes Syndrome Foundation
ICUsteps
Imperial College Healthcare Charity
Independent Cancer Patients' Voice
Input Diabetes
KAITY
Karen Clifford Skcin cancer charity
Kidney Cancer Support Network
Kidney Cancer UK
Kidney Care UK
LATCH Welsh Children's Cancer Charity
Leeds Teaching Hospital Charitable Foundation
Leicestershire AIDS Support Services
Life Education Wessex
Little Hearts Matter
Liver4Life
Lullaby Trust
Lupus Europe
Lupus UK
Macular Society
Maggie's Centre
Make A Wish Foundation
Malnutrition and Nutritional Care in the UK
MAMA Academy
Marie Curie Cancer Care
Martin Fisher Foundation
Melanoma UK
Mesothelioma UK
Motor Neurone Disease Association
Movember Foundation
MPN Voice
Multi Organ Transplant Support
Multiple Births Foundation
Multiple Sclerosis UK
Myasthenia Gravis Association
Myrovlytis Trust
NARA - The Breathing Charity
National Association for Pre-Menstrual Syndrome
National Cancer Research Institute
National Eczema Society

For peer review only

National Kidney Federation
National Obesity Forum
National Osteoporosis Society
NMO Research Foundation
North of England Bone Marrow and Thalassaemia Association
Northern Ireland Chest, Heart and Stroke
Northern Ireland Rare Disease Partnership
OcuMel UK
Olive Tree Cancer Support Centre - Crawley
Oliver King Foundation
Orchid
Organisation for Sickle Cell Anaemia Research and Thalassaemia Support
Ovacome
Paget's Association
Pain Association Scotland
Pain Concern
Pain UK
Patients Association
Patients On Intravenous and Nasogastric Nutrition Therapy
Paula Carr Diabetes Trust
Pelican Cancer Foundation
Pelvic Pain Support Network
Pink Ribbon Foundation – Breast Cancer Charity
Polycystic Kidney Disease Charity
Portsmouth Down Syndrome Association
Positive Action
Prader-Willi Syndrome Association UK
Progressive Supranuclear Palsy Association
Prostate Cancer Research Centre
Prostate Cymru
QUIT
Rainbow Centre for Conductive Education
Rainbow Trust Children's Charity
Rarer Cancers Foundation
Restless Leg Syndrome UK
Richmond Fellowship
River House Trust
Ronald McDonald House Charities
Roy Castle Lung Cancer Foundation
Royal Marsden Cancer Charity
Salamander Trust
Samson Centre for MS
SANE
Scoliosis Association UK
Scotland Patients Association
Scottish Drugs Forum
Scottish Epilepsy Initiative
Scottish Kidney Federation

For peer review only

Scottish Network Children with Arthrities
SeeAbility
Self Help Services
Sense
Service by Emergency Response Volunteers - Suffolk and Cambridgeshire
Short Bowel Survivor and Friends
Silver Star
Skin Care Cymru
Somerville Foundation
Sophia Forum
Spectra
Spinal Injuries Association
Spinal Injuries Scotland
St Thomas Lupus Trust
Steps
SUDEP Action
Sussex Beacon
Tackle Prostate Cancer
Team Margot
Tenovus Cancer Care
Thames Valley Positive Support
Thrombosis UK
Transplant Links
Transverse Myelitis Society
Tree of Hope
Trigeminal Neuralgia Association
UK Chronic Lymphocytic Leukaemia Forum
UK Gout Society
UK Sepsis Trust
Walk the Walk Worldwide
Waverley Care
Willow Foundation
World Cancer Research Fund
World Child Cancer
York Air Ambulance

For peer review only

Number of payments	Value of payments (2016 £)
1	1028.5
1	5070.3
1	101.0
12	47076.4
12	13947.1
1	2519.9
2	7940.8
1	3042.2
6	70151.6
1	129.6
2	39258.9
1	25250.0
1	262.3
2	21041.7
2	16456.2
5	138585.3
2	563.6
1	1057.3
1	303.0
6	38722.8
76	375206.6
1	1616.0
34	329518.1
32	262194.0
1	507.0
1	1014.1
1	1500.0
104	621144.3
1	3809.2
1	205.7
4	35201.1
1	5050.0
42	342673.1
1	20281.1
3	3193.8
3	4145.2
1	10000.0
38	2271217.4
1	10520.8
2	776.4
6	97945.7
3	12160.6
1	981.5
1	12675.7
1	262.3
7	81316.2

For peer review only

5	47893.8
8	55592.3
7	1881.6
1	420.8
1	0.0
6	9784.6
12	95961.9
1	243.4
4	9320.3
26	249099.2
13	29164.3
57	1817634.9
1	0.0
7	6270.9
5	2338.0
1	4089.1
1	1000.0
2	624.9
2	16010.0
1	736.5
1	252.5
12	86711.0
2	120.9
3	5412.1
1	1028.5
3	20369.3
1	4187.3
9	27581.2
9	24748.9
17	281196.7
1	1892.5
1	526.0
3	14664.9
1	2592.9
3	6594.9
4	1547.3
1	15150.0
5	10786.9
2	1384.1
2	804.5
2	767.3
2	4085.1
7	6116.9
1	507.0
6	26164.4
2	11930.2
1	153.6
2	6400.0

For peer review only

57	577521.6
10	13720.9
1	4000.0
1	150.0
1	37.6
1	757.5
1	5070.3
13	63461.6
1	2104.2
2	4156.5
1	4000.0
11	29891.2
1	30322.2
1	2000.0
10	91665.0
9	101336.2
37	183714.4
2	754.3
1	1010.0
1	353.5
4	22216.1
1	518.7
1	252.5
24	248299.0
1	0.0
4	55502.5
1	707.0
1	15.2
3	3412.9
2	764.6
1	883.8
1	202.0
3	643.0
1	15000.0
24	386833.4
7	34571.2
2	280.0
1	1010.0
6	44237.3
1	6084.3
1	505.0
1	603.4
1	2020.0
1	1420.3
1	0.0
1	152.1
1	1000.0
14	23675.7

For peer review only

30	396718.0
3	3508.5
35	326740.6
3	6650.0
4	5775.3
1	1028.5
2	4035.1
2	2080.6
1	3400.0
1	510.9
2	3334.2
2	4184.1
6	64181.1
3	24720.6
10	4636.2
11	16888.4
10	30100.6
48	345520.4
5	29848.9
1	400.0
1	8500.0
4	2654.7
1	10000.0
4	68956.0
1	151.5
1	0.0
1	473.4
3	563.8
1	1052.1
1	639.6
13	79339.8
1	579.3
1	1010.0
62	682970.9
5	12939.6
1	736.5
1	252.5
1	771.4
70	770325.1
1	354.9
2	720.7
1	4500.0
7	83686.8
1	277.7
1	1028.5
2	4056.2
2	78625.5
1	505.0

For peer review only

3	1139.3
1	473.4
2	514.3
1	151.5
1	210.4
1	224.0
12	117958.9
8	19924.8
3	7327.7
2	10120.3
1	1010.0
1	252.5
1	1052.1
4	62950.0
1	151.5
2	2334.0
1	1000.0
13	148469.6
1	5050.0
9	170502.5
2	5736.5
17	45782.5
2	1531.9
1	15150.0
1	151.5
3	4208.3
1	4056.2
3	38407.4
1	0.0
1	405.6
18	103557.2
1	257.6
1	5000.0
15	342275.9
1	0.0

For peer review only

Web Supplement 16. Shared ties in industry and patient organisation data based on the number and value

Ties calculated based on the number of payments

Total number of ties reported in industry data involving at least one payment
Total number of ties reported in patient organisation data involving at least one payment

Number of shared ties (donors and recipients are the same in industry and patient organisation data)
Shared ties as a share of all ties in industry data
Shared ties as a share of all ties in patient organisation data

Number of ties involving the same number of payments
Number of ties involving the same number of payments as a share of shared ties

Number of payments forming ties with the equal number of payments in both datasets
Payments forming ties with the equal number of payments in both datasets as a a share of all payments in in
Payments forming ties with the equal number of payments in both datasets as a a share of all payments in pa

Ties calculated based on the value of payments

Total number of ties reported in industry data involving at least one payment with value >£0
Total number of ties reported in patient data involving at least one payment with value >£0

Number of shared ties (donors and recipients are the same in industry and patient organisation data)
Shared ties as a share of all ties in industry data
Shared ties as a share of all ties in patient organisation data

Number of shared ties involving the same value of payments
Shared ties involving the same alue of payments as a share of shared ties

Value of payments in which donors and recipients provided the same value (2016 £)
Value of shared payments as a share of payments with values >£0 in industry data
Value of shared payments as a share of payments with values >£0 in patient organisation data

For peer review only

All years	2012	2013	2014	2015	2016
1101	369	366	432	479	523
626	252	276	293	319	287
428	110	122	146	168	160
38.9%	29.8%	33.3%	33.8%	35.1%	30.6%
68.4%	43.7%	44.2%	49.8%	52.7%	55.7%
87	45	66	69	89	64
20.3%	40.9%	54.1%	47.3%	53.0%	40.0%
162	47	69	79	97	73
3.8%	6.1%	9.8%	9.6%	10.4%	6.7%
9.8%	16.2%	21.2%	22.8%	26.9%	21.7%
1088	366	357	431	470	522
326	127	122	119	150	140
34	21	24	28	32	24
3.1%	5.7%	6.7%	6.5%	6.8%	4.6%
10.4%	16.5%	19.7%	23.5%	21.3%	17.1%
20	17	22	26	30	23
58.8%	81.0%	91.7%	92.9%	93.8%	95.8%
325,108.4	216,055.8	201,362.3	251,964.6	517,139.2	482,216.0
0.6%	2.8%	3.3%	2.9%	4.5%	2.4%
1.0%	6.2%	4.6%	4.8%	10.7%	3.2%

Web Supplement 17. Overlap between recipient lists reported by donors and patient organ

	Total number of donors with at least one payment	Minimum overlap with patient organisation data	Number of donors with minium overlap with patient organisation data	Donors with minium overlap with patient organisation data as a share of all donors	Number of donors with less than 50% overlap with patient organisation data	Donors with less than 50% overlap with patient organisation data as a share of all donors
All years	63	0.0%	6	9.5%	40	63.5%
2012	30	0.0%	7	23.3%	23	76.7%
2013	38	0.0%	9	23.7%	25	65.8%
2014	45	0.0%	12	26.7%	35	77.8%
2015	51	0.0%	11	21.6%	33	64.7%
2016	44	0.0%	10	22.7%	32	72.7%

For peer review only

isation data - yearly breakdown based on the number of paymen

Maximum overlap with patient organisation data	Number of donors with maximum overlap with patient organisation data	Donors with maximum overlap with patient organisation data as a share of all donors
100.0%	3	4.8%
100.0%	1	3.3%
100.0%	4	10.5%
100.0%	3	6.7%
100.0%	3	5.9%
100.0%	3	6.8%

For peer review only

Web Supplement 18. Overlap between recipient lists reported by donors and patient organ

	Number of donors with at least one payemnt with the value >£0	Minimum overlap with patient organisation data	Number of donors with minium overlap with patient organisation data	Donors with minium overlap with patient organisation data as a share of all donors	Number of donors with less than 50% overlap with patient organisation data	Donors with less than 50% overlap with patient organisation data as a share of all donors
All years	62	0.0%	17	27.4%	56	90.3%
2012	30	0.0%	13	43.3%	29	96.7%
2013	38	0.0%	18	47.4%	36	94.7%
2014	45	0.0%	26	57.8%	45	100.0%
2015	50	0.0%	21	42.0%	42	84.0%
2016	44	0.0%	15	34.1%	39	88.6%

For peer review only

isation data - yearly breakdown based on the value of payments

Maximum overlap with patient organisation data	Number of donors with maximum overlap with patient organisation data	Donors with maximum overlap with patient organisation data as a share of all donors
100.0%	2	3.2%
50.0%	1	3.3%
66.7%	1	2.6%
40.0%	1	2.2%
100.0%	1	2.0%
66.7%	1	2.3%

For peer review only

Web Supplement 19. Overlap between donor lists reported by recipeints and industry data

	Number of recipients with at least 1 payment	Minimum overlap with industry data	Number of recipients with minium overlap with industry data	Recipients with minium overlap with industry data as a share of all recipients	Number of recipients with less than 50% overlap with industry data	Recipients with less than 50% overlap with industry data as a share of all recipients
All years	200	0.0%	14	7.0%	32	16.0%
2012	103	0.0%	37	35.9%	51	49.5%
2013	102	0.0%	38	37.3%	50	49.0%
2014	116	0.0%	34	29.3%	49	42.2%
2015	126	0.0%	37	29.4%	47	37.3%
2016	117	0.0%	28	23.9%	40	34.2%

- yearly breakdown based on the number of payments

Maximum overlap with industry data	Number of recipients with maximum overlap with industry data	Recipeints with maximum overlap with industry data as a share of all recipients
100.0%	102	51.0%
100.0%	29	28.2%
100.0%	24	23.5%
100.0%	41	35.3%
100.0%	49	38.9%
100.0%	50	42.7%

For peer review only

Web Supplement 20. Overlap between donor lists eported by recipeints and industry data ·

	Number of patient organisations with at least one payemnt with the value >0	Minimum overlap with industry data	Number of recipients with minium overlap with industry data	Recipients with minium overlap with industry data as a share of all recipients	Number of recipients with less than 50% overlap with industry data	Recipients with less than 50% overlap with industry data as a share of all recipients
All years	121	0.0%	12	9.9%	18	14.9%
2012	57	0.0%	22	38.6%	30	52.6%
2013	51	0.0%	19	37.3%	26	51.0%
2014	58	0.0%	17	29.3%	21	36.2%
2015	72	0.0%	22	30.6%	26	36.1%
2016	57	0.0%	10	17.5%	15	26.3%

For peer review only

· yearly breakdown based on the value of payments

Maximum overlap with industry data	Number of recipients with maximum overlap with industry data	Recipeints with maximum overlap with industry data as a share of all recipients
100.0%	71	58.7%
100.0%	16	28.1%
100.0%	14	27.5%
100.0%	27	46.6%
100.0%	35	48.6%
100.0%	31	54.4%

For peer review only

STROBE Statement—Checklist of items that should be included in reports of ***cohort studies***

	Item No	Recommendation	Page No
**Title and abstract**	1	(*a*) Indicate the study's design with a commonly used term in the title or the abstract	4
		(*b*) Provide in the abstract an informative and balanced summary of what was done and what was found	4
**Introduction**			
Background/rationale	2	Explain the scientific background and rationale for the investigation being reported	5
Objectives	3	State specific objectives, including any prespecified hypotheses	5
**Methods**			
Study design	4	Present key elements of study design early in the paper	6
Setting	5	Describe the setting, locations, and relevant dates, including periods of recruitment, exposure, follow-up, and data collection	6
Participants	6	(*a*) Give the eligibility criteria, and the sources and methods of selection of participants. Describe methods of follow-up	6
		(*b*) For matched studies, give matching criteria and number of exposed and unexposed	N/A
Variables	7	Clearly define all outcomes, exposures, predictors, potential confounders, and effect modifiers. Give diagnostic criteria, if applicable	7
Data sources/ measurement	8*	For each variable of interest, give sources of data and details of methods of assessment (measurement). Describe comparability of assessment methods if there is more than one group	7
Bias	9	Describe any efforts to address potential sources of bias	6
Study size	10	Explain how the study size was arrived at	6
Quantitative variables	11	Explain how quantitative variables were handled in the analyses. If applicable, describe which groupings were chosen and why	7
Statistical methods	12	(*a*) Describe all statistical methods, including those used to control for confounding	7
		(*b*) Describe any methods used to examine subgroups and interactions	N/A
		(*c*) Explain how missing data were addressed	6
		(*d*) If applicable, explain how loss to follow-up was addressed	N/A
		(*e*) Describe any sensitivity analyses	N/A
**Results**			
Participants	13*	(a) Report numbers of individuals at each stage of study—eg numbers potentially eligible, examined for eligibility, confirmed eligible, included in the study, completing follow-up, and analysed	7
		(b) Give reasons for non-participation at each stage	N/A
		(c) Consider use of a flow diagram	Web supplement 1 and 2
Descriptive data	14*	(a) Give characteristics of study participants (eg demographic, clinical, social) and information on exposures and potential confounders	7-8
		(b) Indicate number of participants with missing data for each variable of interest	6,16
		(c) Summarise follow-up time (eg, average and total amount)	Table 1
Outcome data	15*	Report numbers of outcome events or summary measures over time	7-20

For peer review only

For peer review only - http://bmjopen.bmj.com/site/about/guidelines.xhtml

For peer review only

For peer review only - http://bmjopen.bmj.com/site/about/guidelines.xhtml

Main results	16	(*a*) Give unadjusted estimates and, if applicable, confounder-adjusted estimates and their precision (eg, 95% confidence interval). Make clear which confounders were adjusted for and why they were included	7-20
		(*b*) Report category boundaries when continuous variables were categorized	10-15
		(*c*) If relevant, consider translating estimates of relative risk into absolute risk for a meaningful time period	N/A
Other analyses	17	Report other analyses done—eg analyses of subgroups and interactions, and sensitivity analyses	N/A
**Discussion**			
Key results	18	Summarise key results with reference to study objectives	20-22
Limitations	19	Discuss limitations of the study, taking into account sources of potential bias or imprecision. Discuss both direction and magnitude of any potential bias	22-23
Interpretation	20	Give a cautious overall interpretation of results considering objectives, limitations, multiplicity of analyses, results from similar studies, and other relevant evidence	23
Generalisability	21	Discuss the generalisability (external validity) of the study results	22
**Other information**			
Funding	22	Give the source of funding and the role of the funders for the present study and, if applicable, for the original study on which the present article is based	2

*Give information separately for exposed and unexposed groups.

Note: An Explanation and Elaboration article discusses each checklist item and gives methodological background and published examples of transparent reporting. The STROBE checklist is best used in conjunction with this article (freely available on the Web sites of PLoS Medicine at http://www.plosmedicine.org/, Annals of Internal Medicine at http://www.annals.org/, and Epidemiology at http://www.epidem.com/). Information on the STROBE Initiative is available at http://www.strobe-statement.org.

For peer review only
